# Intelligence, education level, and risk of Parkinson’s disease in European populations: A Mendelian randomization study

**DOI:** 10.3389/fgene.2022.963163

**Published:** 2022-11-10

**Authors:** Jingjing Shi, Jie Tian, Yu Fan, Xiaoyan Hao, Mengjie Li, Jiadi Li, Dongrui Ma, Mengnan Guo, Shuangjie Li, Yuming Xu, Changhe Shi

**Affiliations:** ^1^ Department of Neurology, The First Affiliated Hospital of Zhengzhou University, Zhengzhou University, Zhengzhou, Henan, China; ^2^ Zheng Zhou Railway Vocational and Technical College, Zhengzhou, Henan, China; ^3^ Academy of Medical Sciences of Zhengzhou University, Zhengzhou, Henan, China; ^4^ Henan Key Laboratory of Cerebrovascular Diseases, The First Affiliated Hospital of Zhengzhou University, Zhengzhou University, Zhengzhou, Henan, China; ^5^ Institute of Neuroscience, Zhengzhou University, Zhengzhou, Henan, China

**Keywords:** Parkinson's disease, mendelian randomization, intelligence, educational attainment, cognitive performance

## Abstract

**Background:** A high level of education or intelligence (IQ) is reported to be a risk factor for Parkinson’s disease (PD). The purpose of this study was to systematically examine the causal relationships between IQ, educational attainment (EA), cognitive performance, and PD.

**Methods:** We used summary statistics from genome-wide association studies on IQ, EA, cognitive performance, and PD. Four genome-wide association study (GWAS) data for PD were used to comprehensively explore the causal relationship, including PD GWAS (regardless of sex), age at onset of PD GWAS, male with PD GWAS, and female with PD GWAS data. We conducted a two sample Mendelian randomization (MR) study using the inverse-variance weighted (IVW), weighted median, simple mode, and weighted mode methods to evaluate the causal association between these factors. MR-Egger and MR-PRESSO were used for sensitivity analysis to test and correct horizontal pleiotropy. Multivariate MR (MVMR) was also used to account for the covariation between IQ, EA, and cognition, as well as to explore potential mediating factors.

**Results:** Genetically predicted higher IQ was associated with an increased risk of PD in the entire population, regardless of gender. In the analyses using the IVW method, the odds ratio was 1.37 (*p* = 0.0064). Men with a higher IQ, more years of education, or stronger cognitive ability are more likely to develop PD compared to women. MVMR showed that adjusting for education and cognition largely attenuated the association between IQ and PD, suggesting that education and cognition may mediate the effect of IQ on PD.

**Conclusion:** This study provides genetic support for the causal link between higher IQ and an increased risk of PD.

## Introduction

Parkinson’s disease (PD) is the second most common neurodegenerative disease, with most PD cases occurring after the age of 60. PD is characterized by degeneration of dopaminergic neurons in the substantia nigra, and its clinical manifestations include motor symptoms, such as bradykinesia, tremor, and rigidity (Tolosa et al., 2006; De Virgilio et al., 2016). A high level of education is reported to be a risk factor for PD (Rocca et al., 1996; Frigerio et al., 2005; Park et al., 2005). Additionally, in one study, working with highly complex data was associated with an increased risk of PD (Valdés et al., 2014). Most studies on the link between PD and occupations and education have focused on the fact that some occupations may increase the risk of PD by exposure to environmental factors, such as exposure to toxins in agriculture or exposure to infections in the healthcare industry. For example, many pesticides have neurotoxic properties, and many pesticides containing 1-methyl,-4-phenyl-1,2,3,6-tetra hydropyridine (MPTP) metabolites can cause damage to human substantia nigra dopaminergic neurons. Numerous pesticides increase the risk of PD, mainly by affecting mitochondrial complex I (including rotenone) or causing oxidative stress (Langston et al., 1983; Weisskopf et al., 2010).

However, since intelligence (IQ) is a powerful predictor of education level and a person’s later work situation, cognitive performance may be partially responsible for the link between educational level and PD. The link between higher levels of education and PD may be due to higher levels of IQ. A recent cohort study based on a large population found that people with high IQ were more likely to develop PD (Fardell et al., 2020). However, direct assessments of the causal effects of IQ and education on PD are rarely performed. Thus, in this study, we conducted a two-sample Mendelian random (MR) analysis to examine the causal effects of IQ, educational attainment (EA), and cognitive performance on the risk of PD. MR can support the conversion of observed correlations into causality, offering a potentially robust approach while minimizing deviations caused by confusion and reverse causality (Pierce and Burgess, 2013; Davies et al., 2015; Burgess et al., 2017).

There is growing evidence that men have twice the risk of developing PD compared to women, but women have more rapid disease progression and higher mortality (Moisan et al., 2016; Vaidya et al., 2021). In summary, biological sex is an important factor affecting the occurrence and development of PD. Additionally, there is some evidence that the age at onset (AAO) may be different between males and females with PD (Georgiev et al., 2017). Therefore, we further explored the relationship between PD and intelligence, EA, and cognitive performance using age at onset of PD GWAS (PDAOO GWAS), male with PD GWAS (PDMMALE GWAS), and female with PD GWAS (PDFEMALE GWAS) data.

## Materials and methods

### Exposure

We selected several strong genetic variations, or single nucleotide polymorphisms (SNPs) (*p* < 5 × 10^–8^), which were only related to EA, cognitive (test) performance, or intelligence as instrumental variables (IVs) and applied them to the summary level results of PD case-control GWAS. We then applied these IVs to the summary results of the GWAS in PD cases and controls. We calculated the phenotypic variance of IQ, EA, and cognitive (test) performance explained by each SNP (*R* ^2^) using commonly used formulas: *R* ^2^ = 2 × *EAF* × (1 − *EAF*) × betaˆ2/(2 × *EAF* × (1 − *EAF*) × betaˆ2) + 2 × *EAF* × (1 − *EAF*) × *se* × *N* × betaˆ2) (Papadimitriou et al., 2020). Here, *EAF*, beta, *se*, and *N* represent the effect allele frequency, effect size, standard error, and sample size, respectively. We also calculated the *F*-statistic using formula F = *R* ^2^ (N−2)/(1−*R* ^2^) to assess the presence of weak instrumental variable bias. Here, *F* < 10 indicates a low power of the instrumental variable to explain the exposure. Our study had a very large sample size for each MR analysis and strong estimated effects for each variant (all SNP *F*-statistics > 10). Accordingly, the study had high statistical power to assess the potential associations between IQ, EA, and cognitive (test) performance and PD. Statistical power was calculated based on a 5% type I error using the publicly available tool mRnd (https://shiny.cnsgenomics.com/mRnd/).

A total of 165 approximately independent genome-wide significant (*p* < 5 × 10^–8^) SNPs were identified as associated with fluid intelligence based on intelligence GWAS from a recent genome-wide association study of multiple traits (*n* = 269,867) (Savage et al., 2018). This GWAS was sufficiently covariate adjusted (e.g., age, sex, ancestry principal components). The GWAS for EA is measured as the number of years of schooling that individuals have received. The sample was limited to individuals of European ancestry, and all association analyses were performed at the cohort level. A total of 317 approximately independent genome-wide significant (*p* > 5 × 10^–8^) SNPs were identified as being associated with years of schooling in a GWAS meta-analysis of 766,345 participants (Lee et al., 2018). Additionally, we reviewed GWAS for cognitive (test) performance (*n* = 257,841), a factor highly correlated with EA (Lee et al., 2018), as a meta-analysis of published COGENT consortium findings and results based on a new United Kingdom Biobank (UKB) analysis. We identified 147 genome-wide significant SNPs associated with cognitive performance. The *R* ^2^ and *F*-statistics for each SNP are shown in Supplementary Table S1. All of these instrumental variables have *F*-statistics > 10, suggesting strong IV strength for MR analyses.

### Outcomes

The PD GWAS dataset was from the International Consortium for Parkinson’s Disease Genomics (IPDGC). The PD GWAS dataset used in this study included 33,674 PD cases and 449,056 controls (Nalls et al., 2019). We also performed the largest PD AAO GWAS to date by including 28,568 PD cases (Blauwendraat et al., 2019). Additionally, male GWAS and female GWAS data, all derived from Illumina platform-based genotyping, were obtained from members of the IPDGC, collaborators, and publicly available datasets from UKB genotype data (version 3). The male PD GWAS (MALEPD GWAS) data included 13,020 male PD cases and 89,660 male controls. The female PD GWAS (FEMALEPD GWAS) data included 7,947 female PD cases and 90,662 female controls (Blauwendraat et al., 2021).

The subjects in both the exposure and outcome datasets included in this study were of European ancestry. No ethical approval was required, as our study was a secondary analysis of previously published data.

### Study design

Randomized controlled trials study the effect of a factor by randomizing subjects into a control group and an experimental group. However, in reality, it is very difficult to complete a randomized controlled trial, which requires a lot of manpower and material resources. Sometimes, due to ethical issues, it is almost impossible to study a certain factor. Other approaches are needed, and Mendelian randomization is one of them. MR core is use of Mendel’s second law, also is a free combination law, when two (or more), has been relatively characteristics of parents for hybridization, in child generation produces gametes, in the separation of alleles at the same time, on the same chromosome, gene expression for free combination, this process is similar to the random grouping, randomized controlled trial MR is a randomized controlled trial based on Mendel’s second law (Emdin et al., 2017). MR is an effective method for inferring the causal effects of one trait (phenotype) on another trait (disease risk). The exposures associated with SNPs were instrumental variables in the MR, and their associations with outcomes such as PD were identified. By combining SNP exposure with SNP outcome associations, MR infers whether exposure causes results (Bowden and Holmes, 2019).

**FIGURE 1:**
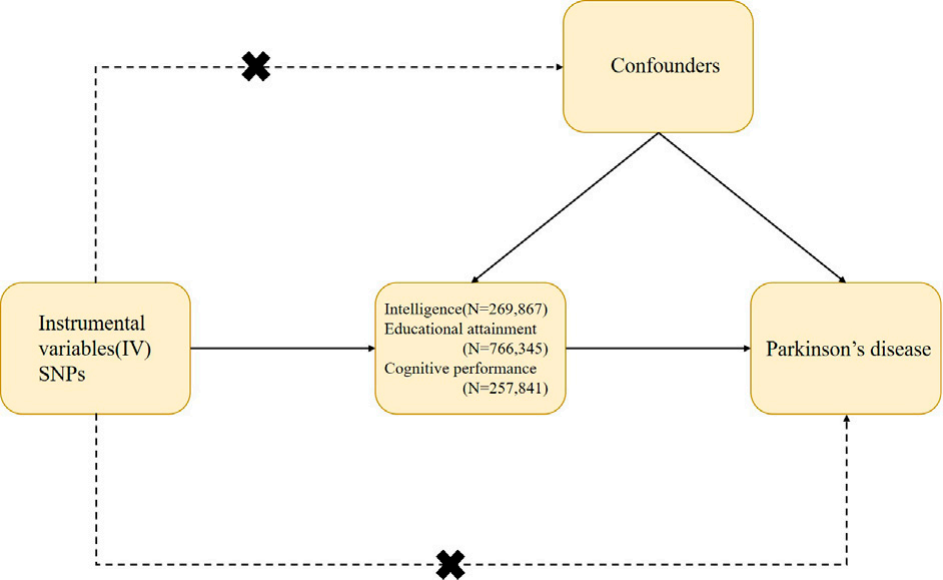
Design and main assumptions of our Mendelian randomization study. SNPs, single nucleotide polymorphisms. GWAS, genome-wide association study; PD, Parkinson’s disease.

We conducted a two-sample MR study to investigate the potential causal effects of IQ, EA, and cognitive (test) performance on PD risk. MR research is based on three main assumptions: 1) IVs are directly associated with exposure (IQ and EA) with genome-wide significance (relevance); 2) IVs should not be associated with confounding factors in the relationship between exposure and outcome in MR analyses (independence); and 3) IVs affect the outcome (exclusion restriction) simply by exposure. Figure 1 provides a schematic diagram of this study.

Using univariate MR to estimate the overall effect of IQ, EA, and cognitive performance (respectively) on PD risk through all possible pathways, we did not exclude the 19 SNPs that overlapped with the GWAS of IQ, EA, and cognition. Since IQ, EA, and cognition are covariates that correlate with each other, this univariate approach produces biased effect estimates (Sanderson et al., 2019). To avoid this problem, we adopted a multivariate MR approach to adjust for IQ, EA, and cognitive (test) performance and to explore potential mediating effects (Hemani et al., 2018). MVMR estimates the direct effect of each exposure on the outcome by accounting for pleiotropy among multiple characteristics.

### Statistical analysis

MR analysis was performed using the TwoSampleMR and MR-PRESSO packages in RStudio (version 4.2). Five MR analysis methods were used, including the inverse-variance weighted (IVW) (Burgess et al., 2015), weighted median (Bowden et al., 2016), MR-Egger (Bowden et al., 2015), simple mode, and weighted mode methods. The main analysis was a random-effects inverse IVW analysis. Simply put, random-effects IVW integrates estimates from each IV, assuming that all IVs are valid, and evaluates causality. There is no pleiotropic effect or invalid effect, so the overall pleiotropy is balanced to zero. Four methods were used to test the sensitivity: a pleiotropy test, a heterogeneity test, a leave-one-out sensitivity analysis, and an MR-PRESSO test (Ong and MacGregor, 2019). An MR-Egger regression was performed to test whether the results had directional pleiotropy. We also performed a Cochran’s *Q* test to assess the heterogeneity of each genetic variation estimate. To measure whether the combined estimates were disproportionately affected by each genetic variation (SNP) and whether the combined estimates were robust, we performed a leave-one-out sensitivity analysis. IVs can only affect the outcome through exposure; that is, there is no gene pleiotropy (Greenland, 2018). The existence of pleiotropy was tested using the intercept term of the MR Egger regression model (Burgess and Thompson, 2017). The intercept term was not 0 (*p* < 0.05), indicating the existence of gene pleiotropy. At the same time, the MR-Presso method was used to evaluate gene pleiotropy, and the estimated value was corrected by eliminating outliers (Verbanck et al., 2018). We used a Bonferroni correction [corrected *p* = 0.05/3 (exposures) = 0.0017] to account for multiple comparisons.

## Results

Genetic associations between all exposed SNPs and the four PD GWAS outcomes are shown in Supplementary Table S1. The results of the main MR analysis are shown in Figure 2 and Supplementary Table S2. Sensitivity analysis results for PD are shown in Supplementary Tables S3, S4. The MR Presso results for each analysis are presented in Supplementary Table S5. Supplementary Figures S1–S12 are scatter plots of SNPs associated with exposure and PD to each MR Analysis. Supplementary Figures S13–S24 are tree plots of individual SNP analyses in the MR Analysis. Supplementary Figures S25–S36 sequentially show plots of leave-one-out analyses in each MR Analysis.

**FIGURE 2:**
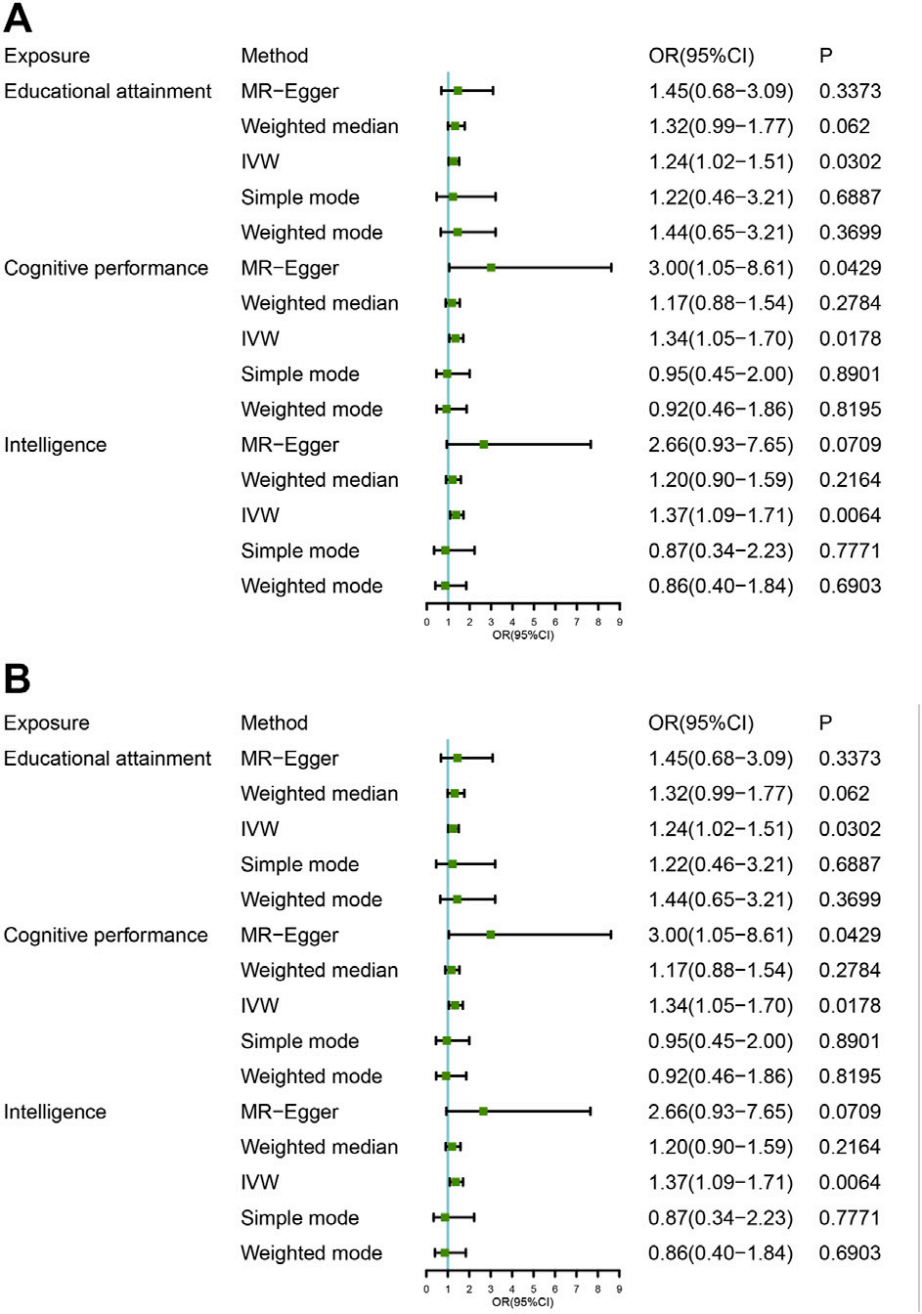
Mendelian randomization analysis of the association between intelligence educational attainment, cognitive performance, and Parkinson disease. OR, odds ratio; CI, confidential interval. **(A)** Parkinson GWAS data for outcomes (no gender difference); **(B)** PD GWAS data for outcomes were exclusively for male patients.

### MR effect of IQ, EA and cognitive performance on PD (no gender difference)

We used 140 independent variants to investigate the causal effect of IQ on PD risk (Supplementary Table S1). Genetically predicted higher IQ was associated with a higher risk of PD. The odds ratio was 1.37 (95% CI: 1.09–1.71, *p* = 0.0064) in the analyses using the IVW method. The results of the main MR analysis are shown in Table 1. Numerically, the relationship between PD and IQ did not reach statistical significance *via* the weighted median (*OR* = 0.20, *p* = 0.21), simple mode (*OR* = 0.87, *p* = 0.78), or weighted mode (*OR* = 0.86, *p* = 0.69) (Supplementary Table S2). These causal associations are presented in the scatter plot (Supplementary Figure S1). Forest plots of the effect of each single SNP on the PD GWAS are provided in Supplementary Figure S13. As seen in the forest plots, the results of individual SNPs may not be significant, and only when the results are combined can a reasonable result be obtained, which is the red line at the bottom, which reflects that high IQ increases the risk of PD under the IVW approach. The MR-Egger regression analysis did not find directional pleiotropy for IQ SNPs (intercept: −0.014, *p* = 0.21) (Supplementary Table S3). Because SNPs have high heterogeneity (Cochran’s *Q* test, *p* ＜ 0.05; Supplementary Table S4), we used a random effect model to estimate the effects of intelligence on PD risk (*p* = 0.042). After the elimination of abnormal SNPs by the MR-Presso Outlier test and the MR-Presso distortion test, the MR results were still statistically significant (*p* = 0.0058; Table 1). Furthermore, a leave-one-out analysis showed that the causal relationship between IQ and PD was not driven by any single SNP (Supplementary Figure S3). The leave-one-out analysis did not find any pleiotropy influenced by a single SNP, and it confirmed the associations. This suggests that people with a higher IQ are more likely to develop PD.

**TABLE 1:** Causal effect and sensitivity analysis results for Parkinson’s Disease.

Exposure	Outcome	SNPs	Outliers	IVW		MR-Egger		MR-Egger intercept		MR-PRESSO global	
		N	N	OR (95%CI)	*p* value	OR (95%CI)	*p* value	Intercept	*p* value	*p* value	Outlier-corrected
											*p* value
Intelligence	PD	140	2	1.37 (1.09–1.71)	**0.0064**	2.66 (0.93–7.65)	0.0709	-0.0138	0.2844	0.001	**0.0058**
Intelligence	PDMALE	144	2	1.39 (1.10–1.76)	**0.0054**	1.26 (0.40–3.94)	0.6916	0.0021	0.8582	<0.001	0.0039
Educational attainment	PD	286	2	1.24 (1.02–1.51)	0.0302	1.45 (0.68–3.09)	0.3373	-0.0022	0.3423	0.363	NA
Educational attainment	PDMALE	303	3	1.50 (1.18–1.92)	**0.0012**	1.24 (0.47–3.26)	0.6603	0.0027	0.6904	<0.001	0.0023
Cognitive performance	PD	132	0	1.34 (1.05–1.70)	0.0178	3.00 (1.05–8.61)	0.0429	-0.0174	0.8413	<0.001	0.0846
Cognitive performance	PDMALE	136	1	1.46 (1.12–1.89)	**0.0045**	2.63 (0.83–8.37)	0.1036	-0.0128	0.3057	<0.001	0.0096

Bold numbers indicate that *p* value is statistically significant.

IVW, inverse-variance weighted; MR-PRESSO, MR pleiotropy residual sum and outlier; OR, odds; CI, confidential; PD, Parkinson's disease (regardless of gender); PDMALE, PD among male.

We did not find significant associations between EA and PD using IVW (95% *CI*: 1.02–1.51, *p* = 0.030) and MR Egger (*p* = 0.3373). The weighted median (*p* = 0.0620) analyses, simple mode (*p* = 0.6887), and weighted mode (*p* = 0.3700) showed no evidence of a higher risk of PD (Table 1). These causal associations are presented in the scatter plot (Supplementary Figure S2). We used 132 independent variants (Supplementary Table S1) to investigate the impact of cognitive (test) performance on PD. The IVW results (IVW: OR = 1.34, 95% CI = 1.05–1.70, *p* = 0.0178) are consistent with the estimation direction of the MR-Egger and weighted median, and the *p-*value was not statistically significant (Supplementary Table S2). These causal associations are presented in the scatter plot (Supplementary Figure S3).

### MR effect of IQ, EA, and cognitive performance on PD among females and males, respectively

We used 144 independent variants to investigate the causal effect of IQ on the risk of PD among men. Genetically predicted higher IQ was associated with a higher risk of PD among males. The odds ratio was 1.37 (95% CI: 1.09–1.71, *p* = 0.0054) in the analyses using the IVW method. The results of the main MR analysis are shown in Table 1. The MR-Egger intercept test (*p* = 0.858) and MR-PRESSO global test (*p* < 0.001) were applied to detect the presence of pleiotropy. After the elimination of abnormal SNPs by the MR-Presso Outlier test and the MR-Presso distortion test, the MR results were still statistically significant (*p* = 0.0039; Table 1). We also observed (Table 1) that EA was related to PD among male, with a somewhat more moderate OR of 1.50 (95% CI: 1.18–1.92; *p* = 0.0012) per SD higher predicted EA. We used 136 independent variants (Supplementary Table S1) to investigate the causal effect of cognitive performance on the risk of PD among men. Genetically predicted higher cognitive performance was associated with a higher risk of PD among males. The odds ratio was 1.37 (95% CI: 1.09–1.71, *p* = 0.0046) in the analyses using the IVW method. The MR-Egger intercept test (*p* = 0.31) and MR-PRESSO global test (*p* < 0.001) were applied to detect the presence of pleiotropy. After the elimination of abnormal SNPs by the MR-Presso Outlier test and the MR-Presso Distortion test, the MR results were still statistically significant (*p* = 0.0096; Table 1). The results of leave-one-out analyses indicated that the genetically predicted associations between intelligence, education, cognition and PD were stable and not strongly driven by individual SNPSs (Supplementary Figures S28–S30). Taken together, there is clear evidence that higher IQ, EA or cognitive ability increase the risk of PD among males. The statistical analysis showed that there is no evidence that IQ (OR, 1.11; 95% CI, 0.87–1.41, 1.12; *p* = 0.39), education (OR, 1.29; 95% CI, 0.99–1.69; *p* = 0.06), or cognitive performance (OR, 1.36; 95% CI, 1.02–1.81, 1.12; *p* = 0.03) are causally related to PD among female.

### MR effect of IQ, EA, and cognitive performance on age at onset in PD patients

There is no evidence of a causal relationship between IQ (OR, 0.75; 95% CI, 0.23–2.48, 1.12; *p* = 0.64), EA (OR, 3.09; 95% CI, 0.88–11.10, 1.12; *p* = 0.08), cognitive ability (OR, 1.86; 95% CI, 0.58–6.02, 1.12; *p* = 0.30), and different AAO in PD patients.

### MVMR effect of IQ, EA, and cognitive performance on PD

Our MVMR studies showed that the association between IQ and PD was greatly attenuated after adjustment for EA and cognitive performance (Supplementary Table S6), both in the overall PD population and in the male PD patients.

## Discussion

We conducted univariate and multivariable MR analysis using large summary statistical GWASs to explore the causal relationship between EA, cognitive performance, IQ, and PD. The results of MR analysis provide genetic support for a causal relationship between higher IQ and increased risk of PD, especially in the male population, where the risk is more pronounced. The effects of EA and cognitive (test) performance on PD were found only in the male group.

After adjusting for EA and cognitive performance by multivariate MR, the effect of IQ on PD disappeared. Genetically predicated intelligence *per se* was not independently associated with PD. The discrepancy between the univariate and MVMR assessments suggests that the effect of IQ on PD is potentially mediated; that is, IQ impacts PD through its effect on educational achievement or cognitive performance rather than by having a direct effect on PD. A recent meta-analysis demonstrated that the relationship between IQ and EA probably runs both ways (Ritchie and Tucker-Drob, 2018). The study, which involved more than 600,000 participants, found that education increased IQ by about 1–5 points (Ritchie and Tucker-Drob, 2018). Therefore, the effect of a high IQ on PD risk may be partly related to EA.

Our findings are consistent with those of previous case-control studies (Fardell et al., 2020). It is important to determine the underlying scientific link between a higher IQ and a higher risk of PD. While little is known about the underlying mechanisms, the following points make the link between IQ and PD credible (Frigerio et al., 2005). First, the relationship between a higher IQ and a higher risk of PD may be related to lifestyle. Studies have found that people with higher IQs or education levels have lower cholesterol (Lara and Amigo, 2018), a factor that has been linked to an increased risk of PD (de Lau et al., 2006; Huang et al., 2008; Gudala et al., 2013; Huang et al., 2015; García-Sanz et al., 2021; Lv et al., 2022). People with higher IQ or education levels are also less likely to smoke (Wennerstad et al., 2010; Modig et al., 2011; Pärna et al., 2014), and abstinence from smoking is a known protective factor against PD (Breckenridge et al., 2016; Delamarre and Meissner, 2017). The influence of IQ or education level on cholesterol may be related to economic level and living habits (Lara and Amigo, 2018). Adults with less education are more likely to smoke because of psychological factors, such as low self-esteem and stress, or because of tobacco advertising. The main reasons they have more difficulty quitting may be that they lack motivation, social support, and adequate resources to purchase nicotine replacement products (McEwen et al., 1997; Hiscock et al., 2012). The mediating role of these factors in the relationship between IQ and PD cannot be completely denied, and further studies are needed.

Second, the causal association between a higher IQ and an increased risk of PD may be related to occupational complexity. Some studies have found that people (especially men) with more complex jobs, such as data analysis and processing, have a higher risk of PD. Jobs involving higher levels of career complexity, such as senior management and professorial roles, require more mental activity and may also involve higher levels of stress (McEwen et al., 1997; Wood et al., 2004). Stress at work can lead to elevated glutamate levels (Wood et al., 2004), which have been linked to PD (Mitchell et al., 1989). Third, there is a number of potential genetic or biological association between IQ and PD. For example, CDC42, the corresponding gene of rs10917152, is associated with PD (Ying et al., 2022). It has been shown that impaired CDC42 signaling regulated by dopamine D2 receptors leads to spine loss and behavioral deficits in PD (Ying et al., 2022). *ESSRG* (a transcription factor estrogen-related receptor gamma), the corresponding gene of rs10779271, deficiency leads to the reduction of genes related to synaptic, mitochondrial function, and autophagy, which causes PD (Fox et al., 2022). Some PD cognitive symptoms may be related to primary neurodegeneration in cortical and higher cognitive areas, or may be mediated by abnormal neural activity (McGregor and Nelson, 2019).

This study has several strengths. Its first major strength is that it utilized three types of large-exposure GWAS data and four types of PD outcome GWAS data to explore these complex associations. This is the most detailed and comprehensive joint investigation of the effects of IQ, education, and cognition on the risk of developing PD to date. By using randomly assigned genetic variants as instrumental variables, our study largely mitigated the effects of confounding factors, thereby greatly reducing bias and providing convincing evidence. Second, the study had high statistical power to assess the potential association of exposure with PD (calculated to be close to 100%); this was possible because of the large sample size for each MR analysis of exposure and the outcome of GWAS, as well the strong estimated effect of each genetic variable (all *F*-statistics > 10).

Our study also has several limitations. First, we did not have data on other risk factors for PD, such as occupational and family income, family social status, and lifestyle (Frigerio et al., 2005). The development of PD is related to metabolic disorders, including obesity, hypertension, diabetes, and hyperlipidemia, which are greatly influenced by lifestyle and socioeconomic factors. Education level and IQ level also influence the occurrence of metabolic diseases through socioeconomic factors. Although the causal relationship between IQ and PD is unlikely to be significantly confounded by these factors, as MR analyses are less susceptible to confounding than observational studies, further investigation is needed into the potential role of these factors in the causal association between IQ and PD. Second, the participants in this study were all European, so the inferred causality may only apply to Europeans, which could affect the generalizations of the MR study.

## Data Availability

The summary statistics of IQ GWAS can be downloaded under https://gwas.mrcieu.ac.uk/datasets/ebi-a-GCST006250/. The summary statistics of EA GWAS can be downloaded under https://gwas.mrcieu.ac.uk/datasets/ieu-a-1239/. The summary statistics of cognitive performance GWAS can be downloaded under https://gwas.mrcieu.ac.uk/datasets/ebi-a-GCST006572/. The summary statistics of PD GWAS by Nalls MA et al. can be downloaded under https://gwas.mrcieu.ac.uk/datasets/ieu-b-7/. The summary statistics of PDAOO GWAS by Blauwendraat C et al. can be downloaded under https://pdgenetics.org/resources. The summary statistics of Male and Female specific PD GWAS can be downloaded https://pdgenetics.org/resources. The code for this study has been uploaded https://github.com/Anonymousxyee/TwoSampleMR-IQtoPD/blob/main/IQtoPD.R.
