# Supplementary material for: Intelligence, education level, and risk of Parkinson’s disease in European populations: A Mendelian randomization study
Source: Front Genet. 2022 Nov 10;13:963163. doi: 10.3389/fgene.2022.963163 (PMC9684183; doi:10.3389/fgene.2022.963163)
Supplement: Supplementary file 1 [file DataSheet2.docx]

**Supplementary Information: Supplementary Table**

| Table | Contents | Page |
| --- | --- | --- |
| Table 1 | Summary of genetic variants used to estimate the effect of exposure and Parkinson's disease risk. | 2 |
| Table 2 | Causal effect of exposure on Parkinson's disease. | 135 |
| Table 3 | Pleiotropy analysis for the causal effect of exposure and Parkinson's disease risk. | 138 |
| Table 4 | Heterogeneity analysis of the association between genetically predicted exposure and Parkinson's disease risk. | 138 |
| Table 5 | MR-presso for the causal effect of exposure and Parkinson disease risk. | 139 |
| Table 6 | Multivariable MR associations of IQ, education and cognitive performance with PD. | 141 |

| | **Supplementary Table 1.** Summary of genetic variants used to estimate the effect of exposure and Parkinson's disease risk. | | | | | | | | | | --- | --- | --- | --- | --- | --- | --- | --- | --- | |  | SNP | beta.exposure | eaf.exposure | pval.outcome | outcome | exposure | R | F | | 1 | rs1009950 | -0.01903 | 0.4184 | 0.6721 | FEMALEPD | Cognitive performance || id:ebi-a-GCST006572 | 0.001321922 | 341.294281 | | 2 | rs10129426 | 0.01926 | 0.5306 | 0.6249 | FEMALEPD | Cognitive performance || id:ebi-a-GCST006572 | 0.001349521 | 348.4293531 | | 3 | rs10189857 | -0.02289 | 0.4184 | 0.0167 | FEMALEPD | Cognitive performance || id:ebi-a-GCST006572 | 0.001344841 | 347.2195289 | | 4 | rs10191758 | 0.01965 | 0.3810 | 0.6958 | FEMALEPD | Cognitive performance || id:ebi-a-GCST006572 | 0.001326443 | 342.463097 | | 5 | rs10497818 | -0.01715 | 0.4303 | 0.9792 | FEMALEPD | Cognitive performance || id:ebi-a-GCST006572 | 0.001344841 | 347.2195289 | | 6 | rs1064608 | -0.01884 | 0.3639 | 0.3508 | FEMALEPD | Cognitive performance || id:ebi-a-GCST006572 | 0.00129543 | 334.4455663 | | 7 | rs10865397 | 0.01675 | 0.5221 | 0.9168 | FEMALEPD | Cognitive performance || id:ebi-a-GCST006572 | 0.001340194 | 346.0180773 | | 8 | rs10874938 | -0.02364 | 0.5068 | 0.2172 | FEMALEPD | Cognitive performance || id:ebi-a-GCST006572 | 0.001358978 | 350.8744713 | | 9 | rs10875914 | 0.02373 | 0.3554 | 0.0474 | FEMALEPD | Cognitive performance || id:ebi-a-GCST006572 | 0.001330996 | 343.6399461 | | 10 | rs10990610 | 0.02498 | 0.1650 | 0.0243 | FEMALEPD | Cognitive performance || id:ebi-a-GCST006572 | 0.001006352 | 259.738245 | | 11 | rs11079849 | 0.01996 | 0.3078 | 0.7733 | FEMALEPD | Cognitive performance || id:ebi-a-GCST006572 | 0.001269978 | 327.8663093 | | 12 | rs11117646 | -0.02248 | 0.1905 | 0.7367 | FEMALEPD | Cognitive performance || id:ebi-a-GCST006572 | 0.001079157 | 278.5493714 | | 13 | rs11123820 | 0.0243 | 0.4065 | 0.8545 | FEMALEPD | Cognitive performance || id:ebi-a-GCST006572 | 0.001326443 | 342.463097 | | 14 | rs11138947 | 0.01737 | 0.7330 | 0.1582 | FEMALEPD | Cognitive performance || id:ebi-a-GCST006572 | 0.001218124 | 314.4629696 | | 15 | rs11210871 | 0.01917 | 0.6701 | 0.0590 | FEMALEPD | Cognitive performance || id:ebi-a-GCST006572 | 0.001241521 | 320.5103344 | | 16 | rs11259916 | 0.01744 | 0.7330 | 0.6507 | FEMALEPD | Cognitive performance || id:ebi-a-GCST006572 | 0.001225824 | 316.4532415 | | 17 | rs112780312 | -0.02121 | 0.2534 | 0.5779 | FEMALEPD | Cognitive performance || id:ebi-a-GCST006572 | 0.001206754 | 311.5240633 | | 18 | rs1144593 | 0.02276 | 0.2993 | 0.6530 | FEMALEPD | Cognitive performance || id:ebi-a-GCST006572 | 0.001253559 | 323.6220852 | | 19 | rs11662271 | -0.0232 | 0.5153 | 0.2238 | FEMALEPD | Cognitive performance || id:ebi-a-GCST006572 | 0.001358978 | 350.8744713 | | 20 | rs11693702 | -0.02045 | 0.4490 | 0.0961 | FEMALEPD | Cognitive performance || id:ebi-a-GCST006572 | 0.001354233 | 349.6476375 | | 21 | rs11720523 | 0.01622 | 0.4456 | 0.7121 | FEMALEPD | Cognitive performance || id:ebi-a-GCST006572 | 0.001344841 | 347.2195289 | | 22 | rs11793831 | 0.02693 | 0.4354 | 0.1836 | FEMALEPD | Cognitive performance || id:ebi-a-GCST006572 | 0.001335579 | 344.8249115 | | 23 | rs12435486 | -0.01823 | 0.2483 | 0.7388 | FEMALEPD | Cognitive performance || id:ebi-a-GCST006572 | 0.001170338 | 302.1124602 | | 24 | rs12439619 | 0.01994 | 0.3129 | 0.3990 | FEMALEPD | Cognitive performance || id:ebi-a-GCST006572 | 0.001261715 | 325.7303724 | | 25 | rs12441495 | -0.02575 | 0.1361 | 0.9134 | FEMALEPD | Cognitive performance || id:ebi-a-GCST006572 | 0.000913871 | 235.8472272 | | 26 | rs12448902 | -0.026 | 0.3197 | 0.0189 | FEMALEPD | Cognitive performance || id:ebi-a-GCST006572 | 0.001326443 | 342.463097 | | 27 | rs12535854 | 0.01783 | 0.6667 | 0.0727 | FEMALEPD | Cognitive performance || id:ebi-a-GCST006572 | 0.001274151 | 328.9448169 | | 28 | rs12536800 | 0.01939 | 0.2806 | 0.0029 | FEMALEPD | Cognitive performance || id:ebi-a-GCST006572 | 0.001206754 | 311.5240633 | | 29 | rs12635303 | 0.01849 | 0.2925 | 0.8384 | FEMALEPD | Cognitive performance || id:ebi-a-GCST006572 | 0.001253559 | 323.6220852 | | 30 | rs12773747 | 0.0205 | 0.2262 | 0.0603 | FEMALEPD | Cognitive performance || id:ebi-a-GCST006572 | 0.001166817 | 301.2024829 | | 31 | rs13107325 | -0.05434 | 0.0901 | 0.0040 | FEMALEPD | Cognitive performance || id:ebi-a-GCST006572 | 0.000715053 | 184.5004139 | | 32 | rs13120565 | 0.01825 | 0.6429 | 0.4374 | FEMALEPD | Cognitive performance || id:ebi-a-GCST006572 | 0.001317432 | 340.1334161 | | 33 | rs13163336 | 0.0317 | 0.1735 | 0.2535 | FEMALEPD | Cognitive performance || id:ebi-a-GCST006572 | 0.000990925 | 255.7524919 | | 34 | rs13253386 | 0.01836 | 0.5051 | 0.5305 | FEMALEPD | Cognitive performance || id:ebi-a-GCST006572 | 0.001358978 | 350.8744713 | | 35 | rs136554 | 0.01586 | 0.4592 | 0.7638 | FEMALEPD | Cognitive performance || id:ebi-a-GCST006572 | 0.001344841 | 347.2195289 | | 36 | rs1391438 | -0.01691 | 0.6854 | 0.8732 | FEMALEPD | Cognitive performance || id:ebi-a-GCST006572 | 0.001265833 | 326.7948507 | | 37 | rs1408579 | 0.0168 | 0.5000 | 0.4833 | FEMALEPD | Cognitive performance || id:ebi-a-GCST006572 | 0.001354233 | 349.6476375 | | 38 | rs1415802 | 0.01718 | 0.4031 | 0.6342 | FEMALEPD | Cognitive performance || id:ebi-a-GCST006572 | 0.001321922 | 341.294281 | | 39 | rs1479073 | -0.01731 | 0.6888 | 0.6099 | FEMALEPD | Cognitive performance || id:ebi-a-GCST006572 | 0.001257624 | 324.6728063 | | 40 | rs148696809 | 0.0406 | 0.0816 | 0.0334 | FEMALEPD | Cognitive performance || id:ebi-a-GCST006572 | 0.000870782 | 224.717358 | | 41 | rs1507010 | 0.01728 | 0.4949 | 0.7364 | FEMALEPD | Cognitive performance || id:ebi-a-GCST006572 | 0.001354233 | 349.6476375 | | 42 | rs1523048 | -0.01776 | 0.6003 | 0.9793 | FEMALEPD | Cognitive performance || id:ebi-a-GCST006572 | 0.001308542 | 337.8352173 | | 43 | rs1567154 | -0.02045 | 0.2653 | 0.5539 | FEMALEPD | Cognitive performance || id:ebi-a-GCST006572 | 0.001110044 | 286.5307287 | | 44 | rs159428 | -0.01676 | 0.5068 | 0.6683 | FEMALEPD | Cognitive performance || id:ebi-a-GCST006572 | 0.001340194 | 346.0180773 | | 45 | rs17002025 | 0.02886 | 0.1378 | 0.2497 | FEMALEPD | Cognitive performance || id:ebi-a-GCST006572 | 0.000892833 | 230.4129593 | | 46 | rs17049085 | -0.02602 | 0.1105 | 0.4381 | FEMALEPD | Cognitive performance || id:ebi-a-GCST006572 | 0.000880669 | 227.2709644 | | 47 | rs17106817 | -0.01854 | 0.2840 | 0.4563 | FEMALEPD | Cognitive performance || id:ebi-a-GCST006572 | 0.00121052 | 312.497576 | | 48 | rs17428810 | -0.01788 | 0.2687 | 0.3574 | FEMALEPD | Cognitive performance || id:ebi-a-GCST006572 | 0.00124952 | 322.578143 | | 49 | rs1812587 | -0.01594 | 0.4456 | 0.2588 | FEMALEPD | Cognitive performance || id:ebi-a-GCST006572 | 0.001349521 | 348.4293531 | | 50 | rs1892419 | -0.0276 | 0.2330 | 0.8860 | FEMALEPD | Cognitive performance || id:ebi-a-GCST006572 | 0.001146128 | 295.8556933 | | 51 | rs1906252 | 0.03071 | 0.4966 | 0.0390 | FEMALEPD | Cognitive performance || id:ebi-a-GCST006572 | 0.001358978 | 350.8744713 | | 52 | rs2005078 | -0.01924 | 0.6667 | 0.7695 | FEMALEPD | Cognitive performance || id:ebi-a-GCST006572 | 0.001274151 | 328.9448169 | | 53 | rs2143103 | 0.02382 | 0.1446 | 0.1118 | FEMALEPD | Cognitive performance || id:ebi-a-GCST006572 | 0.000933672 | 240.9619863 | | 54 | rs2180111 | -0.01812 | 0.7058 | 0.7536 | FEMALEPD | Cognitive performance || id:ebi-a-GCST006572 | 0.00121052 | 312.497576 | | 55 | rs2239647 | 0.02093 | 0.5459 | 0.4843 | FEMALEPD | Cognitive performance || id:ebi-a-GCST006572 | 0.001340194 | 346.0180773 | | 56 | rs2295499 | -0.0191 | 0.4439 | 0.0088 | FEMALEPD | Cognitive performance || id:ebi-a-GCST006572 | 0.001349521 | 348.4293531 | | 57 | rs2352974 | -0.0319 | 0.5000 | 0.1041 | FEMALEPD | Cognitive performance || id:ebi-a-GCST006572 | 0.001358978 | 350.8744713 | | 58 | rs2426132 | 0.027 | 0.4728 | 0.1134 | FEMALEPD | Cognitive performance || id:ebi-a-GCST006572 | 0.001354233 | 349.6476375 | | 59 | rs2439649 | 0.01605 | 0.5391 | 0.0513 | FEMALEPD | Cognitive performance || id:ebi-a-GCST006572 | 0.001354233 | 349.6476375 | | 60 | rs2478281 | -0.02273 | 0.7534 | 0.5730 | FEMALEPD | Cognitive performance || id:ebi-a-GCST006572 | 0.001188267 | 306.7460869 | | 61 | rs26046 | -0.02044 | 0.3673 | 0.1216 | FEMALEPD | Cognitive performance || id:ebi-a-GCST006572 | 0.001326443 | 342.463097 | | 62 | rs2647995 | 0.01861 | 0.2925 | 0.9025 | FEMALEPD | Cognitive performance || id:ebi-a-GCST006572 | 0.001218124 | 314.4629696 | | 63 | rs2652454 | 0.01587 | 0.5221 | 0.0989 | FEMALEPD | Cognitive performance || id:ebi-a-GCST006572 | 0.001349521 | 348.4293531 | | 64 | rs2721173 | -0.01661 | 0.4490 | 0.8413 | FEMALEPD | Cognitive performance || id:ebi-a-GCST006572 | 0.001354233 | 349.6476375 | | 65 | rs2737339 | 0.01827 | 0.4099 | 0.3850 | FEMALEPD | Cognitive performance || id:ebi-a-GCST006572 | 0.001326443 | 342.463097 | | 66 | rs276626 | -0.02075 | 0.1616 | 0.9321 | FEMALEPD | Cognitive performance || id:ebi-a-GCST006572 | 0.00101958 | 263.1558535 | | 67 | rs2799399 | -0.01572 | 0.4167 | 0.2851 | FEMALEPD | Cognitive performance || id:ebi-a-GCST006572 | 0.001344841 | 347.2195289 | | 68 | rs2806048 | 0.01897 | 0.3571 | 0.8399 | FEMALEPD | Cognitive performance || id:ebi-a-GCST006572 | 0.001312972 | 338.9804215 | | 69 | rs2836921 | 0.01632 | 0.3248 | 0.1358 | FEMALEPD | Cognitive performance || id:ebi-a-GCST006572 | 0.001299771 | 335.5678669 | | 70 | rs2852931 | 0.02842 | 0.8486 | 0.7224 | FEMALEPD | Cognitive performance || id:ebi-a-GCST006572 | 0.000880669 | 227.2709644 | | 71 | rs287883 | 0.01858 | 0.3112 | 0.7805 | FEMALEPD | Cognitive performance || id:ebi-a-GCST006572 | 0.001233623 | 318.4688673 | | 72 | rs297589 | 0.01715 | 0.6684 | 0.6630 | FEMALEPD | Cognitive performance || id:ebi-a-GCST006572 | 0.001241521 | 320.5103344 | | 73 | rs2977464 | 0.02091 | 0.1684 | 0.2070 | FEMALEPD | Cognitive performance || id:ebi-a-GCST006572 | 0.001061436 | 273.9704776 | | 74 | rs3113262 | -0.01963 | 0.4932 | 0.8097 | FEMALEPD | Cognitive performance || id:ebi-a-GCST006572 | 0.001354233 | 349.6476375 | | 75 | rs3128341 | 0.03343 | 0.8248 | 0.5254 | FEMALEPD | Cognitive performance || id:ebi-a-GCST006572 | 0.00109748 | 283.2839216 | | 76 | rs335426 | -0.01973 | 0.5000 | 0.3839 | FEMALEPD | Cognitive performance || id:ebi-a-GCST006572 | 0.001344841 | 347.2195289 | | 77 | rs34802460 | 0.01917 | 0.2568 | 0.9719 | FEMALEPD | Cognitive performance || id:ebi-a-GCST006572 | 0.001103726 | 284.898075 | | 78 | rs34811474 | 0.02761 | 0.2296 | 0.6502 | FEMALEPD | Cognitive performance || id:ebi-a-GCST006572 | 0.001119658 | 289.0150992 | | 79 | rs35526560 | -0.01958 | 0.5765 | 0.3111 | FEMALEPD | Cognitive performance || id:ebi-a-GCST006572 | 0.001358978 | 350.8744713 | | 80 | rs35853157 | 0.0172 | 0.3163 | 0.3078 | FEMALEPD | Cognitive performance || id:ebi-a-GCST006572 | 0.001274151 | 328.9448169 | | 81 | rs3735478 | 0.02279 | 0.2857 | 0.1510 | FEMALEPD | Cognitive performance || id:ebi-a-GCST006572 | 0.001229711 | 317.457855 | | 82 | rs3740422 | -0.02553 | 0.3486 | 0.4497 | FEMALEPD | Cognitive performance || id:ebi-a-GCST006572 | 0.00129543 | 334.4455663 | | 83 | rs3860537 | -0.01861 | 0.7585 | 0.7245 | FEMALEPD | Cognitive performance || id:ebi-a-GCST006572 | 0.001152943 | 297.6167391 | | 84 | rs39302 | 0.02088 | 0.8078 | 0.7783 | FEMALEPD | Cognitive performance || id:ebi-a-GCST006572 | 0.001088241 | 280.8966976 | | 85 | rs3943667 | -0.0185 | 0.7228 | 0.7951 | FEMALEPD | Cognitive performance || id:ebi-a-GCST006572 | 0.001237559 | 319.4863397 | | 86 | rs4342312 | -0.01793 | 0.3741 | 0.2239 | FEMALEPD | Cognitive performance || id:ebi-a-GCST006572 | 0.001317432 | 340.1334161 | | 87 | rs4347883 | 0.01595 | 0.4388 | 0.7917 | FEMALEPD | Cognitive performance || id:ebi-a-GCST006572 | 0.001349521 | 348.4293531 | | 88 | rs4463213 | 0.02183 | 0.5408 | 0.3271 | FEMALEPD | Cognitive performance || id:ebi-a-GCST006572 | 0.001349521 | 348.4293531 | | 89 | rs4470366 | 0.01815 | 0.5612 | 0.8472 | FEMALEPD | Cognitive performance || id:ebi-a-GCST006572 | 0.001335579 | 344.8249115 | | 90 | rs4744250 | 0.01834 | 0.3520 | 0.3890 | FEMALEPD | Cognitive performance || id:ebi-a-GCST006572 | 0.001291117 | 333.3307478 | | 91 | rs4937860 | -0.02588 | 0.0935 | 0.6180 | FEMALEPD | Cognitive performance || id:ebi-a-GCST006572 | 0.000835155 | 215.5155697 | | 92 | rs4976976 | 0.01978 | 0.3861 | 0.4131 | FEMALEPD | Cognitive performance || id:ebi-a-GCST006572 | 0.001326443 | 342.463097 | | 93 | rs56135595 | -0.02216 | 0.1412 | 0.7869 | FEMALEPD | Cognitive performance || id:ebi-a-GCST006572 | 0.000968651 | 249.9980608 | | 94 | rs56290130 | -0.01692 | 0.3231 | 0.4572 | FEMALEPD | Cognitive performance || id:ebi-a-GCST006572 | 0.001274151 | 328.9448169 | | 95 | rs5751191 | -0.02214 | 0.4779 | 0.6986 | FEMALEPD | Cognitive performance || id:ebi-a-GCST006572 | 0.001354233 | 349.6476375 | | 96 | rs5757670 | 0.02009 | 0.6582 | 0.2830 | FEMALEPD | Cognitive performance || id:ebi-a-GCST006572 | 0.001257624 | 324.6728063 | | 97 | rs58489175 | 0.02121 | 0.2823 | 0.4769 | FEMALEPD | Cognitive performance || id:ebi-a-GCST006572 | 0.001225824 | 316.4532415 | | 98 | rs602512 | 0.01948 | 0.3861 | 0.0026 | FEMALEPD | Cognitive performance || id:ebi-a-GCST006572 | 0.001321922 | 341.294281 | | 99 | rs61815057 | 0.01681 | 0.3963 | 0.1341 | FEMALEPD | Cognitive performance || id:ebi-a-GCST006572 | 0.001326443 | 342.463097 | | 100 | rs62047970 | 0.01879 | 0.4218 | 0.9446 | FEMALEPD | Cognitive performance || id:ebi-a-GCST006572 | 0.001330996 | 343.6399461 | | 101 | rs62065449 | -0.02626 | 0.2007 | 0.0000 | FEMALEPD | Cognitive performance || id:ebi-a-GCST006572 | 0.001049942 | 271.0006079 | | 102 | rs620729 | 0.01912 | 0.3146 | 0.2479 | FEMALEPD | Cognitive performance || id:ebi-a-GCST006572 | 0.001274151 | 328.9448169 | | 103 | rs62169190 | -0.02043 | 0.1769 | 0.9315 | FEMALEPD | Cognitive performance || id:ebi-a-GCST006572 | 0.001052792 | 271.7370226 | | 104 | rs6509441 | 0.01876 | 0.2313 | 0.1822 | FEMALEPD | Cognitive performance || id:ebi-a-GCST006572 | 0.001139394 | 294.1153657 | | 105 | rs6535809 | -0.01956 | 0.5000 | 0.9424 | FEMALEPD | Cognitive performance || id:ebi-a-GCST006572 | 0.001358978 | 350.8744713 | | 106 | rs6550835 | -0.02479 | 0.3027 | 0.7499 | FEMALEPD | Cognitive performance || id:ebi-a-GCST006572 | 0.001274151 | 328.9448169 | | 107 | rs6587843 | 0.01774 | 0.4711 | 0.0943 | FEMALEPD | Cognitive performance || id:ebi-a-GCST006572 | 0.001358978 | 350.8744713 | | 108 | rs66752974 | -0.02214 | 0.1582 | 0.2974 | FEMALEPD | Cognitive performance || id:ebi-a-GCST006572 | 0.00100897 | 260.4146467 | | 109 | rs6708515 | -0.01671 | 0.6207 | 0.8951 | FEMALEPD | Cognitive performance || id:ebi-a-GCST006572 | 0.001299771 | 335.5678669 | | 110 | rs6718450 | 0.01698 | 0.4252 | 0.0792 | FEMALEPD | Cognitive performance || id:ebi-a-GCST006572 | 0.001308542 | 337.8352173 | | 111 | rs6798941 | 0.01794 | 0.2857 | 0.4262 | FEMALEPD | Cognitive performance || id:ebi-a-GCST006572 | 0.001237559 | 319.4863397 | | 112 | rs6819372 | 0.01865 | 0.5357 | 0.7198 | FEMALEPD | Cognitive performance || id:ebi-a-GCST006572 | 0.001354233 | 349.6476375 | | 113 | rs6860626 | -0.02149 | 0.1412 | 0.5129 | FEMALEPD | Cognitive performance || id:ebi-a-GCST006572 | 0.001030416 | 265.9553839 | | 114 | rs6903716 | -0.01842 | 0.2738 | 0.3779 | FEMALEPD | Cognitive performance || id:ebi-a-GCST006572 | 0.001233623 | 318.4688673 | | 115 | rs6952104 | 0.01864 | 0.5221 | 0.1659 | FEMALEPD | Cognitive performance || id:ebi-a-GCST006572 | 0.001358978 | 350.8744713 | | 116 | rs6975134 | 0.02194 | 0.4218 | 0.3901 | FEMALEPD | Cognitive performance || id:ebi-a-GCST006572 | 0.001330996 | 343.6399461 | | 117 | rs702222 | -0.02002 | 0.3282 | 0.9238 | FEMALEPD | Cognitive performance || id:ebi-a-GCST006572 | 0.001304142 | 336.697725 | | 118 | rs7044246 | 0.01748 | 0.6956 | 0.1694 | FEMALEPD | Cognitive performance || id:ebi-a-GCST006572 | 0.001245508 | 321.5409142 | | 119 | rs7256776 | -0.01864 | 0.2908 | 0.3596 | FEMALEPD | Cognitive performance || id:ebi-a-GCST006572 | 0.001274151 | 328.9448169 | | 120 | rs72739469 | 0.03577 | 0.0544 | 0.8002 | FEMALEPD | Cognitive performance || id:ebi-a-GCST006572 | 0.00062616 | 161.5496354 | | 121 | rs72821233 | -0.0201 | 0.2721 | 0.8307 | FEMALEPD | Cognitive performance || id:ebi-a-GCST006572 | 0.00119929 | 309.5951218 | | 122 | rs7312770 | -0.016 | 0.5136 | 0.5718 | FEMALEPD | Cognitive performance || id:ebi-a-GCST006572 | 0.001349521 | 348.4293531 | | 123 | rs73189617 | 0.01854 | 0.3299 | 0.0813 | FEMALEPD | Cognitive performance || id:ebi-a-GCST006572 | 0.001229711 | 317.457855 | | 124 | rs73845427 | -0.0466 | 0.0289 | 0.4776 | FEMALEPD | Cognitive performance || id:ebi-a-GCST006572 | 0.000480359 | 123.9147761 | | 125 | rs73989053 | -0.02211 | 0.1616 | 0.9504 | FEMALEPD | Cognitive performance || id:ebi-a-GCST006572 | 0.001016907 | 262.4651557 | | 126 | rs74370218 | -0.018 | 0.3827 | 0.7959 | FEMALEPD | Cognitive performance || id:ebi-a-GCST006572 | 0.001312972 | 338.9804215 | | 127 | rs7573001 | -0.01647 | 0.4150 | 0.3028 | FEMALEPD | Cognitive performance || id:ebi-a-GCST006572 | 0.001312972 | 338.9804215 | | 128 | rs7588384 | -0.01891 | 0.3129 | 0.3989 | FEMALEPD | Cognitive performance || id:ebi-a-GCST006572 | 0.00120301 | 310.5565973 | | 129 | rs75973558 | -0.02463 | 0.1190 | 0.7493 | FEMALEPD | Cognitive performance || id:ebi-a-GCST006572 | 0.000909585 | 234.7399632 | | 130 | rs7626560 | 0.02393 | 0.1361 | 0.2960 | FEMALEPD | Cognitive performance || id:ebi-a-GCST006572 | 0.000996014 | 257.0674147 | | 131 | rs77128898 | -0.04626 | 0.0221 | 0.8367 | FEMALEPD | Cognitive performance || id:ebi-a-GCST006572 | 0.00050474 | 130.2073233 | | 132 | rs78358737 | 0.04248 | 0.0340 | 0.7420 | FEMALEPD | Cognitive performance || id:ebi-a-GCST006572 | 0.000564217 | 145.5592785 | | 133 | rs78382112 | 0.03682 | 0.0578 | 0.3035 | FEMALEPD | Cognitive performance || id:ebi-a-GCST006572 | 0.000606574 | 156.4933088 | | 134 | rs7963801 | -0.02296 | 0.5476 | 0.3410 | FEMALEPD | Cognitive performance || id:ebi-a-GCST006572 | 0.001326443 | 342.463097 | | 135 | rs80170948 | -0.04589 | 0.0527 | 0.5556 | FEMALEPD | Cognitive performance || id:ebi-a-GCST006572 | 0.000549041 | 141.6419608 | | 136 | rs8054299 | 0.02556 | 0.3316 | 0.6550 | FEMALEPD | Cognitive performance || id:ebi-a-GCST006572 | 0.001265833 | 326.7948507 | | 137 | rs8058881 | 0.01831 | 0.2500 | 0.0387 | FEMALEPD | Cognitive performance || id:ebi-a-GCST006572 | 0.001159839 | 299.3988752 | | 138 | rs830383 | -0.01952 | 0.6310 | 0.8291 | FEMALEPD | Cognitive performance || id:ebi-a-GCST006572 | 0.001312972 | 338.9804215 | | 139 | rs875361 | -0.01623 | 0.4490 | 0.3762 | FEMALEPD | Cognitive performance || id:ebi-a-GCST006572 | 0.001349521 | 348.4293531 | | 140 | rs889169 | 0.01743 | 0.5867 | 0.1731 | FEMALEPD | Cognitive performance || id:ebi-a-GCST006572 | 0.001291117 | 333.3307478 | | 141 | rs9384679 | -0.02822 | 0.4082 | 0.5388 | FEMALEPD | Cognitive performance || id:ebi-a-GCST006572 | 0.001312972 | 338.9804215 | | 142 | rs9436866 | 0.03309 | 0.0952 | 0.7578 | FEMALEPD | Cognitive performance || id:ebi-a-GCST006572 | 0.000805662 | 207.8985953 | | 143 | rs991871 | 0.0243 | 0.7551 | 0.0903 | FEMALEPD | Cognitive performance || id:ebi-a-GCST006572 | 0.001149525 | 296.7336033 | | 144 | rs9930063 | 0.01633 | 0.4592 | 0.4160 | FEMALEPD | Cognitive performance || id:ebi-a-GCST006572 | 0.001344841 | 347.2195289 | | 1 | rs1009950 | -0.01903 | 0.4184 | 0.9781 | MALEPD | Cognitive performance || id:ebi-a-GCST006572 | 0.001321922 | 341.294281 | | 2 | rs10129426 | 0.01926 | 0.5306 | 0.4853 | MALEPD | Cognitive performance || id:ebi-a-GCST006572 | 0.001349521 | 348.4293531 | | 3 | rs10189857 | -0.02289 | 0.4184 | 0.7026 | MALEPD | Cognitive performance || id:ebi-a-GCST006572 | 0.001344841 | 347.2195289 | | 4 | rs10191758 | 0.01965 | 0.3810 | 0.2072 | MALEPD | Cognitive performance || id:ebi-a-GCST006572 | 0.001326443 | 342.463097 | | 5 | rs10497818 | -0.01715 | 0.4303 | 0.0012 | MALEPD | Cognitive performance || id:ebi-a-GCST006572 | 0.001344841 | 347.2195289 | | 6 | rs1064608 | -0.01884 | 0.3639 | 0.7033 | MALEPD | Cognitive performance || id:ebi-a-GCST006572 | 0.00129543 | 334.4455663 | | 7 | rs10865397 | 0.01675 | 0.5221 | 0.5234 | MALEPD | Cognitive performance || id:ebi-a-GCST006572 | 0.001340194 | 346.0180773 | | 8 | rs10874938 | -0.02364 | 0.5068 | 0.5404 | MALEPD | Cognitive performance || id:ebi-a-GCST006572 | 0.001358978 | 350.8744713 | | 9 | rs10875914 | 0.02373 | 0.3554 | 0.0199 | MALEPD | Cognitive performance || id:ebi-a-GCST006572 | 0.001330996 | 343.6399461 | | 10 | rs10990610 | 0.02498 | 0.1650 | 0.3169 | MALEPD | Cognitive performance || id:ebi-a-GCST006572 | 0.001006352 | 259.738245 | | 11 | rs11079849 | 0.01996 | 0.3078 | 0.6272 | MALEPD | Cognitive performance || id:ebi-a-GCST006572 | 0.001269978 | 327.8663093 | | 12 | rs11117646 | -0.02248 | 0.1905 | 0.4724 | MALEPD | Cognitive performance || id:ebi-a-GCST006572 | 0.001079157 | 278.5493714 | | 13 | rs11123820 | 0.0243 | 0.4065 | 0.5226 | MALEPD | Cognitive performance || id:ebi-a-GCST006572 | 0.001326443 | 342.463097 | | 14 | rs11138947 | 0.01737 | 0.7330 | 0.2994 | MALEPD | Cognitive performance || id:ebi-a-GCST006572 | 0.001218124 | 314.4629696 | | 15 | rs11210871 | 0.01917 | 0.6701 | 0.0652 | MALEPD | Cognitive performance || id:ebi-a-GCST006572 | 0.001241521 | 320.5103344 | | 16 | rs11259916 | 0.01744 | 0.7330 | 0.9077 | MALEPD | Cognitive performance || id:ebi-a-GCST006572 | 0.001225824 | 316.4532415 | | 17 | rs112780312 | -0.02121 | 0.2534 | 0.0089 | MALEPD | Cognitive performance || id:ebi-a-GCST006572 | 0.001206754 | 311.5240633 | | 18 | rs1144593 | 0.02276 | 0.2993 | 0.6321 | MALEPD | Cognitive performance || id:ebi-a-GCST006572 | 0.001253559 | 323.6220852 | | 19 | rs11662271 | -0.0232 | 0.5153 | 0.5052 | MALEPD | Cognitive performance || id:ebi-a-GCST006572 | 0.001358978 | 350.8744713 | | 20 | rs11693702 | -0.02045 | 0.4490 | 0.5938 | MALEPD | Cognitive performance || id:ebi-a-GCST006572 | 0.001354233 | 349.6476375 | | 21 | rs11720523 | 0.01622 | 0.4456 | 0.0096 | MALEPD | Cognitive performance || id:ebi-a-GCST006572 | 0.001344841 | 347.2195289 | | 22 | rs11793831 | 0.02693 | 0.4354 | 0.7457 | MALEPD | Cognitive performance || id:ebi-a-GCST006572 | 0.001335579 | 344.8249115 | | 23 | rs12435486 | -0.01823 | 0.2483 | 0.3146 | MALEPD | Cognitive performance || id:ebi-a-GCST006572 | 0.001170338 | 302.1124602 | | 24 | rs12439619 | 0.01994 | 0.3129 | 0.7840 | MALEPD | Cognitive performance || id:ebi-a-GCST006572 | 0.001261715 | 325.7303724 | | 25 | rs12441495 | -0.02575 | 0.1361 | 0.2744 | MALEPD | Cognitive performance || id:ebi-a-GCST006572 | 0.000913871 | 235.8472272 | | 26 | rs12448902 | -0.026 | 0.3197 | 0.2303 | MALEPD | Cognitive performance || id:ebi-a-GCST006572 | 0.001326443 | 342.463097 | | 27 | rs12535854 | 0.01783 | 0.6667 | 0.0427 | MALEPD | Cognitive performance || id:ebi-a-GCST006572 | 0.001274151 | 328.9448169 | | 28 | rs12536800 | 0.01939 | 0.2806 | 0.1032 | MALEPD | Cognitive performance || id:ebi-a-GCST006572 | 0.001206754 | 311.5240633 | | 29 | rs12635303 | 0.01849 | 0.2925 | 0.3713 | MALEPD | Cognitive performance || id:ebi-a-GCST006572 | 0.001253559 | 323.6220852 | | 30 | rs12773747 | 0.0205 | 0.2262 | 0.8216 | MALEPD | Cognitive performance || id:ebi-a-GCST006572 | 0.001166817 | 301.2024829 | | 31 | rs13107325 | -0.05434 | 0.0901 | 0.0631 | MALEPD | Cognitive performance || id:ebi-a-GCST006572 | 0.000715053 | 184.5004139 | | 32 | rs13120565 | 0.01825 | 0.6429 | 0.9587 | MALEPD | Cognitive performance || id:ebi-a-GCST006572 | 0.001317432 | 340.1334161 | | 33 | rs13163336 | 0.0317 | 0.1735 | 0.8591 | MALEPD | Cognitive performance || id:ebi-a-GCST006572 | 0.000990925 | 255.7524919 | | 34 | rs13253386 | 0.01836 | 0.5051 | 0.5013 | MALEPD | Cognitive performance || id:ebi-a-GCST006572 | 0.001358978 | 350.8744713 | | 35 | rs136554 | 0.01586 | 0.4592 | 0.5403 | MALEPD | Cognitive performance || id:ebi-a-GCST006572 | 0.001344841 | 347.2195289 | | 36 | rs1391438 | -0.01691 | 0.6854 | 0.6165 | MALEPD | Cognitive performance || id:ebi-a-GCST006572 | 0.001265833 | 326.7948507 | | 37 | rs1408579 | 0.0168 | 0.5000 | 0.5540 | MALEPD | Cognitive performance || id:ebi-a-GCST006572 | 0.001354233 | 349.6476375 | | 38 | rs1415802 | 0.01718 | 0.4031 | 0.5187 | MALEPD | Cognitive performance || id:ebi-a-GCST006572 | 0.001321922 | 341.294281 | | 39 | rs1479073 | -0.01731 | 0.6888 | 0.0507 | MALEPD | Cognitive performance || id:ebi-a-GCST006572 | 0.001257624 | 324.6728063 | | 40 | rs148696809 | 0.0406 | 0.0816 | 0.4680 | MALEPD | Cognitive performance || id:ebi-a-GCST006572 | 0.000870782 | 224.717358 | | 41 | rs1507010 | 0.01728 | 0.4949 | 0.1096 | MALEPD | Cognitive performance || id:ebi-a-GCST006572 | 0.001354233 | 349.6476375 | | 42 | rs1523048 | -0.01776 | 0.6003 | 0.1521 | MALEPD | Cognitive performance || id:ebi-a-GCST006572 | 0.001308542 | 337.8352173 | | 43 | rs1567154 | -0.02045 | 0.2653 | 0.1000 | MALEPD | Cognitive performance || id:ebi-a-GCST006572 | 0.001110044 | 286.5307287 | | 44 | rs159428 | -0.01676 | 0.5068 | 0.7582 | MALEPD | Cognitive performance || id:ebi-a-GCST006572 | 0.001340194 | 346.0180773 | | 45 | rs17002025 | 0.02886 | 0.1378 | 0.9469 | MALEPD | Cognitive performance || id:ebi-a-GCST006572 | 0.000892833 | 230.4129593 | | 46 | rs17049085 | -0.02602 | 0.1105 | 0.9284 | MALEPD | Cognitive performance || id:ebi-a-GCST006572 | 0.000880669 | 227.2709644 | | 47 | rs17106817 | -0.01854 | 0.2840 | 0.4474 | MALEPD | Cognitive performance || id:ebi-a-GCST006572 | 0.00121052 | 312.497576 | | 48 | rs17428810 | -0.01788 | 0.2687 | 0.0189 | MALEPD | Cognitive performance || id:ebi-a-GCST006572 | 0.00124952 | 322.578143 | | 49 | rs1812587 | -0.01594 | 0.4456 | 0.2708 | MALEPD | Cognitive performance || id:ebi-a-GCST006572 | 0.001349521 | 348.4293531 | | 50 | rs1892419 | -0.0276 | 0.2330 | 0.3153 | MALEPD | Cognitive performance || id:ebi-a-GCST006572 | 0.001146128 | 295.8556933 | | 51 | rs1906252 | 0.03071 | 0.4966 | 0.1229 | MALEPD | Cognitive performance || id:ebi-a-GCST006572 | 0.001358978 | 350.8744713 | | 52 | rs2005078 | -0.01924 | 0.6667 | 0.2423 | MALEPD | Cognitive performance || id:ebi-a-GCST006572 | 0.001274151 | 328.9448169 | | 53 | rs2143103 | 0.02382 | 0.1446 | 0.2292 | MALEPD | Cognitive performance || id:ebi-a-GCST006572 | 0.000933672 | 240.9619863 | | 54 | rs2180111 | -0.01812 | 0.7058 | 0.7925 | MALEPD | Cognitive performance || id:ebi-a-GCST006572 | 0.00121052 | 312.497576 | | 55 | rs2239647 | 0.02093 | 0.5459 | 0.2727 | MALEPD | Cognitive performance || id:ebi-a-GCST006572 | 0.001340194 | 346.0180773 | | 56 | rs2295499 | -0.0191 | 0.4439 | 0.1009 | MALEPD | Cognitive performance || id:ebi-a-GCST006572 | 0.001349521 | 348.4293531 | | 57 | rs2352974 | -0.0319 | 0.5000 | 0.7323 | MALEPD | Cognitive performance || id:ebi-a-GCST006572 | 0.001358978 | 350.8744713 | | 58 | rs2426132 | 0.027 | 0.4728 | 0.6545 | MALEPD | Cognitive performance || id:ebi-a-GCST006572 | 0.001354233 | 349.6476375 | | 59 | rs2439649 | 0.01605 | 0.5391 | 0.0146 | MALEPD | Cognitive performance || id:ebi-a-GCST006572 | 0.001354233 | 349.6476375 | | 60 | rs2478281 | -0.02273 | 0.7534 | 0.0922 | MALEPD | Cognitive performance || id:ebi-a-GCST006572 | 0.001188267 | 306.7460869 | | 61 | rs26046 | -0.02044 | 0.3673 | 0.3286 | MALEPD | Cognitive performance || id:ebi-a-GCST006572 | 0.001326443 | 342.463097 | | 62 | rs2647995 | 0.01861 | 0.2925 | 0.8459 | MALEPD | Cognitive performance || id:ebi-a-GCST006572 | 0.001218124 | 314.4629696 | | 63 | rs2652454 | 0.01587 | 0.5221 | 0.1737 | MALEPD | Cognitive performance || id:ebi-a-GCST006572 | 0.001349521 | 348.4293531 | | 64 | rs2721173 | -0.01661 | 0.4490 | 0.0457 | MALEPD | Cognitive performance || id:ebi-a-GCST006572 | 0.001354233 | 349.6476375 | | 65 | rs2737339 | 0.01827 | 0.4099 | 0.3495 | MALEPD | Cognitive performance || id:ebi-a-GCST006572 | 0.001326443 | 342.463097 | | 66 | rs276626 | -0.02075 | 0.1616 | 0.9161 | MALEPD | Cognitive performance || id:ebi-a-GCST006572 | 0.00101958 | 263.1558535 | | 67 | rs2799399 | -0.01572 | 0.4167 | 0.9491 | MALEPD | Cognitive performance || id:ebi-a-GCST006572 | 0.001344841 | 347.2195289 | | 68 | rs2806048 | 0.01897 | 0.3571 | 0.7937 | MALEPD | Cognitive performance || id:ebi-a-GCST006572 | 0.001312972 | 338.9804215 | | 69 | rs2836921 | 0.01632 | 0.3248 | 0.4842 | MALEPD | Cognitive performance || id:ebi-a-GCST006572 | 0.001299771 | 335.5678669 | | 70 | rs2852931 | 0.02842 | 0.8486 | 0.8705 | MALEPD | Cognitive performance || id:ebi-a-GCST006572 | 0.000880669 | 227.2709644 | | 71 | rs287883 | 0.01858 | 0.3112 | 0.6542 | MALEPD | Cognitive performance || id:ebi-a-GCST006572 | 0.001233623 | 318.4688673 | | 72 | rs297589 | 0.01715 | 0.6684 | 0.4407 | MALEPD | Cognitive performance || id:ebi-a-GCST006572 | 0.001241521 | 320.5103344 | | 73 | rs2977464 | 0.02091 | 0.1684 | 0.0885 | MALEPD | Cognitive performance || id:ebi-a-GCST006572 | 0.001061436 | 273.9704776 | | 74 | rs3113262 | -0.01963 | 0.4932 | 0.1660 | MALEPD | Cognitive performance || id:ebi-a-GCST006572 | 0.001354233 | 349.6476375 | | 75 | rs3128341 | 0.03343 | 0.8248 | 0.0792 | MALEPD | Cognitive performance || id:ebi-a-GCST006572 | 0.00109748 | 283.2839216 | | 76 | rs335426 | -0.01973 | 0.5000 | 0.9538 | MALEPD | Cognitive performance || id:ebi-a-GCST006572 | 0.001344841 | 347.2195289 | | 77 | rs34802460 | 0.01917 | 0.2568 | 0.1841 | MALEPD | Cognitive performance || id:ebi-a-GCST006572 | 0.001103726 | 284.898075 | | 78 | rs34811474 | 0.02761 | 0.2296 | 0.0088 | MALEPD | Cognitive performance || id:ebi-a-GCST006572 | 0.001119658 | 289.0150992 | | 79 | rs35526560 | -0.01958 | 0.5765 | 0.4057 | MALEPD | Cognitive performance || id:ebi-a-GCST006572 | 0.001358978 | 350.8744713 | | 80 | rs35853157 | 0.0172 | 0.3163 | 0.6792 | MALEPD | Cognitive performance || id:ebi-a-GCST006572 | 0.001274151 | 328.9448169 | | 81 | rs3735478 | 0.02279 | 0.2857 | 0.2959 | MALEPD | Cognitive performance || id:ebi-a-GCST006572 | 0.001229711 | 317.457855 | | 82 | rs3740422 | -0.02553 | 0.3486 | 0.3260 | MALEPD | Cognitive performance || id:ebi-a-GCST006572 | 0.00129543 | 334.4455663 | | 83 | rs3860537 | -0.01861 | 0.7585 | 0.8229 | MALEPD | Cognitive performance || id:ebi-a-GCST006572 | 0.001152943 | 297.6167391 | | 84 | rs39302 | 0.02088 | 0.8078 | 0.9906 | MALEPD | Cognitive performance || id:ebi-a-GCST006572 | 0.001088241 | 280.8966976 | | 85 | rs3943667 | -0.0185 | 0.7228 | 0.6519 | MALEPD | Cognitive performance || id:ebi-a-GCST006572 | 0.001237559 | 319.4863397 | | 86 | rs4342312 | -0.01793 | 0.3741 | 0.4450 | MALEPD | Cognitive performance || id:ebi-a-GCST006572 | 0.001317432 | 340.1334161 | | 87 | rs4347883 | 0.01595 | 0.4388 | 0.2163 | MALEPD | Cognitive performance || id:ebi-a-GCST006572 | 0.001349521 | 348.4293531 | | 88 | rs4463213 | 0.02183 | 0.5408 | 0.0081 | MALEPD | Cognitive performance || id:ebi-a-GCST006572 | 0.001349521 | 348.4293531 | | 89 | rs4470366 | 0.01815 | 0.5612 | 0.5509 | MALEPD | Cognitive performance || id:ebi-a-GCST006572 | 0.001335579 | 344.8249115 | | 90 | rs4744250 | 0.01834 | 0.3520 | 0.0509 | MALEPD | Cognitive performance || id:ebi-a-GCST006572 | 0.001291117 | 333.3307478 | | 91 | rs4937860 | -0.02588 | 0.0935 | 0.0747 | MALEPD | Cognitive performance || id:ebi-a-GCST006572 | 0.000835155 | 215.5155697 | | 92 | rs4976976 | 0.01978 | 0.3861 | 0.7407 | MALEPD | Cognitive performance || id:ebi-a-GCST006572 | 0.001326443 | 342.463097 | | 93 | rs56135595 | -0.02216 | 0.1412 | 0.8694 | MALEPD | Cognitive performance || id:ebi-a-GCST006572 | 0.000968651 | 249.9980608 | | 94 | rs56290130 | -0.01692 | 0.3231 | 0.6633 | MALEPD | Cognitive performance || id:ebi-a-GCST006572 | 0.001274151 | 328.9448169 | | 95 | rs5751191 | -0.02214 | 0.4779 | 0.8300 | MALEPD | Cognitive performance || id:ebi-a-GCST006572 | 0.001354233 | 349.6476375 | | 96 | rs5757670 | 0.02009 | 0.6582 | 0.8927 | MALEPD | Cognitive performance || id:ebi-a-GCST006572 | 0.001257624 | 324.6728063 | | 97 | rs58489175 | 0.02121 | 0.2823 | 0.0726 | MALEPD | Cognitive performance || id:ebi-a-GCST006572 | 0.001225824 | 316.4532415 | | 98 | rs602512 | 0.01948 | 0.3861 | 0.3995 | MALEPD | Cognitive performance || id:ebi-a-GCST006572 | 0.001321922 | 341.294281 | | 99 | rs61815057 | 0.01681 | 0.3963 | 0.5437 | MALEPD | Cognitive performance || id:ebi-a-GCST006572 | 0.001326443 | 342.463097 | | 100 | rs62047970 | 0.01879 | 0.4218 | 0.2715 | MALEPD | Cognitive performance || id:ebi-a-GCST006572 | 0.001330996 | 343.6399461 | | 101 | rs62065449 | -0.02626 | 0.2007 | 0.0000 | MALEPD | Cognitive performance || id:ebi-a-GCST006572 | 0.001049942 | 271.0006079 | | 102 | rs620729 | 0.01912 | 0.3146 | 0.6807 | MALEPD | Cognitive performance || id:ebi-a-GCST006572 | 0.001274151 | 328.9448169 | | 103 | rs62169190 | -0.02043 | 0.1769 | 0.9011 | MALEPD | Cognitive performance || id:ebi-a-GCST006572 | 0.001052792 | 271.7370226 | | 104 | rs6509441 | 0.01876 | 0.2313 | 0.5174 | MALEPD | Cognitive performance || id:ebi-a-GCST006572 | 0.001139394 | 294.1153657 | | 105 | rs6535809 | -0.01956 | 0.5000 | 0.3631 | MALEPD | Cognitive performance || id:ebi-a-GCST006572 | 0.001358978 | 350.8744713 | | 106 | rs6550835 | -0.02479 | 0.3027 | 0.6852 | MALEPD | Cognitive performance || id:ebi-a-GCST006572 | 0.001274151 | 328.9448169 | | 107 | rs6587843 | 0.01774 | 0.4711 | 0.1377 | MALEPD | Cognitive performance || id:ebi-a-GCST006572 | 0.001358978 | 350.8744713 | | 108 | rs66752974 | -0.02214 | 0.1582 | 0.0382 | MALEPD | Cognitive performance || id:ebi-a-GCST006572 | 0.00100897 | 260.4146467 | | 109 | rs6708515 | -0.01671 | 0.6207 | 0.2758 | MALEPD | Cognitive performance || id:ebi-a-GCST006572 | 0.001299771 | 335.5678669 | | 110 | rs6718450 | 0.01698 | 0.4252 | 0.4432 | MALEPD | Cognitive performance || id:ebi-a-GCST006572 | 0.001308542 | 337.8352173 | | 111 | rs6798941 | 0.01794 | 0.2857 | 0.1412 | MALEPD | Cognitive performance || id:ebi-a-GCST006572 | 0.001237559 | 319.4863397 | | 112 | rs6819372 | 0.01865 | 0.5357 | 0.8538 | MALEPD | Cognitive performance || id:ebi-a-GCST006572 | 0.001354233 | 349.6476375 | | 113 | rs6860626 | -0.02149 | 0.1412 | 0.2472 | MALEPD | Cognitive performance || id:ebi-a-GCST006572 | 0.001030416 | 265.9553839 | | 114 | rs6903716 | -0.01842 | 0.2738 | 0.4332 | MALEPD | Cognitive performance || id:ebi-a-GCST006572 | 0.001233623 | 318.4688673 | | 115 | rs6952104 | 0.01864 | 0.5221 | 0.7077 | MALEPD | Cognitive performance || id:ebi-a-GCST006572 | 0.001358978 | 350.8744713 | | 116 | rs6975134 | 0.02194 | 0.4218 | 0.6856 | MALEPD | Cognitive performance || id:ebi-a-GCST006572 | 0.001330996 | 343.6399461 | | 117 | rs702222 | -0.02002 | 0.3282 | 0.9837 | MALEPD | Cognitive performance || id:ebi-a-GCST006572 | 0.001304142 | 336.697725 | | 118 | rs7044246 | 0.01748 | 0.6956 | 0.6437 | MALEPD | Cognitive performance || id:ebi-a-GCST006572 | 0.001245508 | 321.5409142 | | 119 | rs7256776 | -0.01864 | 0.2908 | 0.4761 | MALEPD | Cognitive performance || id:ebi-a-GCST006572 | 0.001274151 | 328.9448169 | | 120 | rs72739469 | 0.03577 | 0.0544 | 0.0392 | MALEPD | Cognitive performance || id:ebi-a-GCST006572 | 0.00062616 | 161.5496354 | | 121 | rs72821233 | -0.0201 | 0.2721 | 0.2383 | MALEPD | Cognitive performance || id:ebi-a-GCST006572 | 0.00119929 | 309.5951218 | | 122 | rs7312770 | -0.016 | 0.5136 | 0.9896 | MALEPD | Cognitive performance || id:ebi-a-GCST006572 | 0.001349521 | 348.4293531 | | 123 | rs73189617 | 0.01854 | 0.3299 | 0.2702 | MALEPD | Cognitive performance || id:ebi-a-GCST006572 | 0.001229711 | 317.457855 | | 124 | rs73845427 | -0.0466 | 0.0289 | 0.8100 | MALEPD | Cognitive performance || id:ebi-a-GCST006572 | 0.000480359 | 123.9147761 | | 125 | rs73989053 | -0.02211 | 0.1616 | 0.3779 | MALEPD | Cognitive performance || id:ebi-a-GCST006572 | 0.001016907 | 262.4651557 | | 126 | rs74370218 | -0.018 | 0.3827 | 0.0128 | MALEPD | Cognitive performance || id:ebi-a-GCST006572 | 0.001312972 | 338.9804215 | | 127 | rs7573001 | -0.01647 | 0.4150 | 0.3453 | MALEPD | Cognitive performance || id:ebi-a-GCST006572 | 0.001312972 | 338.9804215 | | 128 | rs7588384 | -0.01891 | 0.3129 | 0.2999 | MALEPD | Cognitive performance || id:ebi-a-GCST006572 | 0.00120301 | 310.5565973 | | 129 | rs75973558 | -0.02463 | 0.1190 | 0.6131 | MALEPD | Cognitive performance || id:ebi-a-GCST006572 | 0.000909585 | 234.7399632 | | 130 | rs7626560 | 0.02393 | 0.1361 | 0.4855 | MALEPD | Cognitive performance || id:ebi-a-GCST006572 | 0.000996014 | 257.0674147 | | 131 | rs77128898 | -0.04626 | 0.0221 | 0.8310 | MALEPD | Cognitive performance || id:ebi-a-GCST006572 | 0.00050474 | 130.2073233 | | 132 | rs78358737 | 0.04248 | 0.0340 | 0.3741 | MALEPD | Cognitive performance || id:ebi-a-GCST006572 | 0.000564217 | 145.5592785 | | 133 | rs78382112 | 0.03682 | 0.0578 | 0.6948 | MALEPD | Cognitive performance || id:ebi-a-GCST006572 | 0.000606574 | 156.4933088 | | 134 | rs7963801 | -0.02296 | 0.5476 | 0.0494 | MALEPD | Cognitive performance || id:ebi-a-GCST006572 | 0.001326443 | 342.463097 | | 135 | rs80170948 | -0.04589 | 0.0527 | 0.2076 | MALEPD | Cognitive performance || id:ebi-a-GCST006572 | 0.000549041 | 141.6419608 | | 136 | rs8054299 | 0.02556 | 0.3316 | 0.2328 | MALEPD | Cognitive performance || id:ebi-a-GCST006572 | 0.001265833 | 326.7948507 | | 137 | rs8058881 | 0.01831 | 0.2500 | 0.5980 | MALEPD | Cognitive performance || id:ebi-a-GCST006572 | 0.001159839 | 299.3988752 | | 138 | rs830383 | -0.01952 | 0.6310 | 0.4022 | MALEPD | Cognitive performance || id:ebi-a-GCST006572 | 0.001312972 | 338.9804215 | | 139 | rs875361 | -0.01623 | 0.4490 | 0.1484 | MALEPD | Cognitive performance || id:ebi-a-GCST006572 | 0.001349521 | 348.4293531 | | 140 | rs889169 | 0.01743 | 0.5867 | 0.8358 | MALEPD | Cognitive performance || id:ebi-a-GCST006572 | 0.001291117 | 333.3307478 | | 141 | rs9384679 | -0.02822 | 0.4082 | 0.0463 | MALEPD | Cognitive performance || id:ebi-a-GCST006572 | 0.001312972 | 338.9804215 | | 142 | rs9436866 | 0.03309 | 0.0952 | 0.4310 | MALEPD | Cognitive performance || id:ebi-a-GCST006572 | 0.000805662 | 207.8985953 | | 143 | rs991871 | 0.0243 | 0.7551 | 0.7580 | MALEPD | Cognitive performance || id:ebi-a-GCST006572 | 0.001149525 | 296.7336033 | | 144 | rs9930063 | 0.01633 | 0.4592 | 0.6292 | MALEPD | Cognitive performance || id:ebi-a-GCST006572 | 0.001344841 | 347.2195289 | | 1 | rs1009950 | -0.01903 | 0.4184 | 0.8277 | PDAOO | Cognitive performance || id:ebi-a-GCST006572 | 0.001321922 | 341.294281 | | 2 | rs10129426 | 0.01926 | 0.5306 | 0.7930 | PDAOO | Cognitive performance || id:ebi-a-GCST006572 | 0.001349521 | 348.4293531 | | 3 | rs10189857 | -0.02289 | 0.4184 | 0.5668 | PDAOO | Cognitive performance || id:ebi-a-GCST006572 | 0.001344841 | 347.2195289 | | 4 | rs10191758 | 0.01965 | 0.3810 | 0.5898 | PDAOO | Cognitive performance || id:ebi-a-GCST006572 | 0.001326443 | 342.463097 | | 5 | rs10497818 | -0.01715 | 0.4303 | 0.5969 | PDAOO | Cognitive performance || id:ebi-a-GCST006572 | 0.001344841 | 347.2195289 | | 6 | rs1064608 | -0.01884 | 0.3639 | 0.0251 | PDAOO | Cognitive performance || id:ebi-a-GCST006572 | 0.00129543 | 334.4455663 | | 7 | rs10865397 | 0.01675 | 0.5221 | 0.6299 | PDAOO | Cognitive performance || id:ebi-a-GCST006572 | 0.001340194 | 346.0180773 | | 8 | rs10874938 | -0.02364 | 0.5068 | 0.7142 | PDAOO | Cognitive performance || id:ebi-a-GCST006572 | 0.001358978 | 350.8744713 | | 9 | rs10875914 | 0.02373 | 0.3554 | 0.0161 | PDAOO | Cognitive performance || id:ebi-a-GCST006572 | 0.001330996 | 343.6399461 | | 10 | rs10990610 | 0.02498 | 0.1650 | 0.2157 | PDAOO | Cognitive performance || id:ebi-a-GCST006572 | 0.001006352 | 259.738245 | | 11 | rs11079849 | 0.01996 | 0.3078 | 0.5566 | PDAOO | Cognitive performance || id:ebi-a-GCST006572 | 0.001269978 | 327.8663093 | | 12 | rs11117646 | -0.02248 | 0.1905 | 0.7455 | PDAOO | Cognitive performance || id:ebi-a-GCST006572 | 0.001079157 | 278.5493714 | | 13 | rs11123820 | 0.0243 | 0.4065 | 0.0889 | PDAOO | Cognitive performance || id:ebi-a-GCST006572 | 0.001326443 | 342.463097 | | 14 | rs11138947 | 0.01737 | 0.7330 | 0.2384 | PDAOO | Cognitive performance || id:ebi-a-GCST006572 | 0.001218124 | 314.4629696 | | 15 | rs11210871 | 0.01917 | 0.6701 | 0.9992 | PDAOO | Cognitive performance || id:ebi-a-GCST006572 | 0.001241521 | 320.5103344 | | 16 | rs11259916 | 0.01744 | 0.7330 | 0.5647 | PDAOO | Cognitive performance || id:ebi-a-GCST006572 | 0.001225824 | 316.4532415 | | 17 | rs112780312 | -0.02121 | 0.2534 | 0.3354 | PDAOO | Cognitive performance || id:ebi-a-GCST006572 | 0.001206754 | 311.5240633 | | 18 | rs1144593 | 0.02276 | 0.2993 | 0.4355 | PDAOO | Cognitive performance || id:ebi-a-GCST006572 | 0.001253559 | 323.6220852 | | 19 | rs11693702 | -0.02045 | 0.4490 | 0.0035 | PDAOO | Cognitive performance || id:ebi-a-GCST006572 | 0.001354233 | 349.6476375 | | 20 | rs11720523 | 0.01622 | 0.4456 | 0.1326 | PDAOO | Cognitive performance || id:ebi-a-GCST006572 | 0.001344841 | 347.2195289 | | 21 | rs11793831 | 0.02693 | 0.4354 | 0.5424 | PDAOO | Cognitive performance || id:ebi-a-GCST006572 | 0.001335579 | 344.8249115 | | 22 | rs12435486 | -0.01823 | 0.2483 | 0.3172 | PDAOO | Cognitive performance || id:ebi-a-GCST006572 | 0.001170338 | 302.1124602 | | 23 | rs12439619 | 0.01994 | 0.3129 | 0.3296 | PDAOO | Cognitive performance || id:ebi-a-GCST006572 | 0.001261715 | 325.7303724 | | 24 | rs12441495 | -0.02575 | 0.1361 | 0.9667 | PDAOO | Cognitive performance || id:ebi-a-GCST006572 | 0.000913871 | 235.8472272 | | 25 | rs12448902 | -0.026 | 0.3197 | 0.4368 | PDAOO | Cognitive performance || id:ebi-a-GCST006572 | 0.001326443 | 342.463097 | | 26 | rs12535854 | 0.01783 | 0.6667 | 0.4495 | PDAOO | Cognitive performance || id:ebi-a-GCST006572 | 0.001274151 | 328.9448169 | | 27 | rs12536800 | 0.01939 | 0.2806 | 0.2912 | PDAOO | Cognitive performance || id:ebi-a-GCST006572 | 0.001206754 | 311.5240633 | | 28 | rs12635303 | 0.01849 | 0.2925 | 0.7326 | PDAOO | Cognitive performance || id:ebi-a-GCST006572 | 0.001253559 | 323.6220852 | | 29 | rs12773747 | 0.0205 | 0.2262 | 0.0009 | PDAOO | Cognitive performance || id:ebi-a-GCST006572 | 0.001166817 | 301.2024829 | | 30 | rs13107325 | -0.05434 | 0.0901 | 0.4567 | PDAOO | Cognitive performance || id:ebi-a-GCST006572 | 0.000715053 | 184.5004139 | | 31 | rs13120565 | 0.01825 | 0.6429 | 0.9804 | PDAOO | Cognitive performance || id:ebi-a-GCST006572 | 0.001317432 | 340.1334161 | | 32 | rs13163336 | 0.0317 | 0.1735 | 0.2387 | PDAOO | Cognitive performance || id:ebi-a-GCST006572 | 0.000990925 | 255.7524919 | | 33 | rs13253386 | 0.01836 | 0.5051 | 0.1539 | PDAOO | Cognitive performance || id:ebi-a-GCST006572 | 0.001358978 | 350.8744713 | | 34 | rs136554 | 0.01586 | 0.4592 | 0.4110 | PDAOO | Cognitive performance || id:ebi-a-GCST006572 | 0.001344841 | 347.2195289 | | 35 | rs1391438 | -0.01691 | 0.6854 | 0.3170 | PDAOO | Cognitive performance || id:ebi-a-GCST006572 | 0.001265833 | 326.7948507 | | 36 | rs1408579 | 0.0168 | 0.5000 | 0.5804 | PDAOO | Cognitive performance || id:ebi-a-GCST006572 | 0.001354233 | 349.6476375 | | 37 | rs1415802 | 0.01718 | 0.4031 | 0.7435 | PDAOO | Cognitive performance || id:ebi-a-GCST006572 | 0.001321922 | 341.294281 | | 38 | rs1479073 | -0.01731 | 0.6888 | 0.6678 | PDAOO | Cognitive performance || id:ebi-a-GCST006572 | 0.001257624 | 324.6728063 | | 39 | rs148696809 | 0.0406 | 0.0816 | 0.5043 | PDAOO | Cognitive performance || id:ebi-a-GCST006572 | 0.000870782 | 224.717358 | | 40 | rs1507010 | 0.01728 | 0.4949 | 0.4443 | PDAOO | Cognitive performance || id:ebi-a-GCST006572 | 0.001354233 | 349.6476375 | | 41 | rs1523048 | -0.01776 | 0.6003 | 0.1293 | PDAOO | Cognitive performance || id:ebi-a-GCST006572 | 0.001308542 | 337.8352173 | | 42 | rs1567154 | -0.02045 | 0.2653 | 0.1924 | PDAOO | Cognitive performance || id:ebi-a-GCST006572 | 0.001110044 | 286.5307287 | | 43 | rs159428 | -0.01676 | 0.5068 | 0.2727 | PDAOO | Cognitive performance || id:ebi-a-GCST006572 | 0.001340194 | 346.0180773 | | 44 | rs17002025 | 0.02886 | 0.1378 | 0.4322 | PDAOO | Cognitive performance || id:ebi-a-GCST006572 | 0.000892833 | 230.4129593 | | 45 | rs17049085 | -0.02602 | 0.1105 | 0.9056 | PDAOO | Cognitive performance || id:ebi-a-GCST006572 | 0.000880669 | 227.2709644 | | 46 | rs17106817 | -0.01854 | 0.2840 | 0.3305 | PDAOO | Cognitive performance || id:ebi-a-GCST006572 | 0.00121052 | 312.497576 | | 47 | rs17428810 | -0.01788 | 0.2687 | 0.3521 | PDAOO | Cognitive performance || id:ebi-a-GCST006572 | 0.00124952 | 322.578143 | | 48 | rs1812587 | -0.01594 | 0.4456 | 0.8140 | PDAOO | Cognitive performance || id:ebi-a-GCST006572 | 0.001349521 | 348.4293531 | | 49 | rs1892419 | -0.0276 | 0.2330 | 0.6151 | PDAOO | Cognitive performance || id:ebi-a-GCST006572 | 0.001146128 | 295.8556933 | | 50 | rs1906252 | 0.03071 | 0.4966 | 0.4252 | PDAOO | Cognitive performance || id:ebi-a-GCST006572 | 0.001358978 | 350.8744713 | | 51 | rs2005078 | -0.01924 | 0.6667 | 0.6599 | PDAOO | Cognitive performance || id:ebi-a-GCST006572 | 0.001274151 | 328.9448169 | | 52 | rs2143103 | 0.02382 | 0.1446 | 0.4075 | PDAOO | Cognitive performance || id:ebi-a-GCST006572 | 0.000933672 | 240.9619863 | | 53 | rs2180111 | -0.01812 | 0.7058 | 0.7474 | PDAOO | Cognitive performance || id:ebi-a-GCST006572 | 0.00121052 | 312.497576 | | 54 | rs2239647 | 0.02093 | 0.5459 | 0.5118 | PDAOO | Cognitive performance || id:ebi-a-GCST006572 | 0.001340194 | 346.0180773 | | 55 | rs2295499 | -0.0191 | 0.4439 | 0.4038 | PDAOO | Cognitive performance || id:ebi-a-GCST006572 | 0.001349521 | 348.4293531 | | 56 | rs2352974 | -0.0319 | 0.5000 | 0.8756 | PDAOO | Cognitive performance || id:ebi-a-GCST006572 | 0.001358978 | 350.8744713 | | 57 | rs2426132 | 0.027 | 0.4728 | 0.8868 | PDAOO | Cognitive performance || id:ebi-a-GCST006572 | 0.001354233 | 349.6476375 | | 58 | rs2439649 | 0.01605 | 0.5391 | 0.0912 | PDAOO | Cognitive performance || id:ebi-a-GCST006572 | 0.001354233 | 349.6476375 | | 59 | rs2478281 | -0.02273 | 0.7534 | 0.7457 | PDAOO | Cognitive performance || id:ebi-a-GCST006572 | 0.001188267 | 306.7460869 | | 60 | rs26046 | -0.02044 | 0.3673 | 0.2427 | PDAOO | Cognitive performance || id:ebi-a-GCST006572 | 0.001326443 | 342.463097 | | 61 | rs2647995 | 0.01861 | 0.2925 | 0.3760 | PDAOO | Cognitive performance || id:ebi-a-GCST006572 | 0.001218124 | 314.4629696 | | 62 | rs2652454 | 0.01587 | 0.5221 | 0.9537 | PDAOO | Cognitive performance || id:ebi-a-GCST006572 | 0.001349521 | 348.4293531 | | 63 | rs2721173 | -0.01661 | 0.4490 | 0.0977 | PDAOO | Cognitive performance || id:ebi-a-GCST006572 | 0.001354233 | 349.6476375 | | 64 | rs2737339 | 0.01827 | 0.4099 | 0.7083 | PDAOO | Cognitive performance || id:ebi-a-GCST006572 | 0.001326443 | 342.463097 | | 65 | rs2799399 | -0.01572 | 0.4167 | 0.5097 | PDAOO | Cognitive performance || id:ebi-a-GCST006572 | 0.001344841 | 347.2195289 | | 66 | rs2806048 | 0.01897 | 0.3571 | 0.7225 | PDAOO | Cognitive performance || id:ebi-a-GCST006572 | 0.001312972 | 338.9804215 | | 67 | rs2836921 | 0.01632 | 0.3248 | 0.5076 | PDAOO | Cognitive performance || id:ebi-a-GCST006572 | 0.001299771 | 335.5678669 | | 68 | rs2852931 | 0.02842 | 0.8486 | 0.1366 | PDAOO | Cognitive performance || id:ebi-a-GCST006572 | 0.000880669 | 227.2709644 | | 69 | rs287883 | 0.01858 | 0.3112 | 0.2007 | PDAOO | Cognitive performance || id:ebi-a-GCST006572 | 0.001233623 | 318.4688673 | | 70 | rs297589 | 0.01715 | 0.6684 | 0.4142 | PDAOO | Cognitive performance || id:ebi-a-GCST006572 | 0.001241521 | 320.5103344 | | 71 | rs2977464 | 0.02091 | 0.1684 | 0.8340 | PDAOO | Cognitive performance || id:ebi-a-GCST006572 | 0.001061436 | 273.9704776 | | 72 | rs3113262 | -0.01963 | 0.4932 | 0.8641 | PDAOO | Cognitive performance || id:ebi-a-GCST006572 | 0.001354233 | 349.6476375 | | 73 | rs3128341 | 0.03343 | 0.8248 | 0.4305 | PDAOO | Cognitive performance || id:ebi-a-GCST006572 | 0.00109748 | 283.2839216 | | 74 | rs335426 | -0.01973 | 0.5000 | 0.7508 | PDAOO | Cognitive performance || id:ebi-a-GCST006572 | 0.001344841 | 347.2195289 | | 75 | rs34802460 | 0.01917 | 0.2568 | 0.9772 | PDAOO | Cognitive performance || id:ebi-a-GCST006572 | 0.001103726 | 284.898075 | | 76 | rs34811474 | 0.02761 | 0.2296 | 0.1213 | PDAOO | Cognitive performance || id:ebi-a-GCST006572 | 0.001119658 | 289.0150992 | | 77 | rs35526560 | -0.01958 | 0.5765 | 0.1499 | PDAOO | Cognitive performance || id:ebi-a-GCST006572 | 0.001358978 | 350.8744713 | | 78 | rs35853157 | 0.0172 | 0.3163 | 0.1361 | PDAOO | Cognitive performance || id:ebi-a-GCST006572 | 0.001274151 | 328.9448169 | | 79 | rs3735478 | 0.02279 | 0.2857 | 0.6062 | PDAOO | Cognitive performance || id:ebi-a-GCST006572 | 0.001229711 | 317.457855 | | 80 | rs3740422 | -0.02553 | 0.3486 | 0.7817 | PDAOO | Cognitive performance || id:ebi-a-GCST006572 | 0.00129543 | 334.4455663 | | 81 | rs3843954 | -0.02084 | 0.2500 | 0.6315 | PDAOO | Cognitive performance || id:ebi-a-GCST006572 | 0.001149525 | 296.7336033 | | 82 | rs3860537 | -0.01861 | 0.7585 | 0.3362 | PDAOO | Cognitive performance || id:ebi-a-GCST006572 | 0.001152943 | 297.6167391 | | 83 | rs39302 | 0.02088 | 0.8078 | 0.3188 | PDAOO | Cognitive performance || id:ebi-a-GCST006572 | 0.001088241 | 280.8966976 | | 84 | rs3943667 | -0.0185 | 0.7228 | 0.9597 | PDAOO | Cognitive performance || id:ebi-a-GCST006572 | 0.001237559 | 319.4863397 | | 85 | rs4342312 | -0.01793 | 0.3741 | 0.6213 | PDAOO | Cognitive performance || id:ebi-a-GCST006572 | 0.001317432 | 340.1334161 | | 86 | rs4347883 | 0.01595 | 0.4388 | 0.4259 | PDAOO | Cognitive performance || id:ebi-a-GCST006572 | 0.001349521 | 348.4293531 | | 87 | rs4463213 | 0.02183 | 0.5408 | 0.7135 | PDAOO | Cognitive performance || id:ebi-a-GCST006572 | 0.001349521 | 348.4293531 | | 88 | rs4470366 | 0.01815 | 0.5612 | 0.5346 | PDAOO | Cognitive performance || id:ebi-a-GCST006572 | 0.001335579 | 344.8249115 | | 89 | rs4744250 | 0.01834 | 0.3520 | 0.7908 | PDAOO | Cognitive performance || id:ebi-a-GCST006572 | 0.001291117 | 333.3307478 | | 90 | rs4937860 | -0.02588 | 0.0935 | 0.8084 | PDAOO | Cognitive performance || id:ebi-a-GCST006572 | 0.000835155 | 215.5155697 | | 91 | rs4976976 | 0.01978 | 0.3861 | 0.8345 | PDAOO | Cognitive performance || id:ebi-a-GCST006572 | 0.001326443 | 342.463097 | | 92 | rs56135595 | -0.02216 | 0.1412 | 0.5065 | PDAOO | Cognitive performance || id:ebi-a-GCST006572 | 0.000968651 | 249.9980608 | | 93 | rs56290130 | -0.01692 | 0.3231 | 0.2699 | PDAOO | Cognitive performance || id:ebi-a-GCST006572 | 0.001274151 | 328.9448169 | | 94 | rs5751191 | -0.02214 | 0.4779 | 0.0359 | PDAOO | Cognitive performance || id:ebi-a-GCST006572 | 0.001354233 | 349.6476375 | | 95 | rs5757670 | 0.02009 | 0.6582 | 0.8096 | PDAOO | Cognitive performance || id:ebi-a-GCST006572 | 0.001257624 | 324.6728063 | | 96 | rs58489175 | 0.02121 | 0.2823 | 0.9885 | PDAOO | Cognitive performance || id:ebi-a-GCST006572 | 0.001225824 | 316.4532415 | | 97 | rs602512 | 0.01948 | 0.3861 | 0.0391 | PDAOO | Cognitive performance || id:ebi-a-GCST006572 | 0.001321922 | 341.294281 | | 98 | rs61815057 | 0.01681 | 0.3963 | 0.5309 | PDAOO | Cognitive performance || id:ebi-a-GCST006572 | 0.001326443 | 342.463097 | | 99 | rs62047970 | 0.01879 | 0.4218 | 0.5693 | PDAOO | Cognitive performance || id:ebi-a-GCST006572 | 0.001330996 | 343.6399461 | | 100 | rs62065449 | -0.02626 | 0.2007 | 0.6650 | PDAOO | Cognitive performance || id:ebi-a-GCST006572 | 0.001049942 | 271.0006079 | | 101 | rs620729 | 0.01912 | 0.3146 | 0.3084 | PDAOO | Cognitive performance || id:ebi-a-GCST006572 | 0.001274151 | 328.9448169 | | 102 | rs62169190 | -0.02043 | 0.1769 | 0.9401 | PDAOO | Cognitive performance || id:ebi-a-GCST006572 | 0.001052792 | 271.7370226 | | 103 | rs6535809 | -0.01956 | 0.5000 | 0.6245 | PDAOO | Cognitive performance || id:ebi-a-GCST006572 | 0.001358978 | 350.8744713 | | 104 | rs6550835 | -0.02479 | 0.3027 | 0.0751 | PDAOO | Cognitive performance || id:ebi-a-GCST006572 | 0.001274151 | 328.9448169 | | 105 | rs6587843 | 0.01774 | 0.4711 | 0.0549 | PDAOO | Cognitive performance || id:ebi-a-GCST006572 | 0.001358978 | 350.8744713 | | 106 | rs6708515 | -0.01671 | 0.6207 | 0.1065 | PDAOO | Cognitive performance || id:ebi-a-GCST006572 | 0.001299771 | 335.5678669 | | 107 | rs6718450 | 0.01698 | 0.4252 | 0.7063 | PDAOO | Cognitive performance || id:ebi-a-GCST006572 | 0.001308542 | 337.8352173 | | 108 | rs6798941 | 0.01794 | 0.2857 | 0.9038 | PDAOO | Cognitive performance || id:ebi-a-GCST006572 | 0.001237559 | 319.4863397 | | 109 | rs6819372 | 0.01865 | 0.5357 | 0.8637 | PDAOO | Cognitive performance || id:ebi-a-GCST006572 | 0.001354233 | 349.6476375 | | 110 | rs6860626 | -0.02149 | 0.1412 | 0.7704 | PDAOO | Cognitive performance || id:ebi-a-GCST006572 | 0.001030416 | 265.9553839 | | 111 | rs6903716 | -0.01842 | 0.2738 | 0.7235 | PDAOO | Cognitive performance || id:ebi-a-GCST006572 | 0.001233623 | 318.4688673 | | 112 | rs6952104 | 0.01864 | 0.5221 | 0.8022 | PDAOO | Cognitive performance || id:ebi-a-GCST006572 | 0.001358978 | 350.8744713 | | 113 | rs6975134 | 0.02194 | 0.4218 | 0.0045 | PDAOO | Cognitive performance || id:ebi-a-GCST006572 | 0.001330996 | 343.6399461 | | 114 | rs702222 | -0.02002 | 0.3282 | 0.5594 | PDAOO | Cognitive performance || id:ebi-a-GCST006572 | 0.001304142 | 336.697725 | | 115 | rs7044246 | 0.01748 | 0.6956 | 0.4140 | PDAOO | Cognitive performance || id:ebi-a-GCST006572 | 0.001245508 | 321.5409142 | | 116 | rs7256776 | -0.01864 | 0.2908 | 0.6232 | PDAOO | Cognitive performance || id:ebi-a-GCST006572 | 0.001274151 | 328.9448169 | | 117 | rs72739469 | 0.03577 | 0.0544 | 0.3302 | PDAOO | Cognitive performance || id:ebi-a-GCST006572 | 0.00062616 | 161.5496354 | | 118 | rs72821233 | -0.0201 | 0.2721 | 0.7491 | PDAOO | Cognitive performance || id:ebi-a-GCST006572 | 0.00119929 | 309.5951218 | | 119 | rs7312770 | -0.016 | 0.5136 | 0.1524 | PDAOO | Cognitive performance || id:ebi-a-GCST006572 | 0.001349521 | 348.4293531 | | 120 | rs73189617 | 0.01854 | 0.3299 | 0.6603 | PDAOO | Cognitive performance || id:ebi-a-GCST006572 | 0.001229711 | 317.457855 | | 121 | rs73845427 | -0.0466 | 0.0289 | 0.0361 | PDAOO | Cognitive performance || id:ebi-a-GCST006572 | 0.000480359 | 123.9147761 | | 122 | rs73989053 | -0.02211 | 0.1616 | 0.3415 | PDAOO | Cognitive performance || id:ebi-a-GCST006572 | 0.001016907 | 262.4651557 | | 123 | rs74370218 | -0.018 | 0.3827 | 0.3764 | PDAOO | Cognitive performance || id:ebi-a-GCST006572 | 0.001312972 | 338.9804215 | | 124 | rs7573001 | -0.01647 | 0.4150 | 0.1096 | PDAOO | Cognitive performance || id:ebi-a-GCST006572 | 0.001312972 | 338.9804215 | | 125 | rs7588384 | -0.01891 | 0.3129 | 0.5180 | PDAOO | Cognitive performance || id:ebi-a-GCST006572 | 0.00120301 | 310.5565973 | | 126 | rs75973558 | -0.02463 | 0.1190 | 0.5341 | PDAOO | Cognitive performance || id:ebi-a-GCST006572 | 0.000909585 | 234.7399632 | | 127 | rs7599860 | 0.01967 | 0.2279 | 0.2710 | PDAOO | Cognitive performance || id:ebi-a-GCST006572 | 0.001159839 | 299.3988752 | | 128 | rs7626560 | 0.02393 | 0.1361 | 0.2032 | PDAOO | Cognitive performance || id:ebi-a-GCST006572 | 0.000996014 | 257.0674147 | | 129 | rs77128898 | -0.04626 | 0.0221 | 0.5052 | PDAOO | Cognitive performance || id:ebi-a-GCST006572 | 0.00050474 | 130.2073233 | | 130 | rs78358737 | 0.04248 | 0.0340 | 0.8892 | PDAOO | Cognitive performance || id:ebi-a-GCST006572 | 0.000564217 | 145.5592785 | | 131 | rs78382112 | 0.03682 | 0.0578 | 0.3032 | PDAOO | Cognitive performance || id:ebi-a-GCST006572 | 0.000606574 | 156.4933088 | | 132 | rs7963801 | -0.02296 | 0.5476 | 0.6650 | PDAOO | Cognitive performance || id:ebi-a-GCST006572 | 0.001326443 | 342.463097 | | 133 | rs80170948 | -0.04589 | 0.0527 | 0.9566 | PDAOO | Cognitive performance || id:ebi-a-GCST006572 | 0.000549041 | 141.6419608 | | 134 | rs8054299 | 0.02556 | 0.3316 | 0.9289 | PDAOO | Cognitive performance || id:ebi-a-GCST006572 | 0.001265833 | 326.7948507 | | 135 | rs8058881 | 0.01831 | 0.2500 | 0.5973 | PDAOO | Cognitive performance || id:ebi-a-GCST006572 | 0.001159839 | 299.3988752 | | 136 | rs830383 | -0.01952 | 0.6310 | 0.7741 | PDAOO | Cognitive performance || id:ebi-a-GCST006572 | 0.001312972 | 338.9804215 | | 137 | rs875361 | -0.01623 | 0.4490 | 0.6733 | PDAOO | Cognitive performance || id:ebi-a-GCST006572 | 0.001349521 | 348.4293531 | | 138 | rs889169 | 0.01743 | 0.5867 | 0.1566 | PDAOO | Cognitive performance || id:ebi-a-GCST006572 | 0.001291117 | 333.3307478 | | 139 | rs9384679 | -0.02822 | 0.4082 | 0.8912 | PDAOO | Cognitive performance || id:ebi-a-GCST006572 | 0.001312972 | 338.9804215 | | 140 | rs9436866 | 0.03309 | 0.0952 | 0.5357 | PDAOO | Cognitive performance || id:ebi-a-GCST006572 | 0.000805662 | 207.8985953 | | 141 | rs991871 | 0.0243 | 0.7551 | 0.2595 | PDAOO | Cognitive performance || id:ebi-a-GCST006572 | 0.001149525 | 296.7336033 | | 142 | rs9930063 | 0.01633 | 0.4592 | 0.9023 | PDAOO | Cognitive performance || id:ebi-a-GCST006572 | 0.001344841 | 347.2195289 | | 1 | rs10129426 | 0.01926 | 0.5306 | 0.9221 | PD || id:ieu-b-7 | Cognitive performance || id:ebi-a-GCST006572 | 0.001349521 | 348.4293531 | | 2 | rs10189857 | -0.02289 | 0.4184 | 0.0321 | PD || id:ieu-b-7 | Cognitive performance || id:ebi-a-GCST006572 | 0.001344841 | 347.2195289 | | 3 | rs10191758 | 0.01965 | 0.3810 | 0.6688 | PD || id:ieu-b-7 | Cognitive performance || id:ebi-a-GCST006572 | 0.001326443 | 342.463097 | | 4 | rs1035738 | 0.05092 | 0.0272 | 0.7732 | PD || id:ieu-b-7 | Cognitive performance || id:ebi-a-GCST006572 | 0.000504084 | 130.038003 | | 5 | rs10497818 | -0.01715 | 0.4303 | 0.1749 | PD || id:ieu-b-7 | Cognitive performance || id:ebi-a-GCST006572 | 0.001344841 | 347.2195289 | | 6 | rs1064608 | -0.01884 | 0.3639 | 0.2470 | PD || id:ieu-b-7 | Cognitive performance || id:ebi-a-GCST006572 | 0.00129543 | 334.4455663 | | 7 | rs1064608 | -0.01884 | 0.3639 | 0.2470 | PD || id:ieu-b-7 | Cognitive performance || id:ebi-a-GCST006572 | 0.00129543 | 334.4455663 | | 8 | rs1064608 | -0.01884 | 0.3639 | 0.2470 | PD || id:ieu-b-7 | Cognitive performance || id:ebi-a-GCST006572 | 0.00129543 | 334.4455663 | | 9 | rs10865397 | 0.01675 | 0.5221 | 0.7389 | PD || id:ieu-b-7 | Cognitive performance || id:ebi-a-GCST006572 | 0.001340194 | 346.0180773 | | 10 | rs10874938 | -0.02364 | 0.5068 | 0.0527 | PD || id:ieu-b-7 | Cognitive performance || id:ebi-a-GCST006572 | 0.001358978 | 350.8744713 | | 11 | rs10875914 | 0.02373 | 0.3554 | 0.0410 | PD || id:ieu-b-7 | Cognitive performance || id:ebi-a-GCST006572 | 0.001330996 | 343.6399461 | | 12 | rs10990610 | 0.02498 | 0.1650 | 0.0577 | PD || id:ieu-b-7 | Cognitive performance || id:ebi-a-GCST006572 | 0.001006352 | 259.738245 | | 13 | rs11079849 | 0.01996 | 0.3078 | 0.6129 | PD || id:ieu-b-7 | Cognitive performance || id:ebi-a-GCST006572 | 0.001269978 | 327.8663093 | | 14 | rs11123820 | 0.0243 | 0.4065 | 0.0258 | PD || id:ieu-b-7 | Cognitive performance || id:ebi-a-GCST006572 | 0.001326443 | 342.463097 | | 15 | rs11138947 | 0.01737 | 0.7330 | 0.4602 | PD || id:ieu-b-7 | Cognitive performance || id:ebi-a-GCST006572 | 0.001218124 | 314.4629696 | | 16 | rs11210871 | 0.01917 | 0.6701 | 0.6933 | PD || id:ieu-b-7 | Cognitive performance || id:ebi-a-GCST006572 | 0.001241521 | 320.5103344 | | 17 | rs11259916 | 0.01744 | 0.7330 | 0.7676 | PD || id:ieu-b-7 | Cognitive performance || id:ebi-a-GCST006572 | 0.001225824 | 316.4532415 | | 18 | rs112780312 | -0.02121 | 0.2534 | 0.7092 | PD || id:ieu-b-7 | Cognitive performance || id:ebi-a-GCST006572 | 0.001206754 | 311.5240633 | | 19 | rs1144593 | 0.02276 | 0.2993 | 0.8011 | PD || id:ieu-b-7 | Cognitive performance || id:ebi-a-GCST006572 | 0.001253559 | 323.6220852 | | 20 | rs11662271 | -0.0232 | 0.5153 | 0.3555 | PD || id:ieu-b-7 | Cognitive performance || id:ebi-a-GCST006572 | 0.001358978 | 350.8744713 | | 21 | rs11693702 | -0.02045 | 0.4490 | 0.0083 | PD || id:ieu-b-7 | Cognitive performance || id:ebi-a-GCST006572 | 0.001354233 | 349.6476375 | | 22 | rs11720523 | 0.01622 | 0.4456 | 0.3680 | PD || id:ieu-b-7 | Cognitive performance || id:ebi-a-GCST006572 | 0.001344841 | 347.2195289 | | 23 | rs11793831 | 0.02693 | 0.4354 | 0.4824 | PD || id:ieu-b-7 | Cognitive performance || id:ebi-a-GCST006572 | 0.001335579 | 344.8249115 | | 24 | rs12435486 | -0.01823 | 0.2483 | 0.7952 | PD || id:ieu-b-7 | Cognitive performance || id:ebi-a-GCST006572 | 0.001170338 | 302.1124602 | | 25 | rs12439619 | 0.01994 | 0.3129 | 0.1376 | PD || id:ieu-b-7 | Cognitive performance || id:ebi-a-GCST006572 | 0.001261715 | 325.7303724 | | 26 | rs12441495 | -0.02575 | 0.1361 | 0.4379 | PD || id:ieu-b-7 | Cognitive performance || id:ebi-a-GCST006572 | 0.000913871 | 235.8472272 | | 27 | rs12535854 | 0.01783 | 0.6667 | 0.1383 | PD || id:ieu-b-7 | Cognitive performance || id:ebi-a-GCST006572 | 0.001274151 | 328.9448169 | | 28 | rs12536800 | 0.01939 | 0.2806 | 0.0868 | PD || id:ieu-b-7 | Cognitive performance || id:ebi-a-GCST006572 | 0.001206754 | 311.5240633 | | 29 | rs12635303 | 0.01849 | 0.2925 | 0.8259 | PD || id:ieu-b-7 | Cognitive performance || id:ebi-a-GCST006572 | 0.001253559 | 323.6220852 | | 30 | rs12773747 | 0.0205 | 0.2262 | 0.1462 | PD || id:ieu-b-7 | Cognitive performance || id:ebi-a-GCST006572 | 0.001166817 | 301.2024829 | | 31 | rs13107325 | -0.05434 | 0.0901 | 0.3311 | PD || id:ieu-b-7 | Cognitive performance || id:ebi-a-GCST006572 | 0.000715053 | 184.5004139 | | 32 | rs13120565 | 0.01825 | 0.6429 | 0.5537 | PD || id:ieu-b-7 | Cognitive performance || id:ebi-a-GCST006572 | 0.001317432 | 340.1334161 | | 33 | rs13163336 | 0.0317 | 0.1735 | 0.9415 | PD || id:ieu-b-7 | Cognitive performance || id:ebi-a-GCST006572 | 0.000990925 | 255.7524919 | | 34 | rs13253386 | 0.01836 | 0.5051 | 0.7910 | PD || id:ieu-b-7 | Cognitive performance || id:ebi-a-GCST006572 | 0.001358978 | 350.8744713 | | 35 | rs136554 | 0.01586 | 0.4592 | 0.8284 | PD || id:ieu-b-7 | Cognitive performance || id:ebi-a-GCST006572 | 0.001344841 | 347.2195289 | | 36 | rs1391438 | -0.01691 | 0.6854 | 0.7452 | PD || id:ieu-b-7 | Cognitive performance || id:ebi-a-GCST006572 | 0.001265833 | 326.7948507 | | 37 | rs1408579 | 0.0168 | 0.5000 | 0.8230 | PD || id:ieu-b-7 | Cognitive performance || id:ebi-a-GCST006572 | 0.001354233 | 349.6476375 | | 38 | rs1479073 | -0.01731 | 0.6888 | 0.1132 | PD || id:ieu-b-7 | Cognitive performance || id:ebi-a-GCST006572 | 0.001257624 | 324.6728063 | | 39 | rs148696809 | 0.0406 | 0.0816 | 0.2254 | PD || id:ieu-b-7 | Cognitive performance || id:ebi-a-GCST006572 | 0.000870782 | 224.717358 | | 40 | rs1507010 | 0.01728 | 0.4949 | 0.6095 | PD || id:ieu-b-7 | Cognitive performance || id:ebi-a-GCST006572 | 0.001354233 | 349.6476375 | | 41 | rs1523048 | -0.01776 | 0.6003 | 0.8890 | PD || id:ieu-b-7 | Cognitive performance || id:ebi-a-GCST006572 | 0.001308542 | 337.8352173 | | 42 | rs1567154 | -0.02045 | 0.2653 | 0.2917 | PD || id:ieu-b-7 | Cognitive performance || id:ebi-a-GCST006572 | 0.001110044 | 286.5307287 | | 43 | rs159428 | -0.01676 | 0.5068 | 0.4960 | PD || id:ieu-b-7 | Cognitive performance || id:ebi-a-GCST006572 | 0.001340194 | 346.0180773 | | 44 | rs17002025 | 0.02886 | 0.1378 | 0.9741 | PD || id:ieu-b-7 | Cognitive performance || id:ebi-a-GCST006572 | 0.000892833 | 230.4129593 | | 45 | rs17049085 | -0.02602 | 0.1105 | 0.5782 | PD || id:ieu-b-7 | Cognitive performance || id:ebi-a-GCST006572 | 0.000880669 | 227.2709644 | | 46 | rs17106817 | -0.01854 | 0.2840 | 0.8185 | PD || id:ieu-b-7 | Cognitive performance || id:ebi-a-GCST006572 | 0.00121052 | 312.497576 | | 47 | rs17428810 | -0.01788 | 0.2687 | 0.0403 | PD || id:ieu-b-7 | Cognitive performance || id:ebi-a-GCST006572 | 0.00124952 | 322.578143 | | 48 | rs1812587 | -0.01594 | 0.4456 | 0.3738 | PD || id:ieu-b-7 | Cognitive performance || id:ebi-a-GCST006572 | 0.001349521 | 348.4293531 | | 49 | rs1892419 | -0.0276 | 0.2330 | 0.8919 | PD || id:ieu-b-7 | Cognitive performance || id:ebi-a-GCST006572 | 0.001146128 | 295.8556933 | | 50 | rs1906252 | 0.03071 | 0.4966 | 0.0175 | PD || id:ieu-b-7 | Cognitive performance || id:ebi-a-GCST006572 | 0.001358978 | 350.8744713 | | 51 | rs2005078 | -0.01924 | 0.6667 | 0.2369 | PD || id:ieu-b-7 | Cognitive performance || id:ebi-a-GCST006572 | 0.001274151 | 328.9448169 | | 52 | rs2143103 | 0.02382 | 0.1446 | 0.0005 | PD || id:ieu-b-7 | Cognitive performance || id:ebi-a-GCST006572 | 0.000933672 | 240.9619863 | | 53 | rs2180111 | -0.01812 | 0.7058 | 0.3095 | PD || id:ieu-b-7 | Cognitive performance || id:ebi-a-GCST006572 | 0.00121052 | 312.497576 | | 54 | rs2239647 | 0.02093 | 0.5459 | 0.8608 | PD || id:ieu-b-7 | Cognitive performance || id:ebi-a-GCST006572 | 0.001340194 | 346.0180773 | | 55 | rs2295499 | -0.0191 | 0.4439 | 0.1494 | PD || id:ieu-b-7 | Cognitive performance || id:ebi-a-GCST006572 | 0.001349521 | 348.4293531 | | 56 | rs2352974 | -0.0319 | 0.5000 | 0.7076 | PD || id:ieu-b-7 | Cognitive performance || id:ebi-a-GCST006572 | 0.001358978 | 350.8744713 | | 57 | rs2426132 | 0.027 | 0.4728 | 0.0158 | PD || id:ieu-b-7 | Cognitive performance || id:ebi-a-GCST006572 | 0.001354233 | 349.6476375 | | 58 | rs2439649 | 0.01605 | 0.5391 | 0.0037 | PD || id:ieu-b-7 | Cognitive performance || id:ebi-a-GCST006572 | 0.001354233 | 349.6476375 | | 59 | rs2478281 | -0.02273 | 0.7534 | 0.5815 | PD || id:ieu-b-7 | Cognitive performance || id:ebi-a-GCST006572 | 0.001188267 | 306.7460869 | | 60 | rs26046 | -0.02044 | 0.3673 | 0.5129 | PD || id:ieu-b-7 | Cognitive performance || id:ebi-a-GCST006572 | 0.001326443 | 342.463097 | | 61 | rs2647995 | 0.01861 | 0.2925 | 0.3613 | PD || id:ieu-b-7 | Cognitive performance || id:ebi-a-GCST006572 | 0.001218124 | 314.4629696 | | 62 | rs2652454 | 0.01587 | 0.5221 | 0.3670 | PD || id:ieu-b-7 | Cognitive performance || id:ebi-a-GCST006572 | 0.001349521 | 348.4293531 | | 63 | rs2721173 | -0.01661 | 0.4490 | 0.0493 | PD || id:ieu-b-7 | Cognitive performance || id:ebi-a-GCST006572 | 0.001354233 | 349.6476375 | | 64 | rs2737339 | 0.01827 | 0.4099 | 0.5621 | PD || id:ieu-b-7 | Cognitive performance || id:ebi-a-GCST006572 | 0.001326443 | 342.463097 | | 65 | rs276626 | -0.02075 | 0.1616 | 0.4648 | PD || id:ieu-b-7 | Cognitive performance || id:ebi-a-GCST006572 | 0.00101958 | 263.1558535 | | 66 | rs2799399 | -0.01572 | 0.4167 | 0.8402 | PD || id:ieu-b-7 | Cognitive performance || id:ebi-a-GCST006572 | 0.001344841 | 347.2195289 | | 67 | rs2806048 | 0.01897 | 0.3571 | 0.9758 | PD || id:ieu-b-7 | Cognitive performance || id:ebi-a-GCST006572 | 0.001312972 | 338.9804215 | | 68 | rs2836921 | 0.01632 | 0.3248 | 0.5007 | PD || id:ieu-b-7 | Cognitive performance || id:ebi-a-GCST006572 | 0.001299771 | 335.5678669 | | 69 | rs2852931 | 0.02842 | 0.8486 | 0.8802 | PD || id:ieu-b-7 | Cognitive performance || id:ebi-a-GCST006572 | 0.000880669 | 227.2709644 | | 70 | rs297589 | 0.01715 | 0.6684 | 0.3072 | PD || id:ieu-b-7 | Cognitive performance || id:ebi-a-GCST006572 | 0.001241521 | 320.5103344 | | 71 | rs297589 | 0.01715 | 0.6684 | 0.3072 | PD || id:ieu-b-7 | Cognitive performance || id:ebi-a-GCST006572 | 0.001241521 | 320.5103344 | | 72 | rs2977464 | 0.02091 | 0.1684 | 0.1163 | PD || id:ieu-b-7 | Cognitive performance || id:ebi-a-GCST006572 | 0.001061436 | 273.9704776 | | 73 | rs3113262 | -0.01963 | 0.4932 | 0.1166 | PD || id:ieu-b-7 | Cognitive performance || id:ebi-a-GCST006572 | 0.001354233 | 349.6476375 | | 74 | rs3128341 | 0.03343 | 0.8248 | 0.6658 | PD || id:ieu-b-7 | Cognitive performance || id:ebi-a-GCST006572 | 0.00109748 | 283.2839216 | | 75 | rs335426 | -0.01973 | 0.5000 | 0.1606 | PD || id:ieu-b-7 | Cognitive performance || id:ebi-a-GCST006572 | 0.001344841 | 347.2195289 | | 76 | rs34802460 | 0.01917 | 0.2568 | 0.0396 | PD || id:ieu-b-7 | Cognitive performance || id:ebi-a-GCST006572 | 0.001103726 | 284.898075 | | 77 | rs34811474 | 0.02761 | 0.2296 | 0.0098 | PD || id:ieu-b-7 | Cognitive performance || id:ebi-a-GCST006572 | 0.001119658 | 289.0150992 | | 78 | rs35526560 | -0.01958 | 0.5765 | 0.6919 | PD || id:ieu-b-7 | Cognitive performance || id:ebi-a-GCST006572 | 0.001358978 | 350.8744713 | | 79 | rs35853157 | 0.0172 | 0.3163 | 0.1779 | PD || id:ieu-b-7 | Cognitive performance || id:ebi-a-GCST006572 | 0.001274151 | 328.9448169 | | 80 | rs3735478 | 0.02279 | 0.2857 | 0.5409 | PD || id:ieu-b-7 | Cognitive performance || id:ebi-a-GCST006572 | 0.001229711 | 317.457855 | | 81 | rs3843954 | -0.02084 | 0.2500 | 0.4869 | PD || id:ieu-b-7 | Cognitive performance || id:ebi-a-GCST006572 | 0.001149525 | 296.7336033 | | 82 | rs3860537 | -0.01861 | 0.7585 | 0.6862 | PD || id:ieu-b-7 | Cognitive performance || id:ebi-a-GCST006572 | 0.001152943 | 297.6167391 | | 83 | rs3943667 | -0.0185 | 0.7228 | 0.8717 | PD || id:ieu-b-7 | Cognitive performance || id:ebi-a-GCST006572 | 0.001237559 | 319.4863397 | | 84 | rs4342312 | -0.01793 | 0.3741 | 0.0645 | PD || id:ieu-b-7 | Cognitive performance || id:ebi-a-GCST006572 | 0.001317432 | 340.1334161 | | 85 | rs4347883 | 0.01595 | 0.4388 | 0.5591 | PD || id:ieu-b-7 | Cognitive performance || id:ebi-a-GCST006572 | 0.001349521 | 348.4293531 | | 86 | rs4463213 | 0.02183 | 0.5408 | 0.0262 | PD || id:ieu-b-7 | Cognitive performance || id:ebi-a-GCST006572 | 0.001349521 | 348.4293531 | | 87 | rs4744250 | 0.01834 | 0.3520 | 0.0378 | PD || id:ieu-b-7 | Cognitive performance || id:ebi-a-GCST006572 | 0.001291117 | 333.3307478 | | 88 | rs4937860 | -0.02588 | 0.0935 | 0.0152 | PD || id:ieu-b-7 | Cognitive performance || id:ebi-a-GCST006572 | 0.000835155 | 215.5155697 | | 89 | rs4976976 | 0.01978 | 0.3861 | 0.4293 | PD || id:ieu-b-7 | Cognitive performance || id:ebi-a-GCST006572 | 0.001326443 | 342.463097 | | 90 | rs56135595 | -0.02216 | 0.1412 | 0.6185 | PD || id:ieu-b-7 | Cognitive performance || id:ebi-a-GCST006572 | 0.000968651 | 249.9980608 | | 91 | rs56290130 | -0.01692 | 0.3231 | 0.1256 | PD || id:ieu-b-7 | Cognitive performance || id:ebi-a-GCST006572 | 0.001274151 | 328.9448169 | | 92 | rs5751191 | -0.02214 | 0.4779 | 0.7902 | PD || id:ieu-b-7 | Cognitive performance || id:ebi-a-GCST006572 | 0.001354233 | 349.6476375 | | 93 | rs5757670 | 0.02009 | 0.6582 | 0.3482 | PD || id:ieu-b-7 | Cognitive performance || id:ebi-a-GCST006572 | 0.001257624 | 324.6728063 | | 94 | rs58489175 | 0.02121 | 0.2823 | 0.8446 | PD || id:ieu-b-7 | Cognitive performance || id:ebi-a-GCST006572 | 0.001225824 | 316.4532415 | | 95 | rs61815057 | 0.01681 | 0.3963 | 0.2941 | PD || id:ieu-b-7 | Cognitive performance || id:ebi-a-GCST006572 | 0.001326443 | 342.463097 | | 96 | rs62047970 | 0.01879 | 0.4218 | 0.1225 | PD || id:ieu-b-7 | Cognitive performance || id:ebi-a-GCST006572 | 0.001330996 | 343.6399461 | | 97 | rs62065449 | -0.02626 | 0.2007 | 0.0000 | PD || id:ieu-b-7 | Cognitive performance || id:ebi-a-GCST006572 | 0.001049942 | 271.0006079 | | 98 | rs620729 | 0.01912 | 0.3146 | 0.0249 | PD || id:ieu-b-7 | Cognitive performance || id:ebi-a-GCST006572 | 0.001274151 | 328.9448169 | | 99 | rs62169190 | -0.02043 | 0.1769 | 0.5612 | PD || id:ieu-b-7 | Cognitive performance || id:ebi-a-GCST006572 | 0.001052792 | 271.7370226 | | 100 | rs6535809 | -0.01956 | 0.5000 | 0.9160 | PD || id:ieu-b-7 | Cognitive performance || id:ebi-a-GCST006572 | 0.001358978 | 350.8744713 | | 101 | rs6550835 | -0.02479 | 0.3027 | 0.6564 | PD || id:ieu-b-7 | Cognitive performance || id:ebi-a-GCST006572 | 0.001274151 | 328.9448169 | | 102 | rs6587843 | 0.01774 | 0.4711 | 0.1118 | PD || id:ieu-b-7 | Cognitive performance || id:ebi-a-GCST006572 | 0.001358978 | 350.8744713 | | 103 | rs66752974 | -0.02214 | 0.1582 | 0.5330 | PD || id:ieu-b-7 | Cognitive performance || id:ebi-a-GCST006572 | 0.00100897 | 260.4146467 | | 104 | rs6708515 | -0.01671 | 0.6207 | 0.7345 | PD || id:ieu-b-7 | Cognitive performance || id:ebi-a-GCST006572 | 0.001299771 | 335.5678669 | | 105 | rs6718450 | 0.01698 | 0.4252 | 0.0296 | PD || id:ieu-b-7 | Cognitive performance || id:ebi-a-GCST006572 | 0.001308542 | 337.8352173 | | 106 | rs6798941 | 0.01794 | 0.2857 | 0.6764 | PD || id:ieu-b-7 | Cognitive performance || id:ebi-a-GCST006572 | 0.001237559 | 319.4863397 | | 107 | rs6819372 | 0.01865 | 0.5357 | 0.5261 | PD || id:ieu-b-7 | Cognitive performance || id:ebi-a-GCST006572 | 0.001354233 | 349.6476375 | | 108 | rs6860626 | -0.02149 | 0.1412 | 0.7514 | PD || id:ieu-b-7 | Cognitive performance || id:ebi-a-GCST006572 | 0.001030416 | 265.9553839 | | 109 | rs6903716 | -0.01842 | 0.2738 | 0.9128 | PD || id:ieu-b-7 | Cognitive performance || id:ebi-a-GCST006572 | 0.001233623 | 318.4688673 | | 110 | rs6952104 | 0.01864 | 0.5221 | 0.9631 | PD || id:ieu-b-7 | Cognitive performance || id:ebi-a-GCST006572 | 0.001358978 | 350.8744713 | | 111 | rs6975134 | 0.02194 | 0.4218 | 0.1040 | PD || id:ieu-b-7 | Cognitive performance || id:ebi-a-GCST006572 | 0.001330996 | 343.6399461 | | 112 | rs702222 | -0.02002 | 0.3282 | 0.8869 | PD || id:ieu-b-7 | Cognitive performance || id:ebi-a-GCST006572 | 0.001304142 | 336.697725 | | 113 | rs7044246 | 0.01748 | 0.6956 | 0.1880 | PD || id:ieu-b-7 | Cognitive performance || id:ebi-a-GCST006572 | 0.001245508 | 321.5409142 | | 114 | rs7256776 | -0.01864 | 0.2908 | 0.3953 | PD || id:ieu-b-7 | Cognitive performance || id:ebi-a-GCST006572 | 0.001274151 | 328.9448169 | | 115 | rs72739469 | 0.03577 | 0.0544 | 0.0750 | PD || id:ieu-b-7 | Cognitive performance || id:ebi-a-GCST006572 | 0.00062616 | 161.5496354 | | 116 | rs72821233 | -0.0201 | 0.2721 | 0.8654 | PD || id:ieu-b-7 | Cognitive performance || id:ebi-a-GCST006572 | 0.00119929 | 309.5951218 | | 117 | rs7312770 | -0.016 | 0.5136 | 0.6499 | PD || id:ieu-b-7 | Cognitive performance || id:ebi-a-GCST006572 | 0.001349521 | 348.4293531 | | 118 | rs73189617 | 0.01854 | 0.3299 | 0.0157 | PD || id:ieu-b-7 | Cognitive performance || id:ebi-a-GCST006572 | 0.001229711 | 317.457855 | | 119 | rs73845427 | -0.0466 | 0.0289 | 0.7319 | PD || id:ieu-b-7 | Cognitive performance || id:ebi-a-GCST006572 | 0.000480359 | 123.9147761 | | 120 | rs73989053 | -0.02211 | 0.1616 | 0.6395 | PD || id:ieu-b-7 | Cognitive performance || id:ebi-a-GCST006572 | 0.001016907 | 262.4651557 | | 121 | rs74370218 | -0.018 | 0.3827 | 0.0941 | PD || id:ieu-b-7 | Cognitive performance || id:ebi-a-GCST006572 | 0.001312972 | 338.9804215 | | 122 | rs7573001 | -0.01647 | 0.4150 | 0.5234 | PD || id:ieu-b-7 | Cognitive performance || id:ebi-a-GCST006572 | 0.001312972 | 338.9804215 | | 123 | rs75973558 | -0.02463 | 0.1190 | 0.6983 | PD || id:ieu-b-7 | Cognitive performance || id:ebi-a-GCST006572 | 0.000909585 | 234.7399632 | | 124 | rs7599860 | 0.01967 | 0.2279 | 0.5847 | PD || id:ieu-b-7 | Cognitive performance || id:ebi-a-GCST006572 | 0.001159839 | 299.3988752 | | 125 | rs7599860 | 0.01967 | 0.2279 | 0.5847 | PD || id:ieu-b-7 | Cognitive performance || id:ebi-a-GCST006572 | 0.001159839 | 299.3988752 | | 126 | rs7626560 | 0.02393 | 0.1361 | 0.1055 | PD || id:ieu-b-7 | Cognitive performance || id:ebi-a-GCST006572 | 0.000996014 | 257.0674147 | | 127 | rs77128898 | -0.04626 | 0.0221 | 0.6682 | PD || id:ieu-b-7 | Cognitive performance || id:ebi-a-GCST006572 | 0.00050474 | 130.2073233 | | 128 | rs78358737 | 0.04248 | 0.0340 | 0.4849 | PD || id:ieu-b-7 | Cognitive performance || id:ebi-a-GCST006572 | 0.000564217 | 145.5592785 | | 129 | rs78382112 | 0.03682 | 0.0578 | 0.8722 | PD || id:ieu-b-7 | Cognitive performance || id:ebi-a-GCST006572 | 0.000606574 | 156.4933088 | | 130 | rs7963801 | -0.02296 | 0.5476 | 0.5202 | PD || id:ieu-b-7 | Cognitive performance || id:ebi-a-GCST006572 | 0.001326443 | 342.463097 | | 131 | rs8054299 | 0.02556 | 0.3316 | 0.6706 | PD || id:ieu-b-7 | Cognitive performance || id:ebi-a-GCST006572 | 0.001265833 | 326.7948507 | | 132 | rs8058881 | 0.01831 | 0.2500 | 0.6053 | PD || id:ieu-b-7 | Cognitive performance || id:ebi-a-GCST006572 | 0.001159839 | 299.3988752 | | 133 | rs830383 | -0.01952 | 0.6310 | 0.3235 | PD || id:ieu-b-7 | Cognitive performance || id:ebi-a-GCST006572 | 0.001312972 | 338.9804215 | | 134 | rs875361 | -0.01623 | 0.4490 | 0.7816 | PD || id:ieu-b-7 | Cognitive performance || id:ebi-a-GCST006572 | 0.001349521 | 348.4293531 | | 135 | rs889169 | 0.01743 | 0.5867 | 0.2107 | PD || id:ieu-b-7 | Cognitive performance || id:ebi-a-GCST006572 | 0.001291117 | 333.3307478 | | 136 | rs9384679 | -0.02822 | 0.4082 | 0.7069 | PD || id:ieu-b-7 | Cognitive performance || id:ebi-a-GCST006572 | 0.001312972 | 338.9804215 | | 137 | rs9436866 | 0.03309 | 0.0952 | 0.6235 | PD || id:ieu-b-7 | Cognitive performance || id:ebi-a-GCST006572 | 0.000805662 | 207.8985953 | | 138 | rs991871 | 0.0243 | 0.7551 | 0.2777 | PD || id:ieu-b-7 | Cognitive performance || id:ebi-a-GCST006572 | 0.001149525 | 296.7336033 | | 139 | rs9930063 | 0.01633 | 0.4592 | 0.6056 | PD || id:ieu-b-7 | Cognitive performance || id:ebi-a-GCST006572 | 0.001344841 | 347.2195289 | | 1 | rs10073890 | -0.01262 | 0.7364 | 0.5166 | FEMALEPD | Years of schooling || id:ieu-a-1239 | 0.00066532 | 510.2027501 | | 2 | rs1008078 | -0.01738 | 0.4099 | 0.3748 | FEMALEPD | Years of schooling || id:ieu-a-1239 | 0.000753706 | 578.0331735 | | 3 | rs10189857 | -0.01725 | 0.4184 | 0.0167 | FEMALEPD | Years of schooling || id:ieu-a-1239 | 0.000762515 | 584.7937954 | | 4 | rs10191758 | 0.01631 | 0.3810 | 0.6958 | FEMALEPD | Years of schooling || id:ieu-a-1239 | 0.000745099 | 571.4270801 | | 5 | rs10205801 | -0.01053 | 0.5068 | 0.5278 | FEMALEPD | Years of schooling || id:ieu-a-1239 | 0.000762515 | 584.7937954 | | 6 | rs10215082 | 0.01303 | 0.5612 | 0.4987 | FEMALEPD | Years of schooling || id:ieu-a-1239 | 0.000758085 | 581.3938315 | | 7 | rs10240905 | 0.01167 | 0.6684 | 0.6418 | FEMALEPD | Years of schooling || id:ieu-a-1239 | 0.000736686 | 564.970277 | | 8 | rs10456918 | 0.01485 | 0.1820 | 0.4223 | FEMALEPD | Years of schooling || id:ieu-a-1239 | 0.000582203 | 446.4274063 | | 9 | rs10460095 | -0.01066 | 0.5867 | 0.2771 | FEMALEPD | Years of schooling || id:ieu-a-1239 | 0.000762515 | 584.7937954 | | 10 | rs1051474 | 0.01301 | 0.2738 | 0.2103 | FEMALEPD | Years of schooling || id:ieu-a-1239 | 0.000693612 | 531.9135054 | | 11 | rs10760023 | 0.01095 | 0.3316 | 0.9893 | FEMALEPD | Years of schooling || id:ieu-a-1239 | 0.000712549 | 546.4466613 | | 12 | rs10765775 | 0.01488 | 0.3963 | 0.0264 | FEMALEPD | Years of schooling || id:ieu-a-1239 | 0.000740868 | 568.1803353 | | 13 | rs10772644 | 0.01614 | 0.8929 | 0.1154 | FEMALEPD | Years of schooling || id:ieu-a-1239 | 0.000488486 | 374.5308578 | | 14 | rs10773002 | -0.02191 | 0.7211 | 0.4232 | FEMALEPD | Years of schooling || id:ieu-a-1239 | 0.000661945 | 507.6128884 | | 15 | rs10797055 | 0.00986 | 0.5102 | 0.1328 | FEMALEPD | Years of schooling || id:ieu-a-1239 | 0.000758085 | 581.3938315 | | 16 | rs10798418 | -0.00957 | 0.4660 | 0.9499 | FEMALEPD | Years of schooling || id:ieu-a-1239 | 0.000753706 | 578.0331735 | | 17 | rs10856785 | -0.01132 | 0.7296 | 0.3935 | FEMALEPD | Years of schooling || id:ieu-a-1239 | 0.000679171 | 520.8319741 | | 18 | rs10862376 | 0.01616 | 0.1361 | 0.0987 | FEMALEPD | Years of schooling || id:ieu-a-1239 | 0.000545683 | 418.4089499 | | 19 | rs10875121 | 0.01834 | 0.8571 | 0.0479 | FEMALEPD | Years of schooling || id:ieu-a-1239 | 0.000577054 | 442.4767213 | | 20 | rs10887801 | 0.01087 | 0.4371 | 0.2978 | FEMALEPD | Years of schooling || id:ieu-a-1239 | 0.000762515 | 584.7937954 | | 21 | rs10940921 | -0.01089 | 0.5697 | 0.9234 | FEMALEPD | Years of schooling || id:ieu-a-1239 | 0.000736686 | 564.970277 | | 22 | rs10963297 | 0.01904 | 0.2517 | 0.0991 | FEMALEPD | Years of schooling || id:ieu-a-1239 | 0.000658604 | 505.049187 | | 23 | rs10994777 | 0.0146 | 0.1395 | 0.9923 | FEMALEPD | Years of schooling || id:ieu-a-1239 | 0.000562139 | 431.0333578 | | 24 | rs11023749 | 0.01132 | 0.6701 | 0.1777 | FEMALEPD | Years of schooling || id:ieu-a-1239 | 0.000724417 | 555.5541057 | | 25 | rs1105307 | -0.01173 | 0.2449 | 0.2643 | FEMALEPD | Years of schooling || id:ieu-a-1239 | 0.00066873 | 512.8191745 | | 26 | rs1106090 | 0.01173 | 0.6259 | 0.0169 | FEMALEPD | Years of schooling || id:ieu-a-1239 | 0.000745099 | 571.4270801 | | 27 | rs11081529 | -0.01311 | 0.2568 | 0.2798 | FEMALEPD | Years of schooling || id:ieu-a-1239 | 0.000701065 | 537.6330055 | | 28 | rs11123818 | 0.02081 | 0.3946 | 0.7407 | FEMALEPD | Years of schooling || id:ieu-a-1239 | 0.000745099 | 571.4270801 | | 29 | rs111821073 | 0.01385 | 0.1633 | 0.0269 | FEMALEPD | Years of schooling || id:ieu-a-1239 | 0.000550286 | 421.9398271 | | 30 | rs112687095 | 0.01325 | 0.1650 | 0.4571 | FEMALEPD | Years of schooling || id:ieu-a-1239 | 0.000547975 | 420.1669707 | | 31 | rs112806496 | 0.0187 | 0.0850 | 0.5637 | FEMALEPD | Years of schooling || id:ieu-a-1239 | 0.000427652 | 327.8679968 | | 32 | rs113182709 | 0.03225 | 0.0238 | 0.8333 | FEMALEPD | Years of schooling || id:ieu-a-1239 | 0.000230087 | 176.3663828 | | 33 | rs113520408 | 0.01304 | 0.2857 | 0.0042 | FEMALEPD | Years of schooling || id:ieu-a-1239 | 0.000679171 | 520.8319741 | | 34 | rs113615161 | -0.01472 | 0.1395 | 0.8377 | FEMALEPD | Years of schooling || id:ieu-a-1239 | 0.000521686 | 399.9989561 | | 35 | rs1143770 | 0.01136 | 0.5918 | 0.4527 | FEMALEPD | Years of schooling || id:ieu-a-1239 | 0.000758085 | 581.3938315 | | 36 | rs115000530 | 0.02892 | 0.0612 | 0.2434 | FEMALEPD | Years of schooling || id:ieu-a-1239 | 0.000342375 | 262.4665066 | | 37 | rs115454970 | -0.01185 | 0.3044 | 0.4840 | FEMALEPD | Years of schooling || id:ieu-a-1239 | 0.000655297 | 502.5112514 | | 38 | rs11601122 | -0.01947 | 0.1497 | 0.3530 | FEMALEPD | Years of schooling || id:ieu-a-1239 | 0.000567024 | 434.781474 | | 39 | rs11620355 | 0.01756 | 0.1156 | 0.1604 | FEMALEPD | Years of schooling || id:ieu-a-1239 | 0.000434776 | 333.3324634 | | 40 | rs11627087 | -0.01788 | 0.0850 | 0.7185 | FEMALEPD | Years of schooling || id:ieu-a-1239 | 0.000401345 | 307.6915047 | | 41 | rs11635092 | -0.01231 | 0.3639 | 0.3818 | FEMALEPD | Years of schooling || id:ieu-a-1239 | 0.000736686 | 564.970277 | | 42 | rs11657342 | 0.01404 | 0.3554 | 0.3004 | FEMALEPD | Years of schooling || id:ieu-a-1239 | 0.000682725 | 523.558843 | | 43 | rs11663602 | -0.01213 | 0.2568 | 0.3619 | FEMALEPD | Years of schooling || id:ieu-a-1239 | 0.000686316 | 526.3144159 | | 44 | rs11678980 | -0.01744 | 0.4456 | 0.7951 | FEMALEPD | Years of schooling || id:ieu-a-1239 | 0.000758085 | 581.3938315 | | 45 | rs11681861 | -0.01435 | 0.1565 | 0.7645 | FEMALEPD | Years of schooling || id:ieu-a-1239 | 0.000503567 | 386.0993785 | | 46 | rs11694904 | 0.01215 | 0.3384 | 0.8726 | FEMALEPD | Years of schooling || id:ieu-a-1239 | 0.000704852 | 540.5391298 | | 47 | rs11732657 | -0.01274 | 0.7007 | 0.5529 | FEMALEPD | Years of schooling || id:ieu-a-1239 | 0.000661945 | 507.6128884 | | 48 | rs117468730 | -0.03521 | 0.0119 | 0.9361 | FEMALEPD | Years of schooling || id:ieu-a-1239 | 0.000218528 | 167.5037505 | | 49 | rs11752914 | -0.01208 | 0.1973 | 0.2448 | FEMALEPD | Years of schooling || id:ieu-a-1239 | 0.000603753 | 462.9617547 | | 50 | rs11772580 | -0.01199 | 0.2534 | 0.1190 | FEMALEPD | Years of schooling || id:ieu-a-1239 | 0.00064878 | 497.5111394 | | 51 | rs11871429 | -0.01425 | 0.2041 | 0.3813 | FEMALEPD | Years of schooling || id:ieu-a-1239 | 0.000645571 | 495.048213 | | 52 | rs12028010 | -0.01696 | 0.2228 | 0.9473 | FEMALEPD | Years of schooling || id:ieu-a-1239 | 0.000645571 | 495.048213 | | 53 | rs12134151 | -0.01245 | 0.5221 | 0.1880 | FEMALEPD | Years of schooling || id:ieu-a-1239 | 0.000766997 | 588.2337589 | | 54 | rs12332731 | 0.01374 | 0.2024 | 0.9212 | FEMALEPD | Years of schooling || id:ieu-a-1239 | 0.000598218 | 458.7143992 | | 55 | rs12375949 | 0.01447 | 0.5697 | 0.3221 | FEMALEPD | Years of schooling || id:ieu-a-1239 | 0.000758085 | 581.3938315 | | 56 | rs12468040 | -0.01432 | 0.6037 | 0.4135 | FEMALEPD | Years of schooling || id:ieu-a-1239 | 0.000745099 | 571.4270801 | | 57 | rs12503522 | -0.01125 | 0.2483 | 0.7154 | FEMALEPD | Years of schooling || id:ieu-a-1239 | 0.000693612 | 531.9135054 | | 58 | rs12519073 | -0.01221 | 0.2381 | 0.4122 | FEMALEPD | Years of schooling || id:ieu-a-1239 | 0.000645571 | 495.048213 | | 59 | rs12574281 | 0.01077 | 0.3997 | 0.8317 | FEMALEPD | Years of schooling || id:ieu-a-1239 | 0.000740868 | 568.1803353 | | 60 | rs12602286 | 0.01701 | 0.8861 | 0.9759 | FEMALEPD | Years of schooling || id:ieu-a-1239 | 0.000511462 | 392.1558393 | | 61 | rs12643771 | 0.01518 | 0.3112 | 0.1891 | FEMALEPD | Years of schooling || id:ieu-a-1239 | 0.00070868 | 543.4768425 | | 62 | rs12682775 | 0.01187 | 0.2143 | 0.3895 | FEMALEPD | Years of schooling || id:ieu-a-1239 | 0.000639246 | 490.1947991 | | 63 | rs12804787 | -0.01814 | 0.0680 | 0.3609 | FEMALEPD | Years of schooling || id:ieu-a-1239 | 0.000398891 | 305.8095995 | | 64 | rs1291818 | -0.01085 | 0.5153 | 0.7198 | FEMALEPD | Years of schooling || id:ieu-a-1239 | 0.000766997 | 588.2337589 | | 65 | rs12940014 | 0.00936 | 0.5204 | 0.9728 | FEMALEPD | Years of schooling || id:ieu-a-1239 | 0.000766997 | 588.2337589 | | 66 | rs12955211 | 0.01097 | 0.3554 | 0.0092 | FEMALEPD | Years of schooling || id:ieu-a-1239 | 0.000716462 | 549.4491155 | | 67 | rs13010566 | 0.0106 | 0.5612 | 0.4898 | FEMALEPD | Years of schooling || id:ieu-a-1239 | 0.000766997 | 588.2337589 | | 68 | rs13029509 | -0.01049 | 0.4677 | 0.4506 | FEMALEPD | Years of schooling || id:ieu-a-1239 | 0.000766997 | 588.2337589 | | 69 | rs13090388 | 0.02852 | 0.3095 | 0.7405 | FEMALEPD | Years of schooling || id:ieu-a-1239 | 0.00070868 | 543.4768425 | | 70 | rs13130765 | -0.01014 | 0.4558 | 0.8008 | FEMALEPD | Years of schooling || id:ieu-a-1239 | 0.000753706 | 578.0331735 | | 71 | rs13141210 | 0.01361 | 0.5085 | 0.3900 | FEMALEPD | Years of schooling || id:ieu-a-1239 | 0.000758085 | 581.3938315 | | 72 | rs13145650 | -0.01918 | 0.9082 | 0.7477 | FEMALEPD | Years of schooling || id:ieu-a-1239 | 0.000426255 | 326.7965327 | | 73 | rs1334297 | 0.02449 | 0.7840 | 0.4458 | FEMALEPD | Years of schooling || id:ieu-a-1239 | 0.000679171 | 520.8319741 | | 74 | rs13422673 | -0.01201 | 0.4847 | 0.6394 | FEMALEPD | Years of schooling || id:ieu-a-1239 | 0.000766997 | 588.2337589 | | 75 | rs1363862 | -0.01171 | 0.2602 | 0.6372 | FEMALEPD | Years of schooling || id:ieu-a-1239 | 0.000679171 | 520.8319741 | | 76 | rs1381247 | -0.01013 | 0.2908 | 0.9813 | FEMALEPD | Years of schooling || id:ieu-a-1239 | 0.000716462 | 549.4491155 | | 77 | rs1391438 | -0.0167 | 0.6854 | 0.8732 | FEMALEPD | Years of schooling || id:ieu-a-1239 | 0.000712549 | 546.4466613 | | 78 | rs1427298 | 0.0102 | 0.4116 | 0.3598 | FEMALEPD | Years of schooling || id:ieu-a-1239 | 0.000758085 | 581.3938315 | | 79 | rs1450782 | -0.00945 | 0.6020 | 0.0057 | FEMALEPD | Years of schooling || id:ieu-a-1239 | 0.000753706 | 578.0331735 | | 80 | rs1455350 | -0.01614 | 0.4711 | 0.6439 | FEMALEPD | Years of schooling || id:ieu-a-1239 | 0.000766997 | 588.2337589 | | 81 | rs152603 | 0.01019 | 0.3861 | 0.0221 | FEMALEPD | Years of schooling || id:ieu-a-1239 | 0.000736686 | 564.970277 | | 82 | rs1558727 | -0.01069 | 0.4847 | 0.5661 | FEMALEPD | Years of schooling || id:ieu-a-1239 | 0.000766997 | 588.2337589 | | 83 | rs1566085 | 0.01645 | 0.5697 | 0.5153 | FEMALEPD | Years of schooling || id:ieu-a-1239 | 0.000762515 | 584.7937954 | | 84 | rs1569092 | 0.01807 | 0.1820 | 0.5850 | FEMALEPD | Years of schooling || id:ieu-a-1239 | 0.000557337 | 427.3493121 | | 85 | rs1584469 | -0.01303 | 0.3112 | 0.3646 | FEMALEPD | Years of schooling || id:ieu-a-1239 | 0.000704852 | 540.5391298 | | 86 | rs1592757 | -0.01045 | 0.3759 | 0.5192 | FEMALEPD | Years of schooling || id:ieu-a-1239 | 0.000724417 | 555.5541057 | | 87 | rs1595973 | -0.01002 | 0.5595 | 0.2617 | FEMALEPD | Years of schooling || id:ieu-a-1239 | 0.000753706 | 578.0331735 | | 88 | rs1618725 | 0.01477 | 0.5204 | 0.1061 | FEMALEPD | Years of schooling || id:ieu-a-1239 | 0.000749378 | 574.7111438 | | 89 | rs1620977 | -0.02046 | 0.6905 | 0.2715 | FEMALEPD | Years of schooling || id:ieu-a-1239 | 0.00066873 | 512.8191745 | | 90 | rs1671770 | -0.01342 | 0.8061 | 0.8260 | FEMALEPD | Years of schooling || id:ieu-a-1239 | 0.000584813 | 448.429323 | | 91 | rs16846463 | -0.02256 | 0.1173 | 0.8409 | FEMALEPD | Years of schooling || id:ieu-a-1239 | 0.000460881 | 353.3559683 | | 92 | rs16854920 | 0.01007 | 0.3554 | 0.1335 | FEMALEPD | Years of schooling || id:ieu-a-1239 | 0.000720417 | 552.484746 | | 93 | rs1689510 | 0.01761 | 0.3435 | 0.3479 | FEMALEPD | Years of schooling || id:ieu-a-1239 | 0.000724417 | 555.5541057 | | 94 | rs16995054 | -0.0139 | 0.2007 | 0.6001 | FEMALEPD | Years of schooling || id:ieu-a-1239 | 0.00062696 | 480.7679761 | | 95 | rs17048855 | 0.01184 | 0.3248 | 0.5034 | FEMALEPD | Years of schooling || id:ieu-a-1239 | 0.000728461 | 558.6577599 | | 96 | rs17110109 | 0.01023 | 0.3776 | 0.1661 | FEMALEPD | Years of schooling || id:ieu-a-1239 | 0.000745099 | 571.4270801 | | 97 | rs17126938 | 0.01536 | 0.1207 | 0.9997 | FEMALEPD | Years of schooling || id:ieu-a-1239 | 0.000521686 | 399.9989561 | | 98 | rs17425572 | -0.01224 | 0.5425 | 0.2792 | FEMALEPD | Years of schooling || id:ieu-a-1239 | 0.000766997 | 588.2337589 | | 99 | rs17489649 | -0.0139 | 0.3265 | 0.3228 | FEMALEPD | Years of schooling || id:ieu-a-1239 | 0.000720417 | 552.484746 | | 100 | rs175325 | -0.01179 | 0.5816 | 0.6852 | FEMALEPD | Years of schooling || id:ieu-a-1239 | 0.000749378 | 574.7111438 | | 101 | rs17551064 | -0.01493 | 0.1599 | 0.0988 | FEMALEPD | Years of schooling || id:ieu-a-1239 | 0.000567024 | 434.781474 | | 102 | rs17563464 | -0.01477 | 0.2041 | 0.4796 | FEMALEPD | Years of schooling || id:ieu-a-1239 | 0.000615138 | 471.6968822 | | 103 | rs17565975 | -0.01142 | 0.5306 | 0.1885 | FEMALEPD | Years of schooling || id:ieu-a-1239 | 0.000762515 | 584.7937954 | | 104 | rs17598675 | 0.01199 | 0.5187 | 0.0560 | FEMALEPD | Years of schooling || id:ieu-a-1239 | 0.000766997 | 588.2337589 | | 105 | rs176218 | 0.01883 | 0.2007 | 0.1735 | FEMALEPD | Years of schooling || id:ieu-a-1239 | 0.00060656 | 465.1150652 | | 106 | rs1827540 | -0.0106 | 0.4660 | 0.2274 | FEMALEPD | Years of schooling || id:ieu-a-1239 | 0.000766997 | 588.2337589 | | 107 | rs1866823 | 0.01009 | 0.5510 | 0.5655 | FEMALEPD | Years of schooling || id:ieu-a-1239 | 0.000762515 | 584.7937954 | | 108 | rs1882273 | -0.01231 | 0.3469 | 0.2731 | FEMALEPD | Years of schooling || id:ieu-a-1239 | 0.000720417 | 552.484746 | | 109 | rs192436652 | -0.03497 | 0.0221 | 0.3825 | FEMALEPD | Years of schooling || id:ieu-a-1239 | 0.000239373 | 183.4857597 | | 110 | rs1925576 | 0.00997 | 0.4422 | 0.7477 | FEMALEPD | Years of schooling || id:ieu-a-1239 | 0.000762515 | 584.7937954 | | 111 | rs1947114 | 0.01071 | 0.2551 | 0.3242 | FEMALEPD | Years of schooling || id:ieu-a-1239 | 0.000679171 | 520.8319741 | | 112 | rs1949226 | -0.00965 | 0.5901 | 0.3582 | FEMALEPD | Years of schooling || id:ieu-a-1239 | 0.000749378 | 574.7111438 | | 113 | rs1964927 | -0.01423 | 0.6378 | 0.0206 | FEMALEPD | Years of schooling || id:ieu-a-1239 | 0.000736686 | 564.970277 | | 114 | rs2052285 | 0.01123 | 0.5765 | 0.8104 | FEMALEPD | Years of schooling || id:ieu-a-1239 | 0.000745099 | 571.4270801 | | 115 | rs2067854 | 0.01477 | 0.1820 | 0.4748 | FEMALEPD | Years of schooling || id:ieu-a-1239 | 0.000623962 | 478.4676508 | | 116 | rs2179152 | 0.01455 | 0.6429 | 0.8591 | FEMALEPD | Years of schooling || id:ieu-a-1239 | 0.000740868 | 568.1803353 | | 117 | rs2182505 | -0.01086 | 0.7364 | 0.1941 | FEMALEPD | Years of schooling || id:ieu-a-1239 | 0.000679171 | 520.8319741 | | 118 | rs225291 | -0.01205 | 0.8027 | 0.0012 | FEMALEPD | Years of schooling || id:ieu-a-1239 | 0.000609393 | 467.2885001 | | 119 | rs2256965 | -0.01128 | 0.5425 | 0.2403 | FEMALEPD | Years of schooling || id:ieu-a-1239 | 0.000740868 | 568.1803353 | | 120 | rs2283076 | -0.01143 | 0.2143 | 0.5544 | FEMALEPD | Years of schooling || id:ieu-a-1239 | 0.000639246 | 490.1947991 | | 121 | rs2287838 | -0.01152 | 0.5340 | 0.6001 | FEMALEPD | Years of schooling || id:ieu-a-1239 | 0.000762515 | 584.7937954 | | 122 | rs2302761 | 0.01354 | 0.1905 | 0.0726 | FEMALEPD | Years of schooling || id:ieu-a-1239 | 0.000623962 | 478.4676508 | | 123 | rs2347526 | 0.01395 | 0.6378 | 0.4475 | FEMALEPD | Years of schooling || id:ieu-a-1239 | 0.000728461 | 558.6577599 | | 124 | rs2414072 | -0.01005 | 0.4864 | 0.1153 | FEMALEPD | Years of schooling || id:ieu-a-1239 | 0.000762515 | 584.7937954 | | 125 | rs242093 | -0.01031 | 0.5476 | 0.2986 | FEMALEPD | Years of schooling || id:ieu-a-1239 | 0.000758085 | 581.3938315 | | 126 | rs2441111 | 0.01087 | 0.5323 | 0.2424 | FEMALEPD | Years of schooling || id:ieu-a-1239 | 0.000766997 | 588.2337589 | | 127 | rs2447535 | 0.01181 | 0.7245 | 0.6842 | FEMALEPD | Years of schooling || id:ieu-a-1239 | 0.000704852 | 540.5391298 | | 128 | rs2478208 | -0.0106 | 0.5000 | 0.8411 | FEMALEPD | Years of schooling || id:ieu-a-1239 | 0.000766997 | 588.2337589 | | 129 | rs2545798 | -0.01346 | 0.4949 | 0.4587 | FEMALEPD | Years of schooling || id:ieu-a-1239 | 0.000762515 | 584.7937954 | | 130 | rs2554835 | 0.00974 | 0.3980 | 0.3118 | FEMALEPD | Years of schooling || id:ieu-a-1239 | 0.000745099 | 571.4270801 | | 131 | rs2570497 | -0.01233 | 0.6735 | 0.1343 | FEMALEPD | Years of schooling || id:ieu-a-1239 | 0.000736686 | 564.970277 | | 132 | rs2725370 | 0.01536 | 0.7109 | 0.9001 | FEMALEPD | Years of schooling || id:ieu-a-1239 | 0.000697318 | 534.7579627 | | 133 | rs277828 | -0.01091 | 0.2568 | 0.5783 | FEMALEPD | Years of schooling || id:ieu-a-1239 | 0.00066532 | 510.2027501 | | 134 | rs2787101 | 0.00968 | 0.6173 | 0.9582 | FEMALEPD | Years of schooling || id:ieu-a-1239 | 0.000749378 | 574.7111438 | | 135 | rs2819336 | -0.01828 | 0.6616 | 0.7870 | FEMALEPD | Years of schooling || id:ieu-a-1239 | 0.000736686 | 564.970277 | | 136 | rs2820314 | -0.011 | 0.3163 | 0.1141 | FEMALEPD | Years of schooling || id:ieu-a-1239 | 0.000724417 | 555.5541057 | | 137 | rs28373063 | 0.01389 | 0.2075 | 0.5448 | FEMALEPD | Years of schooling || id:ieu-a-1239 | 0.000569499 | 436.6800831 | | 138 | rs28513670 | 0.01477 | 0.1531 | 0.9612 | FEMALEPD | Years of schooling || id:ieu-a-1239 | 0.000579617 | 444.4432845 | | 139 | rs2885198 | -0.01025 | 0.5017 | 0.7206 | FEMALEPD | Years of schooling || id:ieu-a-1239 | 0.000766997 | 588.2337589 | | 140 | rs2901616 | 0.00941 | 0.5170 | 0.8966 | FEMALEPD | Years of schooling || id:ieu-a-1239 | 0.000762515 | 584.7937954 | | 141 | rs2905426 | 0.01037 | 0.6446 | 0.0087 | FEMALEPD | Years of schooling || id:ieu-a-1239 | 0.000720417 | 552.484746 | | 142 | rs2923431 | 0.0114 | 0.6446 | 0.9659 | FEMALEPD | Years of schooling || id:ieu-a-1239 | 0.000740868 | 568.1803353 | | 143 | rs2971970 | 0.01654 | 0.7721 | 0.4365 | FEMALEPD | Years of schooling || id:ieu-a-1239 | 0.000629987 | 483.0905267 | | 144 | rs2998315 | 0.01269 | 0.5782 | 0.6916 | FEMALEPD | Years of schooling || id:ieu-a-1239 | 0.000762515 | 584.7937954 | | 145 | rs3013014 | -0.01024 | 0.6105 | 0.0084 | FEMALEPD | Years of schooling || id:ieu-a-1239 | 0.000758085 | 581.3938315 | | 146 | rs301800 | -0.01516 | 0.8197 | 0.0027 | FEMALEPD | Years of schooling || id:ieu-a-1239 | 0.000582203 | 446.4274063 | | 147 | rs3026996 | -0.01537 | 0.2840 | 0.4119 | FEMALEPD | Years of schooling || id:ieu-a-1239 | 0.000655297 | 502.5112514 | | 148 | rs31940 | 0.01548 | 0.1344 | 0.3084 | FEMALEPD | Years of schooling || id:ieu-a-1239 | 0.000530164 | 406.5030042 | | 149 | rs320693 | 0.01204 | 0.4728 | 0.5782 | FEMALEPD | Years of schooling || id:ieu-a-1239 | 0.000766997 | 588.2337589 | | 150 | rs337637 | 0.01123 | 0.3367 | 0.2581 | FEMALEPD | Years of schooling || id:ieu-a-1239 | 0.000736686 | 564.970277 | | 151 | rs34316 | -0.02016 | 0.5799 | 0.5893 | FEMALEPD | Years of schooling || id:ieu-a-1239 | 0.000736686 | 564.970277 | | 152 | rs34394051 | 0.01392 | 0.1599 | 0.9430 | FEMALEPD | Years of schooling || id:ieu-a-1239 | 0.000543411 | 416.6655793 | | 153 | rs34485537 | 0.01075 | 0.3895 | 0.1839 | FEMALEPD | Years of schooling || id:ieu-a-1239 | 0.000753706 | 578.0331735 | | 154 | rs34853711 | -0.01596 | 0.2466 | 0.9829 | FEMALEPD | Years of schooling || id:ieu-a-1239 | 0.000633043 | 485.4356263 | | 155 | rs35039375 | -0.01983 | 0.0935 | 0.2189 | FEMALEPD | Years of schooling || id:ieu-a-1239 | 0.000445159 | 341.2960376 | | 156 | rs35309068 | 0.01321 | 0.4660 | 0.6083 | FEMALEPD | Years of schooling || id:ieu-a-1239 | 0.000762515 | 584.7937954 | | 157 | rs35316276 | 0.01173 | 0.2942 | 0.2248 | FEMALEPD | Years of schooling || id:ieu-a-1239 | 0.000672174 | 515.4625723 | | 158 | rs35417702 | -0.01445 | 0.5765 | 0.3134 | FEMALEPD | Years of schooling || id:ieu-a-1239 | 0.000766997 | 588.2337589 | | 159 | rs35475880 | -0.01511 | 0.1837 | 0.0282 | FEMALEPD | Years of schooling || id:ieu-a-1239 | 0.00062696 | 480.7679761 | | 160 | rs35532491 | 0.02007 | 0.1071 | 0.3483 | FEMALEPD | Years of schooling || id:ieu-a-1239 | 0.000456049 | 349.6494371 | | 161 | rs36083520 | 0.01629 | 0.1667 | 0.5312 | FEMALEPD | Years of schooling || id:ieu-a-1239 | 0.000584813 | 448.429323 | | 162 | rs36119825 | 0.01063 | 0.4694 | 0.9856 | FEMALEPD | Years of schooling || id:ieu-a-1239 | 0.000762515 | 584.7937954 | | 163 | rs363096 | 0.01363 | 0.5748 | 0.3271 | FEMALEPD | Years of schooling || id:ieu-a-1239 | 0.000758085 | 581.3938315 | | 164 | rs3747631 | 0.02207 | 0.2279 | 0.8562 | FEMALEPD | Years of schooling || id:ieu-a-1239 | 0.00062696 | 480.7679761 | | 165 | rs3788556 | -0.01138 | 0.5408 | 0.9585 | FEMALEPD | Years of schooling || id:ieu-a-1239 | 0.000762515 | 584.7937954 | | 166 | rs3800546 | -0.01183 | 0.2704 | 0.0383 | FEMALEPD | Years of schooling || id:ieu-a-1239 | 0.000672174 | 515.4625723 | | 167 | rs3809634 | 0.01058 | 0.3350 | 0.5551 | FEMALEPD | Years of schooling || id:ieu-a-1239 | 0.000704852 | 540.5391298 | | 168 | rs3890802 | -0.01133 | 0.2687 | 0.6812 | FEMALEPD | Years of schooling || id:ieu-a-1239 | 0.000682725 | 523.558843 | | 169 | rs3897821 | -0.01502 | 0.3503 | 0.5568 | FEMALEPD | Years of schooling || id:ieu-a-1239 | 0.000724417 | 555.5541057 | | 170 | rs401687 | 0.01144 | 0.4558 | 0.6364 | FEMALEPD | Years of schooling || id:ieu-a-1239 | 0.000766997 | 588.2337589 | | 171 | rs406413 | -0.01695 | 0.2109 | 0.3753 | FEMALEPD | Years of schooling || id:ieu-a-1239 | 0.000623962 | 478.4676508 | | 172 | rs4073894 | 0.01524 | 0.1769 | 0.1567 | FEMALEPD | Years of schooling || id:ieu-a-1239 | 0.000618052 | 473.9324124 | | 173 | rs4328757 | 0.01067 | 0.6514 | 0.9988 | FEMALEPD | Years of schooling || id:ieu-a-1239 | 0.000749378 | 574.7111438 | | 174 | rs4352658 | -0.0212 | 0.0901 | 0.2017 | FEMALEPD | Years of schooling || id:ieu-a-1239 | 0.000423488 | 324.6744773 | | 175 | rs4369924 | 0.01362 | 0.1684 | 0.7057 | FEMALEPD | Years of schooling || id:ieu-a-1239 | 0.000557337 | 427.3493121 | | 176 | rs4382592 | 0.01636 | 0.6990 | 0.4773 | FEMALEPD | Years of schooling || id:ieu-a-1239 | 0.000704852 | 540.5391298 | | 177 | rs4384309 | 0.0109 | 0.4796 | 0.3854 | FEMALEPD | Years of schooling || id:ieu-a-1239 | 0.000758085 | 581.3938315 | | 178 | rs4392737 | -0.0097 | 0.3827 | 0.1020 | FEMALEPD | Years of schooling || id:ieu-a-1239 | 0.000753706 | 578.0331735 | | 179 | rs4442732 | -0.01063 | 0.5969 | 0.7263 | FEMALEPD | Years of schooling || id:ieu-a-1239 | 0.000740868 | 568.1803353 | | 180 | rs4497562 | -0.01204 | 0.2823 | 0.6494 | FEMALEPD | Years of schooling || id:ieu-a-1239 | 0.000679171 | 520.8319741 | | 181 | rs4667025 | 0.00957 | 0.3878 | 0.7431 | FEMALEPD | Years of schooling || id:ieu-a-1239 | 0.000749378 | 574.7111438 | | 182 | rs4700393 | 0.02086 | 0.5289 | 0.5194 | FEMALEPD | Years of schooling || id:ieu-a-1239 | 0.000766997 | 588.2337589 | | 183 | rs4726070 | 0.01251 | 0.6207 | 0.3420 | FEMALEPD | Years of schooling || id:ieu-a-1239 | 0.000749378 | 574.7111438 | | 184 | rs4733264 | -0.00954 | 0.6310 | 0.7262 | FEMALEPD | Years of schooling || id:ieu-a-1239 | 0.000749378 | 574.7111438 | | 185 | rs4743923 | 0.00985 | 0.3673 | 0.4343 | FEMALEPD | Years of schooling || id:ieu-a-1239 | 0.000740868 | 568.1803353 | | 186 | rs4757957 | 0.0141 | 0.6514 | 0.8515 | FEMALEPD | Years of schooling || id:ieu-a-1239 | 0.00070868 | 543.4768425 | | 187 | rs4766424 | -0.0141 | 0.9116 | 0.7522 | FEMALEPD | Years of schooling || id:ieu-a-1239 | 0.000505518 | 387.5958877 | | 188 | rs4778058 | 0.01017 | 0.5221 | 0.2576 | FEMALEPD | Years of schooling || id:ieu-a-1239 | 0.000766997 | 588.2337589 | | 189 | rs4787457 | -0.01741 | 0.3146 | 0.1107 | FEMALEPD | Years of schooling || id:ieu-a-1239 | 0.000740868 | 568.1803353 | | 190 | rs4810227 | 0.01272 | 0.6344 | 0.1777 | FEMALEPD | Years of schooling || id:ieu-a-1239 | 0.000745099 | 571.4270801 | | 191 | rs4839155 | -0.01251 | 0.2500 | 0.0586 | FEMALEPD | Years of schooling || id:ieu-a-1239 | 0.000652022 | 499.9986951 | | 192 | rs4846724 | 0.01018 | 0.4915 | 0.4857 | FEMALEPD | Years of schooling || id:ieu-a-1239 | 0.000766997 | 588.2337589 | | 193 | rs4870482 | -0.01083 | 0.2585 | 0.3073 | FEMALEPD | Years of schooling || id:ieu-a-1239 | 0.000686316 | 526.3144159 | | 194 | rs4888746 | -0.00952 | 0.3776 | 0.6988 | FEMALEPD | Years of schooling || id:ieu-a-1239 | 0.000749378 | 574.7111438 | | 195 | rs4904523 | -0.00936 | 0.4592 | 0.9523 | FEMALEPD | Years of schooling || id:ieu-a-1239 | 0.000766997 | 588.2337589 | | 196 | rs4945424 | -0.00992 | 0.4150 | 0.6275 | FEMALEPD | Years of schooling || id:ieu-a-1239 | 0.000762515 | 584.7937954 | | 197 | rs4964046 | 0.01053 | 0.3350 | 0.3080 | FEMALEPD | Years of schooling || id:ieu-a-1239 | 0.00073255 | 561.7962866 | | 198 | rs4972400 | 0.01156 | 0.3520 | 0.0972 | FEMALEPD | Years of schooling || id:ieu-a-1239 | 0.000720417 | 552.484746 | | 199 | rs4984541 | 0.01233 | 0.2415 | 0.9970 | FEMALEPD | Years of schooling || id:ieu-a-1239 | 0.000629987 | 483.0905267 | | 200 | rs535307 | -0.01004 | 0.6769 | 0.7257 | FEMALEPD | Years of schooling || id:ieu-a-1239 | 0.00070868 | 543.4768425 | | 201 | rs55736314 | 0.01431 | 0.4167 | 0.9320 | FEMALEPD | Years of schooling || id:ieu-a-1239 | 0.000749378 | 574.7111438 | | 202 | rs55771711 | 0.01555 | 0.2279 | 0.4066 | FEMALEPD | Years of schooling || id:ieu-a-1239 | 0.000655297 | 502.5112514 | | 203 | rs56391344 | 0.01571 | 0.2381 | 0.4241 | FEMALEPD | Years of schooling || id:ieu-a-1239 | 0.000661945 | 507.6128884 | | 204 | rs575113 | 0.01285 | 0.2772 | 0.8707 | FEMALEPD | Years of schooling || id:ieu-a-1239 | 0.000701065 | 537.6330055 | | 205 | rs59123361 | -0.02094 | 0.1054 | 0.8804 | FEMALEPD | Years of schooling || id:ieu-a-1239 | 0.000448217 | 343.6417148 | | 206 | rs59480703 | -0.01237 | 0.1633 | 0.0404 | FEMALEPD | Years of schooling || id:ieu-a-1239 | 0.00060656 | 465.1150652 | | 207 | rs60483752 | 0.01078 | 0.5816 | 0.6746 | FEMALEPD | Years of schooling || id:ieu-a-1239 | 0.000758085 | 581.3938315 | | 208 | rs6122735 | 0.0105 | 0.4133 | 0.2704 | FEMALEPD | Years of schooling || id:ieu-a-1239 | 0.000749378 | 574.7111438 | | 209 | rs6123924 | -0.01528 | 0.1599 | 0.2605 | FEMALEPD | Years of schooling || id:ieu-a-1239 | 0.000554966 | 425.5308043 | | 210 | rs613872 | -0.0175 | 0.8282 | 0.7370 | FEMALEPD | Years of schooling || id:ieu-a-1239 | 0.000574513 | 440.5274847 | | 211 | rs61747885 | 0.01383 | 0.1412 | 0.3251 | FEMALEPD | Years of schooling || id:ieu-a-1239 | 0.000554966 | 425.5308043 | | 212 | rs62097985 | -0.01288 | 0.4116 | 0.3346 | FEMALEPD | Years of schooling || id:ieu-a-1239 | 0.000758085 | 581.3938315 | | 213 | rs62157915 | 0.02091 | 0.0595 | 0.8291 | FEMALEPD | Years of schooling || id:ieu-a-1239 | 0.000374829 | 287.3555719 | | 214 | rs62183776 | -0.01308 | 0.1905 | 0.1738 | FEMALEPD | Years of schooling || id:ieu-a-1239 | 0.000600973 | 460.8282904 | | 215 | rs62184480 | -0.01528 | 0.2449 | 0.7755 | FEMALEPD | Years of schooling || id:ieu-a-1239 | 0.000682725 | 523.558843 | | 216 | rs62439690 | -0.01087 | 0.2670 | 0.1827 | FEMALEPD | Years of schooling || id:ieu-a-1239 | 0.000672174 | 515.4625723 | | 217 | rs62444881 | 0.01815 | 0.1905 | 0.2394 | FEMALEPD | Years of schooling || id:ieu-a-1239 | 0.000600973 | 460.8282904 | | 218 | rs6493265 | -0.01385 | 0.3895 | 0.0125 | FEMALEPD | Years of schooling || id:ieu-a-1239 | 0.000749378 | 574.7111438 | | 219 | rs6513959 | -0.01177 | 0.2789 | 0.6598 | FEMALEPD | Years of schooling || id:ieu-a-1239 | 0.000704852 | 540.5391298 | | 220 | rs6557171 | 0.01567 | 0.7245 | 0.1272 | FEMALEPD | Years of schooling || id:ieu-a-1239 | 0.000720417 | 552.484746 | | 221 | rs663234 | -0.01005 | 0.6122 | 0.3549 | FEMALEPD | Years of schooling || id:ieu-a-1239 | 0.000749378 | 574.7111438 | | 222 | rs66568921 | 0.01565 | 0.3639 | 0.5653 | FEMALEPD | Years of schooling || id:ieu-a-1239 | 0.000716462 | 549.4491155 | | 223 | rs6731373 | -0.01256 | 0.3367 | 0.3442 | FEMALEPD | Years of schooling || id:ieu-a-1239 | 0.000720417 | 552.484746 | | 224 | rs6731967 | -0.01186 | 0.2092 | 0.8038 | FEMALEPD | Years of schooling || id:ieu-a-1239 | 0.000655297 | 502.5112514 | | 225 | rs67885444 | 0.01406 | 0.1718 | 0.6977 | FEMALEPD | Years of schooling || id:ieu-a-1239 | 0.000562139 | 431.0333578 | | 226 | rs67890737 | -0.01141 | 0.3265 | 0.7516 | FEMALEPD | Years of schooling || id:ieu-a-1239 | 0.000728461 | 558.6577599 | | 227 | rs6803651 | 0.01131 | 0.4150 | 0.2690 | FEMALEPD | Years of schooling || id:ieu-a-1239 | 0.000758085 | 581.3938315 | | 228 | rs6805241 | -0.01413 | 0.1973 | 0.2775 | FEMALEPD | Years of schooling || id:ieu-a-1239 | 0.000642393 | 492.6095518 | | 229 | rs6867851 | -0.012 | 0.4235 | 0.5691 | FEMALEPD | Years of schooling || id:ieu-a-1239 | 0.000753706 | 578.0331735 | | 230 | rs6938002 | -0.01008 | 0.3963 | 0.3882 | FEMALEPD | Years of schooling || id:ieu-a-1239 | 0.000753706 | 578.0331735 | | 231 | rs6959891 | -0.01136 | 0.2959 | 0.8546 | FEMALEPD | Years of schooling || id:ieu-a-1239 | 0.000689944 | 529.0991483 | | 232 | rs7012546 | 0.01009 | 0.4201 | 0.2074 | FEMALEPD | Years of schooling || id:ieu-a-1239 | 0.000758085 | 581.3938315 | | 233 | rs7016302 | 0.01243 | 0.1769 | 0.4543 | FEMALEPD | Years of schooling || id:ieu-a-1239 | 0.000571995 | 438.5953466 | | 234 | rs702606 | -0.01427 | 0.1701 | 0.8641 | FEMALEPD | Years of schooling || id:ieu-a-1239 | 0.000521686 | 399.9989561 | | 235 | rs7029718 | 0.02439 | 0.4354 | 0.2023 | FEMALEPD | Years of schooling || id:ieu-a-1239 | 0.000749378 | 574.7111438 | | 236 | rs7031698 | 0.01248 | 0.7755 | 0.8073 | FEMALEPD | Years of schooling || id:ieu-a-1239 | 0.000633043 | 485.4356263 | | 237 | rs710629 | 0.01053 | 0.6565 | 0.4244 | FEMALEPD | Years of schooling || id:ieu-a-1239 | 0.000736686 | 564.970277 | | 238 | rs71646142 | 0.01286 | 0.1735 | 0.0278 | FEMALEPD | Years of schooling || id:ieu-a-1239 | 0.000600973 | 460.8282904 | | 239 | rs7233920 | -0.01315 | 0.2160 | 0.0634 | FEMALEPD | Years of schooling || id:ieu-a-1239 | 0.000645571 | 495.048213 | | 240 | rs7257460 | -0.01145 | 0.2704 | 0.6810 | FEMALEPD | Years of schooling || id:ieu-a-1239 | 0.000689944 | 529.0991483 | | 241 | rs7278859 | 0.01013 | 0.3078 | 0.7614 | FEMALEPD | Years of schooling || id:ieu-a-1239 | 0.000704852 | 540.5391298 | | 242 | rs72807818 | 0.01915 | 0.1241 | 0.1310 | FEMALEPD | Years of schooling || id:ieu-a-1239 | 0.000517548 | 396.8243612 | | 243 | rs72828517 | 0.01836 | 0.1412 | 0.5424 | FEMALEPD | Years of schooling || id:ieu-a-1239 | 0.000582203 | 446.4274063 | | 244 | rs72840994 | 0.01247 | 0.1820 | 0.6941 | FEMALEPD | Years of schooling || id:ieu-a-1239 | 0.000603753 | 462.9617547 | | 245 | rs730384 | 0.01016 | 0.4558 | 0.1265 | FEMALEPD | Years of schooling || id:ieu-a-1239 | 0.000762515 | 584.7937954 | | 246 | rs7315713 | -0.01022 | 0.6633 | 0.2978 | FEMALEPD | Years of schooling || id:ieu-a-1239 | 0.000697318 | 534.7579627 | | 247 | rs7321274 | -0.01275 | 0.1956 | 0.6608 | FEMALEPD | Years of schooling || id:ieu-a-1239 | 0.000618052 | 473.9324124 | | 248 | rs73301698 | -0.01291 | 0.2262 | 0.6777 | FEMALEPD | Years of schooling || id:ieu-a-1239 | 0.00062696 | 480.7679761 | | 249 | rs7332724 | -0.01149 | 0.2619 | 0.5658 | FEMALEPD | Years of schooling || id:ieu-a-1239 | 0.000689944 | 529.0991483 | | 250 | rs73344830 | -0.0172 | 0.6020 | 0.6116 | FEMALEPD | Years of schooling || id:ieu-a-1239 | 0.000758085 | 581.3938315 | | 251 | rs736282 | -0.01082 | 0.5153 | 0.3926 | FEMALEPD | Years of schooling || id:ieu-a-1239 | 0.000766997 | 588.2337589 | | 252 | rs73874335 | -0.0199 | 0.0595 | 0.3354 | FEMALEPD | Years of schooling || id:ieu-a-1239 | 0.000361336 | 277.0075873 | | 253 | rs743316 | -0.01185 | 0.1820 | 0.1599 | FEMALEPD | Years of schooling || id:ieu-a-1239 | 0.00062696 | 480.7679761 | | 254 | rs74643044 | 0.02323 | 0.0272 | 0.9158 | FEMALEPD | Years of schooling || id:ieu-a-1239 | 0.000337942 | 259.0666814 | | 255 | rs74701752 | 0.01591 | 0.0952 | 0.7675 | FEMALEPD | Years of schooling || id:ieu-a-1239 | 0.000457648 | 350.8762773 | | 256 | rs7481514 | 0.01072 | 0.6650 | 0.7743 | FEMALEPD | Years of schooling || id:ieu-a-1239 | 0.00073255 | 561.7962866 | | 257 | rs74998289 | -0.01821 | 0.2398 | 0.0000 | FEMALEPD | Years of schooling || id:ieu-a-1239 | 0.000612252 | 469.4823428 | | 258 | rs7594904 | 0.00969 | 0.4184 | 0.3352 | FEMALEPD | Years of schooling || id:ieu-a-1239 | 0.000753706 | 578.0331735 | | 259 | rs7603132 | 0.01317 | 0.1548 | 0.4377 | FEMALEPD | Years of schooling || id:ieu-a-1239 | 0.00060656 | 465.1150652 | | 260 | rs76076331 | 0.01873 | 0.1310 | 0.9283 | FEMALEPD | Years of schooling || id:ieu-a-1239 | 0.000525891 | 403.2247541 | | 261 | rs7650602 | 0.00939 | 0.4286 | 0.5324 | FEMALEPD | Years of schooling || id:ieu-a-1239 | 0.000762515 | 584.7937954 | | 262 | rs76608582 | 0.02798 | 0.0408 | 0.5357 | FEMALEPD | Years of schooling || id:ieu-a-1239 | 0.000293149 | 224.7185147 | | 263 | rs76878669 | -0.01399 | 0.2534 | 0.3933 | FEMALEPD | Years of schooling || id:ieu-a-1239 | 0.000636129 | 487.803605 | | 264 | rs77025239 | -0.01422 | 0.1088 | 0.8221 | FEMALEPD | Years of schooling || id:ieu-a-1239 | 0.000557337 | 427.3493121 | | 265 | rs77128898 | -0.02769 | 0.0221 | 0.8367 | FEMALEPD | Years of schooling || id:ieu-a-1239 | 0.000270652 | 207.4683382 | | 266 | rs77702622 | -0.02447 | 0.0765 | 0.6878 | FEMALEPD | Years of schooling || id:ieu-a-1239 | 0.000371627 | 284.8995414 | | 267 | rs77719387 | -0.04597 | 0.0170 | 0.5309 | FEMALEPD | Years of schooling || id:ieu-a-1239 | 0.000179705 | 137.7406874 | | 268 | rs77835879 | -0.01601 | 0.0901 | 0.4791 | FEMALEPD | Years of schooling || id:ieu-a-1239 | 0.000452883 | 347.221316 | | 269 | rs7796203 | -0.01074 | 0.5255 | 0.8978 | FEMALEPD | Years of schooling || id:ieu-a-1239 | 0.000762515 | 584.7937954 | | 270 | rs7803932 | 0.0143 | 0.1565 | 0.3899 | FEMALEPD | Years of schooling || id:ieu-a-1239 | 0.000577054 | 442.4767213 | | 271 | rs7808399 | 0.0107 | 0.5476 | 0.0997 | FEMALEPD | Years of schooling || id:ieu-a-1239 | 0.000762515 | 584.7937954 | | 272 | rs7833201 | -0.01532 | 0.1344 | 0.0507 | FEMALEPD | Years of schooling || id:ieu-a-1239 | 0.000497804 | 381.6783932 | | 273 | rs7863447 | 0.01678 | 0.8333 | 0.8406 | FEMALEPD | Years of schooling || id:ieu-a-1239 | 0.000559727 | 429.1834293 | | 274 | rs78721320 | 0.01307 | 0.2041 | 0.1943 | FEMALEPD | Years of schooling || id:ieu-a-1239 | 0.000595488 | 456.6198129 | | 275 | rs790647 | -0.01482 | 0.2347 | 0.6864 | FEMALEPD | Years of schooling || id:ieu-a-1239 | 0.000645571 | 495.048213 | | 276 | rs7920624 | -0.01181 | 0.4932 | 0.9518 | FEMALEPD | Years of schooling || id:ieu-a-1239 | 0.000766997 | 588.2337589 | | 277 | rs7924036 | 0.01501 | 0.5391 | 0.0592 | FEMALEPD | Years of schooling || id:ieu-a-1239 | 0.000766997 | 588.2337589 | | 278 | rs79265434 | 0.02331 | 0.1173 | 0.4182 | FEMALEPD | Years of schooling || id:ieu-a-1239 | 0.000497804 | 381.6783932 | | 279 | rs79269403 | 0.01447 | 0.2228 | 0.9574 | FEMALEPD | Years of schooling || id:ieu-a-1239 | 0.000639246 | 490.1947991 | | 280 | rs7928622 | 0.01011 | 0.3078 | 0.2590 | FEMALEPD | Years of schooling || id:ieu-a-1239 | 0.000720417 | 552.484746 | | 281 | rs795230 | 0.00952 | 0.4184 | 0.5263 | FEMALEPD | Years of schooling || id:ieu-a-1239 | 0.000758085 | 581.3938315 | | 282 | rs79523955 | -0.01802 | 0.0952 | 0.2117 | FEMALEPD | Years of schooling || id:ieu-a-1239 | 0.000460881 | 353.3559683 | | 283 | rs7977614 | 0.01325 | 0.3078 | 0.1906 | FEMALEPD | Years of schooling || id:ieu-a-1239 | 0.000658604 | 505.049187 | | 284 | rs7993663 | 0.0118 | 0.3571 | 0.4725 | FEMALEPD | Years of schooling || id:ieu-a-1239 | 0.00073255 | 561.7962866 | | 285 | rs8008382 | 0.01208 | 0.6871 | 0.2201 | FEMALEPD | Years of schooling || id:ieu-a-1239 | 0.000704852 | 540.5391298 | | 286 | rs80171383 | 0.0145 | 0.1241 | 0.1224 | FEMALEPD | Years of schooling || id:ieu-a-1239 | 0.000541157 | 414.9366764 | | 287 | rs8020034 | 0.01782 | 0.2058 | 0.2001 | FEMALEPD | Years of schooling || id:ieu-a-1239 | 0.000584813 | 448.429323 | | 288 | rs818415 | 0.01235 | 0.1820 | 0.7719 | FEMALEPD | Years of schooling || id:ieu-a-1239 | 0.000595488 | 456.6198129 | | 289 | rs837080 | 0.01092 | 0.4932 | 0.0874 | FEMALEPD | Years of schooling || id:ieu-a-1239 | 0.000766997 | 588.2337589 | | 290 | rs892612 | 0.01464 | 0.8418 | 0.5213 | FEMALEPD | Years of schooling || id:ieu-a-1239 | 0.000550286 | 421.9398271 | | 291 | rs894067 | 0.01041 | 0.3929 | 0.1966 | FEMALEPD | Years of schooling || id:ieu-a-1239 | 0.000745099 | 571.4270801 | | 292 | rs9289300 | 0.01512 | 0.1837 | 0.4613 | FEMALEPD | Years of schooling || id:ieu-a-1239 | 0.000557337 | 427.3493121 | | 293 | rs9320493 | -0.01394 | 0.8639 | 0.1631 | FEMALEPD | Years of schooling || id:ieu-a-1239 | 0.000543411 | 416.6655793 | | 294 | rs9342482 | 0.01264 | 0.2908 | 0.8206 | FEMALEPD | Years of schooling || id:ieu-a-1239 | 0.000661945 | 507.6128884 | | 295 | rs9349956 | 0.01881 | 0.2398 | 0.4818 | FEMALEPD | Years of schooling || id:ieu-a-1239 | 0.000579617 | 444.4432845 | | 296 | rs9372625 | 0.02383 | 0.4133 | 0.6790 | FEMALEPD | Years of schooling || id:ieu-a-1239 | 0.000740868 | 568.1803353 | | 297 | rs9384679 | -0.00959 | 0.4082 | 0.5388 | FEMALEPD | Years of schooling || id:ieu-a-1239 | 0.000740868 | 568.1803353 | | 298 | rs9386319 | 0.00991 | 0.4269 | 0.5994 | FEMALEPD | Years of schooling || id:ieu-a-1239 | 0.000749378 | 574.7111438 | | 299 | rs9386787 | 0.00958 | 0.5136 | 0.5374 | FEMALEPD | Years of schooling || id:ieu-a-1239 | 0.000766997 | 588.2337589 | | 300 | rs9436866 | 0.01882 | 0.0952 | 0.7578 | FEMALEPD | Years of schooling || id:ieu-a-1239 | 0.000451317 | 346.0198582 | | 301 | rs9503598 | 0.01079 | 0.4388 | 0.4683 | FEMALEPD | Years of schooling || id:ieu-a-1239 | 0.000762515 | 584.7937954 | | 302 | rs9529119 | -0.01295 | 0.8027 | 0.0046 | FEMALEPD | Years of schooling || id:ieu-a-1239 | 0.000639246 | 490.1947991 | | 303 | rs9556958 | -0.0108 | 0.5289 | 0.9926 | FEMALEPD | Years of schooling || id:ieu-a-1239 | 0.000766997 | 588.2337589 | | 304 | rs9616906 | 0.01497 | 0.4235 | 0.1426 | FEMALEPD | Years of schooling || id:ieu-a-1239 | 0.000758085 | 581.3938315 | | 305 | rs9679654 | 0.01042 | 0.4847 | 0.6838 | FEMALEPD | Years of schooling || id:ieu-a-1239 | 0.000758085 | 581.3938315 | | 306 | rs969512 | 0.01249 | 0.2959 | 0.6880 | FEMALEPD | Years of schooling || id:ieu-a-1239 | 0.000728461 | 558.6577599 | | 307 | rs9704097 | -0.0103 | 0.4728 | 0.9731 | FEMALEPD | Years of schooling || id:ieu-a-1239 | 0.000762515 | 584.7937954 | | 308 | rs9882532 | -0.01208 | 0.3639 | 0.8434 | FEMALEPD | Years of schooling || id:ieu-a-1239 | 0.000736686 | 564.970277 | | 309 | rs9914918 | 0.01155 | 0.2823 | 0.9939 | FEMALEPD | Years of schooling || id:ieu-a-1239 | 0.000689944 | 529.0991483 | | 310 | rs9933256 | -0.01134 | 0.4082 | 0.2939 | FEMALEPD | Years of schooling || id:ieu-a-1239 | 0.000758085 | 581.3938315 | | 311 | rs9936270 | -0.0136 | 0.3078 | 0.0292 | FEMALEPD | Years of schooling || id:ieu-a-1239 | 0.000658604 | 505.049187 | | 312 | rs9938678 | 0.01355 | 0.2432 | 0.3615 | FEMALEPD | Years of schooling || id:ieu-a-1239 | 0.000636129 | 487.803605 | | 313 | rs9964724 | 0.01978 | 0.6599 | 0.1176 | FEMALEPD | Years of schooling || id:ieu-a-1239 | 0.000712549 | 546.4466613 | | 314 | rs9995567 | 0.00998 | 0.3827 | 0.5922 | FEMALEPD | Years of schooling || id:ieu-a-1239 | 0.00073255 | 561.7962866 | | 1 | rs10073890 | -0.01262 | 0.7364 | 0.8807 | MALEPD | Years of schooling || id:ieu-a-1239 | 0.00066532 | 510.2027501 | | 2 | rs1008078 | -0.01738 | 0.4099 | 0.5801 | MALEPD | Years of schooling || id:ieu-a-1239 | 0.000753706 | 578.0331735 | | 3 | rs10189857 | -0.01725 | 0.4184 | 0.7026 | MALEPD | Years of schooling || id:ieu-a-1239 | 0.000762515 | 584.7937954 | | 4 | rs10191758 | 0.01631 | 0.3810 | 0.2072 | MALEPD | Years of schooling || id:ieu-a-1239 | 0.000745099 | 571.4270801 | | 5 | rs10205801 | -0.01053 | 0.5068 | 0.0662 | MALEPD | Years of schooling || id:ieu-a-1239 | 0.000762515 | 584.7937954 | | 6 | rs10215082 | 0.01303 | 0.5612 | 0.9560 | MALEPD | Years of schooling || id:ieu-a-1239 | 0.000758085 | 581.3938315 | | 7 | rs10240905 | 0.01167 | 0.6684 | 0.0993 | MALEPD | Years of schooling || id:ieu-a-1239 | 0.000736686 | 564.970277 | | 8 | rs10456918 | 0.01485 | 0.1820 | 0.3815 | MALEPD | Years of schooling || id:ieu-a-1239 | 0.000582203 | 446.4274063 | | 9 | rs10460095 | -0.01066 | 0.5867 | 0.7397 | MALEPD | Years of schooling || id:ieu-a-1239 | 0.000762515 | 584.7937954 | | 10 | rs1051474 | 0.01301 | 0.2738 | 0.4423 | MALEPD | Years of schooling || id:ieu-a-1239 | 0.000693612 | 531.9135054 | | 11 | rs10760023 | 0.01095 | 0.3316 | 0.7144 | MALEPD | Years of schooling || id:ieu-a-1239 | 0.000712549 | 546.4466613 | | 12 | rs10765775 | 0.01488 | 0.3963 | 0.4240 | MALEPD | Years of schooling || id:ieu-a-1239 | 0.000740868 | 568.1803353 | | 13 | rs10772644 | 0.01614 | 0.8929 | 0.9768 | MALEPD | Years of schooling || id:ieu-a-1239 | 0.000488486 | 374.5308578 | | 14 | rs10773002 | -0.02191 | 0.7211 | 0.0557 | MALEPD | Years of schooling || id:ieu-a-1239 | 0.000661945 | 507.6128884 | | 15 | rs10797055 | 0.00986 | 0.5102 | 0.6415 | MALEPD | Years of schooling || id:ieu-a-1239 | 0.000758085 | 581.3938315 | | 16 | rs10798418 | -0.00957 | 0.4660 | 0.3531 | MALEPD | Years of schooling || id:ieu-a-1239 | 0.000753706 | 578.0331735 | | 17 | rs10856785 | -0.01132 | 0.7296 | 0.7140 | MALEPD | Years of schooling || id:ieu-a-1239 | 0.000679171 | 520.8319741 | | 18 | rs10862376 | 0.01616 | 0.1361 | 0.0676 | MALEPD | Years of schooling || id:ieu-a-1239 | 0.000545683 | 418.4089499 | | 19 | rs10875121 | 0.01834 | 0.8571 | 0.2634 | MALEPD | Years of schooling || id:ieu-a-1239 | 0.000577054 | 442.4767213 | | 20 | rs10887801 | 0.01087 | 0.4371 | 0.8803 | MALEPD | Years of schooling || id:ieu-a-1239 | 0.000762515 | 584.7937954 | | 21 | rs10940921 | -0.01089 | 0.5697 | 0.4940 | MALEPD | Years of schooling || id:ieu-a-1239 | 0.000736686 | 564.970277 | | 22 | rs10963297 | 0.01904 | 0.2517 | 0.8713 | MALEPD | Years of schooling || id:ieu-a-1239 | 0.000658604 | 505.049187 | | 23 | rs10994777 | 0.0146 | 0.1395 | 0.4823 | MALEPD | Years of schooling || id:ieu-a-1239 | 0.000562139 | 431.0333578 | | 24 | rs11023749 | 0.01132 | 0.6701 | 0.0552 | MALEPD | Years of schooling || id:ieu-a-1239 | 0.000724417 | 555.5541057 | | 25 | rs1105307 | -0.01173 | 0.2449 | 0.7784 | MALEPD | Years of schooling || id:ieu-a-1239 | 0.00066873 | 512.8191745 | | 26 | rs1106090 | 0.01173 | 0.6259 | 0.3696 | MALEPD | Years of schooling || id:ieu-a-1239 | 0.000745099 | 571.4270801 | | 27 | rs11081529 | -0.01311 | 0.2568 | 0.9637 | MALEPD | Years of schooling || id:ieu-a-1239 | 0.000701065 | 537.6330055 | | 28 | rs11123818 | 0.02081 | 0.3946 | 0.5769 | MALEPD | Years of schooling || id:ieu-a-1239 | 0.000745099 | 571.4270801 | | 29 | rs111821073 | 0.01385 | 0.1633 | 0.2158 | MALEPD | Years of schooling || id:ieu-a-1239 | 0.000550286 | 421.9398271 | | 30 | rs112687095 | 0.01325 | 0.1650 | 0.7335 | MALEPD | Years of schooling || id:ieu-a-1239 | 0.000547975 | 420.1669707 | | 31 | rs112806496 | 0.0187 | 0.0850 | 0.6648 | MALEPD | Years of schooling || id:ieu-a-1239 | 0.000427652 | 327.8679968 | | 32 | rs113182709 | 0.03225 | 0.0238 | 0.2402 | MALEPD | Years of schooling || id:ieu-a-1239 | 0.000230087 | 176.3663828 | | 33 | rs113520408 | 0.01304 | 0.2857 | 0.1118 | MALEPD | Years of schooling || id:ieu-a-1239 | 0.000679171 | 520.8319741 | | 34 | rs113615161 | -0.01472 | 0.1395 | 0.0044 | MALEPD | Years of schooling || id:ieu-a-1239 | 0.000521686 | 399.9989561 | | 35 | rs1143770 | 0.01136 | 0.5918 | 0.3935 | MALEPD | Years of schooling || id:ieu-a-1239 | 0.000758085 | 581.3938315 | | 36 | rs115000530 | 0.02892 | 0.0612 | 0.9338 | MALEPD | Years of schooling || id:ieu-a-1239 | 0.000342375 | 262.4665066 | | 37 | rs115454970 | -0.01185 | 0.3044 | 0.0961 | MALEPD | Years of schooling || id:ieu-a-1239 | 0.000655297 | 502.5112514 | | 38 | rs11601122 | -0.01947 | 0.1497 | 0.4145 | MALEPD | Years of schooling || id:ieu-a-1239 | 0.000567024 | 434.781474 | | 39 | rs11620355 | 0.01756 | 0.1156 | 0.5854 | MALEPD | Years of schooling || id:ieu-a-1239 | 0.000434776 | 333.3324634 | | 40 | rs11627087 | -0.01788 | 0.0850 | 0.0273 | MALEPD | Years of schooling || id:ieu-a-1239 | 0.000401345 | 307.6915047 | | 41 | rs11635092 | -0.01231 | 0.3639 | 0.5297 | MALEPD | Years of schooling || id:ieu-a-1239 | 0.000736686 | 564.970277 | | 42 | rs11657342 | 0.01404 | 0.3554 | 0.6814 | MALEPD | Years of schooling || id:ieu-a-1239 | 0.000682725 | 523.558843 | | 43 | rs11663602 | -0.01213 | 0.2568 | 0.0157 | MALEPD | Years of schooling || id:ieu-a-1239 | 0.000686316 | 526.3144159 | | 44 | rs11678980 | -0.01744 | 0.4456 | 0.2245 | MALEPD | Years of schooling || id:ieu-a-1239 | 0.000758085 | 581.3938315 | | 45 | rs11681861 | -0.01435 | 0.1565 | 0.4380 | MALEPD | Years of schooling || id:ieu-a-1239 | 0.000503567 | 386.0993785 | | 46 | rs11694904 | 0.01215 | 0.3384 | 0.4776 | MALEPD | Years of schooling || id:ieu-a-1239 | 0.000704852 | 540.5391298 | | 47 | rs11732657 | -0.01274 | 0.7007 | 0.9968 | MALEPD | Years of schooling || id:ieu-a-1239 | 0.000661945 | 507.6128884 | | 48 | rs117468730 | -0.03521 | 0.0119 | 0.2698 | MALEPD | Years of schooling || id:ieu-a-1239 | 0.000218528 | 167.5037505 | | 49 | rs11752914 | -0.01208 | 0.1973 | 0.1236 | MALEPD | Years of schooling || id:ieu-a-1239 | 0.000603753 | 462.9617547 | | 50 | rs11772580 | -0.01199 | 0.2534 | 0.2053 | MALEPD | Years of schooling || id:ieu-a-1239 | 0.00064878 | 497.5111394 | | 51 | rs11871429 | -0.01425 | 0.2041 | 0.9941 | MALEPD | Years of schooling || id:ieu-a-1239 | 0.000645571 | 495.048213 | | 52 | rs12028010 | -0.01696 | 0.2228 | 0.1844 | MALEPD | Years of schooling || id:ieu-a-1239 | 0.000645571 | 495.048213 | | 53 | rs12134151 | -0.01245 | 0.5221 | 0.5781 | MALEPD | Years of schooling || id:ieu-a-1239 | 0.000766997 | 588.2337589 | | 54 | rs12332731 | 0.01374 | 0.2024 | 0.7550 | MALEPD | Years of schooling || id:ieu-a-1239 | 0.000598218 | 458.7143992 | | 55 | rs12375949 | 0.01447 | 0.5697 | 0.5323 | MALEPD | Years of schooling || id:ieu-a-1239 | 0.000758085 | 581.3938315 | | 56 | rs12468040 | -0.01432 | 0.6037 | 0.6824 | MALEPD | Years of schooling || id:ieu-a-1239 | 0.000745099 | 571.4270801 | | 57 | rs12503522 | -0.01125 | 0.2483 | 0.7694 | MALEPD | Years of schooling || id:ieu-a-1239 | 0.000693612 | 531.9135054 | | 58 | rs12519073 | -0.01221 | 0.2381 | 0.0858 | MALEPD | Years of schooling || id:ieu-a-1239 | 0.000645571 | 495.048213 | | 59 | rs12574281 | 0.01077 | 0.3997 | 0.3639 | MALEPD | Years of schooling || id:ieu-a-1239 | 0.000740868 | 568.1803353 | | 60 | rs12602286 | 0.01701 | 0.8861 | 0.0335 | MALEPD | Years of schooling || id:ieu-a-1239 | 0.000511462 | 392.1558393 | | 61 | rs12643771 | 0.01518 | 0.3112 | 0.7269 | MALEPD | Years of schooling || id:ieu-a-1239 | 0.00070868 | 543.4768425 | | 62 | rs12682775 | 0.01187 | 0.2143 | 0.6411 | MALEPD | Years of schooling || id:ieu-a-1239 | 0.000639246 | 490.1947991 | | 63 | rs12804787 | -0.01814 | 0.0680 | 0.3120 | MALEPD | Years of schooling || id:ieu-a-1239 | 0.000398891 | 305.8095995 | | 64 | rs1291818 | -0.01085 | 0.5153 | 0.1975 | MALEPD | Years of schooling || id:ieu-a-1239 | 0.000766997 | 588.2337589 | | 65 | rs12940014 | 0.00936 | 0.5204 | 0.0764 | MALEPD | Years of schooling || id:ieu-a-1239 | 0.000766997 | 588.2337589 | | 66 | rs12955211 | 0.01097 | 0.3554 | 0.8029 | MALEPD | Years of schooling || id:ieu-a-1239 | 0.000716462 | 549.4491155 | | 67 | rs13010566 | 0.0106 | 0.5612 | 0.0578 | MALEPD | Years of schooling || id:ieu-a-1239 | 0.000766997 | 588.2337589 | | 68 | rs13029509 | -0.01049 | 0.4677 | 0.4973 | MALEPD | Years of schooling || id:ieu-a-1239 | 0.000766997 | 588.2337589 | | 69 | rs13090388 | 0.02852 | 0.3095 | 0.3154 | MALEPD | Years of schooling || id:ieu-a-1239 | 0.00070868 | 543.4768425 | | 70 | rs13130765 | -0.01014 | 0.4558 | 0.2240 | MALEPD | Years of schooling || id:ieu-a-1239 | 0.000753706 | 578.0331735 | | 71 | rs13141210 | 0.01361 | 0.5085 | 0.2954 | MALEPD | Years of schooling || id:ieu-a-1239 | 0.000758085 | 581.3938315 | | 72 | rs13145650 | -0.01918 | 0.9082 | 0.2538 | MALEPD | Years of schooling || id:ieu-a-1239 | 0.000426255 | 326.7965327 | | 73 | rs1334297 | 0.02449 | 0.7840 | 0.5587 | MALEPD | Years of schooling || id:ieu-a-1239 | 0.000679171 | 520.8319741 | | 74 | rs13422673 | -0.01201 | 0.4847 | 0.0071 | MALEPD | Years of schooling || id:ieu-a-1239 | 0.000766997 | 588.2337589 | | 75 | rs1363862 | -0.01171 | 0.2602 | 0.3184 | MALEPD | Years of schooling || id:ieu-a-1239 | 0.000679171 | 520.8319741 | | 76 | rs1381247 | -0.01013 | 0.2908 | 0.9152 | MALEPD | Years of schooling || id:ieu-a-1239 | 0.000716462 | 549.4491155 | | 77 | rs1391438 | -0.0167 | 0.6854 | 0.6165 | MALEPD | Years of schooling || id:ieu-a-1239 | 0.000712549 | 546.4466613 | | 78 | rs1427298 | 0.0102 | 0.4116 | 0.0490 | MALEPD | Years of schooling || id:ieu-a-1239 | 0.000758085 | 581.3938315 | | 79 | rs1450782 | -0.00945 | 0.6020 | 0.3918 | MALEPD | Years of schooling || id:ieu-a-1239 | 0.000753706 | 578.0331735 | | 80 | rs1455350 | -0.01614 | 0.4711 | 0.0052 | MALEPD | Years of schooling || id:ieu-a-1239 | 0.000766997 | 588.2337589 | | 81 | rs152603 | 0.01019 | 0.3861 | 0.1585 | MALEPD | Years of schooling || id:ieu-a-1239 | 0.000736686 | 564.970277 | | 82 | rs1558727 | -0.01069 | 0.4847 | 0.6021 | MALEPD | Years of schooling || id:ieu-a-1239 | 0.000766997 | 588.2337589 | | 83 | rs1566085 | 0.01645 | 0.5697 | 0.6109 | MALEPD | Years of schooling || id:ieu-a-1239 | 0.000762515 | 584.7937954 | | 84 | rs1569092 | 0.01807 | 0.1820 | 0.2242 | MALEPD | Years of schooling || id:ieu-a-1239 | 0.000557337 | 427.3493121 | | 85 | rs1584469 | -0.01303 | 0.3112 | 0.7844 | MALEPD | Years of schooling || id:ieu-a-1239 | 0.000704852 | 540.5391298 | | 86 | rs1592757 | -0.01045 | 0.3759 | 0.7847 | MALEPD | Years of schooling || id:ieu-a-1239 | 0.000724417 | 555.5541057 | | 87 | rs1595973 | -0.01002 | 0.5595 | 0.4459 | MALEPD | Years of schooling || id:ieu-a-1239 | 0.000753706 | 578.0331735 | | 88 | rs1618725 | 0.01477 | 0.5204 | 0.0617 | MALEPD | Years of schooling || id:ieu-a-1239 | 0.000749378 | 574.7111438 | | 89 | rs1620977 | -0.02046 | 0.6905 | 0.3899 | MALEPD | Years of schooling || id:ieu-a-1239 | 0.00066873 | 512.8191745 | | 90 | rs1671770 | -0.01342 | 0.8061 | 0.3444 | MALEPD | Years of schooling || id:ieu-a-1239 | 0.000584813 | 448.429323 | | 91 | rs16846463 | -0.02256 | 0.1173 | 0.0133 | MALEPD | Years of schooling || id:ieu-a-1239 | 0.000460881 | 353.3559683 | | 92 | rs16854920 | 0.01007 | 0.3554 | 0.5124 | MALEPD | Years of schooling || id:ieu-a-1239 | 0.000720417 | 552.484746 | | 93 | rs1689510 | 0.01761 | 0.3435 | 0.5851 | MALEPD | Years of schooling || id:ieu-a-1239 | 0.000724417 | 555.5541057 | | 94 | rs16995054 | -0.0139 | 0.2007 | 0.1663 | MALEPD | Years of schooling || id:ieu-a-1239 | 0.00062696 | 480.7679761 | | 95 | rs17048855 | 0.01184 | 0.3248 | 0.3994 | MALEPD | Years of schooling || id:ieu-a-1239 | 0.000728461 | 558.6577599 | | 96 | rs17110109 | 0.01023 | 0.3776 | 0.1220 | MALEPD | Years of schooling || id:ieu-a-1239 | 0.000745099 | 571.4270801 | | 97 | rs17126938 | 0.01536 | 0.1207 | 0.4698 | MALEPD | Years of schooling || id:ieu-a-1239 | 0.000521686 | 399.9989561 | | 98 | rs17425572 | -0.01224 | 0.5425 | 0.3868 | MALEPD | Years of schooling || id:ieu-a-1239 | 0.000766997 | 588.2337589 | | 99 | rs17489649 | -0.0139 | 0.3265 | 0.1914 | MALEPD | Years of schooling || id:ieu-a-1239 | 0.000720417 | 552.484746 | | 100 | rs175325 | -0.01179 | 0.5816 | 0.8721 | MALEPD | Years of schooling || id:ieu-a-1239 | 0.000749378 | 574.7111438 | | 101 | rs17551064 | -0.01493 | 0.1599 | 0.5662 | MALEPD | Years of schooling || id:ieu-a-1239 | 0.000567024 | 434.781474 | | 102 | rs17563464 | -0.01477 | 0.2041 | 0.9250 | MALEPD | Years of schooling || id:ieu-a-1239 | 0.000615138 | 471.6968822 | | 103 | rs17565975 | -0.01142 | 0.5306 | 0.8617 | MALEPD | Years of schooling || id:ieu-a-1239 | 0.000762515 | 584.7937954 | | 104 | rs17598675 | 0.01199 | 0.5187 | 0.6288 | MALEPD | Years of schooling || id:ieu-a-1239 | 0.000766997 | 588.2337589 | | 105 | rs176218 | 0.01883 | 0.2007 | 0.7208 | MALEPD | Years of schooling || id:ieu-a-1239 | 0.00060656 | 465.1150652 | | 106 | rs1827540 | -0.0106 | 0.4660 | 0.2054 | MALEPD | Years of schooling || id:ieu-a-1239 | 0.000766997 | 588.2337589 | | 107 | rs1866823 | 0.01009 | 0.5510 | 0.7299 | MALEPD | Years of schooling || id:ieu-a-1239 | 0.000762515 | 584.7937954 | | 108 | rs1882273 | -0.01231 | 0.3469 | 0.2772 | MALEPD | Years of schooling || id:ieu-a-1239 | 0.000720417 | 552.484746 | | 109 | rs192436652 | -0.03497 | 0.0221 | 0.5276 | MALEPD | Years of schooling || id:ieu-a-1239 | 0.000239373 | 183.4857597 | | 110 | rs1925576 | 0.00997 | 0.4422 | 0.9682 | MALEPD | Years of schooling || id:ieu-a-1239 | 0.000762515 | 584.7937954 | | 111 | rs1947114 | 0.01071 | 0.2551 | 0.7192 | MALEPD | Years of schooling || id:ieu-a-1239 | 0.000679171 | 520.8319741 | | 112 | rs1949226 | -0.00965 | 0.5901 | 0.1735 | MALEPD | Years of schooling || id:ieu-a-1239 | 0.000749378 | 574.7111438 | | 113 | rs1964927 | -0.01423 | 0.6378 | 0.9422 | MALEPD | Years of schooling || id:ieu-a-1239 | 0.000736686 | 564.970277 | | 114 | rs2052285 | 0.01123 | 0.5765 | 0.1583 | MALEPD | Years of schooling || id:ieu-a-1239 | 0.000745099 | 571.4270801 | | 115 | rs2067854 | 0.01477 | 0.1820 | 0.2428 | MALEPD | Years of schooling || id:ieu-a-1239 | 0.000623962 | 478.4676508 | | 116 | rs2179152 | 0.01455 | 0.6429 | 0.2487 | MALEPD | Years of schooling || id:ieu-a-1239 | 0.000740868 | 568.1803353 | | 117 | rs2182505 | -0.01086 | 0.7364 | 0.1927 | MALEPD | Years of schooling || id:ieu-a-1239 | 0.000679171 | 520.8319741 | | 118 | rs225291 | -0.01205 | 0.8027 | 0.2282 | MALEPD | Years of schooling || id:ieu-a-1239 | 0.000609393 | 467.2885001 | | 119 | rs2256965 | -0.01128 | 0.5425 | 0.6002 | MALEPD | Years of schooling || id:ieu-a-1239 | 0.000740868 | 568.1803353 | | 120 | rs2283076 | -0.01143 | 0.2143 | 0.3725 | MALEPD | Years of schooling || id:ieu-a-1239 | 0.000639246 | 490.1947991 | | 121 | rs2287838 | -0.01152 | 0.5340 | 0.0268 | MALEPD | Years of schooling || id:ieu-a-1239 | 0.000762515 | 584.7937954 | | 122 | rs2302761 | 0.01354 | 0.1905 | 0.0565 | MALEPD | Years of schooling || id:ieu-a-1239 | 0.000623962 | 478.4676508 | | 123 | rs2347526 | 0.01395 | 0.6378 | 0.5296 | MALEPD | Years of schooling || id:ieu-a-1239 | 0.000728461 | 558.6577599 | | 124 | rs2414072 | -0.01005 | 0.4864 | 0.0916 | MALEPD | Years of schooling || id:ieu-a-1239 | 0.000762515 | 584.7937954 | | 125 | rs242093 | -0.01031 | 0.5476 | 0.2265 | MALEPD | Years of schooling || id:ieu-a-1239 | 0.000758085 | 581.3938315 | | 126 | rs2441111 | 0.01087 | 0.5323 | 0.3882 | MALEPD | Years of schooling || id:ieu-a-1239 | 0.000766997 | 588.2337589 | | 127 | rs2447535 | 0.01181 | 0.7245 | 0.5428 | MALEPD | Years of schooling || id:ieu-a-1239 | 0.000704852 | 540.5391298 | | 128 | rs2478208 | -0.0106 | 0.5000 | 0.8397 | MALEPD | Years of schooling || id:ieu-a-1239 | 0.000766997 | 588.2337589 | | 129 | rs2545798 | -0.01346 | 0.4949 | 0.3793 | MALEPD | Years of schooling || id:ieu-a-1239 | 0.000762515 | 584.7937954 | | 130 | rs2554835 | 0.00974 | 0.3980 | 0.3684 | MALEPD | Years of schooling || id:ieu-a-1239 | 0.000745099 | 571.4270801 | | 131 | rs2570497 | -0.01233 | 0.6735 | 0.4170 | MALEPD | Years of schooling || id:ieu-a-1239 | 0.000736686 | 564.970277 | | 132 | rs2725370 | 0.01536 | 0.7109 | 0.6289 | MALEPD | Years of schooling || id:ieu-a-1239 | 0.000697318 | 534.7579627 | | 133 | rs277828 | -0.01091 | 0.2568 | 0.1819 | MALEPD | Years of schooling || id:ieu-a-1239 | 0.00066532 | 510.2027501 | | 134 | rs2787101 | 0.00968 | 0.6173 | 0.1012 | MALEPD | Years of schooling || id:ieu-a-1239 | 0.000749378 | 574.7111438 | | 135 | rs2819336 | -0.01828 | 0.6616 | 0.0183 | MALEPD | Years of schooling || id:ieu-a-1239 | 0.000736686 | 564.970277 | | 136 | rs2820314 | -0.011 | 0.3163 | 0.0601 | MALEPD | Years of schooling || id:ieu-a-1239 | 0.000724417 | 555.5541057 | | 137 | rs28373063 | 0.01389 | 0.2075 | 0.5186 | MALEPD | Years of schooling || id:ieu-a-1239 | 0.000569499 | 436.6800831 | | 138 | rs28513670 | 0.01477 | 0.1531 | 0.7673 | MALEPD | Years of schooling || id:ieu-a-1239 | 0.000579617 | 444.4432845 | | 139 | rs2885198 | -0.01025 | 0.5017 | 0.6511 | MALEPD | Years of schooling || id:ieu-a-1239 | 0.000766997 | 588.2337589 | | 140 | rs2901616 | 0.00941 | 0.5170 | 0.2349 | MALEPD | Years of schooling || id:ieu-a-1239 | 0.000762515 | 584.7937954 | | 141 | rs2905426 | 0.01037 | 0.6446 | 0.6183 | MALEPD | Years of schooling || id:ieu-a-1239 | 0.000720417 | 552.484746 | | 142 | rs2923431 | 0.0114 | 0.6446 | 0.1126 | MALEPD | Years of schooling || id:ieu-a-1239 | 0.000740868 | 568.1803353 | | 143 | rs2971970 | 0.01654 | 0.7721 | 0.8207 | MALEPD | Years of schooling || id:ieu-a-1239 | 0.000629987 | 483.0905267 | | 144 | rs2998315 | 0.01269 | 0.5782 | 0.9795 | MALEPD | Years of schooling || id:ieu-a-1239 | 0.000762515 | 584.7937954 | | 145 | rs3013014 | -0.01024 | 0.6105 | 0.4729 | MALEPD | Years of schooling || id:ieu-a-1239 | 0.000758085 | 581.3938315 | | 146 | rs301800 | -0.01516 | 0.8197 | 0.1570 | MALEPD | Years of schooling || id:ieu-a-1239 | 0.000582203 | 446.4274063 | | 147 | rs3026996 | -0.01537 | 0.2840 | 0.1938 | MALEPD | Years of schooling || id:ieu-a-1239 | 0.000655297 | 502.5112514 | | 148 | rs31940 | 0.01548 | 0.1344 | 0.8092 | MALEPD | Years of schooling || id:ieu-a-1239 | 0.000530164 | 406.5030042 | | 149 | rs320693 | 0.01204 | 0.4728 | 0.9211 | MALEPD | Years of schooling || id:ieu-a-1239 | 0.000766997 | 588.2337589 | | 150 | rs337637 | 0.01123 | 0.3367 | 0.1369 | MALEPD | Years of schooling || id:ieu-a-1239 | 0.000736686 | 564.970277 | | 151 | rs34316 | -0.02016 | 0.5799 | 0.7733 | MALEPD | Years of schooling || id:ieu-a-1239 | 0.000736686 | 564.970277 | | 152 | rs34394051 | 0.01392 | 0.1599 | 0.2591 | MALEPD | Years of schooling || id:ieu-a-1239 | 0.000543411 | 416.6655793 | | 153 | rs34485537 | 0.01075 | 0.3895 | 0.7188 | MALEPD | Years of schooling || id:ieu-a-1239 | 0.000753706 | 578.0331735 | | 154 | rs34853711 | -0.01596 | 0.2466 | 0.5626 | MALEPD | Years of schooling || id:ieu-a-1239 | 0.000633043 | 485.4356263 | | 155 | rs35039375 | -0.01983 | 0.0935 | 0.8442 | MALEPD | Years of schooling || id:ieu-a-1239 | 0.000445159 | 341.2960376 | | 156 | rs35309068 | 0.01321 | 0.4660 | 0.7564 | MALEPD | Years of schooling || id:ieu-a-1239 | 0.000762515 | 584.7937954 | | 157 | rs35316276 | 0.01173 | 0.2942 | 0.5979 | MALEPD | Years of schooling || id:ieu-a-1239 | 0.000672174 | 515.4625723 | | 158 | rs35417702 | -0.01445 | 0.5765 | 0.3963 | MALEPD | Years of schooling || id:ieu-a-1239 | 0.000766997 | 588.2337589 | | 159 | rs35475880 | -0.01511 | 0.1837 | 0.0925 | MALEPD | Years of schooling || id:ieu-a-1239 | 0.00062696 | 480.7679761 | | 160 | rs35532491 | 0.02007 | 0.1071 | 0.3417 | MALEPD | Years of schooling || id:ieu-a-1239 | 0.000456049 | 349.6494371 | | 161 | rs36083520 | 0.01629 | 0.1667 | 0.0409 | MALEPD | Years of schooling || id:ieu-a-1239 | 0.000584813 | 448.429323 | | 162 | rs36119825 | 0.01063 | 0.4694 | 0.7065 | MALEPD | Years of schooling || id:ieu-a-1239 | 0.000762515 | 584.7937954 | | 163 | rs363096 | 0.01363 | 0.5748 | 0.2663 | MALEPD | Years of schooling || id:ieu-a-1239 | 0.000758085 | 581.3938315 | | 164 | rs3747631 | 0.02207 | 0.2279 | 0.0026 | MALEPD | Years of schooling || id:ieu-a-1239 | 0.00062696 | 480.7679761 | | 165 | rs3788556 | -0.01138 | 0.5408 | 0.8177 | MALEPD | Years of schooling || id:ieu-a-1239 | 0.000762515 | 584.7937954 | | 166 | rs3800546 | -0.01183 | 0.2704 | 0.6164 | MALEPD | Years of schooling || id:ieu-a-1239 | 0.000672174 | 515.4625723 | | 167 | rs3809634 | 0.01058 | 0.3350 | 0.2708 | MALEPD | Years of schooling || id:ieu-a-1239 | 0.000704852 | 540.5391298 | | 168 | rs3890802 | -0.01133 | 0.2687 | 0.9875 | MALEPD | Years of schooling || id:ieu-a-1239 | 0.000682725 | 523.558843 | | 169 | rs3897821 | -0.01502 | 0.3503 | 0.0037 | MALEPD | Years of schooling || id:ieu-a-1239 | 0.000724417 | 555.5541057 | | 170 | rs401687 | 0.01144 | 0.4558 | 0.1637 | MALEPD | Years of schooling || id:ieu-a-1239 | 0.000766997 | 588.2337589 | | 171 | rs406413 | -0.01695 | 0.2109 | 0.1591 | MALEPD | Years of schooling || id:ieu-a-1239 | 0.000623962 | 478.4676508 | | 172 | rs4073894 | 0.01524 | 0.1769 | 0.9135 | MALEPD | Years of schooling || id:ieu-a-1239 | 0.000618052 | 473.9324124 | | 173 | rs4328757 | 0.01067 | 0.6514 | 0.5371 | MALEPD | Years of schooling || id:ieu-a-1239 | 0.000749378 | 574.7111438 | | 174 | rs4352658 | -0.0212 | 0.0901 | 0.2153 | MALEPD | Years of schooling || id:ieu-a-1239 | 0.000423488 | 324.6744773 | | 175 | rs4369924 | 0.01362 | 0.1684 | 0.8960 | MALEPD | Years of schooling || id:ieu-a-1239 | 0.000557337 | 427.3493121 | | 176 | rs4382592 | 0.01636 | 0.6990 | 0.8408 | MALEPD | Years of schooling || id:ieu-a-1239 | 0.000704852 | 540.5391298 | | 177 | rs4384309 | 0.0109 | 0.4796 | 0.8262 | MALEPD | Years of schooling || id:ieu-a-1239 | 0.000758085 | 581.3938315 | | 178 | rs4392737 | -0.0097 | 0.3827 | 0.6180 | MALEPD | Years of schooling || id:ieu-a-1239 | 0.000753706 | 578.0331735 | | 179 | rs4442732 | -0.01063 | 0.5969 | 0.4025 | MALEPD | Years of schooling || id:ieu-a-1239 | 0.000740868 | 568.1803353 | | 180 | rs4497562 | -0.01204 | 0.2823 | 0.0606 | MALEPD | Years of schooling || id:ieu-a-1239 | 0.000679171 | 520.8319741 | | 181 | rs4667025 | 0.00957 | 0.3878 | 0.5172 | MALEPD | Years of schooling || id:ieu-a-1239 | 0.000749378 | 574.7111438 | | 182 | rs4700393 | 0.02086 | 0.5289 | 0.7780 | MALEPD | Years of schooling || id:ieu-a-1239 | 0.000766997 | 588.2337589 | | 183 | rs4726070 | 0.01251 | 0.6207 | 0.8741 | MALEPD | Years of schooling || id:ieu-a-1239 | 0.000749378 | 574.7111438 | | 184 | rs4733264 | -0.00954 | 0.6310 | 0.8139 | MALEPD | Years of schooling || id:ieu-a-1239 | 0.000749378 | 574.7111438 | | 185 | rs4743923 | 0.00985 | 0.3673 | 0.0896 | MALEPD | Years of schooling || id:ieu-a-1239 | 0.000740868 | 568.1803353 | | 186 | rs4757957 | 0.0141 | 0.6514 | 0.7341 | MALEPD | Years of schooling || id:ieu-a-1239 | 0.00070868 | 543.4768425 | | 187 | rs4766424 | -0.0141 | 0.9116 | 0.5462 | MALEPD | Years of schooling || id:ieu-a-1239 | 0.000505518 | 387.5958877 | | 188 | rs4778058 | 0.01017 | 0.5221 | 0.1260 | MALEPD | Years of schooling || id:ieu-a-1239 | 0.000766997 | 588.2337589 | | 189 | rs4787457 | -0.01741 | 0.3146 | 0.4132 | MALEPD | Years of schooling || id:ieu-a-1239 | 0.000740868 | 568.1803353 | | 190 | rs4810227 | 0.01272 | 0.6344 | 0.7226 | MALEPD | Years of schooling || id:ieu-a-1239 | 0.000745099 | 571.4270801 | | 191 | rs4839155 | -0.01251 | 0.2500 | 0.4232 | MALEPD | Years of schooling || id:ieu-a-1239 | 0.000652022 | 499.9986951 | | 192 | rs4846724 | 0.01018 | 0.4915 | 0.1225 | MALEPD | Years of schooling || id:ieu-a-1239 | 0.000766997 | 588.2337589 | | 193 | rs4870482 | -0.01083 | 0.2585 | 0.9754 | MALEPD | Years of schooling || id:ieu-a-1239 | 0.000686316 | 526.3144159 | | 194 | rs4888746 | -0.00952 | 0.3776 | 0.3606 | MALEPD | Years of schooling || id:ieu-a-1239 | 0.000749378 | 574.7111438 | | 195 | rs4904523 | -0.00936 | 0.4592 | 0.5621 | MALEPD | Years of schooling || id:ieu-a-1239 | 0.000766997 | 588.2337589 | | 196 | rs4945424 | -0.00992 | 0.4150 | 0.8932 | MALEPD | Years of schooling || id:ieu-a-1239 | 0.000762515 | 584.7937954 | | 197 | rs4964046 | 0.01053 | 0.3350 | 0.0233 | MALEPD | Years of schooling || id:ieu-a-1239 | 0.00073255 | 561.7962866 | | 198 | rs4972400 | 0.01156 | 0.3520 | 0.5570 | MALEPD | Years of schooling || id:ieu-a-1239 | 0.000720417 | 552.484746 | | 199 | rs4984541 | 0.01233 | 0.2415 | 0.4761 | MALEPD | Years of schooling || id:ieu-a-1239 | 0.000629987 | 483.0905267 | | 200 | rs535307 | -0.01004 | 0.6769 | 0.1911 | MALEPD | Years of schooling || id:ieu-a-1239 | 0.00070868 | 543.4768425 | | 201 | rs55736314 | 0.01431 | 0.4167 | 0.0137 | MALEPD | Years of schooling || id:ieu-a-1239 | 0.000749378 | 574.7111438 | | 202 | rs55771711 | 0.01555 | 0.2279 | 0.3389 | MALEPD | Years of schooling || id:ieu-a-1239 | 0.000655297 | 502.5112514 | | 203 | rs56391344 | 0.01571 | 0.2381 | 0.5195 | MALEPD | Years of schooling || id:ieu-a-1239 | 0.000661945 | 507.6128884 | | 204 | rs575113 | 0.01285 | 0.2772 | 0.6186 | MALEPD | Years of schooling || id:ieu-a-1239 | 0.000701065 | 537.6330055 | | 205 | rs59123361 | -0.02094 | 0.1054 | 0.2283 | MALEPD | Years of schooling || id:ieu-a-1239 | 0.000448217 | 343.6417148 | | 206 | rs59480703 | -0.01237 | 0.1633 | 0.4300 | MALEPD | Years of schooling || id:ieu-a-1239 | 0.00060656 | 465.1150652 | | 207 | rs60483752 | 0.01078 | 0.5816 | 0.3945 | MALEPD | Years of schooling || id:ieu-a-1239 | 0.000758085 | 581.3938315 | | 208 | rs6122735 | 0.0105 | 0.4133 | 0.6316 | MALEPD | Years of schooling || id:ieu-a-1239 | 0.000749378 | 574.7111438 | | 209 | rs6123924 | -0.01528 | 0.1599 | 0.7013 | MALEPD | Years of schooling || id:ieu-a-1239 | 0.000554966 | 425.5308043 | | 210 | rs613872 | -0.0175 | 0.8282 | 0.6745 | MALEPD | Years of schooling || id:ieu-a-1239 | 0.000574513 | 440.5274847 | | 211 | rs61747885 | 0.01383 | 0.1412 | 0.3722 | MALEPD | Years of schooling || id:ieu-a-1239 | 0.000554966 | 425.5308043 | | 212 | rs62097985 | -0.01288 | 0.4116 | 0.5172 | MALEPD | Years of schooling || id:ieu-a-1239 | 0.000758085 | 581.3938315 | | 213 | rs62157915 | 0.02091 | 0.0595 | 0.1584 | MALEPD | Years of schooling || id:ieu-a-1239 | 0.000374829 | 287.3555719 | | 214 | rs62183776 | -0.01308 | 0.1905 | 0.1161 | MALEPD | Years of schooling || id:ieu-a-1239 | 0.000600973 | 460.8282904 | | 215 | rs62184480 | -0.01528 | 0.2449 | 0.6283 | MALEPD | Years of schooling || id:ieu-a-1239 | 0.000682725 | 523.558843 | | 216 | rs62439690 | -0.01087 | 0.2670 | 0.8404 | MALEPD | Years of schooling || id:ieu-a-1239 | 0.000672174 | 515.4625723 | | 217 | rs62444881 | 0.01815 | 0.1905 | 0.6699 | MALEPD | Years of schooling || id:ieu-a-1239 | 0.000600973 | 460.8282904 | | 218 | rs6493265 | -0.01385 | 0.3895 | 0.0357 | MALEPD | Years of schooling || id:ieu-a-1239 | 0.000749378 | 574.7111438 | | 219 | rs6513959 | -0.01177 | 0.2789 | 0.4900 | MALEPD | Years of schooling || id:ieu-a-1239 | 0.000704852 | 540.5391298 | | 220 | rs6557171 | 0.01567 | 0.7245 | 0.5348 | MALEPD | Years of schooling || id:ieu-a-1239 | 0.000720417 | 552.484746 | | 221 | rs663234 | -0.01005 | 0.6122 | 0.8191 | MALEPD | Years of schooling || id:ieu-a-1239 | 0.000749378 | 574.7111438 | | 222 | rs66568921 | 0.01565 | 0.3639 | 0.2682 | MALEPD | Years of schooling || id:ieu-a-1239 | 0.000716462 | 549.4491155 | | 223 | rs6731373 | -0.01256 | 0.3367 | 0.0936 | MALEPD | Years of schooling || id:ieu-a-1239 | 0.000720417 | 552.484746 | | 224 | rs6731967 | -0.01186 | 0.2092 | 0.3129 | MALEPD | Years of schooling || id:ieu-a-1239 | 0.000655297 | 502.5112514 | | 225 | rs67885444 | 0.01406 | 0.1718 | 0.1024 | MALEPD | Years of schooling || id:ieu-a-1239 | 0.000562139 | 431.0333578 | | 226 | rs67890737 | -0.01141 | 0.3265 | 0.1064 | MALEPD | Years of schooling || id:ieu-a-1239 | 0.000728461 | 558.6577599 | | 227 | rs6803651 | 0.01131 | 0.4150 | 0.7692 | MALEPD | Years of schooling || id:ieu-a-1239 | 0.000758085 | 581.3938315 | | 228 | rs6805241 | -0.01413 | 0.1973 | 0.2640 | MALEPD | Years of schooling || id:ieu-a-1239 | 0.000642393 | 492.6095518 | | 229 | rs6867851 | -0.012 | 0.4235 | 0.7075 | MALEPD | Years of schooling || id:ieu-a-1239 | 0.000753706 | 578.0331735 | | 230 | rs6938002 | -0.01008 | 0.3963 | 0.7869 | MALEPD | Years of schooling || id:ieu-a-1239 | 0.000753706 | 578.0331735 | | 231 | rs6959891 | -0.01136 | 0.2959 | 0.4689 | MALEPD | Years of schooling || id:ieu-a-1239 | 0.000689944 | 529.0991483 | | 232 | rs7012546 | 0.01009 | 0.4201 | 0.5884 | MALEPD | Years of schooling || id:ieu-a-1239 | 0.000758085 | 581.3938315 | | 233 | rs7016302 | 0.01243 | 0.1769 | 0.6773 | MALEPD | Years of schooling || id:ieu-a-1239 | 0.000571995 | 438.5953466 | | 234 | rs702606 | -0.01427 | 0.1701 | 0.9020 | MALEPD | Years of schooling || id:ieu-a-1239 | 0.000521686 | 399.9989561 | | 235 | rs7029718 | 0.02439 | 0.4354 | 0.7682 | MALEPD | Years of schooling || id:ieu-a-1239 | 0.000749378 | 574.7111438 | | 236 | rs7031698 | 0.01248 | 0.7755 | 0.0653 | MALEPD | Years of schooling || id:ieu-a-1239 | 0.000633043 | 485.4356263 | | 237 | rs710629 | 0.01053 | 0.6565 | 0.2091 | MALEPD | Years of schooling || id:ieu-a-1239 | 0.000736686 | 564.970277 | | 238 | rs71646142 | 0.01286 | 0.1735 | 0.0963 | MALEPD | Years of schooling || id:ieu-a-1239 | 0.000600973 | 460.8282904 | | 239 | rs7233920 | -0.01315 | 0.2160 | 0.5424 | MALEPD | Years of schooling || id:ieu-a-1239 | 0.000645571 | 495.048213 | | 240 | rs7257460 | -0.01145 | 0.2704 | 0.2535 | MALEPD | Years of schooling || id:ieu-a-1239 | 0.000689944 | 529.0991483 | | 241 | rs7278859 | 0.01013 | 0.3078 | 0.3525 | MALEPD | Years of schooling || id:ieu-a-1239 | 0.000704852 | 540.5391298 | | 242 | rs72807818 | 0.01915 | 0.1241 | 0.2290 | MALEPD | Years of schooling || id:ieu-a-1239 | 0.000517548 | 396.8243612 | | 243 | rs72828517 | 0.01836 | 0.1412 | 0.9782 | MALEPD | Years of schooling || id:ieu-a-1239 | 0.000582203 | 446.4274063 | | 244 | rs72840994 | 0.01247 | 0.1820 | 0.3760 | MALEPD | Years of schooling || id:ieu-a-1239 | 0.000603753 | 462.9617547 | | 245 | rs730384 | 0.01016 | 0.4558 | 0.9736 | MALEPD | Years of schooling || id:ieu-a-1239 | 0.000762515 | 584.7937954 | | 246 | rs7315713 | -0.01022 | 0.6633 | 0.4269 | MALEPD | Years of schooling || id:ieu-a-1239 | 0.000697318 | 534.7579627 | | 247 | rs7321274 | -0.01275 | 0.1956 | 0.1576 | MALEPD | Years of schooling || id:ieu-a-1239 | 0.000618052 | 473.9324124 | | 248 | rs73301698 | -0.01291 | 0.2262 | 0.4361 | MALEPD | Years of schooling || id:ieu-a-1239 | 0.00062696 | 480.7679761 | | 249 | rs7332724 | -0.01149 | 0.2619 | 0.8402 | MALEPD | Years of schooling || id:ieu-a-1239 | 0.000689944 | 529.0991483 | | 250 | rs73344830 | -0.0172 | 0.6020 | 0.9102 | MALEPD | Years of schooling || id:ieu-a-1239 | 0.000758085 | 581.3938315 | | 251 | rs736282 | -0.01082 | 0.5153 | 0.4729 | MALEPD | Years of schooling || id:ieu-a-1239 | 0.000766997 | 588.2337589 | | 252 | rs73874335 | -0.0199 | 0.0595 | 0.6670 | MALEPD | Years of schooling || id:ieu-a-1239 | 0.000361336 | 277.0075873 | | 253 | rs743316 | -0.01185 | 0.1820 | 0.2579 | MALEPD | Years of schooling || id:ieu-a-1239 | 0.00062696 | 480.7679761 | | 254 | rs74643044 | 0.02323 | 0.0272 | 0.1014 | MALEPD | Years of schooling || id:ieu-a-1239 | 0.000337942 | 259.0666814 | | 255 | rs74701752 | 0.01591 | 0.0952 | 0.1089 | MALEPD | Years of schooling || id:ieu-a-1239 | 0.000457648 | 350.8762773 | | 256 | rs7481514 | 0.01072 | 0.6650 | 0.9611 | MALEPD | Years of schooling || id:ieu-a-1239 | 0.00073255 | 561.7962866 | | 257 | rs74998289 | -0.01821 | 0.2398 | 0.0000 | MALEPD | Years of schooling || id:ieu-a-1239 | 0.000612252 | 469.4823428 | | 258 | rs7594904 | 0.00969 | 0.4184 | 0.7979 | MALEPD | Years of schooling || id:ieu-a-1239 | 0.000753706 | 578.0331735 | | 259 | rs7603132 | 0.01317 | 0.1548 | 0.0632 | MALEPD | Years of schooling || id:ieu-a-1239 | 0.00060656 | 465.1150652 | | 260 | rs76076331 | 0.01873 | 0.1310 | 0.3813 | MALEPD | Years of schooling || id:ieu-a-1239 | 0.000525891 | 403.2247541 | | 261 | rs7650602 | 0.00939 | 0.4286 | 0.6051 | MALEPD | Years of schooling || id:ieu-a-1239 | 0.000762515 | 584.7937954 | | 262 | rs76608582 | 0.02798 | 0.0408 | 0.6501 | MALEPD | Years of schooling || id:ieu-a-1239 | 0.000293149 | 224.7185147 | | 263 | rs76878669 | -0.01399 | 0.2534 | 0.4010 | MALEPD | Years of schooling || id:ieu-a-1239 | 0.000636129 | 487.803605 | | 264 | rs77025239 | -0.01422 | 0.1088 | 0.2032 | MALEPD | Years of schooling || id:ieu-a-1239 | 0.000557337 | 427.3493121 | | 265 | rs77128898 | -0.02769 | 0.0221 | 0.8310 | MALEPD | Years of schooling || id:ieu-a-1239 | 0.000270652 | 207.4683382 | | 266 | rs77702622 | -0.02447 | 0.0765 | 0.4781 | MALEPD | Years of schooling || id:ieu-a-1239 | 0.000371627 | 284.8995414 | | 267 | rs77719387 | -0.04597 | 0.0170 | 0.3222 | MALEPD | Years of schooling || id:ieu-a-1239 | 0.000179705 | 137.7406874 | | 268 | rs77835879 | -0.01601 | 0.0901 | 0.8903 | MALEPD | Years of schooling || id:ieu-a-1239 | 0.000452883 | 347.221316 | | 269 | rs7796203 | -0.01074 | 0.5255 | 0.2869 | MALEPD | Years of schooling || id:ieu-a-1239 | 0.000762515 | 584.7937954 | | 270 | rs7803932 | 0.0143 | 0.1565 | 0.0296 | MALEPD | Years of schooling || id:ieu-a-1239 | 0.000577054 | 442.4767213 | | 271 | rs7808399 | 0.0107 | 0.5476 | 0.6222 | MALEPD | Years of schooling || id:ieu-a-1239 | 0.000762515 | 584.7937954 | | 272 | rs7833201 | -0.01532 | 0.1344 | 0.0846 | MALEPD | Years of schooling || id:ieu-a-1239 | 0.000497804 | 381.6783932 | | 273 | rs7863447 | 0.01678 | 0.8333 | 0.3634 | MALEPD | Years of schooling || id:ieu-a-1239 | 0.000559727 | 429.1834293 | | 274 | rs78721320 | 0.01307 | 0.2041 | 0.7589 | MALEPD | Years of schooling || id:ieu-a-1239 | 0.000595488 | 456.6198129 | | 275 | rs790647 | -0.01482 | 0.2347 | 0.5606 | MALEPD | Years of schooling || id:ieu-a-1239 | 0.000645571 | 495.048213 | | 276 | rs7920624 | -0.01181 | 0.4932 | 0.2374 | MALEPD | Years of schooling || id:ieu-a-1239 | 0.000766997 | 588.2337589 | | 277 | rs7924036 | 0.01501 | 0.5391 | 0.4588 | MALEPD | Years of schooling || id:ieu-a-1239 | 0.000766997 | 588.2337589 | | 278 | rs79265434 | 0.02331 | 0.1173 | 0.0438 | MALEPD | Years of schooling || id:ieu-a-1239 | 0.000497804 | 381.6783932 | | 279 | rs79269403 | 0.01447 | 0.2228 | 0.6099 | MALEPD | Years of schooling || id:ieu-a-1239 | 0.000639246 | 490.1947991 | | 280 | rs7928622 | 0.01011 | 0.3078 | 0.6450 | MALEPD | Years of schooling || id:ieu-a-1239 | 0.000720417 | 552.484746 | | 281 | rs795230 | 0.00952 | 0.4184 | 0.9968 | MALEPD | Years of schooling || id:ieu-a-1239 | 0.000758085 | 581.3938315 | | 282 | rs79523955 | -0.01802 | 0.0952 | 0.8927 | MALEPD | Years of schooling || id:ieu-a-1239 | 0.000460881 | 353.3559683 | | 283 | rs7977614 | 0.01325 | 0.3078 | 0.0783 | MALEPD | Years of schooling || id:ieu-a-1239 | 0.000658604 | 505.049187 | | 284 | rs7993663 | 0.0118 | 0.3571 | 0.3338 | MALEPD | Years of schooling || id:ieu-a-1239 | 0.00073255 | 561.7962866 | | 285 | rs8008382 | 0.01208 | 0.6871 | 0.7015 | MALEPD | Years of schooling || id:ieu-a-1239 | 0.000704852 | 540.5391298 | | 286 | rs80171383 | 0.0145 | 0.1241 | 0.5727 | MALEPD | Years of schooling || id:ieu-a-1239 | 0.000541157 | 414.9366764 | | 287 | rs8020034 | 0.01782 | 0.2058 | 0.6435 | MALEPD | Years of schooling || id:ieu-a-1239 | 0.000584813 | 448.429323 | | 288 | rs818415 | 0.01235 | 0.1820 | 0.2580 | MALEPD | Years of schooling || id:ieu-a-1239 | 0.000595488 | 456.6198129 | | 289 | rs837080 | 0.01092 | 0.4932 | 0.0001 | MALEPD | Years of schooling || id:ieu-a-1239 | 0.000766997 | 588.2337589 | | 290 | rs892612 | 0.01464 | 0.8418 | 0.9938 | MALEPD | Years of schooling || id:ieu-a-1239 | 0.000550286 | 421.9398271 | | 291 | rs894067 | 0.01041 | 0.3929 | 0.6335 | MALEPD | Years of schooling || id:ieu-a-1239 | 0.000745099 | 571.4270801 | | 292 | rs9289300 | 0.01512 | 0.1837 | 0.5752 | MALEPD | Years of schooling || id:ieu-a-1239 | 0.000557337 | 427.3493121 | | 293 | rs9320493 | -0.01394 | 0.8639 | 0.6172 | MALEPD | Years of schooling || id:ieu-a-1239 | 0.000543411 | 416.6655793 | | 294 | rs9342482 | 0.01264 | 0.2908 | 0.8916 | MALEPD | Years of schooling || id:ieu-a-1239 | 0.000661945 | 507.6128884 | | 295 | rs9349956 | 0.01881 | 0.2398 | 0.8938 | MALEPD | Years of schooling || id:ieu-a-1239 | 0.000579617 | 444.4432845 | | 296 | rs9372625 | 0.02383 | 0.4133 | 0.3624 | MALEPD | Years of schooling || id:ieu-a-1239 | 0.000740868 | 568.1803353 | | 297 | rs9384679 | -0.00959 | 0.4082 | 0.0463 | MALEPD | Years of schooling || id:ieu-a-1239 | 0.000740868 | 568.1803353 | | 298 | rs9386319 | 0.00991 | 0.4269 | 0.9127 | MALEPD | Years of schooling || id:ieu-a-1239 | 0.000749378 | 574.7111438 | | 299 | rs9386787 | 0.00958 | 0.5136 | 0.8626 | MALEPD | Years of schooling || id:ieu-a-1239 | 0.000766997 | 588.2337589 | | 300 | rs9436866 | 0.01882 | 0.0952 | 0.4310 | MALEPD | Years of schooling || id:ieu-a-1239 | 0.000451317 | 346.0198582 | | 301 | rs9503598 | 0.01079 | 0.4388 | 0.2117 | MALEPD | Years of schooling || id:ieu-a-1239 | 0.000762515 | 584.7937954 | | 302 | rs9529119 | -0.01295 | 0.8027 | 0.2104 | MALEPD | Years of schooling || id:ieu-a-1239 | 0.000639246 | 490.1947991 | | 303 | rs9556958 | -0.0108 | 0.5289 | 0.7236 | MALEPD | Years of schooling || id:ieu-a-1239 | 0.000766997 | 588.2337589 | | 304 | rs9616906 | 0.01497 | 0.4235 | 0.1559 | MALEPD | Years of schooling || id:ieu-a-1239 | 0.000758085 | 581.3938315 | | 305 | rs9679654 | 0.01042 | 0.4847 | 0.1050 | MALEPD | Years of schooling || id:ieu-a-1239 | 0.000758085 | 581.3938315 | | 306 | rs969512 | 0.01249 | 0.2959 | 0.3410 | MALEPD | Years of schooling || id:ieu-a-1239 | 0.000728461 | 558.6577599 | | 307 | rs9704097 | -0.0103 | 0.4728 | 0.1433 | MALEPD | Years of schooling || id:ieu-a-1239 | 0.000762515 | 584.7937954 | | 308 | rs9882532 | -0.01208 | 0.3639 | 0.6620 | MALEPD | Years of schooling || id:ieu-a-1239 | 0.000736686 | 564.970277 | | 309 | rs9914918 | 0.01155 | 0.2823 | 0.9992 | MALEPD | Years of schooling || id:ieu-a-1239 | 0.000689944 | 529.0991483 | | 310 | rs9933256 | -0.01134 | 0.4082 | 0.7914 | MALEPD | Years of schooling || id:ieu-a-1239 | 0.000758085 | 581.3938315 | | 311 | rs9936270 | -0.0136 | 0.3078 | 0.9868 | MALEPD | Years of schooling || id:ieu-a-1239 | 0.000658604 | 505.049187 | | 312 | rs9938678 | 0.01355 | 0.2432 | 0.0185 | MALEPD | Years of schooling || id:ieu-a-1239 | 0.000636129 | 487.803605 | | 313 | rs9964724 | 0.01978 | 0.6599 | 0.5694 | MALEPD | Years of schooling || id:ieu-a-1239 | 0.000712549 | 546.4466613 | | 314 | rs9995567 | 0.00998 | 0.3827 | 0.7693 | MALEPD | Years of schooling || id:ieu-a-1239 | 0.00073255 | 561.7962866 | | 1 | rs10073890 | -0.01262 | 0.7364 | 0.4070 | PDAOO | Years of schooling || id:ieu-a-1239 | 0.00066532 | 510.2027501 | | 2 | rs1008078 | -0.01738 | 0.4099 | 0.6312 | PDAOO | Years of schooling || id:ieu-a-1239 | 0.000753706 | 578.0331735 | | 3 | rs10189857 | -0.01725 | 0.4184 | 0.5668 | PDAOO | Years of schooling || id:ieu-a-1239 | 0.000762515 | 584.7937954 | | 4 | rs10191758 | 0.01631 | 0.3810 | 0.5898 | PDAOO | Years of schooling || id:ieu-a-1239 | 0.000745099 | 571.4270801 | | 5 | rs10205801 | -0.01053 | 0.5068 | 0.6717 | PDAOO | Years of schooling || id:ieu-a-1239 | 0.000762515 | 584.7937954 | | 6 | rs10215082 | 0.01303 | 0.5612 | 0.0467 | PDAOO | Years of schooling || id:ieu-a-1239 | 0.000758085 | 581.3938315 | | 7 | rs10240905 | 0.01167 | 0.6684 | 0.3166 | PDAOO | Years of schooling || id:ieu-a-1239 | 0.000736686 | 564.970277 | | 8 | rs10456918 | 0.01485 | 0.1820 | 0.4730 | PDAOO | Years of schooling || id:ieu-a-1239 | 0.000582203 | 446.4274063 | | 9 | rs10460095 | -0.01066 | 0.5867 | 0.2501 | PDAOO | Years of schooling || id:ieu-a-1239 | 0.000762515 | 584.7937954 | | 10 | rs1051474 | 0.01301 | 0.2738 | 0.4063 | PDAOO | Years of schooling || id:ieu-a-1239 | 0.000693612 | 531.9135054 | | 11 | rs10760023 | 0.01095 | 0.3316 | 0.7838 | PDAOO | Years of schooling || id:ieu-a-1239 | 0.000712549 | 546.4466613 | | 12 | rs10765775 | 0.01488 | 0.3963 | 0.0655 | PDAOO | Years of schooling || id:ieu-a-1239 | 0.000740868 | 568.1803353 | | 13 | rs10772644 | 0.01614 | 0.8929 | 0.7387 | PDAOO | Years of schooling || id:ieu-a-1239 | 0.000488486 | 374.5308578 | | 14 | rs10773002 | -0.02191 | 0.7211 | 0.7028 | PDAOO | Years of schooling || id:ieu-a-1239 | 0.000661945 | 507.6128884 | | 15 | rs10797055 | 0.00986 | 0.5102 | 0.4181 | PDAOO | Years of schooling || id:ieu-a-1239 | 0.000758085 | 581.3938315 | | 16 | rs10798418 | -0.00957 | 0.4660 | 0.2916 | PDAOO | Years of schooling || id:ieu-a-1239 | 0.000753706 | 578.0331735 | | 17 | rs10856785 | -0.01132 | 0.7296 | 0.3887 | PDAOO | Years of schooling || id:ieu-a-1239 | 0.000679171 | 520.8319741 | | 18 | rs10862376 | 0.01616 | 0.1361 | 0.7859 | PDAOO | Years of schooling || id:ieu-a-1239 | 0.000545683 | 418.4089499 | | 19 | rs10875121 | 0.01834 | 0.8571 | 0.9025 | PDAOO | Years of schooling || id:ieu-a-1239 | 0.000577054 | 442.4767213 | | 20 | rs10887801 | 0.01087 | 0.4371 | 0.1124 | PDAOO | Years of schooling || id:ieu-a-1239 | 0.000762515 | 584.7937954 | | 21 | rs10940921 | -0.01089 | 0.5697 | 0.1379 | PDAOO | Years of schooling || id:ieu-a-1239 | 0.000736686 | 564.970277 | | 22 | rs10963297 | 0.01904 | 0.2517 | 0.3838 | PDAOO | Years of schooling || id:ieu-a-1239 | 0.000658604 | 505.049187 | | 23 | rs10994777 | 0.0146 | 0.1395 | 0.2384 | PDAOO | Years of schooling || id:ieu-a-1239 | 0.000562139 | 431.0333578 | | 24 | rs11023749 | 0.01132 | 0.6701 | 0.9640 | PDAOO | Years of schooling || id:ieu-a-1239 | 0.000724417 | 555.5541057 | | 25 | rs1106090 | 0.01173 | 0.6259 | 0.4006 | PDAOO | Years of schooling || id:ieu-a-1239 | 0.000745099 | 571.4270801 | | 26 | rs11081529 | -0.01311 | 0.2568 | 0.9720 | PDAOO | Years of schooling || id:ieu-a-1239 | 0.000701065 | 537.6330055 | | 27 | rs11123818 | 0.02081 | 0.3946 | 0.0705 | PDAOO | Years of schooling || id:ieu-a-1239 | 0.000745099 | 571.4270801 | | 28 | rs111821073 | 0.01385 | 0.1633 | 0.2565 | PDAOO | Years of schooling || id:ieu-a-1239 | 0.000550286 | 421.9398271 | | 29 | rs112687095 | 0.01325 | 0.1650 | 0.2110 | PDAOO | Years of schooling || id:ieu-a-1239 | 0.000547975 | 420.1669707 | | 30 | rs112806496 | 0.0187 | 0.0850 | 0.7909 | PDAOO | Years of schooling || id:ieu-a-1239 | 0.000427652 | 327.8679968 | | 31 | rs113182709 | 0.03225 | 0.0238 | 0.5767 | PDAOO | Years of schooling || id:ieu-a-1239 | 0.000230087 | 176.3663828 | | 32 | rs113520408 | 0.01304 | 0.2857 | 0.3978 | PDAOO | Years of schooling || id:ieu-a-1239 | 0.000679171 | 520.8319741 | | 33 | rs113615161 | -0.01472 | 0.1395 | 0.1988 | PDAOO | Years of schooling || id:ieu-a-1239 | 0.000521686 | 399.9989561 | | 34 | rs1143770 | 0.01136 | 0.5918 | 0.4196 | PDAOO | Years of schooling || id:ieu-a-1239 | 0.000758085 | 581.3938315 | | 35 | rs115000530 | 0.02892 | 0.0612 | 0.8264 | PDAOO | Years of schooling || id:ieu-a-1239 | 0.000342375 | 262.4665066 | | 36 | rs115454970 | -0.01185 | 0.3044 | 0.3742 | PDAOO | Years of schooling || id:ieu-a-1239 | 0.000655297 | 502.5112514 | | 37 | rs11601122 | -0.01947 | 0.1497 | 0.9012 | PDAOO | Years of schooling || id:ieu-a-1239 | 0.000567024 | 434.781474 | | 38 | rs11620355 | 0.01756 | 0.1156 | 0.2822 | PDAOO | Years of schooling || id:ieu-a-1239 | 0.000434776 | 333.3324634 | | 39 | rs11627087 | -0.01788 | 0.0850 | 0.6473 | PDAOO | Years of schooling || id:ieu-a-1239 | 0.000401345 | 307.6915047 | | 40 | rs11657342 | 0.01404 | 0.3554 | 0.7008 | PDAOO | Years of schooling || id:ieu-a-1239 | 0.000682725 | 523.558843 | | 41 | rs11663602 | -0.01213 | 0.2568 | 0.2104 | PDAOO | Years of schooling || id:ieu-a-1239 | 0.000686316 | 526.3144159 | | 42 | rs11678980 | -0.01744 | 0.4456 | 0.6783 | PDAOO | Years of schooling || id:ieu-a-1239 | 0.000758085 | 581.3938315 | | 43 | rs11681861 | -0.01435 | 0.1565 | 0.7959 | PDAOO | Years of schooling || id:ieu-a-1239 | 0.000503567 | 386.0993785 | | 44 | rs11694904 | 0.01215 | 0.3384 | 0.9794 | PDAOO | Years of schooling || id:ieu-a-1239 | 0.000704852 | 540.5391298 | | 45 | rs11732657 | -0.01274 | 0.7007 | 0.4667 | PDAOO | Years of schooling || id:ieu-a-1239 | 0.000661945 | 507.6128884 | | 46 | rs117468730 | -0.03521 | 0.0119 | 0.8831 | PDAOO | Years of schooling || id:ieu-a-1239 | 0.000218528 | 167.5037505 | | 47 | rs11752914 | -0.01208 | 0.1973 | 0.8006 | PDAOO | Years of schooling || id:ieu-a-1239 | 0.000603753 | 462.9617547 | | 48 | rs11772580 | -0.01199 | 0.2534 | 0.1764 | PDAOO | Years of schooling || id:ieu-a-1239 | 0.00064878 | 497.5111394 | | 49 | rs117799466 | 0.01173 | 0.3776 | 0.4166 | PDAOO | Years of schooling || id:ieu-a-1239 | 0.000658604 | 505.049187 | | 50 | rs11871429 | -0.01425 | 0.2041 | 0.9706 | PDAOO | Years of schooling || id:ieu-a-1239 | 0.000645571 | 495.048213 | | 51 | rs12028010 | -0.01696 | 0.2228 | 0.5051 | PDAOO | Years of schooling || id:ieu-a-1239 | 0.000645571 | 495.048213 | | 52 | rs12134151 | -0.01245 | 0.5221 | 0.9743 | PDAOO | Years of schooling || id:ieu-a-1239 | 0.000766997 | 588.2337589 | | 53 | rs12332731 | 0.01374 | 0.2024 | 0.2899 | PDAOO | Years of schooling || id:ieu-a-1239 | 0.000598218 | 458.7143992 | | 54 | rs12375949 | 0.01447 | 0.5697 | 0.0360 | PDAOO | Years of schooling || id:ieu-a-1239 | 0.000758085 | 581.3938315 | | 55 | rs12468040 | -0.01432 | 0.6037 | 0.0717 | PDAOO | Years of schooling || id:ieu-a-1239 | 0.000745099 | 571.4270801 | | 56 | rs12503522 | -0.01125 | 0.2483 | 0.6192 | PDAOO | Years of schooling || id:ieu-a-1239 | 0.000693612 | 531.9135054 | | 57 | rs12519073 | -0.01221 | 0.2381 | 0.9845 | PDAOO | Years of schooling || id:ieu-a-1239 | 0.000645571 | 495.048213 | | 58 | rs12574281 | 0.01077 | 0.3997 | 0.5404 | PDAOO | Years of schooling || id:ieu-a-1239 | 0.000740868 | 568.1803353 | | 59 | rs12602286 | 0.01701 | 0.8861 | 0.0843 | PDAOO | Years of schooling || id:ieu-a-1239 | 0.000511462 | 392.1558393 | | 60 | rs12643771 | 0.01518 | 0.3112 | 0.3037 | PDAOO | Years of schooling || id:ieu-a-1239 | 0.00070868 | 543.4768425 | | 61 | rs12682775 | 0.01187 | 0.2143 | 0.1648 | PDAOO | Years of schooling || id:ieu-a-1239 | 0.000639246 | 490.1947991 | | 62 | rs12804787 | -0.01814 | 0.0680 | 0.7075 | PDAOO | Years of schooling || id:ieu-a-1239 | 0.000398891 | 305.8095995 | | 63 | rs1291818 | -0.01085 | 0.5153 | 0.4466 | PDAOO | Years of schooling || id:ieu-a-1239 | 0.000766997 | 588.2337589 | | 64 | rs12940014 | 0.00936 | 0.5204 | 0.7497 | PDAOO | Years of schooling || id:ieu-a-1239 | 0.000766997 | 588.2337589 | | 65 | rs12955211 | 0.01097 | 0.3554 | 0.0388 | PDAOO | Years of schooling || id:ieu-a-1239 | 0.000716462 | 549.4491155 | | 66 | rs13010566 | 0.0106 | 0.5612 | 0.1280 | PDAOO | Years of schooling || id:ieu-a-1239 | 0.000766997 | 588.2337589 | | 67 | rs13029509 | -0.01049 | 0.4677 | 0.8593 | PDAOO | Years of schooling || id:ieu-a-1239 | 0.000766997 | 588.2337589 | | 68 | rs13090388 | 0.02852 | 0.3095 | 0.5638 | PDAOO | Years of schooling || id:ieu-a-1239 | 0.00070868 | 543.4768425 | | 69 | rs13130765 | -0.01014 | 0.4558 | 0.6098 | PDAOO | Years of schooling || id:ieu-a-1239 | 0.000753706 | 578.0331735 | | 70 | rs13141210 | 0.01361 | 0.5085 | 0.4568 | PDAOO | Years of schooling || id:ieu-a-1239 | 0.000758085 | 581.3938315 | | 71 | rs13145650 | -0.01918 | 0.9082 | 0.2934 | PDAOO | Years of schooling || id:ieu-a-1239 | 0.000426255 | 326.7965327 | | 72 | rs1334297 | 0.02449 | 0.7840 | 0.0351 | PDAOO | Years of schooling || id:ieu-a-1239 | 0.000679171 | 520.8319741 | | 73 | rs13422673 | -0.01201 | 0.4847 | 0.7081 | PDAOO | Years of schooling || id:ieu-a-1239 | 0.000766997 | 588.2337589 | | 74 | rs1363862 | -0.01171 | 0.2602 | 0.0867 | PDAOO | Years of schooling || id:ieu-a-1239 | 0.000679171 | 520.8319741 | | 75 | rs1381247 | -0.01013 | 0.2908 | 0.0821 | PDAOO | Years of schooling || id:ieu-a-1239 | 0.000716462 | 549.4491155 | | 76 | rs1391438 | -0.0167 | 0.6854 | 0.3170 | PDAOO | Years of schooling || id:ieu-a-1239 | 0.000712549 | 546.4466613 | | 77 | rs1427298 | 0.0102 | 0.4116 | 0.3737 | PDAOO | Years of schooling || id:ieu-a-1239 | 0.000758085 | 581.3938315 | | 78 | rs1450782 | -0.00945 | 0.6020 | 0.5134 | PDAOO | Years of schooling || id:ieu-a-1239 | 0.000753706 | 578.0331735 | | 79 | rs1455350 | -0.01614 | 0.4711 | 0.7248 | PDAOO | Years of schooling || id:ieu-a-1239 | 0.000766997 | 588.2337589 | | 80 | rs152603 | 0.01019 | 0.3861 | 0.4402 | PDAOO | Years of schooling || id:ieu-a-1239 | 0.000736686 | 564.970277 | | 81 | rs1566085 | 0.01645 | 0.5697 | 0.2342 | PDAOO | Years of schooling || id:ieu-a-1239 | 0.000762515 | 584.7937954 | | 82 | rs1569092 | 0.01807 | 0.1820 | 0.8313 | PDAOO | Years of schooling || id:ieu-a-1239 | 0.000557337 | 427.3493121 | | 83 | rs1584469 | -0.01303 | 0.3112 | 0.5465 | PDAOO | Years of schooling || id:ieu-a-1239 | 0.000704852 | 540.5391298 | | 84 | rs1592757 | -0.01045 | 0.3759 | 0.9519 | PDAOO | Years of schooling || id:ieu-a-1239 | 0.000724417 | 555.5541057 | | 85 | rs1595973 | -0.01002 | 0.5595 | 0.8098 | PDAOO | Years of schooling || id:ieu-a-1239 | 0.000753706 | 578.0331735 | | 86 | rs1618725 | 0.01477 | 0.5204 | 0.9238 | PDAOO | Years of schooling || id:ieu-a-1239 | 0.000749378 | 574.7111438 | | 87 | rs1620977 | -0.02046 | 0.6905 | 0.8640 | PDAOO | Years of schooling || id:ieu-a-1239 | 0.00066873 | 512.8191745 | | 88 | rs1671770 | -0.01342 | 0.8061 | 0.2585 | PDAOO | Years of schooling || id:ieu-a-1239 | 0.000584813 | 448.429323 | | 89 | rs16846463 | -0.02256 | 0.1173 | 0.2350 | PDAOO | Years of schooling || id:ieu-a-1239 | 0.000460881 | 353.3559683 | | 90 | rs16854920 | 0.01007 | 0.3554 | 0.2705 | PDAOO | Years of schooling || id:ieu-a-1239 | 0.000720417 | 552.484746 | | 91 | rs1689510 | 0.01761 | 0.3435 | 0.5223 | PDAOO | Years of schooling || id:ieu-a-1239 | 0.000724417 | 555.5541057 | | 92 | rs16995054 | -0.0139 | 0.2007 | 0.9837 | PDAOO | Years of schooling || id:ieu-a-1239 | 0.00062696 | 480.7679761 | | 93 | rs17048855 | 0.01184 | 0.3248 | 0.5909 | PDAOO | Years of schooling || id:ieu-a-1239 | 0.000728461 | 558.6577599 | | 94 | rs17110109 | 0.01023 | 0.3776 | 0.2800 | PDAOO | Years of schooling || id:ieu-a-1239 | 0.000745099 | 571.4270801 | | 95 | rs17126938 | 0.01536 | 0.1207 | 0.0950 | PDAOO | Years of schooling || id:ieu-a-1239 | 0.000521686 | 399.9989561 | | 96 | rs17425572 | -0.01224 | 0.5425 | 0.9958 | PDAOO | Years of schooling || id:ieu-a-1239 | 0.000766997 | 588.2337589 | | 97 | rs175325 | -0.01179 | 0.5816 | 0.9729 | PDAOO | Years of schooling || id:ieu-a-1239 | 0.000749378 | 574.7111438 | | 98 | rs17551064 | -0.01493 | 0.1599 | 0.3307 | PDAOO | Years of schooling || id:ieu-a-1239 | 0.000567024 | 434.781474 | | 99 | rs17563464 | -0.01477 | 0.2041 | 0.9198 | PDAOO | Years of schooling || id:ieu-a-1239 | 0.000615138 | 471.6968822 | | 100 | rs17565975 | -0.01142 | 0.5306 | 0.2561 | PDAOO | Years of schooling || id:ieu-a-1239 | 0.000762515 | 584.7937954 | | 101 | rs17598675 | 0.01199 | 0.5187 | 0.7313 | PDAOO | Years of schooling || id:ieu-a-1239 | 0.000766997 | 588.2337589 | | 102 | rs176218 | 0.01883 | 0.2007 | 0.0104 | PDAOO | Years of schooling || id:ieu-a-1239 | 0.00060656 | 465.1150652 | | 103 | rs1827540 | -0.0106 | 0.4660 | 0.7693 | PDAOO | Years of schooling || id:ieu-a-1239 | 0.000766997 | 588.2337589 | | 104 | rs1866823 | 0.01009 | 0.5510 | 0.2267 | PDAOO | Years of schooling || id:ieu-a-1239 | 0.000762515 | 584.7937954 | | 105 | rs1882273 | -0.01231 | 0.3469 | 0.8322 | PDAOO | Years of schooling || id:ieu-a-1239 | 0.000720417 | 552.484746 | | 106 | rs192436652 | -0.03497 | 0.0221 | 0.4995 | PDAOO | Years of schooling || id:ieu-a-1239 | 0.000239373 | 183.4857597 | | 107 | rs1925576 | 0.00997 | 0.4422 | 0.4276 | PDAOO | Years of schooling || id:ieu-a-1239 | 0.000762515 | 584.7937954 | | 108 | rs1947114 | 0.01071 | 0.2551 | 0.5223 | PDAOO | Years of schooling || id:ieu-a-1239 | 0.000679171 | 520.8319741 | | 109 | rs1949226 | -0.00965 | 0.5901 | 0.2354 | PDAOO | Years of schooling || id:ieu-a-1239 | 0.000749378 | 574.7111438 | | 110 | rs1964927 | -0.01423 | 0.6378 | 0.6690 | PDAOO | Years of schooling || id:ieu-a-1239 | 0.000736686 | 564.970277 | | 111 | rs2052285 | 0.01123 | 0.5765 | 0.3309 | PDAOO | Years of schooling || id:ieu-a-1239 | 0.000745099 | 571.4270801 | | 112 | rs2067854 | 0.01477 | 0.1820 | 0.8852 | PDAOO | Years of schooling || id:ieu-a-1239 | 0.000623962 | 478.4676508 | | 113 | rs2179152 | 0.01455 | 0.6429 | 0.9792 | PDAOO | Years of schooling || id:ieu-a-1239 | 0.000740868 | 568.1803353 | | 114 | rs2182505 | -0.01086 | 0.7364 | 0.6789 | PDAOO | Years of schooling || id:ieu-a-1239 | 0.000679171 | 520.8319741 | | 115 | rs225291 | -0.01205 | 0.8027 | 0.1994 | PDAOO | Years of schooling || id:ieu-a-1239 | 0.000609393 | 467.2885001 | | 116 | rs2256965 | -0.01128 | 0.5425 | 0.7341 | PDAOO | Years of schooling || id:ieu-a-1239 | 0.000740868 | 568.1803353 | | 117 | rs2283076 | -0.01143 | 0.2143 | 0.5382 | PDAOO | Years of schooling || id:ieu-a-1239 | 0.000639246 | 490.1947991 | | 118 | rs2287838 | -0.01152 | 0.5340 | 0.1460 | PDAOO | Years of schooling || id:ieu-a-1239 | 0.000762515 | 584.7937954 | | 119 | rs2302761 | 0.01354 | 0.1905 | 0.8647 | PDAOO | Years of schooling || id:ieu-a-1239 | 0.000623962 | 478.4676508 | | 120 | rs2414072 | -0.01005 | 0.4864 | 0.2055 | PDAOO | Years of schooling || id:ieu-a-1239 | 0.000762515 | 584.7937954 | | 121 | rs242093 | -0.01031 | 0.5476 | 0.1936 | PDAOO | Years of schooling || id:ieu-a-1239 | 0.000758085 | 581.3938315 | | 122 | rs2441111 | 0.01087 | 0.5323 | 0.6772 | PDAOO | Years of schooling || id:ieu-a-1239 | 0.000766997 | 588.2337589 | | 123 | rs2478208 | -0.0106 | 0.5000 | 0.5852 | PDAOO | Years of schooling || id:ieu-a-1239 | 0.000766997 | 588.2337589 | | 124 | rs2545798 | -0.01346 | 0.4949 | 0.8837 | PDAOO | Years of schooling || id:ieu-a-1239 | 0.000762515 | 584.7937954 | | 125 | rs2554835 | 0.00974 | 0.3980 | 0.0558 | PDAOO | Years of schooling || id:ieu-a-1239 | 0.000745099 | 571.4270801 | | 126 | rs2570497 | -0.01233 | 0.6735 | 0.7124 | PDAOO | Years of schooling || id:ieu-a-1239 | 0.000736686 | 564.970277 | | 127 | rs2725370 | 0.01536 | 0.7109 | 0.0090 | PDAOO | Years of schooling || id:ieu-a-1239 | 0.000697318 | 534.7579627 | | 128 | rs277828 | -0.01091 | 0.2568 | 0.6594 | PDAOO | Years of schooling || id:ieu-a-1239 | 0.00066532 | 510.2027501 | | 129 | rs2787101 | 0.00968 | 0.6173 | 0.2292 | PDAOO | Years of schooling || id:ieu-a-1239 | 0.000749378 | 574.7111438 | | 130 | rs2819336 | -0.01828 | 0.6616 | 0.1811 | PDAOO | Years of schooling || id:ieu-a-1239 | 0.000736686 | 564.970277 | | 131 | rs2820314 | -0.011 | 0.3163 | 0.7310 | PDAOO | Years of schooling || id:ieu-a-1239 | 0.000724417 | 555.5541057 | | 132 | rs28373063 | 0.01389 | 0.2075 | 0.4158 | PDAOO | Years of schooling || id:ieu-a-1239 | 0.000569499 | 436.6800831 | | 133 | rs28513670 | 0.01477 | 0.1531 | 0.7124 | PDAOO | Years of schooling || id:ieu-a-1239 | 0.000579617 | 444.4432845 | | 134 | rs2885198 | -0.01025 | 0.5017 | 0.3863 | PDAOO | Years of schooling || id:ieu-a-1239 | 0.000766997 | 588.2337589 | | 135 | rs2905426 | 0.01037 | 0.6446 | 0.7364 | PDAOO | Years of schooling || id:ieu-a-1239 | 0.000720417 | 552.484746 | | 136 | rs2923431 | 0.0114 | 0.6446 | 0.4026 | PDAOO | Years of schooling || id:ieu-a-1239 | 0.000740868 | 568.1803353 | | 137 | rs2971970 | 0.01654 | 0.7721 | 0.5276 | PDAOO | Years of schooling || id:ieu-a-1239 | 0.000629987 | 483.0905267 | | 138 | rs2998315 | 0.01269 | 0.5782 | 0.2116 | PDAOO | Years of schooling || id:ieu-a-1239 | 0.000762515 | 584.7937954 | | 139 | rs3013014 | -0.01024 | 0.6105 | 0.1779 | PDAOO | Years of schooling || id:ieu-a-1239 | 0.000758085 | 581.3938315 | | 140 | rs301800 | -0.01516 | 0.8197 | 0.8770 | PDAOO | Years of schooling || id:ieu-a-1239 | 0.000582203 | 446.4274063 | | 141 | rs3026996 | -0.01537 | 0.2840 | 0.0473 | PDAOO | Years of schooling || id:ieu-a-1239 | 0.000655297 | 502.5112514 | | 142 | rs31940 | 0.01548 | 0.1344 | 0.3365 | PDAOO | Years of schooling || id:ieu-a-1239 | 0.000530164 | 406.5030042 | | 143 | rs320693 | 0.01204 | 0.4728 | 0.0318 | PDAOO | Years of schooling || id:ieu-a-1239 | 0.000766997 | 588.2337589 | | 144 | rs337637 | 0.01123 | 0.3367 | 0.0387 | PDAOO | Years of schooling || id:ieu-a-1239 | 0.000736686 | 564.970277 | | 145 | rs34316 | -0.02016 | 0.5799 | 0.3702 | PDAOO | Years of schooling || id:ieu-a-1239 | 0.000736686 | 564.970277 | | 146 | rs34394051 | 0.01392 | 0.1599 | 0.2272 | PDAOO | Years of schooling || id:ieu-a-1239 | 0.000543411 | 416.6655793 | | 147 | rs34485537 | 0.01075 | 0.3895 | 0.2823 | PDAOO | Years of schooling || id:ieu-a-1239 | 0.000753706 | 578.0331735 | | 148 | rs34853711 | -0.01596 | 0.2466 | 0.8578 | PDAOO | Years of schooling || id:ieu-a-1239 | 0.000633043 | 485.4356263 | | 149 | rs35039375 | -0.01983 | 0.0935 | 0.5330 | PDAOO | Years of schooling || id:ieu-a-1239 | 0.000445159 | 341.2960376 | | 150 | rs35309068 | 0.01321 | 0.4660 | 0.8939 | PDAOO | Years of schooling || id:ieu-a-1239 | 0.000762515 | 584.7937954 | | 151 | rs35316276 | 0.01173 | 0.2942 | 0.7292 | PDAOO | Years of schooling || id:ieu-a-1239 | 0.000672174 | 515.4625723 | | 152 | rs35417702 | -0.01445 | 0.5765 | 0.1493 | PDAOO | Years of schooling || id:ieu-a-1239 | 0.000766997 | 588.2337589 | | 153 | rs35475880 | -0.01511 | 0.1837 | 0.7475 | PDAOO | Years of schooling || id:ieu-a-1239 | 0.00062696 | 480.7679761 | | 154 | rs36083520 | 0.01629 | 0.1667 | 0.0836 | PDAOO | Years of schooling || id:ieu-a-1239 | 0.000584813 | 448.429323 | | 155 | rs36119825 | 0.01063 | 0.4694 | 0.5196 | PDAOO | Years of schooling || id:ieu-a-1239 | 0.000762515 | 584.7937954 | | 156 | rs363096 | 0.01363 | 0.5748 | 0.8623 | PDAOO | Years of schooling || id:ieu-a-1239 | 0.000758085 | 581.3938315 | | 157 | rs3747631 | 0.02207 | 0.2279 | 0.1477 | PDAOO | Years of schooling || id:ieu-a-1239 | 0.00062696 | 480.7679761 | | 158 | rs3788556 | -0.01138 | 0.5408 | 0.1354 | PDAOO | Years of schooling || id:ieu-a-1239 | 0.000762515 | 584.7937954 | | 159 | rs3800546 | -0.01183 | 0.2704 | 0.9035 | PDAOO | Years of schooling || id:ieu-a-1239 | 0.000672174 | 515.4625723 | | 160 | rs3809634 | 0.01058 | 0.3350 | 0.8269 | PDAOO | Years of schooling || id:ieu-a-1239 | 0.000704852 | 540.5391298 | | 161 | rs3890802 | -0.01133 | 0.2687 | 0.6860 | PDAOO | Years of schooling || id:ieu-a-1239 | 0.000682725 | 523.558843 | | 162 | rs3897821 | -0.01502 | 0.3503 | 0.4263 | PDAOO | Years of schooling || id:ieu-a-1239 | 0.000724417 | 555.5541057 | | 163 | rs401687 | 0.01144 | 0.4558 | 0.8018 | PDAOO | Years of schooling || id:ieu-a-1239 | 0.000766997 | 588.2337589 | | 164 | rs406413 | -0.01695 | 0.2109 | 0.4539 | PDAOO | Years of schooling || id:ieu-a-1239 | 0.000623962 | 478.4676508 | | 165 | rs4073894 | 0.01524 | 0.1769 | 0.9029 | PDAOO | Years of schooling || id:ieu-a-1239 | 0.000618052 | 473.9324124 | | 166 | rs4328757 | 0.01067 | 0.6514 | 0.2092 | PDAOO | Years of schooling || id:ieu-a-1239 | 0.000749378 | 574.7111438 | | 167 | rs4352658 | -0.0212 | 0.0901 | 0.3831 | PDAOO | Years of schooling || id:ieu-a-1239 | 0.000423488 | 324.6744773 | | 168 | rs4369924 | 0.01362 | 0.1684 | 0.8389 | PDAOO | Years of schooling || id:ieu-a-1239 | 0.000557337 | 427.3493121 | | 169 | rs4382592 | 0.01636 | 0.6990 | 0.7202 | PDAOO | Years of schooling || id:ieu-a-1239 | 0.000704852 | 540.5391298 | | 170 | rs4384309 | 0.0109 | 0.4796 | 0.8338 | PDAOO | Years of schooling || id:ieu-a-1239 | 0.000758085 | 581.3938315 | | 171 | rs4392737 | -0.0097 | 0.3827 | 0.3821 | PDAOO | Years of schooling || id:ieu-a-1239 | 0.000753706 | 578.0331735 | | 172 | rs4442732 | -0.01063 | 0.5969 | 0.2458 | PDAOO | Years of schooling || id:ieu-a-1239 | 0.000740868 | 568.1803353 | | 173 | rs4497562 | -0.01204 | 0.2823 | 0.5144 | PDAOO | Years of schooling || id:ieu-a-1239 | 0.000679171 | 520.8319741 | | 174 | rs4667025 | 0.00957 | 0.3878 | 0.1434 | PDAOO | Years of schooling || id:ieu-a-1239 | 0.000749378 | 574.7111438 | | 175 | rs4700393 | 0.02086 | 0.5289 | 0.0227 | PDAOO | Years of schooling || id:ieu-a-1239 | 0.000766997 | 588.2337589 | | 176 | rs4726070 | 0.01251 | 0.6207 | 0.1433 | PDAOO | Years of schooling || id:ieu-a-1239 | 0.000749378 | 574.7111438 | | 177 | rs4733264 | -0.00954 | 0.6310 | 0.0750 | PDAOO | Years of schooling || id:ieu-a-1239 | 0.000749378 | 574.7111438 | | 178 | rs4743923 | 0.00985 | 0.3673 | 0.6727 | PDAOO | Years of schooling || id:ieu-a-1239 | 0.000740868 | 568.1803353 | | 179 | rs4757957 | 0.0141 | 0.6514 | 0.0134 | PDAOO | Years of schooling || id:ieu-a-1239 | 0.00070868 | 543.4768425 | | 180 | rs4766424 | -0.0141 | 0.9116 | 0.1502 | PDAOO | Years of schooling || id:ieu-a-1239 | 0.000505518 | 387.5958877 | | 181 | rs4787457 | -0.01741 | 0.3146 | 0.4753 | PDAOO | Years of schooling || id:ieu-a-1239 | 0.000740868 | 568.1803353 | | 182 | rs4810227 | 0.01272 | 0.6344 | 0.6446 | PDAOO | Years of schooling || id:ieu-a-1239 | 0.000745099 | 571.4270801 | | 183 | rs4839155 | -0.01251 | 0.2500 | 0.3104 | PDAOO | Years of schooling || id:ieu-a-1239 | 0.000652022 | 499.9986951 | | 184 | rs4846724 | 0.01018 | 0.4915 | 0.8228 | PDAOO | Years of schooling || id:ieu-a-1239 | 0.000766997 | 588.2337589 | | 185 | rs4870482 | -0.01083 | 0.2585 | 0.2342 | PDAOO | Years of schooling || id:ieu-a-1239 | 0.000686316 | 526.3144159 | | 186 | rs4888746 | -0.00952 | 0.3776 | 0.7106 | PDAOO | Years of schooling || id:ieu-a-1239 | 0.000749378 | 574.7111438 | | 187 | rs4904523 | -0.00936 | 0.4592 | 0.3458 | PDAOO | Years of schooling || id:ieu-a-1239 | 0.000766997 | 588.2337589 | | 188 | rs4945424 | -0.00992 | 0.4150 | 0.0562 | PDAOO | Years of schooling || id:ieu-a-1239 | 0.000762515 | 584.7937954 | | 189 | rs4964046 | 0.01053 | 0.3350 | 0.9260 | PDAOO | Years of schooling || id:ieu-a-1239 | 0.00073255 | 561.7962866 | | 190 | rs4972400 | 0.01156 | 0.3520 | 0.7521 | PDAOO | Years of schooling || id:ieu-a-1239 | 0.000720417 | 552.484746 | | 191 | rs4984541 | 0.01233 | 0.2415 | 0.8283 | PDAOO | Years of schooling || id:ieu-a-1239 | 0.000629987 | 483.0905267 | | 192 | rs535307 | -0.01004 | 0.6769 | 0.7273 | PDAOO | Years of schooling || id:ieu-a-1239 | 0.00070868 | 543.4768425 | | 193 | rs55736314 | 0.01431 | 0.4167 | 0.1762 | PDAOO | Years of schooling || id:ieu-a-1239 | 0.000749378 | 574.7111438 | | 194 | rs55771711 | 0.01555 | 0.2279 | 0.5939 | PDAOO | Years of schooling || id:ieu-a-1239 | 0.000655297 | 502.5112514 | | 195 | rs56391344 | 0.01571 | 0.2381 | 0.0543 | PDAOO | Years of schooling || id:ieu-a-1239 | 0.000661945 | 507.6128884 | | 196 | rs575113 | 0.01285 | 0.2772 | 0.2738 | PDAOO | Years of schooling || id:ieu-a-1239 | 0.000701065 | 537.6330055 | | 197 | rs59123361 | -0.02094 | 0.1054 | 0.8040 | PDAOO | Years of schooling || id:ieu-a-1239 | 0.000448217 | 343.6417148 | | 198 | rs60483752 | 0.01078 | 0.5816 | 0.9929 | PDAOO | Years of schooling || id:ieu-a-1239 | 0.000758085 | 581.3938315 | | 199 | rs6122735 | 0.0105 | 0.4133 | 0.5581 | PDAOO | Years of schooling || id:ieu-a-1239 | 0.000749378 | 574.7111438 | | 200 | rs6123924 | -0.01528 | 0.1599 | 0.8652 | PDAOO | Years of schooling || id:ieu-a-1239 | 0.000554966 | 425.5308043 | | 201 | rs613872 | -0.0175 | 0.8282 | 0.4282 | PDAOO | Years of schooling || id:ieu-a-1239 | 0.000574513 | 440.5274847 | | 202 | rs61747885 | 0.01383 | 0.1412 | 0.9189 | PDAOO | Years of schooling || id:ieu-a-1239 | 0.000554966 | 425.5308043 | | 203 | rs62097985 | -0.01288 | 0.4116 | 0.0679 | PDAOO | Years of schooling || id:ieu-a-1239 | 0.000758085 | 581.3938315 | | 204 | rs62157915 | 0.02091 | 0.0595 | 0.5084 | PDAOO | Years of schooling || id:ieu-a-1239 | 0.000374829 | 287.3555719 | | 205 | rs62183776 | -0.01308 | 0.1905 | 0.4861 | PDAOO | Years of schooling || id:ieu-a-1239 | 0.000600973 | 460.8282904 | | 206 | rs62184480 | -0.01528 | 0.2449 | 0.9882 | PDAOO | Years of schooling || id:ieu-a-1239 | 0.000682725 | 523.558843 | | 207 | rs622169 | 0.00999 | 0.4677 | 0.0670 | PDAOO | Years of schooling || id:ieu-a-1239 | 0.00073255 | 561.7962866 | | 208 | rs62439690 | -0.01087 | 0.2670 | 0.1406 | PDAOO | Years of schooling || id:ieu-a-1239 | 0.000672174 | 515.4625723 | | 209 | rs62444881 | 0.01815 | 0.1905 | 0.1418 | PDAOO | Years of schooling || id:ieu-a-1239 | 0.000600973 | 460.8282904 | | 210 | rs6493265 | -0.01385 | 0.3895 | 0.0205 | PDAOO | Years of schooling || id:ieu-a-1239 | 0.000749378 | 574.7111438 | | 211 | rs6513959 | -0.01177 | 0.2789 | 0.7378 | PDAOO | Years of schooling || id:ieu-a-1239 | 0.000704852 | 540.5391298 | | 212 | rs6557171 | 0.01567 | 0.7245 | 0.4967 | PDAOO | Years of schooling || id:ieu-a-1239 | 0.000720417 | 552.484746 | | 213 | rs663234 | -0.01005 | 0.6122 | 0.7764 | PDAOO | Years of schooling || id:ieu-a-1239 | 0.000749378 | 574.7111438 | | 214 | rs66568921 | 0.01565 | 0.3639 | 0.9522 | PDAOO | Years of schooling || id:ieu-a-1239 | 0.000716462 | 549.4491155 | | 215 | rs6731373 | -0.01256 | 0.3367 | 0.1278 | PDAOO | Years of schooling || id:ieu-a-1239 | 0.000720417 | 552.484746 | | 216 | rs6731967 | -0.01186 | 0.2092 | 0.6913 | PDAOO | Years of schooling || id:ieu-a-1239 | 0.000655297 | 502.5112514 | | 217 | rs67885444 | 0.01406 | 0.1718 | 0.0833 | PDAOO | Years of schooling || id:ieu-a-1239 | 0.000562139 | 431.0333578 | | 218 | rs67890737 | -0.01141 | 0.3265 | 0.5935 | PDAOO | Years of schooling || id:ieu-a-1239 | 0.000728461 | 558.6577599 | | 219 | rs6803651 | 0.01131 | 0.4150 | 0.6639 | PDAOO | Years of schooling || id:ieu-a-1239 | 0.000758085 | 581.3938315 | | 220 | rs6867851 | -0.012 | 0.4235 | 0.8496 | PDAOO | Years of schooling || id:ieu-a-1239 | 0.000753706 | 578.0331735 | | 221 | rs6938002 | -0.01008 | 0.3963 | 0.6804 | PDAOO | Years of schooling || id:ieu-a-1239 | 0.000753706 | 578.0331735 | | 222 | rs6959891 | -0.01136 | 0.2959 | 0.3548 | PDAOO | Years of schooling || id:ieu-a-1239 | 0.000689944 | 529.0991483 | | 223 | rs7012546 | 0.01009 | 0.4201 | 0.3166 | PDAOO | Years of schooling || id:ieu-a-1239 | 0.000758085 | 581.3938315 | | 224 | rs7016302 | 0.01243 | 0.1769 | 0.9644 | PDAOO | Years of schooling || id:ieu-a-1239 | 0.000571995 | 438.5953466 | | 225 | rs7029718 | 0.02439 | 0.4354 | 0.5647 | PDAOO | Years of schooling || id:ieu-a-1239 | 0.000749378 | 574.7111438 | | 226 | rs7031698 | 0.01248 | 0.7755 | 0.4931 | PDAOO | Years of schooling || id:ieu-a-1239 | 0.000633043 | 485.4356263 | | 227 | rs710629 | 0.01053 | 0.6565 | 0.5734 | PDAOO | Years of schooling || id:ieu-a-1239 | 0.000736686 | 564.970277 | | 228 | rs71646142 | 0.01286 | 0.1735 | 0.1721 | PDAOO | Years of schooling || id:ieu-a-1239 | 0.000600973 | 460.8282904 | | 229 | rs7233920 | -0.01315 | 0.2160 | 0.3362 | PDAOO | Years of schooling || id:ieu-a-1239 | 0.000645571 | 495.048213 | | 230 | rs7257460 | -0.01145 | 0.2704 | 0.4739 | PDAOO | Years of schooling || id:ieu-a-1239 | 0.000689944 | 529.0991483 | | 231 | rs7278859 | 0.01013 | 0.3078 | 0.0861 | PDAOO | Years of schooling || id:ieu-a-1239 | 0.000704852 | 540.5391298 | | 232 | rs72828517 | 0.01836 | 0.1412 | 0.9488 | PDAOO | Years of schooling || id:ieu-a-1239 | 0.000582203 | 446.4274063 | | 233 | rs72840994 | 0.01247 | 0.1820 | 0.4388 | PDAOO | Years of schooling || id:ieu-a-1239 | 0.000603753 | 462.9617547 | | 234 | rs730384 | 0.01016 | 0.4558 | 0.1821 | PDAOO | Years of schooling || id:ieu-a-1239 | 0.000762515 | 584.7937954 | | 235 | rs7315713 | -0.01022 | 0.6633 | 0.2585 | PDAOO | Years of schooling || id:ieu-a-1239 | 0.000697318 | 534.7579627 | | 236 | rs7321274 | -0.01275 | 0.1956 | 0.0126 | PDAOO | Years of schooling || id:ieu-a-1239 | 0.000618052 | 473.9324124 | | 237 | rs73301698 | -0.01291 | 0.2262 | 0.0764 | PDAOO | Years of schooling || id:ieu-a-1239 | 0.00062696 | 480.7679761 | | 238 | rs7332724 | -0.01149 | 0.2619 | 0.6553 | PDAOO | Years of schooling || id:ieu-a-1239 | 0.000689944 | 529.0991483 | | 239 | rs73344830 | -0.0172 | 0.6020 | 0.6087 | PDAOO | Years of schooling || id:ieu-a-1239 | 0.000758085 | 581.3938315 | | 240 | rs736282 | -0.01082 | 0.5153 | 0.6340 | PDAOO | Years of schooling || id:ieu-a-1239 | 0.000766997 | 588.2337589 | | 241 | rs743316 | -0.01185 | 0.1820 | 0.2409 | PDAOO | Years of schooling || id:ieu-a-1239 | 0.00062696 | 480.7679761 | | 242 | rs74643044 | 0.02323 | 0.0272 | 0.2862 | PDAOO | Years of schooling || id:ieu-a-1239 | 0.000337942 | 259.0666814 | | 243 | rs74701752 | 0.01591 | 0.0952 | 0.5781 | PDAOO | Years of schooling || id:ieu-a-1239 | 0.000457648 | 350.8762773 | | 244 | rs7481514 | 0.01072 | 0.6650 | 0.9494 | PDAOO | Years of schooling || id:ieu-a-1239 | 0.00073255 | 561.7962866 | | 245 | rs74998289 | -0.01821 | 0.2398 | 0.5477 | PDAOO | Years of schooling || id:ieu-a-1239 | 0.000612252 | 469.4823428 | | 246 | rs7594904 | 0.00969 | 0.4184 | 0.9165 | PDAOO | Years of schooling || id:ieu-a-1239 | 0.000753706 | 578.0331735 | | 247 | rs7603132 | 0.01317 | 0.1548 | 0.4578 | PDAOO | Years of schooling || id:ieu-a-1239 | 0.00060656 | 465.1150652 | | 248 | rs76076331 | 0.01873 | 0.1310 | 0.6194 | PDAOO | Years of schooling || id:ieu-a-1239 | 0.000525891 | 403.2247541 | | 249 | rs7650602 | 0.00939 | 0.4286 | 0.1757 | PDAOO | Years of schooling || id:ieu-a-1239 | 0.000762515 | 584.7937954 | | 250 | rs76878669 | -0.01399 | 0.2534 | 0.1403 | PDAOO | Years of schooling || id:ieu-a-1239 | 0.000636129 | 487.803605 | | 251 | rs77025239 | -0.01422 | 0.1088 | 0.5850 | PDAOO | Years of schooling || id:ieu-a-1239 | 0.000557337 | 427.3493121 | | 252 | rs77128898 | -0.02769 | 0.0221 | 0.5052 | PDAOO | Years of schooling || id:ieu-a-1239 | 0.000270652 | 207.4683382 | | 253 | rs77702622 | -0.02447 | 0.0765 | 0.1308 | PDAOO | Years of schooling || id:ieu-a-1239 | 0.000371627 | 284.8995414 | | 254 | rs77719387 | -0.04597 | 0.0170 | 0.5823 | PDAOO | Years of schooling || id:ieu-a-1239 | 0.000179705 | 137.7406874 | | 255 | rs77835879 | -0.01601 | 0.0901 | 0.8051 | PDAOO | Years of schooling || id:ieu-a-1239 | 0.000452883 | 347.221316 | | 256 | rs7796203 | -0.01074 | 0.5255 | 0.0464 | PDAOO | Years of schooling || id:ieu-a-1239 | 0.000762515 | 584.7937954 | | 257 | rs7803932 | 0.0143 | 0.1565 | 0.2100 | PDAOO | Years of schooling || id:ieu-a-1239 | 0.000577054 | 442.4767213 | | 258 | rs7833201 | -0.01532 | 0.1344 | 0.5681 | PDAOO | Years of schooling || id:ieu-a-1239 | 0.000497804 | 381.6783932 | | 259 | rs7863447 | 0.01678 | 0.8333 | 0.2645 | PDAOO | Years of schooling || id:ieu-a-1239 | 0.000559727 | 429.1834293 | | 260 | rs78721320 | 0.01307 | 0.2041 | 0.5880 | PDAOO | Years of schooling || id:ieu-a-1239 | 0.000595488 | 456.6198129 | | 261 | rs790647 | -0.01482 | 0.2347 | 0.3277 | PDAOO | Years of schooling || id:ieu-a-1239 | 0.000645571 | 495.048213 | | 262 | rs7920624 | -0.01181 | 0.4932 | 0.0910 | PDAOO | Years of schooling || id:ieu-a-1239 | 0.000766997 | 588.2337589 | | 263 | rs7924036 | 0.01501 | 0.5391 | 0.8388 | PDAOO | Years of schooling || id:ieu-a-1239 | 0.000766997 | 588.2337589 | | 264 | rs79265434 | 0.02331 | 0.1173 | 0.3634 | PDAOO | Years of schooling || id:ieu-a-1239 | 0.000497804 | 381.6783932 | | 265 | rs79269403 | 0.01447 | 0.2228 | 0.8138 | PDAOO | Years of schooling || id:ieu-a-1239 | 0.000639246 | 490.1947991 | | 266 | rs7928622 | 0.01011 | 0.3078 | 0.6550 | PDAOO | Years of schooling || id:ieu-a-1239 | 0.000720417 | 552.484746 | | 267 | rs795230 | 0.00952 | 0.4184 | 0.4233 | PDAOO | Years of schooling || id:ieu-a-1239 | 0.000758085 | 581.3938315 | | 268 | rs79523955 | -0.01802 | 0.0952 | 0.7454 | PDAOO | Years of schooling || id:ieu-a-1239 | 0.000460881 | 353.3559683 | | 269 | rs7977614 | 0.01325 | 0.3078 | 0.3722 | PDAOO | Years of schooling || id:ieu-a-1239 | 0.000658604 | 505.049187 | | 270 | rs7993663 | 0.0118 | 0.3571 | 0.8580 | PDAOO | Years of schooling || id:ieu-a-1239 | 0.00073255 | 561.7962866 | | 271 | rs8008382 | 0.01208 | 0.6871 | 0.3490 | PDAOO | Years of schooling || id:ieu-a-1239 | 0.000704852 | 540.5391298 | | 272 | rs80171383 | 0.0145 | 0.1241 | 0.0284 | PDAOO | Years of schooling || id:ieu-a-1239 | 0.000541157 | 414.9366764 | | 273 | rs8020034 | 0.01782 | 0.2058 | 0.3782 | PDAOO | Years of schooling || id:ieu-a-1239 | 0.000584813 | 448.429323 | | 274 | rs818415 | 0.01235 | 0.1820 | 0.0726 | PDAOO | Years of schooling || id:ieu-a-1239 | 0.000595488 | 456.6198129 | | 275 | rs837080 | 0.01092 | 0.4932 | 0.6390 | PDAOO | Years of schooling || id:ieu-a-1239 | 0.000766997 | 588.2337589 | | 276 | rs892612 | 0.01464 | 0.8418 | 0.7881 | PDAOO | Years of schooling || id:ieu-a-1239 | 0.000550286 | 421.9398271 | | 277 | rs894067 | 0.01041 | 0.3929 | 0.2705 | PDAOO | Years of schooling || id:ieu-a-1239 | 0.000745099 | 571.4270801 | | 278 | rs9289300 | 0.01512 | 0.1837 | 0.9565 | PDAOO | Years of schooling || id:ieu-a-1239 | 0.000557337 | 427.3493121 | | 279 | rs9320493 | -0.01394 | 0.8639 | 0.2222 | PDAOO | Years of schooling || id:ieu-a-1239 | 0.000543411 | 416.6655793 | | 280 | rs9342482 | 0.01264 | 0.2908 | 0.9241 | PDAOO | Years of schooling || id:ieu-a-1239 | 0.000661945 | 507.6128884 | | 281 | rs9349956 | 0.01881 | 0.2398 | 0.7276 | PDAOO | Years of schooling || id:ieu-a-1239 | 0.000579617 | 444.4432845 | | 282 | rs9372625 | 0.02383 | 0.4133 | 0.3554 | PDAOO | Years of schooling || id:ieu-a-1239 | 0.000740868 | 568.1803353 | | 283 | rs9384679 | -0.00959 | 0.4082 | 0.8912 | PDAOO | Years of schooling || id:ieu-a-1239 | 0.000740868 | 568.1803353 | | 284 | rs9386319 | 0.00991 | 0.4269 | 0.4327 | PDAOO | Years of schooling || id:ieu-a-1239 | 0.000749378 | 574.7111438 | | 285 | rs9386787 | 0.00958 | 0.5136 | 0.6713 | PDAOO | Years of schooling || id:ieu-a-1239 | 0.000766997 | 588.2337589 | | 286 | rs9436866 | 0.01882 | 0.0952 | 0.5357 | PDAOO | Years of schooling || id:ieu-a-1239 | 0.000451317 | 346.0198582 | | 287 | rs9503598 | 0.01079 | 0.4388 | 0.9674 | PDAOO | Years of schooling || id:ieu-a-1239 | 0.000762515 | 584.7937954 | | 288 | rs9529119 | -0.01295 | 0.8027 | 0.4593 | PDAOO | Years of schooling || id:ieu-a-1239 | 0.000639246 | 490.1947991 | | 289 | rs9556958 | -0.0108 | 0.5289 | 0.1792 | PDAOO | Years of schooling || id:ieu-a-1239 | 0.000766997 | 588.2337589 | | 290 | rs9616906 | 0.01497 | 0.4235 | 0.1762 | PDAOO | Years of schooling || id:ieu-a-1239 | 0.000758085 | 581.3938315 | | 291 | rs9679654 | 0.01042 | 0.4847 | 0.7171 | PDAOO | Years of schooling || id:ieu-a-1239 | 0.000758085 | 581.3938315 | | 292 | rs969512 | 0.01249 | 0.2959 | 0.8525 | PDAOO | Years of schooling || id:ieu-a-1239 | 0.000728461 | 558.6577599 | | 293 | rs9704097 | -0.0103 | 0.4728 | 0.0027 | PDAOO | Years of schooling || id:ieu-a-1239 | 0.000762515 | 584.7937954 | | 294 | rs9882532 | -0.01208 | 0.3639 | 0.5723 | PDAOO | Years of schooling || id:ieu-a-1239 | 0.000736686 | 564.970277 | | 295 | rs9914918 | 0.01155 | 0.2823 | 0.5703 | PDAOO | Years of schooling || id:ieu-a-1239 | 0.000689944 | 529.0991483 | | 296 | rs9933256 | -0.01134 | 0.4082 | 0.0570 | PDAOO | Years of schooling || id:ieu-a-1239 | 0.000758085 | 581.3938315 | | 297 | rs9936270 | -0.0136 | 0.3078 | 0.6473 | PDAOO | Years of schooling || id:ieu-a-1239 | 0.000658604 | 505.049187 | | 298 | rs9938678 | 0.01355 | 0.2432 | 0.7112 | PDAOO | Years of schooling || id:ieu-a-1239 | 0.000636129 | 487.803605 | | 299 | rs9964724 | 0.01978 | 0.6599 | 0.5291 | PDAOO | Years of schooling || id:ieu-a-1239 | 0.000712549 | 546.4466613 | | 300 | rs9995567 | 0.00998 | 0.3827 | 0.2687 | PDAOO | Years of schooling || id:ieu-a-1239 | 0.00073255 | 561.7962866 | | 1 | rs10073890 | -0.01262 | 0.7364 | 0.0017 | PD || id:ieu-b-7 | Years of schooling || id:ieu-a-1239 | 0.001087386 | 834.2180505 | | 2 | rs1008078 | -0.01738 | 0.4099 | 0.6388 | PD || id:ieu-b-7 | Years of schooling || id:ieu-a-1239 | 0.001231774 | 945.1256526 | | 3 | rs10189857 | -0.01725 | 0.4184 | 0.0321 | PD || id:ieu-b-7 | Years of schooling || id:ieu-a-1239 | 0.001246163 | 956.1797538 | | 4 | rs10191758 | 0.01631 | 0.3810 | 0.6688 | PD || id:ieu-b-7 | Years of schooling || id:ieu-a-1239 | 0.001217714 | 934.3242166 | | 5 | rs10205801 | -0.01053 | 0.5068 | 0.1083 | PD || id:ieu-b-7 | Years of schooling || id:ieu-a-1239 | 0.001246163 | 956.1797538 | | 6 | rs10215082 | 0.01303 | 0.5612 | 0.0516 | PD || id:ieu-b-7 | Years of schooling || id:ieu-a-1239 | 0.001238927 | 950.6205692 | | 7 | rs10240905 | 0.01167 | 0.6684 | 0.7388 | PD || id:ieu-b-7 | Years of schooling || id:ieu-a-1239 | 0.001203971 | 923.7668808 | | 8 | rs10456918 | 0.01485 | 0.1820 | 0.6287 | PD || id:ieu-b-7 | Years of schooling || id:ieu-a-1239 | 0.000951592 | 729.9407942 | | 9 | rs10760023 | 0.01095 | 0.3316 | 0.5184 | PD || id:ieu-b-7 | Years of schooling || id:ieu-a-1239 | 0.001164542 | 893.4794421 | | 10 | rs10765775 | 0.01488 | 0.3963 | 0.0730 | PD || id:ieu-b-7 | Years of schooling || id:ieu-a-1239 | 0.001210803 | 929.0155563 | | 11 | rs10772644 | 0.01614 | 0.8929 | 0.9469 | PD || id:ieu-b-7 | Years of schooling || id:ieu-a-1239 | 0.000798462 | 612.3847861 | | 12 | rs10773002 | -0.02191 | 0.7211 | 0.1613 | PD || id:ieu-b-7 | Years of schooling || id:ieu-a-1239 | 0.001081873 | 829.9834411 | | 13 | rs10797055 | 0.00986 | 0.5102 | 0.0146 | PD || id:ieu-b-7 | Years of schooling || id:ieu-a-1239 | 0.001238927 | 950.6205692 | | 14 | rs10798418 | -0.00957 | 0.4660 | 0.5551 | PD || id:ieu-b-7 | Years of schooling || id:ieu-a-1239 | 0.001231774 | 945.1256526 | | 15 | rs10856785 | -0.01132 | 0.7296 | 0.5547 | PD || id:ieu-b-7 | Years of schooling || id:ieu-a-1239 | 0.001110015 | 851.5975932 | | 16 | rs10862376 | 0.01616 | 0.1361 | 0.7255 | PD || id:ieu-b-7 | Years of schooling || id:ieu-a-1239 | 0.000891922 | 684.1286105 | | 17 | rs10875121 | 0.01834 | 0.8571 | 0.6426 | PD || id:ieu-b-7 | Years of schooling || id:ieu-a-1239 | 0.000943179 | 723.4811412 | | 18 | rs10887801 | 0.01087 | 0.4371 | 0.9694 | PD || id:ieu-b-7 | Years of schooling || id:ieu-a-1239 | 0.001246163 | 956.1797538 | | 19 | rs10940921 | -0.01089 | 0.5697 | 0.9190 | PD || id:ieu-b-7 | Years of schooling || id:ieu-a-1239 | 0.001203971 | 923.7668808 | | 20 | rs10963297 | 0.01904 | 0.2517 | 0.3698 | PD || id:ieu-b-7 | Years of schooling || id:ieu-a-1239 | 0.001076414 | 825.7916056 | | 21 | rs10994777 | 0.0146 | 0.1395 | 0.2800 | PD || id:ieu-b-7 | Years of schooling || id:ieu-a-1239 | 0.000918809 | 704.770422 | | 22 | rs11023749 | 0.01132 | 0.6701 | 0.4981 | PD || id:ieu-b-7 | Years of schooling || id:ieu-a-1239 | 0.001183929 | 908.3707661 | | 23 | rs1105307 | -0.01173 | 0.2449 | 0.5621 | PD || id:ieu-b-7 | Years of schooling || id:ieu-a-1239 | 0.001092957 | 838.4960918 | | 24 | rs1105307 | -0.01173 | 0.2449 | 0.5621 | PD || id:ieu-b-7 | Years of schooling || id:ieu-a-1239 | 0.001092957 | 838.4960918 | | 25 | rs1106090 | 0.01173 | 0.6259 | 0.2564 | PD || id:ieu-b-7 | Years of schooling || id:ieu-a-1239 | 0.001217714 | 934.3242166 | | 26 | rs11081529 | -0.01311 | 0.2568 | 0.1733 | PD || id:ieu-b-7 | Years of schooling || id:ieu-a-1239 | 0.001145781 | 879.0684833 | | 27 | rs112687095 | 0.01325 | 0.1650 | 0.1450 | PD || id:ieu-b-7 | Years of schooling || id:ieu-a-1239 | 0.000895667 | 687.0031004 | | 28 | rs112806496 | 0.0187 | 0.0850 | 0.9541 | PD || id:ieu-b-7 | Years of schooling || id:ieu-a-1239 | 0.000699051 | 536.0876652 | | 29 | rs113182709 | 0.03225 | 0.0238 | 0.7891 | PD || id:ieu-b-7 | Years of schooling || id:ieu-a-1239 | 0.000376154 | 288.3716718 | | 30 | rs113520408 | 0.01304 | 0.2857 | 0.0411 | PD || id:ieu-b-7 | Years of schooling || id:ieu-a-1239 | 0.001110015 | 851.5975932 | | 31 | rs113615161 | -0.01472 | 0.1395 | 0.9873 | PD || id:ieu-b-7 | Years of schooling || id:ieu-a-1239 | 0.000852711 | 654.0269516 | | 32 | rs1143770 | 0.01136 | 0.5918 | 0.2508 | PD || id:ieu-b-7 | Years of schooling || id:ieu-a-1239 | 0.001238927 | 950.6205692 | | 33 | rs115000530 | 0.02892 | 0.0612 | 0.6614 | PD || id:ieu-b-7 | Years of schooling || id:ieu-a-1239 | 0.000559686 | 429.151543 | | 34 | rs115454970 | -0.01185 | 0.3044 | 0.4089 | PD || id:ieu-b-7 | Years of schooling || id:ieu-a-1239 | 0.001071011 | 821.641899 | | 35 | rs11601122 | -0.01947 | 0.1497 | 0.3813 | PD || id:ieu-b-7 | Years of schooling || id:ieu-a-1239 | 0.000926791 | 710.8988604 | | 36 | rs11620355 | 0.01756 | 0.1156 | 0.7001 | PD || id:ieu-b-7 | Years of schooling || id:ieu-a-1239 | 0.000710694 | 545.0224597 | | 37 | rs11627087 | -0.01788 | 0.0850 | 0.9219 | PD || id:ieu-b-7 | Years of schooling || id:ieu-a-1239 | 0.000656061 | 503.0976551 | | 38 | rs11635092 | -0.01231 | 0.3639 | 0.8944 | PD || id:ieu-b-7 | Years of schooling || id:ieu-a-1239 | 0.001203971 | 923.7668808 | | 39 | rs11657342 | 0.01404 | 0.3554 | 0.4264 | PD || id:ieu-b-7 | Years of schooling || id:ieu-a-1239 | 0.00111582 | 856.0562194 | | 40 | rs11663602 | -0.01213 | 0.2568 | 0.6804 | PD || id:ieu-b-7 | Years of schooling || id:ieu-a-1239 | 0.001121686 | 860.5617784 | | 41 | rs11678980 | -0.01744 | 0.4456 | 0.0221 | PD || id:ieu-b-7 | Years of schooling || id:ieu-a-1239 | 0.001238927 | 950.6205692 | | 42 | rs11681861 | -0.01435 | 0.1565 | 0.2865 | PD || id:ieu-b-7 | Years of schooling || id:ieu-a-1239 | 0.000823105 | 631.3001463 | | 43 | rs11694904 | 0.01215 | 0.3384 | 0.8545 | PD || id:ieu-b-7 | Years of schooling || id:ieu-a-1239 | 0.001151967 | 883.8202049 | | 44 | rs11732657 | -0.01274 | 0.7007 | 0.1970 | PD || id:ieu-b-7 | Years of schooling || id:ieu-a-1239 | 0.001081873 | 829.9834411 | | 45 | rs117468730 | -0.03521 | 0.0119 | 0.2943 | PD || id:ieu-b-7 | Years of schooling || id:ieu-a-1239 | 0.000357259 | 273.880633 | | 46 | rs11752914 | -0.01208 | 0.1973 | 0.8876 | PD || id:ieu-b-7 | Years of schooling || id:ieu-a-1239 | 0.000986802 | 756.9756384 | | 47 | rs11772580 | -0.01199 | 0.2534 | 0.1175 | PD || id:ieu-b-7 | Years of schooling || id:ieu-a-1239 | 0.001060366 | 813.4663577 | | 48 | rs11871429 | -0.01425 | 0.2041 | 0.8505 | PD || id:ieu-b-7 | Years of schooling || id:ieu-a-1239 | 0.001055122 | 809.4392965 | | 49 | rs12028010 | -0.01696 | 0.2228 | 0.6713 | PD || id:ieu-b-7 | Years of schooling || id:ieu-a-1239 | 0.001055122 | 809.4392965 | | 50 | rs12134151 | -0.01245 | 0.5221 | 0.0626 | PD || id:ieu-b-7 | Years of schooling || id:ieu-a-1239 | 0.001253484 | 961.8043406 | | 51 | rs12332731 | 0.01374 | 0.2024 | 0.3836 | PD || id:ieu-b-7 | Years of schooling || id:ieu-a-1239 | 0.000977757 | 750.0309078 | | 52 | rs12375949 | 0.01447 | 0.5697 | 0.9505 | PD || id:ieu-b-7 | Years of schooling || id:ieu-a-1239 | 0.001238927 | 950.6205692 | | 53 | rs12468040 | -0.01432 | 0.6037 | 0.8725 | PD || id:ieu-b-7 | Years of schooling || id:ieu-a-1239 | 0.001217714 | 934.3242166 | | 54 | rs12503522 | -0.01125 | 0.2483 | 0.7036 | PD || id:ieu-b-7 | Years of schooling || id:ieu-a-1239 | 0.001133606 | 869.716691 | | 55 | rs12519073 | -0.01221 | 0.2381 | 0.5538 | PD || id:ieu-b-7 | Years of schooling || id:ieu-a-1239 | 0.001055122 | 809.4392965 | | 56 | rs12574281 | 0.01077 | 0.3997 | 0.5505 | PD || id:ieu-b-7 | Years of schooling || id:ieu-a-1239 | 0.001210803 | 929.0155563 | | 57 | rs12602286 | 0.01701 | 0.8861 | 0.0834 | PD || id:ieu-b-7 | Years of schooling || id:ieu-a-1239 | 0.000836005 | 641.2028937 | | 58 | rs12643771 | 0.01518 | 0.3112 | 0.3394 | PD || id:ieu-b-7 | Years of schooling || id:ieu-a-1239 | 0.001158221 | 888.6235755 | | 59 | rs12682775 | 0.01187 | 0.2143 | 0.8507 | PD || id:ieu-b-7 | Years of schooling || id:ieu-a-1239 | 0.001044788 | 801.5036172 | | 60 | rs12804787 | -0.01814 | 0.0680 | 0.0918 | PD || id:ieu-b-7 | Years of schooling || id:ieu-a-1239 | 0.000652051 | 500.0206052 | | 61 | rs1291818 | -0.01085 | 0.5153 | 0.4368 | PD || id:ieu-b-7 | Years of schooling || id:ieu-a-1239 | 0.001253484 | 961.8043406 | | 62 | rs12940014 | 0.00936 | 0.5204 | 0.4566 | PD || id:ieu-b-7 | Years of schooling || id:ieu-a-1239 | 0.001253484 | 961.8043406 | | 63 | rs12955211 | 0.01097 | 0.3554 | 0.2993 | PD || id:ieu-b-7 | Years of schooling || id:ieu-a-1239 | 0.001170934 | 898.3886698 | | 64 | rs13010566 | 0.0106 | 0.5612 | 0.1307 | PD || id:ieu-b-7 | Years of schooling || id:ieu-a-1239 | 0.001253484 | 961.8043406 | | 65 | rs13029509 | -0.01049 | 0.4677 | 0.7761 | PD || id:ieu-b-7 | Years of schooling || id:ieu-a-1239 | 0.001253484 | 961.8043406 | | 66 | rs13090388 | 0.02852 | 0.3095 | 0.3438 | PD || id:ieu-b-7 | Years of schooling || id:ieu-a-1239 | 0.001158221 | 888.6235755 | | 67 | rs13141210 | 0.01361 | 0.5085 | 0.7431 | PD || id:ieu-b-7 | Years of schooling || id:ieu-a-1239 | 0.001238927 | 950.6205692 | | 68 | rs13145650 | -0.01918 | 0.9082 | 0.9431 | PD || id:ieu-b-7 | Years of schooling || id:ieu-a-1239 | 0.000696768 | 534.3357448 | | 69 | rs1334297 | 0.02449 | 0.7840 | 0.7528 | PD || id:ieu-b-7 | Years of schooling || id:ieu-a-1239 | 0.001110015 | 851.5975932 | | 70 | rs13422673 | -0.01201 | 0.4847 | 0.2492 | PD || id:ieu-b-7 | Years of schooling || id:ieu-a-1239 | 0.001253484 | 961.8043406 | | 71 | rs1363862 | -0.01171 | 0.2602 | 0.7761 | PD || id:ieu-b-7 | Years of schooling || id:ieu-a-1239 | 0.001110015 | 851.5975932 | | 72 | rs1381247 | -0.01013 | 0.2908 | 0.6772 | PD || id:ieu-b-7 | Years of schooling || id:ieu-a-1239 | 0.001170934 | 898.3886698 | | 73 | rs1391438 | -0.0167 | 0.6854 | 0.7452 | PD || id:ieu-b-7 | Years of schooling || id:ieu-a-1239 | 0.001164542 | 893.4794421 | | 74 | rs1427298 | 0.0102 | 0.4116 | 0.7682 | PD || id:ieu-b-7 | Years of schooling || id:ieu-a-1239 | 0.001238927 | 950.6205692 | | 75 | rs1450782 | -0.00945 | 0.6020 | 0.7805 | PD || id:ieu-b-7 | Years of schooling || id:ieu-a-1239 | 0.001231774 | 945.1256526 | | 76 | rs1455350 | -0.01614 | 0.4711 | 0.1228 | PD || id:ieu-b-7 | Years of schooling || id:ieu-a-1239 | 0.001253484 | 961.8043406 | | 77 | rs152603 | 0.01019 | 0.3861 | 0.4753 | PD || id:ieu-b-7 | Years of schooling || id:ieu-a-1239 | 0.001203971 | 923.7668808 | | 78 | rs1558727 | -0.01069 | 0.4847 | 0.3690 | PD || id:ieu-b-7 | Years of schooling || id:ieu-a-1239 | 0.001253484 | 961.8043406 | | 79 | rs1566085 | 0.01645 | 0.5697 | 0.7715 | PD || id:ieu-b-7 | Years of schooling || id:ieu-a-1239 | 0.001246163 | 956.1797538 | | 80 | rs1569092 | 0.01807 | 0.1820 | 0.2949 | PD || id:ieu-b-7 | Years of schooling || id:ieu-a-1239 | 0.000910963 | 698.7467432 | | 81 | rs1584469 | -0.01303 | 0.3112 | 0.8430 | PD || id:ieu-b-7 | Years of schooling || id:ieu-a-1239 | 0.001151967 | 883.8202049 | | 82 | rs1592757 | -0.01045 | 0.3759 | 0.2155 | PD || id:ieu-b-7 | Years of schooling || id:ieu-a-1239 | 0.001183929 | 908.3707661 | | 83 | rs1595973 | -0.01002 | 0.5595 | 0.0908 | PD || id:ieu-b-7 | Years of schooling || id:ieu-a-1239 | 0.001231774 | 945.1256526 | | 84 | rs1620977 | -0.02046 | 0.6905 | 0.6638 | PD || id:ieu-b-7 | Years of schooling || id:ieu-a-1239 | 0.001092957 | 838.4960918 | | 85 | rs1671770 | -0.01342 | 0.8061 | 0.9014 | PD || id:ieu-b-7 | Years of schooling || id:ieu-a-1239 | 0.000955856 | 733.2140713 | | 86 | rs16846463 | -0.02256 | 0.1173 | 0.4515 | PD || id:ieu-b-7 | Years of schooling || id:ieu-a-1239 | 0.000753353 | 577.7623247 | | 87 | rs16854920 | 0.01007 | 0.3554 | 0.0564 | PD || id:ieu-b-7 | Years of schooling || id:ieu-a-1239 | 0.001177395 | 903.3521431 | | 88 | rs1689510 | 0.01761 | 0.3435 | 0.5245 | PD || id:ieu-b-7 | Years of schooling || id:ieu-a-1239 | 0.001183929 | 908.3707661 | | 89 | rs16995054 | -0.0139 | 0.2007 | 0.4656 | PD || id:ieu-b-7 | Years of schooling || id:ieu-a-1239 | 0.001024717 | 786.0900861 | | 90 | rs17048855 | 0.01184 | 0.3248 | 0.9413 | PD || id:ieu-b-7 | Years of schooling || id:ieu-a-1239 | 0.001190535 | 913.4454631 | | 91 | rs17110109 | 0.01023 | 0.3776 | 0.8892 | PD || id:ieu-b-7 | Years of schooling || id:ieu-a-1239 | 0.001217714 | 934.3242166 | | 92 | rs17126938 | 0.01536 | 0.1207 | 0.0885 | PD || id:ieu-b-7 | Years of schooling || id:ieu-a-1239 | 0.000852711 | 654.0269516 | | 93 | rs17425572 | -0.01224 | 0.5425 | 0.8711 | PD || id:ieu-b-7 | Years of schooling || id:ieu-a-1239 | 0.001253484 | 961.8043406 | | 94 | rs17489649 | -0.0139 | 0.3265 | 0.5313 | PD || id:ieu-b-7 | Years of schooling || id:ieu-a-1239 | 0.001177395 | 903.3521431 | | 95 | rs175325 | -0.01179 | 0.5816 | 0.9401 | PD || id:ieu-b-7 | Years of schooling || id:ieu-a-1239 | 0.001224704 | 939.693896 | | 96 | rs17563464 | -0.01477 | 0.2041 | 0.3637 | PD || id:ieu-b-7 | Years of schooling || id:ieu-a-1239 | 0.001005402 | 771.2581976 | | 97 | rs17565975 | -0.01142 | 0.5306 | 0.0799 | PD || id:ieu-b-7 | Years of schooling || id:ieu-a-1239 | 0.001246163 | 956.1797538 | | 98 | rs17598675 | 0.01199 | 0.5187 | 0.2469 | PD || id:ieu-b-7 | Years of schooling || id:ieu-a-1239 | 0.001253484 | 961.8043406 | | 99 | rs176218 | 0.01883 | 0.2007 | 0.0133 | PD || id:ieu-b-7 | Years of schooling || id:ieu-a-1239 | 0.000991387 | 760.4964554 | | 100 | rs1827540 | -0.0106 | 0.4660 | 0.3922 | PD || id:ieu-b-7 | Years of schooling || id:ieu-a-1239 | 0.001253484 | 961.8043406 | | 101 | rs1866823 | 0.01009 | 0.5510 | 0.2884 | PD || id:ieu-b-7 | Years of schooling || id:ieu-a-1239 | 0.001246163 | 956.1797538 | | 102 | rs1882273 | -0.01231 | 0.3469 | 0.3501 | PD || id:ieu-b-7 | Years of schooling || id:ieu-a-1239 | 0.001177395 | 903.3521431 | | 103 | rs192436652 | -0.03497 | 0.0221 | 0.1805 | PD || id:ieu-b-7 | Years of schooling || id:ieu-a-1239 | 0.000391333 | 300.0123631 | | 104 | rs1925576 | 0.00997 | 0.4422 | 0.7697 | PD || id:ieu-b-7 | Years of schooling || id:ieu-a-1239 | 0.001246163 | 956.1797538 | | 105 | rs1947114 | 0.01071 | 0.2551 | 0.2418 | PD || id:ieu-b-7 | Years of schooling || id:ieu-a-1239 | 0.001110015 | 851.5975932 | | 106 | rs1949226 | -0.00965 | 0.5901 | 0.8879 | PD || id:ieu-b-7 | Years of schooling || id:ieu-a-1239 | 0.001224704 | 939.693896 | | 107 | rs1964927 | -0.01423 | 0.6378 | 0.5384 | PD || id:ieu-b-7 | Years of schooling || id:ieu-a-1239 | 0.001203971 | 923.7668808 | | 108 | rs2179152 | 0.01455 | 0.6429 | 0.2747 | PD || id:ieu-b-7 | Years of schooling || id:ieu-a-1239 | 0.001210803 | 929.0155563 | | 109 | rs2256965 | -0.01128 | 0.5425 | 0.6935 | PD || id:ieu-b-7 | Years of schooling || id:ieu-a-1239 | 0.001210803 | 929.0155563 | | 110 | rs2283076 | -0.01143 | 0.2143 | 0.2381 | PD || id:ieu-b-7 | Years of schooling || id:ieu-a-1239 | 0.001044788 | 801.5036172 | | 111 | rs2287838 | -0.01152 | 0.5340 | 0.0042 | PD || id:ieu-b-7 | Years of schooling || id:ieu-a-1239 | 0.001246163 | 956.1797538 | | 112 | rs2302761 | 0.01354 | 0.1905 | 0.0063 | PD || id:ieu-b-7 | Years of schooling || id:ieu-a-1239 | 0.001019819 | 782.3288895 | | 113 | rs2347526 | 0.01395 | 0.6378 | 0.7840 | PD || id:ieu-b-7 | Years of schooling || id:ieu-a-1239 | 0.001190535 | 913.4454631 | | 114 | rs2414072 | -0.01005 | 0.4864 | 0.0271 | PD || id:ieu-b-7 | Years of schooling || id:ieu-a-1239 | 0.001246163 | 956.1797538 | | 115 | rs242093 | -0.01031 | 0.5476 | 0.7198 | PD || id:ieu-b-7 | Years of schooling || id:ieu-a-1239 | 0.001238927 | 950.6205692 | | 116 | rs2441111 | 0.01087 | 0.5323 | 0.8990 | PD || id:ieu-b-7 | Years of schooling || id:ieu-a-1239 | 0.001253484 | 961.8043406 | | 117 | rs2447535 | 0.01181 | 0.7245 | 0.3390 | PD || id:ieu-b-7 | Years of schooling || id:ieu-a-1239 | 0.001151967 | 883.8202049 | | 118 | rs2478208 | -0.0106 | 0.5000 | 0.8458 | PD || id:ieu-b-7 | Years of schooling || id:ieu-a-1239 | 0.001253484 | 961.8043406 | | 119 | rs2545798 | -0.01346 | 0.4949 | 0.1599 | PD || id:ieu-b-7 | Years of schooling || id:ieu-a-1239 | 0.001246163 | 956.1797538 | | 120 | rs2554835 | 0.00974 | 0.3980 | 0.5714 | PD || id:ieu-b-7 | Years of schooling || id:ieu-a-1239 | 0.001217714 | 934.3242166 | | 121 | rs2570497 | -0.01233 | 0.6735 | 0.7529 | PD || id:ieu-b-7 | Years of schooling || id:ieu-a-1239 | 0.001203971 | 923.7668808 | | 122 | rs2725370 | 0.01536 | 0.7109 | 0.9191 | PD || id:ieu-b-7 | Years of schooling || id:ieu-a-1239 | 0.001139661 | 874.3675824 | | 123 | rs277828 | -0.01091 | 0.2568 | 0.4768 | PD || id:ieu-b-7 | Years of schooling || id:ieu-a-1239 | 0.001087386 | 834.2180505 | | 124 | rs2787101 | 0.00968 | 0.6173 | 0.0281 | PD || id:ieu-b-7 | Years of schooling || id:ieu-a-1239 | 0.001224704 | 939.693896 | | 125 | rs2819336 | -0.01828 | 0.6616 | 0.0902 | PD || id:ieu-b-7 | Years of schooling || id:ieu-a-1239 | 0.001203971 | 923.7668808 | | 126 | rs2820314 | -0.011 | 0.3163 | 0.1291 | PD || id:ieu-b-7 | Years of schooling || id:ieu-a-1239 | 0.001183929 | 908.3707661 | | 127 | rs28373063 | 0.01389 | 0.2075 | 0.1820 | PD || id:ieu-b-7 | Years of schooling || id:ieu-a-1239 | 0.000930835 | 714.0032223 | | 128 | rs28513670 | 0.01477 | 0.1531 | 0.7237 | PD || id:ieu-b-7 | Years of schooling || id:ieu-a-1239 | 0.000947367 | 726.6966129 | | 129 | rs2885198 | -0.01025 | 0.5017 | 0.6855 | PD || id:ieu-b-7 | Years of schooling || id:ieu-a-1239 | 0.001253484 | 961.8043406 | | 130 | rs2923431 | 0.0114 | 0.6446 | 0.7330 | PD || id:ieu-b-7 | Years of schooling || id:ieu-a-1239 | 0.001210803 | 929.0155563 | | 131 | rs2971970 | 0.01654 | 0.7721 | 0.6260 | PD || id:ieu-b-7 | Years of schooling || id:ieu-a-1239 | 0.001029662 | 789.8876227 | | 132 | rs2998315 | 0.01269 | 0.5782 | 0.6252 | PD || id:ieu-b-7 | Years of schooling || id:ieu-a-1239 | 0.001246163 | 956.1797538 | | 133 | rs3013014 | -0.01024 | 0.6105 | 0.0239 | PD || id:ieu-b-7 | Years of schooling || id:ieu-a-1239 | 0.001238927 | 950.6205692 | | 134 | rs301800 | -0.01516 | 0.8197 | 0.7413 | PD || id:ieu-b-7 | Years of schooling || id:ieu-a-1239 | 0.000951592 | 729.9407942 | | 135 | rs3026996 | -0.01537 | 0.2840 | 0.9676 | PD || id:ieu-b-7 | Years of schooling || id:ieu-a-1239 | 0.001071011 | 821.641899 | | 136 | rs320693 | 0.01204 | 0.4728 | 0.7556 | PD || id:ieu-b-7 | Years of schooling || id:ieu-a-1239 | 0.001253484 | 961.8043406 | | 137 | rs337637 | 0.01123 | 0.3367 | 0.5682 | PD || id:ieu-b-7 | Years of schooling || id:ieu-a-1239 | 0.001203971 | 923.7668808 | | 138 | rs34316 | -0.02016 | 0.5799 | 0.4693 | PD || id:ieu-b-7 | Years of schooling || id:ieu-a-1239 | 0.001203971 | 923.7668808 | | 139 | rs34316 | -0.02016 | 0.5799 | 0.4693 | PD || id:ieu-b-7 | Years of schooling || id:ieu-a-1239 | 0.001203971 | 923.7668808 | | 140 | rs34485537 | 0.01075 | 0.3895 | 0.1942 | PD || id:ieu-b-7 | Years of schooling || id:ieu-a-1239 | 0.001231774 | 945.1256526 | | 141 | rs34853711 | -0.01596 | 0.2466 | 0.2194 | PD || id:ieu-b-7 | Years of schooling || id:ieu-a-1239 | 0.001034655 | 793.7220286 | | 142 | rs35039375 | -0.01983 | 0.0935 | 0.6525 | PD || id:ieu-b-7 | Years of schooling || id:ieu-a-1239 | 0.00072766 | 558.0434741 | | 143 | rs35309068 | 0.01321 | 0.4660 | 0.8534 | PD || id:ieu-b-7 | Years of schooling || id:ieu-a-1239 | 0.001246163 | 956.1797538 | | 144 | rs35316276 | 0.01173 | 0.2942 | 0.5158 | PD || id:ieu-b-7 | Years of schooling || id:ieu-a-1239 | 0.001098584 | 842.8182366 | | 145 | rs35417702 | -0.01445 | 0.5765 | 0.6986 | PD || id:ieu-b-7 | Years of schooling || id:ieu-a-1239 | 0.001253484 | 961.8043406 | | 146 | rs35475880 | -0.01511 | 0.1837 | 0.6906 | PD || id:ieu-b-7 | Years of schooling || id:ieu-a-1239 | 0.001024717 | 786.0900861 | | 147 | rs35532491 | 0.02007 | 0.1071 | 0.6508 | PD || id:ieu-b-7 | Years of schooling || id:ieu-a-1239 | 0.000745457 | 571.7018808 | | 148 | rs36083520 | 0.01629 | 0.1667 | 0.0948 | PD || id:ieu-b-7 | Years of schooling || id:ieu-a-1239 | 0.000955856 | 733.2140713 | | 149 | rs36119825 | 0.01063 | 0.4694 | 0.8806 | PD || id:ieu-b-7 | Years of schooling || id:ieu-a-1239 | 0.001246163 | 956.1797538 | | 150 | rs363096 | 0.01363 | 0.5748 | 0.1503 | PD || id:ieu-b-7 | Years of schooling || id:ieu-a-1239 | 0.001238927 | 950.6205692 | | 151 | rs3747631 | 0.02207 | 0.2279 | 0.6158 | PD || id:ieu-b-7 | Years of schooling || id:ieu-a-1239 | 0.001024717 | 786.0900861 | | 152 | rs3788556 | -0.01138 | 0.5408 | 0.4150 | PD || id:ieu-b-7 | Years of schooling || id:ieu-a-1239 | 0.001246163 | 956.1797538 | | 153 | rs3800546 | -0.01183 | 0.2704 | 0.5281 | PD || id:ieu-b-7 | Years of schooling || id:ieu-a-1239 | 0.001098584 | 842.8182366 | | 154 | rs3809634 | 0.01058 | 0.3350 | 0.7312 | PD || id:ieu-b-7 | Years of schooling || id:ieu-a-1239 | 0.001151967 | 883.8202049 | | 155 | rs3890802 | -0.01133 | 0.2687 | 0.4883 | PD || id:ieu-b-7 | Years of schooling || id:ieu-a-1239 | 0.00111582 | 856.0562194 | | 156 | rs3897821 | -0.01502 | 0.3503 | 0.5028 | PD || id:ieu-b-7 | Years of schooling || id:ieu-a-1239 | 0.001183929 | 908.3707661 | | 157 | rs401687 | 0.01144 | 0.4558 | 0.8213 | PD || id:ieu-b-7 | Years of schooling || id:ieu-a-1239 | 0.001253484 | 961.8043406 | | 158 | rs406413 | -0.01695 | 0.2109 | 0.0087 | PD || id:ieu-b-7 | Years of schooling || id:ieu-a-1239 | 0.001019819 | 782.3288895 | | 159 | rs4073894 | 0.01524 | 0.1769 | 0.2239 | PD || id:ieu-b-7 | Years of schooling || id:ieu-a-1239 | 0.001010162 | 774.9134498 | | 160 | rs4328757 | 0.01067 | 0.6514 | 0.5888 | PD || id:ieu-b-7 | Years of schooling || id:ieu-a-1239 | 0.001224704 | 939.693896 | | 161 | rs4352658 | -0.0212 | 0.0901 | 0.1607 | PD || id:ieu-b-7 | Years of schooling || id:ieu-a-1239 | 0.000692247 | 530.8660321 | | 162 | rs4352658 | -0.0212 | 0.0901 | 0.1607 | PD || id:ieu-b-7 | Years of schooling || id:ieu-a-1239 | 0.000692247 | 530.8660321 | | 163 | rs4369924 | 0.01362 | 0.1684 | 0.7993 | PD || id:ieu-b-7 | Years of schooling || id:ieu-a-1239 | 0.000910963 | 698.7467432 | | 164 | rs4382592 | 0.01636 | 0.6990 | 0.1901 | PD || id:ieu-b-7 | Years of schooling || id:ieu-a-1239 | 0.001151967 | 883.8202049 | | 165 | rs4384309 | 0.0109 | 0.4796 | 0.0104 | PD || id:ieu-b-7 | Years of schooling || id:ieu-a-1239 | 0.001238927 | 950.6205692 | | 166 | rs4392737 | -0.0097 | 0.3827 | 0.7646 | PD || id:ieu-b-7 | Years of schooling || id:ieu-a-1239 | 0.001231774 | 945.1256526 | | 167 | rs4442732 | -0.01063 | 0.5969 | 0.8691 | PD || id:ieu-b-7 | Years of schooling || id:ieu-a-1239 | 0.001210803 | 929.0155563 | | 168 | rs4497562 | -0.01204 | 0.2823 | 0.6368 | PD || id:ieu-b-7 | Years of schooling || id:ieu-a-1239 | 0.001110015 | 851.5975932 | | 169 | rs4667025 | 0.00957 | 0.3878 | 0.7793 | PD || id:ieu-b-7 | Years of schooling || id:ieu-a-1239 | 0.001224704 | 939.693896 | | 170 | rs4700393 | 0.02086 | 0.5289 | 0.5859 | PD || id:ieu-b-7 | Years of schooling || id:ieu-a-1239 | 0.001253484 | 961.8043406 | | 171 | rs4743923 | 0.00985 | 0.3673 | 0.0771 | PD || id:ieu-b-7 | Years of schooling || id:ieu-a-1239 | 0.001210803 | 929.0155563 | | 172 | rs4757957 | 0.0141 | 0.6514 | 0.7081 | PD || id:ieu-b-7 | Years of schooling || id:ieu-a-1239 | 0.001158221 | 888.6235755 | | 173 | rs4766424 | -0.0141 | 0.9116 | 0.3604 | PD || id:ieu-b-7 | Years of schooling || id:ieu-a-1239 | 0.000826292 | 633.7470461 | | 174 | rs4778058 | 0.01017 | 0.5221 | 0.7344 | PD || id:ieu-b-7 | Years of schooling || id:ieu-a-1239 | 0.001253484 | 961.8043406 | | 175 | rs4787457 | -0.01741 | 0.3146 | 0.1376 | PD || id:ieu-b-7 | Years of schooling || id:ieu-a-1239 | 0.001210803 | 929.0155563 | | 176 | rs4810227 | 0.01272 | 0.6344 | 0.3038 | PD || id:ieu-b-7 | Years of schooling || id:ieu-a-1239 | 0.001217714 | 934.3242166 | | 177 | rs4839155 | -0.01251 | 0.2500 | 0.9126 | PD || id:ieu-b-7 | Years of schooling || id:ieu-a-1239 | 0.001065662 | 817.5336895 | | 178 | rs4846724 | 0.01018 | 0.4915 | 0.2507 | PD || id:ieu-b-7 | Years of schooling || id:ieu-a-1239 | 0.001253484 | 961.8043406 | | 179 | rs4870482 | -0.01083 | 0.2585 | 0.6445 | PD || id:ieu-b-7 | Years of schooling || id:ieu-a-1239 | 0.001121686 | 860.5617784 | | 180 | rs4888746 | -0.00952 | 0.3776 | 0.8884 | PD || id:ieu-b-7 | Years of schooling || id:ieu-a-1239 | 0.001224704 | 939.693896 | | 181 | rs4904523 | -0.00936 | 0.4592 | 0.5276 | PD || id:ieu-b-7 | Years of schooling || id:ieu-a-1239 | 0.001253484 | 961.8043406 | | 182 | rs4945424 | -0.00992 | 0.4150 | 0.5246 | PD || id:ieu-b-7 | Years of schooling || id:ieu-a-1239 | 0.001246163 | 956.1797538 | | 183 | rs4964046 | 0.01053 | 0.3350 | 0.1085 | PD || id:ieu-b-7 | Years of schooling || id:ieu-a-1239 | 0.001197215 | 918.5771792 | | 184 | rs4972400 | 0.01156 | 0.3520 | 0.5754 | PD || id:ieu-b-7 | Years of schooling || id:ieu-a-1239 | 0.001177395 | 903.3521431 | | 185 | rs4984541 | 0.01233 | 0.2415 | 0.6942 | PD || id:ieu-b-7 | Years of schooling || id:ieu-a-1239 | 0.001029662 | 789.8876227 | | 186 | rs510706 | 0.01071 | 0.6156 | 0.3333 | PD || id:ieu-b-7 | Years of schooling || id:ieu-a-1239 | 0.001183929 | 908.3707661 | | 187 | rs535307 | -0.01004 | 0.6769 | 0.4358 | PD || id:ieu-b-7 | Years of schooling || id:ieu-a-1239 | 0.001158221 | 888.6235755 | | 188 | rs55736314 | 0.01431 | 0.4167 | 0.6723 | PD || id:ieu-b-7 | Years of schooling || id:ieu-a-1239 | 0.001224704 | 939.693896 | | 189 | rs56391344 | 0.01571 | 0.2381 | 0.8060 | PD || id:ieu-b-7 | Years of schooling || id:ieu-a-1239 | 0.001081873 | 829.9834411 | | 190 | rs575113 | 0.01285 | 0.2772 | 0.9463 | PD || id:ieu-b-7 | Years of schooling || id:ieu-a-1239 | 0.001145781 | 879.0684833 | | 191 | rs59123361 | -0.02094 | 0.1054 | 0.8148 | PD || id:ieu-b-7 | Years of schooling || id:ieu-a-1239 | 0.000732658 | 561.8788244 | | 192 | rs59480703 | -0.01237 | 0.1633 | 0.9341 | PD || id:ieu-b-7 | Years of schooling || id:ieu-a-1239 | 0.000991387 | 760.4964554 | | 193 | rs6122735 | 0.0105 | 0.4133 | 0.0247 | PD || id:ieu-b-7 | Years of schooling || id:ieu-a-1239 | 0.001224704 | 939.693896 | | 194 | rs6123924 | -0.01528 | 0.1599 | 0.0237 | PD || id:ieu-b-7 | Years of schooling || id:ieu-a-1239 | 0.00090709 | 695.7733528 | | 195 | rs613872 | -0.0175 | 0.8282 | 0.2640 | PD || id:ieu-b-7 | Years of schooling || id:ieu-a-1239 | 0.000939028 | 720.2939996 | | 196 | rs61747885 | 0.01383 | 0.1412 | 0.7688 | PD || id:ieu-b-7 | Years of schooling || id:ieu-a-1239 | 0.00090709 | 695.7733528 | | 197 | rs62097985 | -0.01288 | 0.4116 | 0.2500 | PD || id:ieu-b-7 | Years of schooling || id:ieu-a-1239 | 0.001238927 | 950.6205692 | | 198 | rs62157915 | 0.02091 | 0.0595 | 0.4516 | PD || id:ieu-b-7 | Years of schooling || id:ieu-a-1239 | 0.000612727 | 469.846948 | | 199 | rs62183776 | -0.01308 | 0.1905 | 0.4236 | PD || id:ieu-b-7 | Years of schooling || id:ieu-a-1239 | 0.000982259 | 753.4872714 | | 200 | rs62184480 | -0.01528 | 0.2449 | 0.0261 | PD || id:ieu-b-7 | Years of schooling || id:ieu-a-1239 | 0.00111582 | 856.0562194 | | 201 | rs622169 | 0.00999 | 0.4677 | 0.1811 | PD || id:ieu-b-7 | Years of schooling || id:ieu-a-1239 | 0.001197215 | 918.5771792 | | 202 | rs62439690 | -0.01087 | 0.2670 | 0.0891 | PD || id:ieu-b-7 | Years of schooling || id:ieu-a-1239 | 0.001098584 | 842.8182366 | | 203 | rs62444881 | 0.01815 | 0.1905 | 0.6323 | PD || id:ieu-b-7 | Years of schooling || id:ieu-a-1239 | 0.000982259 | 753.4872714 | | 204 | rs6493265 | -0.01385 | 0.3895 | 0.0957 | PD || id:ieu-b-7 | Years of schooling || id:ieu-a-1239 | 0.001224704 | 939.693896 | | 205 | rs6513959 | -0.01177 | 0.2789 | 0.4628 | PD || id:ieu-b-7 | Years of schooling || id:ieu-a-1239 | 0.001151967 | 883.8202049 | | 206 | rs6557171 | 0.01567 | 0.7245 | 0.5827 | PD || id:ieu-b-7 | Years of schooling || id:ieu-a-1239 | 0.001177395 | 903.3521431 | | 207 | rs663234 | -0.01005 | 0.6122 | 0.2226 | PD || id:ieu-b-7 | Years of schooling || id:ieu-a-1239 | 0.001224704 | 939.693896 | | 208 | rs66568921 | 0.01565 | 0.3639 | 0.6803 | PD || id:ieu-b-7 | Years of schooling || id:ieu-a-1239 | 0.001170934 | 898.3886698 | | 209 | rs66568921 | 0.01565 | 0.3639 | 0.6803 | PD || id:ieu-b-7 | Years of schooling || id:ieu-a-1239 | 0.001170934 | 898.3886698 | | 210 | rs6731373 | -0.01256 | 0.3367 | 0.9111 | PD || id:ieu-b-7 | Years of schooling || id:ieu-a-1239 | 0.001177395 | 903.3521431 | | 211 | rs6731967 | -0.01186 | 0.2092 | 0.7698 | PD || id:ieu-b-7 | Years of schooling || id:ieu-a-1239 | 0.001071011 | 821.641899 | | 212 | rs67885444 | 0.01406 | 0.1718 | 0.0562 | PD || id:ieu-b-7 | Years of schooling || id:ieu-a-1239 | 0.000918809 | 704.770422 | | 213 | rs67890737 | -0.01141 | 0.3265 | 0.2845 | PD || id:ieu-b-7 | Years of schooling || id:ieu-a-1239 | 0.001190535 | 913.4454631 | | 214 | rs6803651 | 0.01131 | 0.4150 | 0.6018 | PD || id:ieu-b-7 | Years of schooling || id:ieu-a-1239 | 0.001238927 | 950.6205692 | | 215 | rs6805241 | -0.01413 | 0.1973 | 0.4123 | PD || id:ieu-b-7 | Years of schooling || id:ieu-a-1239 | 0.00104993 | 805.4519108 | | 216 | rs6867851 | -0.012 | 0.4235 | 0.6816 | PD || id:ieu-b-7 | Years of schooling || id:ieu-a-1239 | 0.001231774 | 945.1256526 | | 217 | rs6938002 | -0.01008 | 0.3963 | 0.4430 | PD || id:ieu-b-7 | Years of schooling || id:ieu-a-1239 | 0.001231774 | 945.1256526 | | 218 | rs6959891 | -0.01136 | 0.2959 | 0.5493 | PD || id:ieu-b-7 | Years of schooling || id:ieu-a-1239 | 0.001127615 | 865.1150153 | | 219 | rs7012546 | 0.01009 | 0.4201 | 0.8251 | PD || id:ieu-b-7 | Years of schooling || id:ieu-a-1239 | 0.001238927 | 950.6205692 | | 220 | rs7016302 | 0.01243 | 0.1769 | 0.3670 | PD || id:ieu-b-7 | Years of schooling || id:ieu-a-1239 | 0.000934913 | 717.1348154 | | 221 | rs702606 | -0.01427 | 0.1701 | 0.6206 | PD || id:ieu-b-7 | Years of schooling || id:ieu-a-1239 | 0.000852711 | 654.0269516 | | 222 | rs7029718 | 0.02439 | 0.4354 | 0.4727 | PD || id:ieu-b-7 | Years of schooling || id:ieu-a-1239 | 0.001224704 | 939.693896 | | 223 | rs7031698 | 0.01248 | 0.7755 | 0.6465 | PD || id:ieu-b-7 | Years of schooling || id:ieu-a-1239 | 0.001034655 | 793.7220286 | | 224 | rs710629 | 0.01053 | 0.6565 | 0.7027 | PD || id:ieu-b-7 | Years of schooling || id:ieu-a-1239 | 0.001203971 | 923.7668808 | | 225 | rs71646142 | 0.01286 | 0.1735 | 0.1767 | PD || id:ieu-b-7 | Years of schooling || id:ieu-a-1239 | 0.000982259 | 753.4872714 | | 226 | rs7233920 | -0.01315 | 0.2160 | 0.5635 | PD || id:ieu-b-7 | Years of schooling || id:ieu-a-1239 | 0.001055122 | 809.4392965 | | 227 | rs7257460 | -0.01145 | 0.2704 | 0.2012 | PD || id:ieu-b-7 | Years of schooling || id:ieu-a-1239 | 0.001127615 | 865.1150153 | | 228 | rs7278859 | 0.01013 | 0.3078 | 0.3920 | PD || id:ieu-b-7 | Years of schooling || id:ieu-a-1239 | 0.001151967 | 883.8202049 | | 229 | rs72807818 | 0.01915 | 0.1241 | 0.2613 | PD || id:ieu-b-7 | Years of schooling || id:ieu-a-1239 | 0.000845949 | 648.8362615 | | 230 | rs72807818 | 0.01915 | 0.1241 | 0.2613 | PD || id:ieu-b-7 | Years of schooling || id:ieu-a-1239 | 0.000845949 | 648.8362615 | | 231 | rs72828517 | 0.01836 | 0.1412 | 0.6419 | PD || id:ieu-b-7 | Years of schooling || id:ieu-a-1239 | 0.000951592 | 729.9407942 | | 232 | rs72840994 | 0.01247 | 0.1820 | 0.1781 | PD || id:ieu-b-7 | Years of schooling || id:ieu-a-1239 | 0.000986802 | 756.9756384 | | 233 | rs730384 | 0.01016 | 0.4558 | 0.7548 | PD || id:ieu-b-7 | Years of schooling || id:ieu-a-1239 | 0.001246163 | 956.1797538 | | 234 | rs73301698 | -0.01291 | 0.2262 | 0.9600 | PD || id:ieu-b-7 | Years of schooling || id:ieu-a-1239 | 0.001024717 | 786.0900861 | | 235 | rs7332724 | -0.01149 | 0.2619 | 0.4165 | PD || id:ieu-b-7 | Years of schooling || id:ieu-a-1239 | 0.001127615 | 865.1150153 | | 236 | rs73344830 | -0.0172 | 0.6020 | 0.6333 | PD || id:ieu-b-7 | Years of schooling || id:ieu-a-1239 | 0.001238927 | 950.6205692 | | 237 | rs736282 | -0.01082 | 0.5153 | 0.4573 | PD || id:ieu-b-7 | Years of schooling || id:ieu-a-1239 | 0.001253484 | 961.8043406 | | 238 | rs73874335 | -0.0199 | 0.0595 | 0.5598 | PD || id:ieu-b-7 | Years of schooling || id:ieu-a-1239 | 0.000590675 | 452.9272518 | | 239 | rs743316 | -0.01185 | 0.1820 | 0.4646 | PD || id:ieu-b-7 | Years of schooling || id:ieu-a-1239 | 0.001024717 | 786.0900861 | | 240 | rs74643044 | 0.02323 | 0.0272 | 0.0679 | PD || id:ieu-b-7 | Years of schooling || id:ieu-a-1239 | 0.00055244 | 423.5925852 | | 241 | rs74701752 | 0.01591 | 0.0952 | 0.4164 | PD || id:ieu-b-7 | Years of schooling || id:ieu-a-1239 | 0.000748071 | 573.7078523 | | 242 | rs7481514 | 0.01072 | 0.6650 | 0.6301 | PD || id:ieu-b-7 | Years of schooling || id:ieu-a-1239 | 0.001197215 | 918.5771792 | | 243 | rs7594904 | 0.00969 | 0.4184 | 0.8613 | PD || id:ieu-b-7 | Years of schooling || id:ieu-a-1239 | 0.001231774 | 945.1256526 | | 244 | rs7603132 | 0.01317 | 0.1548 | 0.8137 | PD || id:ieu-b-7 | Years of schooling || id:ieu-a-1239 | 0.000991387 | 760.4964554 | | 245 | rs76076331 | 0.01873 | 0.1310 | 0.7167 | PD || id:ieu-b-7 | Years of schooling || id:ieu-a-1239 | 0.000859582 | 659.3013625 | | 246 | rs7650602 | 0.00939 | 0.4286 | 0.9523 | PD || id:ieu-b-7 | Years of schooling || id:ieu-a-1239 | 0.001246163 | 956.1797538 | | 247 | rs76608582 | 0.02798 | 0.0408 | 0.1426 | PD || id:ieu-b-7 | Years of schooling || id:ieu-a-1239 | 0.00047923 | 367.4308717 | | 248 | rs76878669 | -0.01399 | 0.2534 | 0.3836 | PD || id:ieu-b-7 | Years of schooling || id:ieu-a-1239 | 0.001039697 | 797.5938434 | | 249 | rs77025239 | -0.01422 | 0.1088 | 0.8073 | PD || id:ieu-b-7 | Years of schooling || id:ieu-a-1239 | 0.000910963 | 698.7467432 | | 250 | rs77128898 | -0.02769 | 0.0221 | 0.6682 | PD || id:ieu-b-7 | Years of schooling || id:ieu-a-1239 | 0.000442459 | 339.2255973 | | 251 | rs77702622 | -0.02447 | 0.0765 | 0.2186 | PD || id:ieu-b-7 | Years of schooling || id:ieu-a-1239 | 0.000607493 | 465.8311621 | | 252 | rs77719387 | -0.04597 | 0.0170 | 0.3225 | PD || id:ieu-b-7 | Years of schooling || id:ieu-a-1239 | 0.000293798 | 225.2158924 | | 253 | rs77835879 | -0.01601 | 0.0901 | 0.7302 | PD || id:ieu-b-7 | Years of schooling || id:ieu-a-1239 | 0.000740284 | 567.7317288 | | 254 | rs7796203 | -0.01074 | 0.5255 | 0.2531 | PD || id:ieu-b-7 | Years of schooling || id:ieu-a-1239 | 0.001246163 | 956.1797538 | | 255 | rs7803932 | 0.0143 | 0.1565 | 0.3094 | PD || id:ieu-b-7 | Years of schooling || id:ieu-a-1239 | 0.000943179 | 723.4811412 | | 256 | rs7808399 | 0.0107 | 0.5476 | 0.4665 | PD || id:ieu-b-7 | Years of schooling || id:ieu-a-1239 | 0.001246163 | 956.1797538 | | 257 | rs7833201 | -0.01532 | 0.1344 | 0.9206 | PD || id:ieu-b-7 | Years of schooling || id:ieu-a-1239 | 0.000813687 | 624.0715187 | | 258 | rs7863447 | 0.01678 | 0.8333 | 0.8918 | PD || id:ieu-b-7 | Years of schooling || id:ieu-a-1239 | 0.000914869 | 701.7456562 | | 259 | rs78721320 | 0.01307 | 0.2041 | 0.7001 | PD || id:ieu-b-7 | Years of schooling || id:ieu-a-1239 | 0.000973297 | 746.6061091 | | 260 | rs790647 | -0.01482 | 0.2347 | 0.4060 | PD || id:ieu-b-7 | Years of schooling || id:ieu-a-1239 | 0.001055122 | 809.4392965 | | 261 | rs7920624 | -0.01181 | 0.4932 | 0.7439 | PD || id:ieu-b-7 | Years of schooling || id:ieu-a-1239 | 0.001253484 | 961.8043406 | | 262 | rs7924036 | 0.01501 | 0.5391 | 0.9145 | PD || id:ieu-b-7 | Years of schooling || id:ieu-a-1239 | 0.001253484 | 961.8043406 | | 263 | rs79265434 | 0.02331 | 0.1173 | 0.6251 | PD || id:ieu-b-7 | Years of schooling || id:ieu-a-1239 | 0.000813687 | 624.0715187 | | 264 | rs79269403 | 0.01447 | 0.2228 | 0.3437 | PD || id:ieu-b-7 | Years of schooling || id:ieu-a-1239 | 0.001044788 | 801.5036172 | | 265 | rs7928622 | 0.01011 | 0.3078 | 0.8431 | PD || id:ieu-b-7 | Years of schooling || id:ieu-a-1239 | 0.001177395 | 903.3521431 | | 266 | rs795230 | 0.00952 | 0.4184 | 0.7897 | PD || id:ieu-b-7 | Years of schooling || id:ieu-a-1239 | 0.001238927 | 950.6205692 | | 267 | rs79523955 | -0.01802 | 0.0952 | 0.8800 | PD || id:ieu-b-7 | Years of schooling || id:ieu-a-1239 | 0.000753353 | 577.7623247 | | 268 | rs7977614 | 0.01325 | 0.3078 | 0.5968 | PD || id:ieu-b-7 | Years of schooling || id:ieu-a-1239 | 0.001076414 | 825.7916056 | | 269 | rs7993663 | 0.0118 | 0.3571 | 0.2048 | PD || id:ieu-b-7 | Years of schooling || id:ieu-a-1239 | 0.001197215 | 918.5771792 | | 270 | rs8008382 | 0.01208 | 0.6871 | 0.9926 | PD || id:ieu-b-7 | Years of schooling || id:ieu-a-1239 | 0.001151967 | 883.8202049 | | 271 | rs80171383 | 0.0145 | 0.1241 | 0.9094 | PD || id:ieu-b-7 | Years of schooling || id:ieu-a-1239 | 0.000884527 | 678.4511946 | | 272 | rs8020034 | 0.01782 | 0.2058 | 0.9583 | PD || id:ieu-b-7 | Years of schooling || id:ieu-a-1239 | 0.000955856 | 733.2140713 | | 273 | rs818415 | 0.01235 | 0.1820 | 0.1232 | PD || id:ieu-b-7 | Years of schooling || id:ieu-a-1239 | 0.000973297 | 746.6061091 | | 274 | rs892612 | 0.01464 | 0.8418 | 0.0856 | PD || id:ieu-b-7 | Years of schooling || id:ieu-a-1239 | 0.000899442 | 689.9018477 | | 275 | rs894067 | 0.01041 | 0.3929 | 0.2418 | PD || id:ieu-b-7 | Years of schooling || id:ieu-a-1239 | 0.001217714 | 934.3242166 | | 276 | rs9289300 | 0.01512 | 0.1837 | 0.4752 | PD || id:ieu-b-7 | Years of schooling || id:ieu-a-1239 | 0.000910963 | 698.7467432 | | 277 | rs9320493 | -0.01394 | 0.8639 | 0.2508 | PD || id:ieu-b-7 | Years of schooling || id:ieu-a-1239 | 0.000888209 | 681.2780746 | | 278 | rs9342482 | 0.01264 | 0.2908 | 0.3429 | PD || id:ieu-b-7 | Years of schooling || id:ieu-a-1239 | 0.001081873 | 829.9834411 | | 279 | rs9349956 | 0.01881 | 0.2398 | 0.0131 | PD || id:ieu-b-7 | Years of schooling || id:ieu-a-1239 | 0.000947367 | 726.6966129 | | 280 | rs9372625 | 0.02383 | 0.4133 | 0.0276 | PD || id:ieu-b-7 | Years of schooling || id:ieu-a-1239 | 0.001210803 | 929.0155563 | | 281 | rs9384679 | -0.00959 | 0.4082 | 0.7069 | PD || id:ieu-b-7 | Years of schooling || id:ieu-a-1239 | 0.001210803 | 929.0155563 | | 282 | rs9386319 | 0.00991 | 0.4269 | 0.8142 | PD || id:ieu-b-7 | Years of schooling || id:ieu-a-1239 | 0.001224704 | 939.693896 | | 283 | rs9386787 | 0.00958 | 0.5136 | 0.9162 | PD || id:ieu-b-7 | Years of schooling || id:ieu-a-1239 | 0.001253484 | 961.8043406 | | 284 | rs9436866 | 0.01882 | 0.0952 | 0.6235 | PD || id:ieu-b-7 | Years of schooling || id:ieu-a-1239 | 0.000737724 | 565.7672592 | | 285 | rs9503598 | 0.01079 | 0.4388 | 0.9404 | PD || id:ieu-b-7 | Years of schooling || id:ieu-a-1239 | 0.001246163 | 956.1797538 | | 286 | rs9529119 | -0.01295 | 0.8027 | 0.8621 | PD || id:ieu-b-7 | Years of schooling || id:ieu-a-1239 | 0.001044788 | 801.5036172 | | 287 | rs9556958 | -0.0108 | 0.5289 | 0.9411 | PD || id:ieu-b-7 | Years of schooling || id:ieu-a-1239 | 0.001253484 | 961.8043406 | | 288 | rs9616906 | 0.01497 | 0.4235 | 0.4031 | PD || id:ieu-b-7 | Years of schooling || id:ieu-a-1239 | 0.001238927 | 950.6205692 | | 289 | rs9679654 | 0.01042 | 0.4847 | 0.0821 | PD || id:ieu-b-7 | Years of schooling || id:ieu-a-1239 | 0.001238927 | 950.6205692 | | 290 | rs969512 | 0.01249 | 0.2959 | 0.1800 | PD || id:ieu-b-7 | Years of schooling || id:ieu-a-1239 | 0.001190535 | 913.4454631 | | 291 | rs9704097 | -0.0103 | 0.4728 | 0.3566 | PD || id:ieu-b-7 | Years of schooling || id:ieu-a-1239 | 0.001246163 | 956.1797538 | | 292 | rs9914918 | 0.01155 | 0.2823 | 0.3446 | PD || id:ieu-b-7 | Years of schooling || id:ieu-a-1239 | 0.001127615 | 865.1150153 | | 293 | rs9936270 | -0.0136 | 0.3078 | 0.8135 | PD || id:ieu-b-7 | Years of schooling || id:ieu-a-1239 | 0.001076414 | 825.7916056 | | 294 | rs9938678 | 0.01355 | 0.2432 | 0.4642 | PD || id:ieu-b-7 | Years of schooling || id:ieu-a-1239 | 0.001039697 | 797.5938434 | | 295 | rs9964724 | 0.01978 | 0.6599 | 0.7692 | PD || id:ieu-b-7 | Years of schooling || id:ieu-a-1239 | 0.001164542 | 893.4794421 | | 296 | rs9995567 | 0.00998 | 0.3827 | 0.7250 | PD || id:ieu-b-7 | Years of schooling || id:ieu-a-1239 | 0.001197215 | 918.5771792 | | 1 | rs1007934 | 0.0160767 | 0.3962 | 0.4848 | FEMALEPD | Intelligence || id:ebi-a-GCST006250 | 0.001318968 | 356.4133816 | | 2 | rs10189857 | -0.018996 | 0.4153 | 0.0167 | FEMALEPD | Intelligence || id:ebi-a-GCST006250 | 0.001345759 | 363.6627617 | | 3 | rs10189912 | 0.0193366 | 0.3770 | 0.6886 | FEMALEPD | Intelligence || id:ebi-a-GCST006250 | 0.001297202 | 350.5240685 | | 4 | rs1054442 | 0.0214635 | 0.3666 | 0.0859 | FEMALEPD | Intelligence || id:ebi-a-GCST006250 | 0.001313987 | 355.0656127 | | 5 | rs10779271 | -0.016375 | 0.3133 | 0.9989 | FEMALEPD | Intelligence || id:ebi-a-GCST006250 | 0.001265171 | 341.8579395 | | 6 | rs10917152 | 0.0242133 | 0.1361 | 0.1077 | FEMALEPD | Intelligence || id:ebi-a-GCST006250 | 0.000914323 | 246.9696815 | | 7 | rs10954779 | -0.016377 | 0.5476 | 0.2787 | FEMALEPD | Intelligence || id:ebi-a-GCST006250 | 0.001339915 | 362.0813273 | | 8 | rs11076962 | -0.016936 | 0.2856 | 0.0940 | FEMALEPD | Intelligence || id:ebi-a-GCST006250 | 0.001216756 | 328.7600031 | | 9 | rs11079849 | 0.0165478 | 0.3255 | 0.7733 | FEMALEPD | Intelligence || id:ebi-a-GCST006250 | 0.001250421 | 337.8672945 | | 10 | rs11210871 | 0.0173454 | 0.6862 | 0.0590 | FEMALEPD | Intelligence || id:ebi-a-GCST006250 | 0.001264597 | 341.7025761 | | 11 | rs112780312 | -0.018284 | 0.2834 | 0.5779 | FEMALEPD | Intelligence || id:ebi-a-GCST006250 | 0.001194109 | 322.6334144 | | 12 | rs1145123 | -0.020557 | 0.4769 | 0.1190 | FEMALEPD | Intelligence || id:ebi-a-GCST006250 | 0.001335198 | 360.8049578 | | 13 | rs115064 | -0.016096 | 0.3963 | 0.1979 | FEMALEPD | Intelligence || id:ebi-a-GCST006250 | 0.001315078 | 355.3608677 | | 14 | rs11605348 | -0.016607 | 0.3507 | 0.3468 | FEMALEPD | Intelligence || id:ebi-a-GCST006250 | 0.001277987 | 345.3251568 | | 15 | rs11623436 | -0.015774 | 0.4670 | 0.0731 | FEMALEPD | Intelligence || id:ebi-a-GCST006250 | 0.001345627 | 363.6270573 | | 16 | rs11634187 | -0.022032 | 0.1523 | 0.2802 | FEMALEPD | Intelligence || id:ebi-a-GCST006250 | 0.000959754 | 259.252825 | | 17 | rs11646221 | 0.0177354 | 0.5702 | 0.6783 | FEMALEPD | Intelligence || id:ebi-a-GCST006250 | 0.001335184 | 360.8010524 | | 18 | rs11678106 | 0.0160859 | 0.5029 | 0.0709 | FEMALEPD | Intelligence || id:ebi-a-GCST006250 | 0.00134808 | 364.2907167 | | 19 | rs11720523 | 0.0183255 | 0.4336 | 0.7121 | FEMALEPD | Intelligence || id:ebi-a-GCST006250 | 0.001334395 | 360.5876862 | | 20 | rs11793831 | 0.0278338 | 0.3971 | 0.1836 | FEMALEPD | Intelligence || id:ebi-a-GCST006250 | 0.001319579 | 356.578599 | | 21 | rs11898362 | -0.017981 | 0.2884 | 0.0680 | FEMALEPD | Intelligence || id:ebi-a-GCST006250 | 0.001230453 | 332.4653449 | | 22 | rs12026245 | -0.01786 | 0.4978 | 0.0940 | FEMALEPD | Intelligence || id:ebi-a-GCST006250 | 0.00135568 | 366.3473031 | | 23 | rs12035012 | -0.026992 | 0.2236 | 0.9653 | FEMALEPD | Intelligence || id:ebi-a-GCST006250 | 0.001117474 | 301.9046062 | | 24 | rs12190777 | -0.01703 | 0.2723 | 0.6674 | FEMALEPD | Intelligence || id:ebi-a-GCST006250 | 0.001197067 | 323.433789 | | 25 | rs1233578 | 0.0237975 | 0.1595 | 0.0399 | FEMALEPD | Intelligence || id:ebi-a-GCST006250 | 0.000948621 | 256.2427839 | | 26 | rs12470949 | 0.0171675 | 0.7262 | 0.1905 | FEMALEPD | Intelligence || id:ebi-a-GCST006250 | 0.001225148 | 331.0302031 | | 27 | rs12535854 | 0.0182259 | 0.6574 | 0.0727 | FEMALEPD | Intelligence || id:ebi-a-GCST006250 | 0.001253267 | 338.6373096 | | 28 | rs12646225 | 0.0251275 | 0.1113 | 0.0554 | FEMALEPD | Intelligence || id:ebi-a-GCST006250 | 0.00087829 | 237.2281556 | | 29 | rs1280049 | -0.014993 | 0.5106 | 0.2272 | FEMALEPD | Intelligence || id:ebi-a-GCST006250 | 0.001356037 | 366.4439608 | | 30 | rs12886584 | -0.020517 | 0.1914 | 0.4176 | FEMALEPD | Intelligence || id:ebi-a-GCST006250 | 0.001035786 | 279.8121296 | | 31 | rs13024268 | -0.016661 | 0.3834 | 0.2508 | FEMALEPD | Intelligence || id:ebi-a-GCST006250 | 0.001285443 | 347.3426661 | | 32 | rs13071190 | -0.018095 | 0.3224 | 0.8035 | FEMALEPD | Intelligence || id:ebi-a-GCST006250 | 0.001268645 | 342.7977954 | | 33 | rs13165296 | -0.019636 | 0.1769 | 0.4717 | FEMALEPD | Intelligence || id:ebi-a-GCST006250 | 0.001051534 | 284.0710492 | | 34 | rs13212044 | -0.018368 | 0.2480 | 0.5687 | FEMALEPD | Intelligence || id:ebi-a-GCST006250 | 0.001141749 | 308.4702197 | | 35 | rs13223152 | -0.017645 | 0.3956 | 0.5747 | FEMALEPD | Intelligence || id:ebi-a-GCST006250 | 0.001329268 | 359.2004817 | | 36 | rs13253386 | 0.0201329 | 0.4870 | 0.5305 | FEMALEPD | Intelligence || id:ebi-a-GCST006250 | 0.001346561 | 363.8797838 | | 37 | rs13276212 | 0.0150707 | 0.5109 | 0.3253 | FEMALEPD | Intelligence || id:ebi-a-GCST006250 | 0.001343379 | 363.0185173 | | 38 | rs13395129 | 0.0168652 | 0.3184 | 0.3840 | FEMALEPD | Intelligence || id:ebi-a-GCST006250 | 0.001235026 | 333.702385 | | 39 | rs1362739 | 0.0209452 | 0.4711 | 0.4988 | FEMALEPD | Intelligence || id:ebi-a-GCST006250 | 0.001353689 | 365.8085669 | | 40 | rs1369429 | -0.017633 | 0.6790 | 0.7635 | FEMALEPD | Intelligence || id:ebi-a-GCST006250 | 0.001277775 | 345.2679261 | | 41 | rs1408579 | 0.0160494 | 0.4806 | 0.4833 | FEMALEPD | Intelligence || id:ebi-a-GCST006250 | 0.001346537 | 363.8731634 | | 42 | rs144026674 | 0.0413066 | 0.0495 | 0.9748 | FEMALEPD | Intelligence || id:ebi-a-GCST006250 | 0.000495928 | 133.9000815 | | 43 | rs144246 | 0.0154911 | 0.3780 | 0.0570 | FEMALEPD | Intelligence || id:ebi-a-GCST006250 | 0.00130316 | 352.1361048 | | 44 | rs1589652 | -0.017094 | 0.5601 | 0.7293 | FEMALEPD | Intelligence || id:ebi-a-GCST006250 | 0.001341132 | 362.4106973 | | 45 | rs166820 | 0.0243341 | 0.1610 | 0.2274 | FEMALEPD | Intelligence || id:ebi-a-GCST006250 | 0.001028489 | 277.8390047 | | 46 | rs17002025 | 0.0255982 | 0.1243 | 0.2497 | FEMALEPD | Intelligence || id:ebi-a-GCST006250 | 0.000868801 | 234.6629251 | | 47 | rs17106817 | -0.016911 | 0.2864 | 0.4563 | FEMALEPD | Intelligence || id:ebi-a-GCST006250 | 0.001223591 | 330.6088501 | | 48 | rs17128425 | 0.0255567 | 0.0992 | 0.9510 | FEMALEPD | Intelligence || id:ebi-a-GCST006250 | 0.000814773 | 220.0581374 | | 49 | rs17199964 | -0.039187 | 0.0728 | 0.0237 | FEMALEPD | Intelligence || id:ebi-a-GCST006250 | 0.000648641 | 175.1591927 | | 50 | rs1727307 | -0.017817 | 0.7161 | 0.2422 | FEMALEPD | Intelligence || id:ebi-a-GCST006250 | 0.00123096 | 332.6024636 | | 51 | rs17698176 | 0.0201149 | 0.2170 | 0.0087 | FEMALEPD | Intelligence || id:ebi-a-GCST006250 | 0.001037909 | 280.3864283 | | 52 | rs1812587 | -0.017339 | 0.4539 | 0.2588 | FEMALEPD | Intelligence || id:ebi-a-GCST006250 | 0.00133754 | 361.4387498 | | 53 | rs1831539 | 0.0172035 | 0.4731 | 0.0490 | FEMALEPD | Intelligence || id:ebi-a-GCST006250 | 0.001339678 | 362.0170978 | | 54 | rs1840847 | 0.0163419 | 0.3489 | 0.0360 | FEMALEPD | Intelligence || id:ebi-a-GCST006250 | 0.001283796 | 346.8968422 | | 55 | rs1906252 | 0.0316616 | 0.4812 | 0.0390 | FEMALEPD | Intelligence || id:ebi-a-GCST006250 | 0.001349818 | 364.7611121 | | 56 | rs190925241 | 0.0334286 | 0.0840 | 0.5095 | FEMALEPD | Intelligence || id:ebi-a-GCST006250 | 0.000677162 | 182.8661536 | | 57 | rs1962047 | -0.019533 | 0.3540 | 0.1751 | FEMALEPD | Intelligence || id:ebi-a-GCST006250 | 0.001292713 | 349.3094412 | | 58 | rs1972860 | -0.017556 | 0.3230 | 0.4170 | FEMALEPD | Intelligence || id:ebi-a-GCST006250 | 0.001262912 | 341.2466477 | | 59 | rs2007176 | -0.015401 | 0.4541 | 0.8066 | FEMALEPD | Intelligence || id:ebi-a-GCST006250 | 0.001336966 | 361.2833563 | | 60 | rs2008514 | -0.028679 | 0.3766 | 0.0248 | FEMALEPD | Intelligence || id:ebi-a-GCST006250 | 0.001321998 | 357.2333462 | | 61 | rs2071407 | 0.0219741 | 0.6239 | 0.0362 | FEMALEPD | Intelligence || id:ebi-a-GCST006250 | 0.001294424 | 349.7725024 | | 62 | rs2072490 | 0.0169959 | 0.5138 | 0.7815 | FEMALEPD | Intelligence || id:ebi-a-GCST006250 | 0.001348193 | 364.3212423 | | 63 | rs2111490 | -0.015491 | 0.5334 | 0.2036 | FEMALEPD | Intelligence || id:ebi-a-GCST006250 | 0.001344207 | 363.2426875 | | 64 | rs2239647 | 0.0205367 | 0.5416 | 0.4843 | FEMALEPD | Intelligence || id:ebi-a-GCST006250 | 0.001337753 | 361.49624 | | 65 | rs2268894 | 0.0207848 | 0.5459 | 0.1823 | FEMALEPD | Intelligence || id:ebi-a-GCST006250 | 0.001346346 | 363.8215329 | | 66 | rs2285640 | 0.0175137 | 0.5377 | 0.9157 | FEMALEPD | Intelligence || id:ebi-a-GCST006250 | 0.00133856 | 361.7146083 | | 67 | rs2309812 | 0.0228352 | 0.3764 | 0.7493 | FEMALEPD | Intelligence || id:ebi-a-GCST006250 | 0.001300872 | 351.5171907 | | 68 | rs2352974 | -0.030841 | 0.4726 | 0.1041 | FEMALEPD | Intelligence || id:ebi-a-GCST006250 | 0.001344949 | 363.4433565 | | 69 | rs2373353 | 0.0163232 | 0.3654 | 0.7762 | FEMALEPD | Intelligence || id:ebi-a-GCST006250 | 0.001282099 | 346.4377582 | | 70 | rs2393967 | 0.0187089 | 0.3262 | 0.0321 | FEMALEPD | Intelligence || id:ebi-a-GCST006250 | 0.001249198 | 337.5365686 | | 71 | rs2420551 | -0.028596 | 0.8926 | 0.7642 | FEMALEPD | Intelligence || id:ebi-a-GCST006250 | 0.000852321 | 230.2077615 | | 72 | rs2450333 | -0.018829 | 0.5327 | 0.3642 | FEMALEPD | Intelligence || id:ebi-a-GCST006250 | 0.00132214 | 357.2716353 | | 73 | rs2457192 | -0.019751 | 0.7036 | 0.0387 | FEMALEPD | Intelligence || id:ebi-a-GCST006250 | 0.001182056 | 319.37319 | | 74 | rs2478286 | -0.025786 | 0.7421 | 0.5967 | FEMALEPD | Intelligence || id:ebi-a-GCST006250 | 0.001183597 | 319.7898929 | | 75 | rs2508713 | 0.0165308 | 0.3838 | 0.0028 | FEMALEPD | Intelligence || id:ebi-a-GCST006250 | 0.001302683 | 352.0071912 | | 76 | rs2558096 | 0.0156318 | 0.5751 | 0.9519 | FEMALEPD | Intelligence || id:ebi-a-GCST006250 | 0.001334236 | 360.5447832 | | 77 | rs2647995 | 0.0197491 | 0.2786 | 0.9025 | FEMALEPD | Intelligence || id:ebi-a-GCST006250 | 0.001215862 | 328.5180732 | | 78 | rs2678210 | -0.018786 | 0.2931 | 0.1332 | FEMALEPD | Intelligence || id:ebi-a-GCST006250 | 0.001214933 | 328.2668005 | | 79 | rs2721173 | -0.016233 | 0.4754 | 0.8413 | FEMALEPD | Intelligence || id:ebi-a-GCST006250 | 0.00135363 | 365.7925096 | | 80 | rs2726491 | -0.02828 | 0.3667 | 0.5459 | FEMALEPD | Intelligence || id:ebi-a-GCST006250 | 0.00129551 | 350.0663692 | | 81 | rs2836921 | 0.0203465 | 0.3452 | 0.1358 | FEMALEPD | Intelligence || id:ebi-a-GCST006250 | 0.001249068 | 337.5012535 | | 82 | rs28620532 | 0.0163543 | 0.3466 | 0.0670 | FEMALEPD | Intelligence || id:ebi-a-GCST006250 | 0.001280792 | 346.0840606 | | 83 | rs287879 | 0.0188666 | 0.2876 | 0.7306 | FEMALEPD | Intelligence || id:ebi-a-GCST006250 | 0.001203697 | 325.2272832 | | 84 | rs2885208 | -0.018939 | 0.1800 | 0.5686 | FEMALEPD | Intelligence || id:ebi-a-GCST006250 | 0.001068536 | 288.6689632 | | 85 | rs2920940 | 0.0247422 | 0.7670 | 0.1350 | FEMALEPD | Intelligence || id:ebi-a-GCST006250 | 0.001138266 | 307.5282202 | | 86 | rs2955280 | -0.014915 | 0.5099 | 0.1640 | FEMALEPD | Intelligence || id:ebi-a-GCST006250 | 0.001353452 | 365.7443461 | | 87 | rs297578 | 0.0181036 | 0.7053 | 0.6071 | FEMALEPD | Intelligence || id:ebi-a-GCST006250 | 0.001230891 | 332.5836584 | | 88 | rs2987390 | 0.0178028 | 0.2755 | 0.0048 | FEMALEPD | Intelligence || id:ebi-a-GCST006250 | 0.001185221 | 320.2292182 | | 89 | rs3128341 | 0.031727 | 0.8036 | 0.5254 | FEMALEPD | Intelligence || id:ebi-a-GCST006250 | 0.001083141 | 292.6188082 | | 90 | rs31768 | -0.018177 | 0.7028 | 0.1685 | FEMALEPD | Intelligence || id:ebi-a-GCST006250 | 0.001211894 | 327.4445023 | | 91 | rs329672 | 0.0174305 | 0.6338 | 0.7734 | FEMALEPD | Intelligence || id:ebi-a-GCST006250 | 0.00129702 | 350.4749282 | | 92 | rs34316 | -0.021049 | 0.5766 | 0.5893 | FEMALEPD | Intelligence || id:ebi-a-GCST006250 | 0.001337188 | 361.3434085 | | 93 | rs34811474 | 0.0289955 | 0.2198 | 0.6502 | FEMALEPD | Intelligence || id:ebi-a-GCST006250 | 0.001030002 | 278.2479678 | | 94 | rs35608616 | -0.018089 | 0.3568 | 0.1068 | FEMALEPD | Intelligence || id:ebi-a-GCST006250 | 0.001260056 | 340.4740077 | | 95 | rs35731967 | -0.021838 | 0.1852 | 0.3226 | FEMALEPD | Intelligence || id:ebi-a-GCST006250 | 0.001011998 | 273.3796229 | | 96 | rs36033 | -0.015969 | 0.4198 | 0.0541 | FEMALEPD | Intelligence || id:ebi-a-GCST006250 | 0.00132743 | 358.7031311 | | 97 | rs3740422 | -0.024102 | 0.3489 | 0.4497 | FEMALEPD | Intelligence || id:ebi-a-GCST006250 | 0.001271078 | 343.4559442 | | 98 | rs3860537 | -0.018854 | 0.7795 | 0.7245 | FEMALEPD | Intelligence || id:ebi-a-GCST006250 | 0.001088045 | 293.9451521 | | 99 | rs405321 | -0.016432 | 0.3143 | 0.6112 | FEMALEPD | Intelligence || id:ebi-a-GCST006250 | 0.001244119 | 336.1624715 | | 100 | rs4463213 | 0.0190655 | 0.5383 | 0.3271 | FEMALEPD | Intelligence || id:ebi-a-GCST006250 | 0.001354392 | 365.9986857 | | 101 | rs4484297 | 0.0182667 | 0.2489 | 0.1537 | FEMALEPD | Intelligence || id:ebi-a-GCST006250 | 0.001171144 | 316.4213083 | | 102 | rs4667954 | -0.017315 | 0.2839 | 0.3368 | FEMALEPD | Intelligence || id:ebi-a-GCST006250 | 0.001218329 | 329.1853225 | | 103 | rs4725065 | 0.0165317 | 0.5156 | 0.2980 | FEMALEPD | Intelligence || id:ebi-a-GCST006250 | 0.001352464 | 365.4770018 | | 104 | rs4731392 | 0.0217412 | 0.3163 | 0.2830 | FEMALEPD | Intelligence || id:ebi-a-GCST006250 | 0.001244182 | 336.1794233 | | 105 | rs4793161 | 0.0177187 | 0.7739 | 0.3312 | FEMALEPD | Intelligence || id:ebi-a-GCST006250 | 0.001138882 | 307.6947611 | | 106 | rs4821995 | -0.015999 | 0.6593 | 0.7394 | FEMALEPD | Intelligence || id:ebi-a-GCST006250 | 0.001287243 | 347.8295584 | | 107 | rs4852252 | 0.0207869 | 0.5711 | 0.9491 | FEMALEPD | Intelligence || id:ebi-a-GCST006250 | 0.001346919 | 363.9764684 | | 108 | rs4976976 | 0.0173172 | 0.4005 | 0.4131 | FEMALEPD | Intelligence || id:ebi-a-GCST006250 | 0.001332385 | 360.0437056 | | 109 | rs4981713 | -0.016189 | 0.3787 | 0.5919 | FEMALEPD | Intelligence || id:ebi-a-GCST006250 | 0.001322645 | 357.4082665 | | 110 | rs55754731 | -0.021369 | 0.1713 | 0.5734 | FEMALEPD | Intelligence || id:ebi-a-GCST006250 | 0.001007363 | 272.1260794 | | 111 | rs55763037 | -0.018345 | 0.2141 | 0.1845 | FEMALEPD | Intelligence || id:ebi-a-GCST006250 | 0.001110342 | 299.9755785 | | 112 | rs56150095 | -0.021968 | 0.5319 | 0.3292 | FEMALEPD | Intelligence || id:ebi-a-GCST006250 | 0.00134709 | 364.0228422 | | 113 | rs566237 | 0.0187161 | 0.3193 | 0.2731 | FEMALEPD | Intelligence || id:ebi-a-GCST006250 | 0.001260768 | 340.6665493 | | 114 | rs5750830 | 0.0228911 | 0.7165 | 0.1372 | FEMALEPD | Intelligence || id:ebi-a-GCST006250 | 0.001183695 | 319.8164844 | | 115 | rs58593843 | -0.027683 | 0.0724 | 0.4039 | FEMALEPD | Intelligence || id:ebi-a-GCST006250 | 0.000795952 | 214.9708044 | | 116 | rs59142272 | 0.0226956 | 0.1749 | 0.6599 | FEMALEPD | Intelligence || id:ebi-a-GCST006250 | 0.001004574 | 271.3720916 | | 117 | rs600806 | -0.019293 | 0.7362 | 0.9720 | FEMALEPD | Intelligence || id:ebi-a-GCST006250 | 0.001203209 | 325.0951199 | | 118 | rs6019535 | 0.0251054 | 0.3056 | 0.2993 | FEMALEPD | Intelligence || id:ebi-a-GCST006250 | 0.001243593 | 336.0201442 | | 119 | rs60262711 | 0.0159492 | 0.3690 | 0.1007 | FEMALEPD | Intelligence || id:ebi-a-GCST006250 | 0.001309807 | 353.9345743 | | 120 | rs62181012 | -0.021146 | 0.1937 | 0.1813 | FEMALEPD | Intelligence || id:ebi-a-GCST006250 | 0.001054164 | 284.7821511 | | 121 | rs62198803 | 0.0190651 | 0.2449 | 0.9217 | FEMALEPD | Intelligence || id:ebi-a-GCST006250 | 0.001147551 | 310.0396509 | | 122 | rs6508220 | 0.022748 | 0.4826 | 0.2544 | FEMALEPD | Intelligence || id:ebi-a-GCST006250 | 0.001351823 | 365.3034375 | | 123 | rs6535809 | -0.019648 | 0.4898 | 0.9424 | FEMALEPD | Intelligence || id:ebi-a-GCST006250 | 0.001353447 | 365.7430084 | | 124 | rs6539284 | 0.0194809 | 0.4151 | 0.2885 | FEMALEPD | Intelligence || id:ebi-a-GCST006250 | 0.001308858 | 353.6779558 | | 125 | rs6550835 | -0.024809 | 0.3240 | 0.7499 | FEMALEPD | Intelligence || id:ebi-a-GCST006250 | 0.00126364 | 341.443562 | | 126 | rs6668048 | -0.021458 | 0.5075 | 0.2146 | FEMALEPD | Intelligence || id:ebi-a-GCST006250 | 0.001353417 | 365.7349824 | | 127 | rs66954617 | 0.0208823 | 0.6207 | 0.7039 | FEMALEPD | Intelligence || id:ebi-a-GCST006250 | 0.001305911 | 352.8804393 | | 128 | rs67482514 | 0.0178589 | 0.2465 | 0.9606 | FEMALEPD | Intelligence || id:ebi-a-GCST006250 | 0.001146099 | 309.6469964 | | 129 | rs6770622 | -0.044961 | 0.0375 | 0.5924 | FEMALEPD | Intelligence || id:ebi-a-GCST006250 | 0.000539534 | 145.6799866 | | 130 | rs6819372 | 0.0197961 | 0.5292 | 0.7198 | FEMALEPD | Intelligence || id:ebi-a-GCST006250 | 0.001356186 | 366.4842498 | | 131 | rs6860963 | 0.0202625 | 0.1892 | 0.2952 | FEMALEPD | Intelligence || id:ebi-a-GCST006250 | 0.001064851 | 287.6724505 | | 132 | rs6903716 | -0.017758 | 0.2990 | 0.3779 | FEMALEPD | Intelligence || id:ebi-a-GCST006250 | 0.001243986 | 336.1263135 | | 133 | rs702222 | -0.019831 | 0.3576 | 0.9238 | FEMALEPD | Intelligence || id:ebi-a-GCST006250 | 0.001288586 | 348.1928964 | | 134 | rs7069887 | -0.022532 | 0.1562 | 0.3287 | FEMALEPD | Intelligence || id:ebi-a-GCST006250 | 0.000949837 | 256.5715093 | | 135 | rs7116046 | 0.0157066 | 0.3918 | 0.3562 | FEMALEPD | Intelligence || id:ebi-a-GCST006250 | 0.001302006 | 351.8238999 | | 136 | rs7248006 | 0.019175 | 0.6222 | 0.2297 | FEMALEPD | Intelligence || id:ebi-a-GCST006250 | 0.001312168 | 354.5733525 | | 137 | rs72739469 | 0.0343573 | 0.0604 | 0.8002 | FEMALEPD | Intelligence || id:ebi-a-GCST006250 | 0.000655637 | 177.0496909 | | 138 | rs72768642 | 0.0306239 | 0.0680 | 0.7980 | FEMALEPD | Intelligence || id:ebi-a-GCST006250 | 0.000685244 | 185.0501654 | | 139 | rs73068339 | 0.0188582 | 0.2754 | 0.9705 | FEMALEPD | Intelligence || id:ebi-a-GCST006250 | 0.001215216 | 328.3433278 | | 140 | rs7312919 | -0.018146 | 0.3500 | 0.4831 | FEMALEPD | Intelligence || id:ebi-a-GCST006250 | 0.001269597 | 343.0553383 | | 141 | rs7357604 | -0.015705 | 0.3964 | 0.7592 | FEMALEPD | Intelligence || id:ebi-a-GCST006250 | 0.001312302 | 354.609816 | | 142 | rs7573001 | -0.016251 | 0.3947 | 0.3028 | FEMALEPD | Intelligence || id:ebi-a-GCST006250 | 0.001294139 | 349.6954441 | | 143 | rs75973558 | -0.025636 | 0.1265 | 0.7493 | FEMALEPD | Intelligence || id:ebi-a-GCST006250 | 0.000829151 | 223.9444499 | | 144 | rs7640196 | -0.017454 | 0.2256 | 0.8940 | FEMALEPD | Intelligence || id:ebi-a-GCST006250 | 0.001173287 | 317.0010806 | | 145 | rs7652296 | -0.016533 | 0.4039 | 0.4861 | FEMALEPD | Intelligence || id:ebi-a-GCST006250 | 0.001321946 | 357.2193089 | | 146 | rs7731260 | 0.0152959 | 0.4934 | 0.3018 | FEMALEPD | Intelligence || id:ebi-a-GCST006250 | 0.001348512 | 364.4075378 | | 147 | rs78084033 | 0.0228755 | 0.1377 | 0.6212 | FEMALEPD | Intelligence || id:ebi-a-GCST006250 | 0.000914229 | 246.9440665 | | 148 | rs7941785 | -0.015512 | 0.6375 | 0.3970 | FEMALEPD | Intelligence || id:ebi-a-GCST006250 | 0.001302583 | 351.9799331 | | 149 | rs799444 | -0.018415 | 0.5454 | 0.0317 | FEMALEPD | Intelligence || id:ebi-a-GCST006250 | 0.001341166 | 362.4198915 | | 150 | rs8006700 | -0.018227 | 0.6888 | 0.6750 | FEMALEPD | Intelligence || id:ebi-a-GCST006250 | 0.001262925 | 341.2501413 | | 151 | rs80170948 | -0.045381 | 0.0433 | 0.5556 | FEMALEPD | Intelligence || id:ebi-a-GCST006250 | 0.000502005 | 135.5414908 | | 152 | rs8025964 | 0.0170306 | 0.4624 | 0.3420 | FEMALEPD | Intelligence || id:ebi-a-GCST006250 | 0.001346312 | 363.8122675 | | 153 | rs8051038 | 0.018924 | 0.7335 | 0.0275 | FEMALEPD | Intelligence || id:ebi-a-GCST006250 | 0.001176614 | 317.9010077 | | 154 | rs8054299 | 0.0230055 | 0.3347 | 0.6550 | FEMALEPD | Intelligence || id:ebi-a-GCST006250 | 0.00126442 | 341.6547106 | | 155 | rs889169 | 0.0160711 | 0.6154 | 0.1731 | FEMALEPD | Intelligence || id:ebi-a-GCST006250 | 0.001279646 | 345.774121 | | 156 | rs913264 | 0.0197245 | 0.2958 | 0.5981 | FEMALEPD | Intelligence || id:ebi-a-GCST006250 | 0.001223187 | 330.4995832 | | 157 | rs9384679 | -0.026724 | 0.3866 | 0.5388 | FEMALEPD | Intelligence || id:ebi-a-GCST006250 | 0.001329488 | 359.2598434 | | 158 | rs9503599 | 0.0171108 | 0.4401 | 0.3924 | FEMALEPD | Intelligence || id:ebi-a-GCST006250 | 0.001328782 | 359.0689234 | | 159 | rs9516855 | -0.033427 | 0.0531 | 0.9001 | FEMALEPD | Intelligence || id:ebi-a-GCST006250 | 0.000607449 | 164.0289397 | | 160 | rs9569206 | 0.0154126 | 0.3601 | 0.5498 | FEMALEPD | Intelligence || id:ebi-a-GCST006250 | 0.001310265 | 354.0586357 | | 161 | rs967569 | -0.017976 | 0.6417 | 0.8029 | FEMALEPD | Intelligence || id:ebi-a-GCST006250 | 0.001264312 | 341.6255309 | | 162 | rs9888986 | -0.023502 | 0.1134 | 0.2533 | FEMALEPD | Intelligence || id:ebi-a-GCST006250 | 0.000868632 | 234.6172283 | | 1 | rs1007934 | 0.0160767 | 0.3948 | 0.9441 | MALEPD | Intelligence || id:ebi-a-GCST006250 | 0.001318968 | 356.4133816 | | 2 | rs10189857 | -0.018996 | 0.4174 | 0.7026 | MALEPD | Intelligence || id:ebi-a-GCST006250 | 0.001345759 | 363.6627617 | | 3 | rs10189912 | 0.0193366 | 0.3762 | 0.1261 | MALEPD | Intelligence || id:ebi-a-GCST006250 | 0.001297202 | 350.5240685 | | 4 | rs1054442 | 0.0214635 | 0.3662 | 0.0005 | MALEPD | Intelligence || id:ebi-a-GCST006250 | 0.001313987 | 355.0656127 | | 5 | rs10779271 | -0.016375 | 0.3152 | 0.9573 | MALEPD | Intelligence || id:ebi-a-GCST006250 | 0.001265171 | 341.8579395 | | 6 | rs10917152 | 0.0242133 | 0.1357 | 0.1879 | MALEPD | Intelligence || id:ebi-a-GCST006250 | 0.000914323 | 246.9696815 | | 7 | rs10954779 | -0.016377 | 0.5479 | 0.3768 | MALEPD | Intelligence || id:ebi-a-GCST006250 | 0.001339915 | 362.0813273 | | 8 | rs11076962 | -0.016936 | 0.2857 | 0.7846 | MALEPD | Intelligence || id:ebi-a-GCST006250 | 0.001216756 | 328.7600031 | | 9 | rs11079849 | 0.0165478 | 0.3254 | 0.6272 | MALEPD | Intelligence || id:ebi-a-GCST006250 | 0.001250421 | 337.8672945 | | 10 | rs11210871 | 0.0173454 | 0.6875 | 0.0652 | MALEPD | Intelligence || id:ebi-a-GCST006250 | 0.001264597 | 341.7025761 | | 11 | rs112780312 | -0.018284 | 0.2837 | 0.0089 | MALEPD | Intelligence || id:ebi-a-GCST006250 | 0.001194109 | 322.6334144 | | 12 | rs1145123 | -0.020557 | 0.4752 | 0.1914 | MALEPD | Intelligence || id:ebi-a-GCST006250 | 0.001335198 | 360.8049578 | | 13 | rs115064 | -0.016096 | 0.3986 | 0.6596 | MALEPD | Intelligence || id:ebi-a-GCST006250 | 0.001315078 | 355.3608677 | | 14 | rs11605348 | -0.016607 | 0.3496 | 0.6734 | MALEPD | Intelligence || id:ebi-a-GCST006250 | 0.001277987 | 345.3251568 | | 15 | rs11623436 | -0.015774 | 0.4674 | 0.0381 | MALEPD | Intelligence || id:ebi-a-GCST006250 | 0.001345627 | 363.6270573 | | 16 | rs11634187 | -0.022032 | 0.1551 | 0.5771 | MALEPD | Intelligence || id:ebi-a-GCST006250 | 0.000959754 | 259.252825 | | 17 | rs11646221 | 0.0177354 | 0.5730 | 0.0429 | MALEPD | Intelligence || id:ebi-a-GCST006250 | 0.001335184 | 360.8010524 | | 18 | rs11678106 | 0.0160859 | 0.5046 | 0.8764 | MALEPD | Intelligence || id:ebi-a-GCST006250 | 0.00134808 | 364.2907167 | | 19 | rs11720523 | 0.0183255 | 0.4315 | 0.0096 | MALEPD | Intelligence || id:ebi-a-GCST006250 | 0.001334395 | 360.5876862 | | 20 | rs11793831 | 0.0278338 | 0.3988 | 0.7457 | MALEPD | Intelligence || id:ebi-a-GCST006250 | 0.001319579 | 356.578599 | | 21 | rs11898362 | -0.017981 | 0.2905 | 0.5284 | MALEPD | Intelligence || id:ebi-a-GCST006250 | 0.001230453 | 332.4653449 | | 22 | rs12026245 | -0.01786 | 0.5008 | 0.5004 | MALEPD | Intelligence || id:ebi-a-GCST006250 | 0.00135568 | 366.3473031 | | 23 | rs12035012 | -0.026992 | 0.2236 | 0.2592 | MALEPD | Intelligence || id:ebi-a-GCST006250 | 0.001117474 | 301.9046062 | | 24 | rs12190777 | -0.01703 | 0.2700 | 0.0826 | MALEPD | Intelligence || id:ebi-a-GCST006250 | 0.001197067 | 323.433789 | | 25 | rs1233578 | 0.0237975 | 0.1593 | 0.6414 | MALEPD | Intelligence || id:ebi-a-GCST006250 | 0.000948621 | 256.2427839 | | 26 | rs12470949 | 0.0171675 | 0.7240 | 0.1682 | MALEPD | Intelligence || id:ebi-a-GCST006250 | 0.001225148 | 331.0302031 | | 27 | rs12535854 | 0.0182259 | 0.6601 | 0.0427 | MALEPD | Intelligence || id:ebi-a-GCST006250 | 0.001253267 | 338.6373096 | | 28 | rs12646225 | 0.0251275 | 0.1098 | 0.1058 | MALEPD | Intelligence || id:ebi-a-GCST006250 | 0.00087829 | 237.2281556 | | 29 | rs1280049 | -0.014993 | 0.5106 | 0.1623 | MALEPD | Intelligence || id:ebi-a-GCST006250 | 0.001356037 | 366.4439608 | | 30 | rs12886584 | -0.020517 | 0.1932 | 0.9428 | MALEPD | Intelligence || id:ebi-a-GCST006250 | 0.001035786 | 279.8121296 | | 31 | rs13024268 | -0.016661 | 0.3846 | 0.2068 | MALEPD | Intelligence || id:ebi-a-GCST006250 | 0.001285443 | 347.3426661 | | 32 | rs13071190 | -0.018095 | 0.3225 | 0.0165 | MALEPD | Intelligence || id:ebi-a-GCST006250 | 0.001268645 | 342.7977954 | | 33 | rs13165296 | -0.019636 | 0.1768 | 0.2320 | MALEPD | Intelligence || id:ebi-a-GCST006250 | 0.001051534 | 284.0710492 | | 34 | rs13212044 | -0.018368 | 0.2503 | 0.9596 | MALEPD | Intelligence || id:ebi-a-GCST006250 | 0.001141749 | 308.4702197 | | 35 | rs13223152 | -0.017645 | 0.3962 | 0.6561 | MALEPD | Intelligence || id:ebi-a-GCST006250 | 0.001329268 | 359.2004817 | | 36 | rs13253386 | 0.0201329 | 0.4921 | 0.5013 | MALEPD | Intelligence || id:ebi-a-GCST006250 | 0.001346561 | 363.8797838 | | 37 | rs13276212 | 0.0150707 | 0.5108 | 0.8479 | MALEPD | Intelligence || id:ebi-a-GCST006250 | 0.001343379 | 363.0185173 | | 38 | rs13395129 | 0.0168652 | 0.3173 | 0.9915 | MALEPD | Intelligence || id:ebi-a-GCST006250 | 0.001235026 | 333.702385 | | 39 | rs1362739 | 0.0209452 | 0.4702 | 0.8971 | MALEPD | Intelligence || id:ebi-a-GCST006250 | 0.001353689 | 365.8085669 | | 40 | rs1369429 | -0.017633 | 0.6822 | 0.4924 | MALEPD | Intelligence || id:ebi-a-GCST006250 | 0.001277775 | 345.2679261 | | 41 | rs1408579 | 0.0160494 | 0.4825 | 0.5540 | MALEPD | Intelligence || id:ebi-a-GCST006250 | 0.001346537 | 363.8731634 | | 42 | rs144026674 | 0.0413066 | 0.0479 | 0.7371 | MALEPD | Intelligence || id:ebi-a-GCST006250 | 0.000495928 | 133.9000815 | | 43 | rs144246 | 0.0154911 | 0.3781 | 0.8132 | MALEPD | Intelligence || id:ebi-a-GCST006250 | 0.00130316 | 352.1361048 | | 44 | rs1589652 | -0.017094 | 0.5626 | 0.1082 | MALEPD | Intelligence || id:ebi-a-GCST006250 | 0.001341132 | 362.4106973 | | 45 | rs166820 | 0.0243341 | 0.1599 | 0.8380 | MALEPD | Intelligence || id:ebi-a-GCST006250 | 0.001028489 | 277.8390047 | | 46 | rs17002025 | 0.0255982 | 0.1241 | 0.9469 | MALEPD | Intelligence || id:ebi-a-GCST006250 | 0.000868801 | 234.6629251 | | 47 | rs17106817 | -0.016911 | 0.2868 | 0.4474 | MALEPD | Intelligence || id:ebi-a-GCST006250 | 0.001223591 | 330.6088501 | | 48 | rs17128425 | 0.0255567 | 0.0987 | 0.7411 | MALEPD | Intelligence || id:ebi-a-GCST006250 | 0.000814773 | 220.0581374 | | 49 | rs17199964 | -0.039187 | 0.0747 | 0.0137 | MALEPD | Intelligence || id:ebi-a-GCST006250 | 0.000648641 | 175.1591927 | | 50 | rs1727307 | -0.017817 | 0.7131 | 0.0223 | MALEPD | Intelligence || id:ebi-a-GCST006250 | 0.00123096 | 332.6024636 | | 51 | rs17698176 | 0.0201149 | 0.2171 | 0.0000 | MALEPD | Intelligence || id:ebi-a-GCST006250 | 0.001037909 | 280.3864283 | | 52 | rs1812587 | -0.017339 | 0.4548 | 0.2708 | MALEPD | Intelligence || id:ebi-a-GCST006250 | 0.00133754 | 361.4387498 | | 53 | rs1831539 | 0.0172035 | 0.4736 | 0.1179 | MALEPD | Intelligence || id:ebi-a-GCST006250 | 0.001339678 | 362.0170978 | | 54 | rs1840847 | 0.0163419 | 0.3446 | 0.8520 | MALEPD | Intelligence || id:ebi-a-GCST006250 | 0.001283796 | 346.8968422 | | 55 | rs1906252 | 0.0316616 | 0.4820 | 0.1229 | MALEPD | Intelligence || id:ebi-a-GCST006250 | 0.001349818 | 364.7611121 | | 56 | rs190925241 | 0.0334286 | 0.0834 | 0.2028 | MALEPD | Intelligence || id:ebi-a-GCST006250 | 0.000677162 | 182.8661536 | | 57 | rs1962047 | -0.019533 | 0.3561 | 0.1175 | MALEPD | Intelligence || id:ebi-a-GCST006250 | 0.001292713 | 349.3094412 | | 58 | rs1972860 | -0.017556 | 0.3210 | 0.5095 | MALEPD | Intelligence || id:ebi-a-GCST006250 | 0.001262912 | 341.2466477 | | 59 | rs2007176 | -0.015401 | 0.4544 | 0.0030 | MALEPD | Intelligence || id:ebi-a-GCST006250 | 0.001336966 | 361.2833563 | | 60 | rs2008514 | -0.028679 | 0.3786 | 0.2037 | MALEPD | Intelligence || id:ebi-a-GCST006250 | 0.001321998 | 357.2333462 | | 61 | rs2071407 | 0.0219741 | 0.6268 | 0.4448 | MALEPD | Intelligence || id:ebi-a-GCST006250 | 0.001294424 | 349.7725024 | | 62 | rs2072490 | 0.0169959 | 0.5160 | 0.5424 | MALEPD | Intelligence || id:ebi-a-GCST006250 | 0.001348193 | 364.3212423 | | 63 | rs2111490 | -0.015491 | 0.5299 | 0.7263 | MALEPD | Intelligence || id:ebi-a-GCST006250 | 0.001344207 | 363.2426875 | | 64 | rs2239647 | 0.0205367 | 0.5419 | 0.2727 | MALEPD | Intelligence || id:ebi-a-GCST006250 | 0.001337753 | 361.49624 | | 65 | rs2268894 | 0.0207848 | 0.5432 | 0.9757 | MALEPD | Intelligence || id:ebi-a-GCST006250 | 0.001346346 | 363.8215329 | | 66 | rs2285640 | 0.0175137 | 0.5334 | 0.1081 | MALEPD | Intelligence || id:ebi-a-GCST006250 | 0.00133856 | 361.7146083 | | 67 | rs2309812 | 0.0228352 | 0.3777 | 0.5526 | MALEPD | Intelligence || id:ebi-a-GCST006250 | 0.001300872 | 351.5171907 | | 68 | rs2352974 | -0.030841 | 0.4767 | 0.7323 | MALEPD | Intelligence || id:ebi-a-GCST006250 | 0.001344949 | 363.4433565 | | 69 | rs2373353 | 0.0163232 | 0.3661 | 0.9298 | MALEPD | Intelligence || id:ebi-a-GCST006250 | 0.001282099 | 346.4377582 | | 70 | rs2393967 | 0.0187089 | 0.3255 | 0.4975 | MALEPD | Intelligence || id:ebi-a-GCST006250 | 0.001249198 | 337.5365686 | | 71 | rs2420551 | -0.028596 | 0.8943 | 0.5757 | MALEPD | Intelligence || id:ebi-a-GCST006250 | 0.000852321 | 230.2077615 | | 72 | rs2450333 | -0.018829 | 0.5347 | 0.5003 | MALEPD | Intelligence || id:ebi-a-GCST006250 | 0.00132214 | 357.2716353 | | 73 | rs2457192 | -0.019751 | 0.6998 | 0.4398 | MALEPD | Intelligence || id:ebi-a-GCST006250 | 0.001182056 | 319.37319 | | 74 | rs2478286 | -0.025786 | 0.7431 | 0.0913 | MALEPD | Intelligence || id:ebi-a-GCST006250 | 0.001183597 | 319.7898929 | | 75 | rs2508713 | 0.0165308 | 0.3856 | 0.3993 | MALEPD | Intelligence || id:ebi-a-GCST006250 | 0.001302683 | 352.0071912 | | 76 | rs2558096 | 0.0156318 | 0.5805 | 0.7617 | MALEPD | Intelligence || id:ebi-a-GCST006250 | 0.001334236 | 360.5447832 | | 77 | rs2647995 | 0.0197491 | 0.2769 | 0.8459 | MALEPD | Intelligence || id:ebi-a-GCST006250 | 0.001215862 | 328.5180732 | | 78 | rs2678210 | -0.018786 | 0.2954 | 0.1503 | MALEPD | Intelligence || id:ebi-a-GCST006250 | 0.001214933 | 328.2668005 | | 79 | rs2721173 | -0.016233 | 0.4747 | 0.0457 | MALEPD | Intelligence || id:ebi-a-GCST006250 | 0.00135363 | 365.7925096 | | 80 | rs2726491 | -0.02828 | 0.3682 | 0.2661 | MALEPD | Intelligence || id:ebi-a-GCST006250 | 0.00129551 | 350.0663692 | | 81 | rs2836921 | 0.0203465 | 0.3462 | 0.4842 | MALEPD | Intelligence || id:ebi-a-GCST006250 | 0.001249068 | 337.5012535 | | 82 | rs28620532 | 0.0163543 | 0.3482 | 0.4209 | MALEPD | Intelligence || id:ebi-a-GCST006250 | 0.001280792 | 346.0840606 | | 83 | rs287879 | 0.0188666 | 0.2898 | 0.6195 | MALEPD | Intelligence || id:ebi-a-GCST006250 | 0.001203697 | 325.2272832 | | 84 | rs2885208 | -0.018939 | 0.1791 | 0.0575 | MALEPD | Intelligence || id:ebi-a-GCST006250 | 0.001068536 | 288.6689632 | | 85 | rs2920940 | 0.0247422 | 0.7639 | 0.7407 | MALEPD | Intelligence || id:ebi-a-GCST006250 | 0.001138266 | 307.5282202 | | 86 | rs2955280 | -0.014915 | 0.5062 | 0.1014 | MALEPD | Intelligence || id:ebi-a-GCST006250 | 0.001353452 | 365.7443461 | | 87 | rs297578 | 0.0181036 | 0.7053 | 0.4461 | MALEPD | Intelligence || id:ebi-a-GCST006250 | 0.001230891 | 332.5836584 | | 88 | rs2987390 | 0.0178028 | 0.2750 | 0.7083 | MALEPD | Intelligence || id:ebi-a-GCST006250 | 0.001185221 | 320.2292182 | | 89 | rs3128341 | 0.031727 | 0.8021 | 0.0792 | MALEPD | Intelligence || id:ebi-a-GCST006250 | 0.001083141 | 292.6188082 | | 90 | rs31768 | -0.018177 | 0.7014 | 0.2675 | MALEPD | Intelligence || id:ebi-a-GCST006250 | 0.001211894 | 327.4445023 | | 91 | rs329672 | 0.0174305 | 0.6342 | 0.7009 | MALEPD | Intelligence || id:ebi-a-GCST006250 | 0.00129702 | 350.4749282 | | 92 | rs34316 | -0.021049 | 0.5775 | 0.7733 | MALEPD | Intelligence || id:ebi-a-GCST006250 | 0.001337188 | 361.3434085 | | 93 | rs34811474 | 0.0289955 | 0.2211 | 0.0088 | MALEPD | Intelligence || id:ebi-a-GCST006250 | 0.001030002 | 278.2479678 | | 94 | rs35608616 | -0.018089 | 0.3590 | 0.4654 | MALEPD | Intelligence || id:ebi-a-GCST006250 | 0.001260056 | 340.4740077 | | 95 | rs35731967 | -0.021838 | 0.1858 | 0.9713 | MALEPD | Intelligence || id:ebi-a-GCST006250 | 0.001011998 | 273.3796229 | | 96 | rs36033 | -0.015969 | 0.4218 | 0.3460 | MALEPD | Intelligence || id:ebi-a-GCST006250 | 0.00132743 | 358.7031311 | | 97 | rs3740422 | -0.024102 | 0.3486 | 0.3260 | MALEPD | Intelligence || id:ebi-a-GCST006250 | 0.001271078 | 343.4559442 | | 98 | rs3860537 | -0.018854 | 0.7790 | 0.8229 | MALEPD | Intelligence || id:ebi-a-GCST006250 | 0.001088045 | 293.9451521 | | 99 | rs405321 | -0.016432 | 0.3118 | 0.1114 | MALEPD | Intelligence || id:ebi-a-GCST006250 | 0.001244119 | 336.1624715 | | 100 | rs4463213 | 0.0190655 | 0.5427 | 0.0081 | MALEPD | Intelligence || id:ebi-a-GCST006250 | 0.001354392 | 365.9986857 | | 101 | rs4484297 | 0.0182667 | 0.2482 | 0.4169 | MALEPD | Intelligence || id:ebi-a-GCST006250 | 0.001171144 | 316.4213083 | | 102 | rs4667954 | -0.017315 | 0.2807 | 0.2991 | MALEPD | Intelligence || id:ebi-a-GCST006250 | 0.001218329 | 329.1853225 | | 103 | rs4725065 | 0.0165317 | 0.5167 | 0.3968 | MALEPD | Intelligence || id:ebi-a-GCST006250 | 0.001352464 | 365.4770018 | | 104 | rs4731392 | 0.0217412 | 0.3169 | 0.0460 | MALEPD | Intelligence || id:ebi-a-GCST006250 | 0.001244182 | 336.1794233 | | 105 | rs4793161 | 0.0177187 | 0.7736 | 0.7910 | MALEPD | Intelligence || id:ebi-a-GCST006250 | 0.001138882 | 307.6947611 | | 106 | rs4821995 | -0.015999 | 0.6603 | 0.0003 | MALEPD | Intelligence || id:ebi-a-GCST006250 | 0.001287243 | 347.8295584 | | 107 | rs4852252 | 0.0207869 | 0.5717 | 0.4703 | MALEPD | Intelligence || id:ebi-a-GCST006250 | 0.001346919 | 363.9764684 | | 108 | rs4976976 | 0.0173172 | 0.4002 | 0.7407 | MALEPD | Intelligence || id:ebi-a-GCST006250 | 0.001332385 | 360.0437056 | | 109 | rs4981713 | -0.016189 | 0.3793 | 0.0722 | MALEPD | Intelligence || id:ebi-a-GCST006250 | 0.001322645 | 357.4082665 | | 110 | rs55754731 | -0.021369 | 0.1714 | 0.5649 | MALEPD | Intelligence || id:ebi-a-GCST006250 | 0.001007363 | 272.1260794 | | 111 | rs55763037 | -0.018345 | 0.2153 | 0.4815 | MALEPD | Intelligence || id:ebi-a-GCST006250 | 0.001110342 | 299.9755785 | | 112 | rs56150095 | -0.021968 | 0.5312 | 0.3489 | MALEPD | Intelligence || id:ebi-a-GCST006250 | 0.00134709 | 364.0228422 | | 113 | rs566237 | 0.0187161 | 0.3198 | 0.8864 | MALEPD | Intelligence || id:ebi-a-GCST006250 | 0.001260768 | 340.6665493 | | 114 | rs5750830 | 0.0228911 | 0.7203 | 0.7417 | MALEPD | Intelligence || id:ebi-a-GCST006250 | 0.001183695 | 319.8164844 | | 115 | rs58593843 | -0.027683 | 0.0710 | 0.1031 | MALEPD | Intelligence || id:ebi-a-GCST006250 | 0.000795952 | 214.9708044 | | 116 | rs59142272 | 0.0226956 | 0.1738 | 0.2212 | MALEPD | Intelligence || id:ebi-a-GCST006250 | 0.001004574 | 271.3720916 | | 117 | rs600806 | -0.019293 | 0.7343 | 0.8313 | MALEPD | Intelligence || id:ebi-a-GCST006250 | 0.001203209 | 325.0951199 | | 118 | rs6019535 | 0.0251054 | 0.3096 | 0.2310 | MALEPD | Intelligence || id:ebi-a-GCST006250 | 0.001243593 | 336.0201442 | | 119 | rs60262711 | 0.0159492 | 0.3702 | 0.3696 | MALEPD | Intelligence || id:ebi-a-GCST006250 | 0.001309807 | 353.9345743 | | 120 | rs62181012 | -0.021146 | 0.1955 | 0.8116 | MALEPD | Intelligence || id:ebi-a-GCST006250 | 0.001054164 | 284.7821511 | | 121 | rs62198803 | 0.0190651 | 0.2468 | 0.9597 | MALEPD | Intelligence || id:ebi-a-GCST006250 | 0.001147551 | 310.0396509 | | 122 | rs6508220 | 0.022748 | 0.4826 | 0.4658 | MALEPD | Intelligence || id:ebi-a-GCST006250 | 0.001351823 | 365.3034375 | | 123 | rs6535809 | -0.019648 | 0.4904 | 0.3631 | MALEPD | Intelligence || id:ebi-a-GCST006250 | 0.001353447 | 365.7430084 | | 124 | rs6539284 | 0.0194809 | 0.4188 | 0.0386 | MALEPD | Intelligence || id:ebi-a-GCST006250 | 0.001308858 | 353.6779558 | | 125 | rs6550835 | -0.024809 | 0.3240 | 0.6852 | MALEPD | Intelligence || id:ebi-a-GCST006250 | 0.00126364 | 341.443562 | | 126 | rs6668048 | -0.021458 | 0.5063 | 0.5233 | MALEPD | Intelligence || id:ebi-a-GCST006250 | 0.001353417 | 365.7349824 | | 127 | rs66954617 | 0.0208823 | 0.6217 | 0.0184 | MALEPD | Intelligence || id:ebi-a-GCST006250 | 0.001305911 | 352.8804393 | | 128 | rs67482514 | 0.0178589 | 0.2436 | 0.2450 | MALEPD | Intelligence || id:ebi-a-GCST006250 | 0.001146099 | 309.6469964 | | 129 | rs6770622 | -0.044961 | 0.0384 | 0.6990 | MALEPD | Intelligence || id:ebi-a-GCST006250 | 0.000539534 | 145.6799866 | | 130 | rs6819372 | 0.0197961 | 0.5298 | 0.8538 | MALEPD | Intelligence || id:ebi-a-GCST006250 | 0.001356186 | 366.4842498 | | 131 | rs6860963 | 0.0202625 | 0.1899 | 0.6840 | MALEPD | Intelligence || id:ebi-a-GCST006250 | 0.001064851 | 287.6724505 | | 132 | rs6903716 | -0.017758 | 0.2979 | 0.4332 | MALEPD | Intelligence || id:ebi-a-GCST006250 | 0.001243986 | 336.1263135 | | 133 | rs702222 | -0.019831 | 0.3552 | 0.9837 | MALEPD | Intelligence || id:ebi-a-GCST006250 | 0.001288586 | 348.1928964 | | 134 | rs7069887 | -0.022532 | 0.1541 | 0.4390 | MALEPD | Intelligence || id:ebi-a-GCST006250 | 0.000949837 | 256.5715093 | | 135 | rs7116046 | 0.0157066 | 0.3882 | 0.4394 | MALEPD | Intelligence || id:ebi-a-GCST006250 | 0.001302006 | 351.8238999 | | 136 | rs7248006 | 0.019175 | 0.6214 | 0.9222 | MALEPD | Intelligence || id:ebi-a-GCST006250 | 0.001312168 | 354.5733525 | | 137 | rs72739469 | 0.0343573 | 0.0588 | 0.0392 | MALEPD | Intelligence || id:ebi-a-GCST006250 | 0.000655637 | 177.0496909 | | 138 | rs72768642 | 0.0306239 | 0.0678 | 0.4273 | MALEPD | Intelligence || id:ebi-a-GCST006250 | 0.000685244 | 185.0501654 | | 139 | rs73068339 | 0.0188582 | 0.2761 | 0.3096 | MALEPD | Intelligence || id:ebi-a-GCST006250 | 0.001215216 | 328.3433278 | | 140 | rs7312919 | -0.018146 | 0.3508 | 0.2784 | MALEPD | Intelligence || id:ebi-a-GCST006250 | 0.001269597 | 343.0553383 | | 141 | rs7357604 | -0.015705 | 0.3985 | 0.8726 | MALEPD | Intelligence || id:ebi-a-GCST006250 | 0.001312302 | 354.609816 | | 142 | rs7573001 | -0.016251 | 0.3952 | 0.3453 | MALEPD | Intelligence || id:ebi-a-GCST006250 | 0.001294139 | 349.6954441 | | 143 | rs75973558 | -0.025636 | 0.1279 | 0.6131 | MALEPD | Intelligence || id:ebi-a-GCST006250 | 0.000829151 | 223.9444499 | | 144 | rs7640196 | -0.017454 | 0.2255 | 0.0618 | MALEPD | Intelligence || id:ebi-a-GCST006250 | 0.001173287 | 317.0010806 | | 145 | rs7652296 | -0.016533 | 0.4024 | 0.0691 | MALEPD | Intelligence || id:ebi-a-GCST006250 | 0.001321946 | 357.2193089 | | 146 | rs7731260 | 0.0152959 | 0.4898 | 0.2450 | MALEPD | Intelligence || id:ebi-a-GCST006250 | 0.001348512 | 364.4075378 | | 147 | rs78084033 | 0.0228755 | 0.1373 | 0.1486 | MALEPD | Intelligence || id:ebi-a-GCST006250 | 0.000914229 | 246.9440665 | | 148 | rs7941785 | -0.015512 | 0.6368 | 0.6190 | MALEPD | Intelligence || id:ebi-a-GCST006250 | 0.001302583 | 351.9799331 | | 149 | rs799444 | -0.018415 | 0.5456 | 0.8187 | MALEPD | Intelligence || id:ebi-a-GCST006250 | 0.001341166 | 362.4198915 | | 150 | rs8006700 | -0.018227 | 0.6915 | 0.5516 | MALEPD | Intelligence || id:ebi-a-GCST006250 | 0.001262925 | 341.2501413 | | 151 | rs80170948 | -0.045381 | 0.0427 | 0.2076 | MALEPD | Intelligence || id:ebi-a-GCST006250 | 0.000502005 | 135.5414908 | | 152 | rs8025964 | 0.0170306 | 0.4638 | 0.2660 | MALEPD | Intelligence || id:ebi-a-GCST006250 | 0.001346312 | 363.8122675 | | 153 | rs8051038 | 0.018924 | 0.7329 | 0.1714 | MALEPD | Intelligence || id:ebi-a-GCST006250 | 0.001176614 | 317.9010077 | | 154 | rs8054299 | 0.0230055 | 0.3331 | 0.2328 | MALEPD | Intelligence || id:ebi-a-GCST006250 | 0.00126442 | 341.6547106 | | 155 | rs889169 | 0.0160711 | 0.6161 | 0.8358 | MALEPD | Intelligence || id:ebi-a-GCST006250 | 0.001279646 | 345.774121 | | 156 | rs913264 | 0.0197245 | 0.2961 | 0.2053 | MALEPD | Intelligence || id:ebi-a-GCST006250 | 0.001223187 | 330.4995832 | | 157 | rs9384679 | -0.026724 | 0.3839 | 0.0463 | MALEPD | Intelligence || id:ebi-a-GCST006250 | 0.001329488 | 359.2598434 | | 158 | rs9503599 | 0.0171108 | 0.4413 | 0.2955 | MALEPD | Intelligence || id:ebi-a-GCST006250 | 0.001328782 | 359.0689234 | | 159 | rs9516855 | -0.033427 | 0.0533 | 0.4243 | MALEPD | Intelligence || id:ebi-a-GCST006250 | 0.000607449 | 164.0289397 | | 160 | rs9569206 | 0.0154126 | 0.3576 | 0.4263 | MALEPD | Intelligence || id:ebi-a-GCST006250 | 0.001310265 | 354.0586357 | | 161 | rs967569 | -0.017976 | 0.6402 | 0.2717 | MALEPD | Intelligence || id:ebi-a-GCST006250 | 0.001264312 | 341.6255309 | | 162 | rs9888986 | -0.023502 | 0.1129 | 0.0730 | MALEPD | Intelligence || id:ebi-a-GCST006250 | 0.000868632 | 234.6172283 | | 1 | rs1007934 | 0.0160767 | 0.4039 | 0.5241 | PDAOO | Intelligence || id:ebi-a-GCST006250 | 0.001318968 | 356.4133816 | | 2 | rs10189857 | -0.018996 | 0.4255 | 0.5668 | PDAOO | Intelligence || id:ebi-a-GCST006250 | 0.001345759 | 363.6627617 | | 3 | rs1054442 | 0.0214635 | 0.3730 | 0.3050 | PDAOO | Intelligence || id:ebi-a-GCST006250 | 0.001313987 | 355.0656127 | | 4 | rs10779271 | -0.016375 | 0.3108 | 0.3390 | PDAOO | Intelligence || id:ebi-a-GCST006250 | 0.001265171 | 341.8579395 | | 5 | rs10917152 | 0.0242133 | 0.1301 | 0.2721 | PDAOO | Intelligence || id:ebi-a-GCST006250 | 0.000914323 | 246.9696815 | | 6 | rs10954779 | -0.016377 | 0.5560 | 0.8244 | PDAOO | Intelligence || id:ebi-a-GCST006250 | 0.001339915 | 362.0813273 | | 7 | rs11076962 | -0.016936 | 0.2837 | 0.4549 | PDAOO | Intelligence || id:ebi-a-GCST006250 | 0.001216756 | 328.7600031 | | 8 | rs11079849 | 0.0165478 | 0.3288 | 0.5566 | PDAOO | Intelligence || id:ebi-a-GCST006250 | 0.001250421 | 337.8672945 | | 9 | rs11210871 | 0.0173454 | 0.6940 | 0.9992 | PDAOO | Intelligence || id:ebi-a-GCST006250 | 0.001264597 | 341.7025761 | | 10 | rs112780312 | -0.018284 | 0.2876 | 0.3354 | PDAOO | Intelligence || id:ebi-a-GCST006250 | 0.001194109 | 322.6334144 | | 11 | rs1145123 | -0.020557 | 0.4955 | 0.5947 | PDAOO | Intelligence || id:ebi-a-GCST006250 | 0.001335198 | 360.8049578 | | 12 | rs115064 | -0.016096 | 0.3879 | 0.8202 | PDAOO | Intelligence || id:ebi-a-GCST006250 | 0.001315078 | 355.3608677 | | 13 | rs11605348 | -0.016607 | 0.3504 | 0.0441 | PDAOO | Intelligence || id:ebi-a-GCST006250 | 0.001277987 | 345.3251568 | | 14 | rs11623436 | -0.015774 | 0.4603 | 0.8155 | PDAOO | Intelligence || id:ebi-a-GCST006250 | 0.001345627 | 363.6270573 | | 15 | rs11634187 | -0.022032 | 0.1538 | 0.1251 | PDAOO | Intelligence || id:ebi-a-GCST006250 | 0.000959754 | 259.252825 | | 16 | rs11646221 | 0.0177354 | 0.5769 | 0.7967 | PDAOO | Intelligence || id:ebi-a-GCST006250 | 0.001335184 | 360.8010524 | | 17 | rs11678106 | 0.0160859 | 0.5063 | 0.7692 | PDAOO | Intelligence || id:ebi-a-GCST006250 | 0.00134808 | 364.2907167 | | 18 | rs11720523 | 0.0183255 | 0.4335 | 0.1326 | PDAOO | Intelligence || id:ebi-a-GCST006250 | 0.001334395 | 360.5876862 | | 19 | rs11793831 | 0.0278338 | 0.4069 | 0.5424 | PDAOO | Intelligence || id:ebi-a-GCST006250 | 0.001319579 | 356.578599 | | 20 | rs11898362 | -0.017981 | 0.2894 | 0.6969 | PDAOO | Intelligence || id:ebi-a-GCST006250 | 0.001230453 | 332.4653449 | | 21 | rs12026245 | -0.01786 | 0.5038 | 0.8237 | PDAOO | Intelligence || id:ebi-a-GCST006250 | 0.00135568 | 366.3473031 | | 22 | rs12035012 | -0.026992 | 0.2241 | 0.5185 | PDAOO | Intelligence || id:ebi-a-GCST006250 | 0.001117474 | 301.9046062 | | 23 | rs12190777 | -0.01703 | 0.2821 | 0.3858 | PDAOO | Intelligence || id:ebi-a-GCST006250 | 0.001197067 | 323.433789 | | 24 | rs1233578 | 0.0237975 | 0.1545 | 0.6761 | PDAOO | Intelligence || id:ebi-a-GCST006250 | 0.000948621 | 256.2427839 | | 25 | rs12470949 | 0.0171675 | 0.7189 | 0.8045 | PDAOO | Intelligence || id:ebi-a-GCST006250 | 0.001225148 | 331.0302031 | | 26 | rs12535854 | 0.0182259 | 0.6663 | 0.4495 | PDAOO | Intelligence || id:ebi-a-GCST006250 | 0.001253267 | 338.6373096 | | 27 | rs12646225 | 0.0251275 | 0.1195 | 0.3079 | PDAOO | Intelligence || id:ebi-a-GCST006250 | 0.00087829 | 237.2281556 | | 28 | rs1280049 | -0.014993 | 0.5145 | 0.1192 | PDAOO | Intelligence || id:ebi-a-GCST006250 | 0.001356037 | 366.4439608 | | 29 | rs12886584 | -0.020517 | 0.1925 | 0.0345 | PDAOO | Intelligence || id:ebi-a-GCST006250 | 0.001035786 | 279.8121296 | | 30 | rs13024268 | -0.016661 | 0.3908 | 0.8024 | PDAOO | Intelligence || id:ebi-a-GCST006250 | 0.001285443 | 347.3426661 | | 31 | rs13071190 | -0.018095 | 0.3193 | 0.5864 | PDAOO | Intelligence || id:ebi-a-GCST006250 | 0.001268645 | 342.7977954 | | 32 | rs13165296 | -0.019636 | 0.1746 | 0.7051 | PDAOO | Intelligence || id:ebi-a-GCST006250 | 0.001051534 | 284.0710492 | | 33 | rs13212044 | -0.018368 | 0.2462 | 0.6878 | PDAOO | Intelligence || id:ebi-a-GCST006250 | 0.001141749 | 308.4702197 | | 34 | rs13223152 | -0.017645 | 0.4047 | 0.7293 | PDAOO | Intelligence || id:ebi-a-GCST006250 | 0.001329268 | 359.2004817 | | 35 | rs13253386 | 0.0201329 | 0.4912 | 0.1539 | PDAOO | Intelligence || id:ebi-a-GCST006250 | 0.001346561 | 363.8797838 | | 36 | rs13276212 | 0.0150707 | 0.5094 | 0.4194 | PDAOO | Intelligence || id:ebi-a-GCST006250 | 0.001343379 | 363.0185173 | | 37 | rs13395129 | 0.0168652 | 0.3295 | 0.2081 | PDAOO | Intelligence || id:ebi-a-GCST006250 | 0.001235026 | 333.702385 | | 38 | rs1362739 | 0.0209452 | 0.4705 | 0.1454 | PDAOO | Intelligence || id:ebi-a-GCST006250 | 0.001353689 | 365.8085669 | | 39 | rs1369429 | -0.017633 | 0.6800 | 0.9023 | PDAOO | Intelligence || id:ebi-a-GCST006250 | 0.001277775 | 345.2679261 | | 40 | rs1408579 | 0.0160494 | 0.4723 | 0.5804 | PDAOO | Intelligence || id:ebi-a-GCST006250 | 0.001346537 | 363.8731634 | | 41 | rs144026674 | 0.0413066 | 0.0469 | 0.1201 | PDAOO | Intelligence || id:ebi-a-GCST006250 | 0.000495928 | 133.9000815 | | 42 | rs144246 | 0.0154911 | 0.3677 | 0.0164 | PDAOO | Intelligence || id:ebi-a-GCST006250 | 0.00130316 | 352.1361048 | | 43 | rs1589652 | -0.017094 | 0.5637 | 0.0754 | PDAOO | Intelligence || id:ebi-a-GCST006250 | 0.001341132 | 362.4106973 | | 44 | rs166820 | 0.0243341 | 0.1634 | 0.4268 | PDAOO | Intelligence || id:ebi-a-GCST006250 | 0.001028489 | 277.8390047 | | 45 | rs17002025 | 0.0255982 | 0.1212 | 0.4322 | PDAOO | Intelligence || id:ebi-a-GCST006250 | 0.000868801 | 234.6629251 | | 46 | rs17106817 | -0.016911 | 0.2811 | 0.3305 | PDAOO | Intelligence || id:ebi-a-GCST006250 | 0.001223591 | 330.6088501 | | 47 | rs17128425 | 0.0255567 | 0.0980 | 0.0746 | PDAOO | Intelligence || id:ebi-a-GCST006250 | 0.000814773 | 220.0581374 | | 48 | rs17199964 | -0.039187 | 0.0704 | 0.3157 | PDAOO | Intelligence || id:ebi-a-GCST006250 | 0.000648641 | 175.1591927 | | 49 | rs1727307 | -0.017817 | 0.7157 | 0.9362 | PDAOO | Intelligence || id:ebi-a-GCST006250 | 0.00123096 | 332.6024636 | | 50 | rs17698176 | 0.0201149 | 0.2244 | 0.4534 | PDAOO | Intelligence || id:ebi-a-GCST006250 | 0.001037909 | 280.3864283 | | 51 | rs1812587 | -0.017339 | 0.4613 | 0.8140 | PDAOO | Intelligence || id:ebi-a-GCST006250 | 0.00133754 | 361.4387498 | | 52 | rs1831539 | 0.0172035 | 0.4781 | 0.0607 | PDAOO | Intelligence || id:ebi-a-GCST006250 | 0.001339678 | 362.0170978 | | 53 | rs1840847 | 0.0163419 | 0.3578 | 0.7437 | PDAOO | Intelligence || id:ebi-a-GCST006250 | 0.001283796 | 346.8968422 | | 54 | rs1906252 | 0.0316616 | 0.4852 | 0.4252 | PDAOO | Intelligence || id:ebi-a-GCST006250 | 0.001349818 | 364.7611121 | | 55 | rs190925241 | 0.0334286 | 0.0804 | 0.1531 | PDAOO | Intelligence || id:ebi-a-GCST006250 | 0.000677162 | 182.8661536 | | 56 | rs1962047 | -0.019533 | 0.3680 | 0.5811 | PDAOO | Intelligence || id:ebi-a-GCST006250 | 0.001292713 | 349.3094412 | | 57 | rs1972860 | -0.017556 | 0.3195 | 0.7922 | PDAOO | Intelligence || id:ebi-a-GCST006250 | 0.001262912 | 341.2466477 | | 58 | rs2007176 | -0.015401 | 0.4639 | 0.6718 | PDAOO | Intelligence || id:ebi-a-GCST006250 | 0.001336966 | 361.2833563 | | 59 | rs2071407 | 0.0219741 | 0.6300 | 0.2734 | PDAOO | Intelligence || id:ebi-a-GCST006250 | 0.001294424 | 349.7725024 | | 60 | rs2072490 | 0.0169959 | 0.5159 | 0.1071 | PDAOO | Intelligence || id:ebi-a-GCST006250 | 0.001348193 | 364.3212423 | | 61 | rs2111490 | -0.015491 | 0.5314 | 0.9901 | PDAOO | Intelligence || id:ebi-a-GCST006250 | 0.001344207 | 363.2426875 | | 62 | rs2239647 | 0.0205367 | 0.5404 | 0.5118 | PDAOO | Intelligence || id:ebi-a-GCST006250 | 0.001337753 | 361.49624 | | 63 | rs2268894 | 0.0207848 | 0.5442 | 0.0057 | PDAOO | Intelligence || id:ebi-a-GCST006250 | 0.001346346 | 363.8215329 | | 64 | rs2285640 | 0.0175137 | 0.5273 | 0.3831 | PDAOO | Intelligence || id:ebi-a-GCST006250 | 0.00133856 | 361.7146083 | | 65 | rs2309812 | 0.0228352 | 0.3817 | 0.0734 | PDAOO | Intelligence || id:ebi-a-GCST006250 | 0.001300872 | 351.5171907 | | 66 | rs2352974 | -0.030841 | 0.4653 | 0.8756 | PDAOO | Intelligence || id:ebi-a-GCST006250 | 0.001344949 | 363.4433565 | | 67 | rs2373353 | 0.0163232 | 0.3684 | 0.4839 | PDAOO | Intelligence || id:ebi-a-GCST006250 | 0.001282099 | 346.4377582 | | 68 | rs2393967 | 0.0187089 | 0.3210 | 0.6187 | PDAOO | Intelligence || id:ebi-a-GCST006250 | 0.001249198 | 337.5365686 | | 69 | rs2420551 | -0.028596 | 0.8945 | 0.7195 | PDAOO | Intelligence || id:ebi-a-GCST006250 | 0.000852321 | 230.2077615 | | 70 | rs2450333 | -0.018829 | 0.5283 | 0.7031 | PDAOO | Intelligence || id:ebi-a-GCST006250 | 0.00132214 | 357.2716353 | | 71 | rs2457192 | -0.019751 | 0.6976 | 0.2973 | PDAOO | Intelligence || id:ebi-a-GCST006250 | 0.001182056 | 319.37319 | | 72 | rs2478286 | -0.025786 | 0.7412 | 0.7409 | PDAOO | Intelligence || id:ebi-a-GCST006250 | 0.001183597 | 319.7898929 | | 73 | rs2508713 | 0.0165308 | 0.3751 | 0.0415 | PDAOO | Intelligence || id:ebi-a-GCST006250 | 0.001302683 | 352.0071912 | | 74 | rs2558096 | 0.0156318 | 0.5840 | 0.1510 | PDAOO | Intelligence || id:ebi-a-GCST006250 | 0.001334236 | 360.5447832 | | 75 | rs2647995 | 0.0197491 | 0.2782 | 0.3760 | PDAOO | Intelligence || id:ebi-a-GCST006250 | 0.001215862 | 328.5180732 | | 76 | rs2678210 | -0.018786 | 0.2972 | 0.3759 | PDAOO | Intelligence || id:ebi-a-GCST006250 | 0.001214933 | 328.2668005 | | 77 | rs2721173 | -0.016233 | 0.4778 | 0.0977 | PDAOO | Intelligence || id:ebi-a-GCST006250 | 0.00135363 | 365.7925096 | | 78 | rs2726491 | -0.02828 | 0.3679 | 0.5224 | PDAOO | Intelligence || id:ebi-a-GCST006250 | 0.00129551 | 350.0663692 | | 79 | rs2836921 | 0.0203465 | 0.3365 | 0.5076 | PDAOO | Intelligence || id:ebi-a-GCST006250 | 0.001249068 | 337.5012535 | | 80 | rs28620532 | 0.0163543 | 0.3510 | 0.2528 | PDAOO | Intelligence || id:ebi-a-GCST006250 | 0.001280792 | 346.0840606 | | 81 | rs287879 | 0.0188666 | 0.2857 | 0.2436 | PDAOO | Intelligence || id:ebi-a-GCST006250 | 0.001203697 | 325.2272832 | | 82 | rs2885208 | -0.018939 | 0.1867 | 0.7732 | PDAOO | Intelligence || id:ebi-a-GCST006250 | 0.001068536 | 288.6689632 | | 83 | rs2920940 | 0.0247422 | 0.7628 | 0.2667 | PDAOO | Intelligence || id:ebi-a-GCST006250 | 0.001138266 | 307.5282202 | | 84 | rs2955280 | -0.014915 | 0.5059 | 0.1254 | PDAOO | Intelligence || id:ebi-a-GCST006250 | 0.001353452 | 365.7443461 | | 85 | rs297578 | 0.0181036 | 0.6991 | 0.4396 | PDAOO | Intelligence || id:ebi-a-GCST006250 | 0.001230891 | 332.5836584 | | 86 | rs2987390 | 0.0178028 | 0.2691 | 0.8396 | PDAOO | Intelligence || id:ebi-a-GCST006250 | 0.001185221 | 320.2292182 | | 87 | rs3128341 | 0.031727 | 0.7994 | 0.4305 | PDAOO | Intelligence || id:ebi-a-GCST006250 | 0.001083141 | 292.6188082 | | 88 | rs31768 | -0.018177 | 0.6970 | 0.8601 | PDAOO | Intelligence || id:ebi-a-GCST006250 | 0.001211894 | 327.4445023 | | 89 | rs329672 | 0.0174305 | 0.6347 | 0.3161 | PDAOO | Intelligence || id:ebi-a-GCST006250 | 0.00129702 | 350.4749282 | | 90 | rs34316 | -0.021049 | 0.5767 | 0.3702 | PDAOO | Intelligence || id:ebi-a-GCST006250 | 0.001337188 | 361.3434085 | | 91 | rs34320898 | 0.0228693 | 0.1903 | 0.8486 | PDAOO | Intelligence || id:ebi-a-GCST006250 | 0.000962991 | 260.1281892 | | 92 | rs34811474 | 0.0289955 | 0.2221 | 0.1213 | PDAOO | Intelligence || id:ebi-a-GCST006250 | 0.001030002 | 278.2479678 | | 93 | rs35608616 | -0.018089 | 0.3534 | 0.4678 | PDAOO | Intelligence || id:ebi-a-GCST006250 | 0.001260056 | 340.4740077 | | 94 | rs35731967 | -0.021838 | 0.1849 | 0.7313 | PDAOO | Intelligence || id:ebi-a-GCST006250 | 0.001011998 | 273.3796229 | | 95 | rs36033 | -0.015969 | 0.4260 | 0.6312 | PDAOO | Intelligence || id:ebi-a-GCST006250 | 0.00132743 | 358.7031311 | | 96 | rs3740422 | -0.024102 | 0.3489 | 0.7817 | PDAOO | Intelligence || id:ebi-a-GCST006250 | 0.001271078 | 343.4559442 | | 97 | rs3843954 | -0.020758 | 0.2634 | 0.6315 | PDAOO | Intelligence || id:ebi-a-GCST006250 | 0.001107346 | 299.1651999 | | 98 | rs3860537 | -0.018854 | 0.7811 | 0.3362 | PDAOO | Intelligence || id:ebi-a-GCST006250 | 0.001088045 | 293.9451521 | | 99 | rs405321 | -0.016432 | 0.3147 | 0.7152 | PDAOO | Intelligence || id:ebi-a-GCST006250 | 0.001244119 | 336.1624715 | | 100 | rs4463213 | 0.0190655 | 0.5378 | 0.7135 | PDAOO | Intelligence || id:ebi-a-GCST006250 | 0.001354392 | 365.9986857 | | 101 | rs4484297 | 0.0182667 | 0.2554 | 0.7043 | PDAOO | Intelligence || id:ebi-a-GCST006250 | 0.001171144 | 316.4213083 | | 102 | rs4667954 | -0.017315 | 0.2795 | 0.5725 | PDAOO | Intelligence || id:ebi-a-GCST006250 | 0.001218329 | 329.1853225 | | 103 | rs4731392 | 0.0217412 | 0.3114 | 0.7289 | PDAOO | Intelligence || id:ebi-a-GCST006250 | 0.001244182 | 336.1794233 | | 104 | rs4793161 | 0.0177187 | 0.7717 | 0.8250 | PDAOO | Intelligence || id:ebi-a-GCST006250 | 0.001138882 | 307.6947611 | | 105 | rs4821995 | -0.015999 | 0.6564 | 0.9793 | PDAOO | Intelligence || id:ebi-a-GCST006250 | 0.001287243 | 347.8295584 | | 106 | rs4852252 | 0.0207869 | 0.5683 | 0.5220 | PDAOO | Intelligence || id:ebi-a-GCST006250 | 0.001346919 | 363.9764684 | | 107 | rs4976976 | 0.0173172 | 0.4044 | 0.8345 | PDAOO | Intelligence || id:ebi-a-GCST006250 | 0.001332385 | 360.0437056 | | 108 | rs4981713 | -0.016189 | 0.3892 | 0.3288 | PDAOO | Intelligence || id:ebi-a-GCST006250 | 0.001322645 | 357.4082665 | | 109 | rs55754731 | -0.021369 | 0.1632 | 0.1933 | PDAOO | Intelligence || id:ebi-a-GCST006250 | 0.001007363 | 272.1260794 | | 110 | rs55763037 | -0.018345 | 0.2246 | 0.1037 | PDAOO | Intelligence || id:ebi-a-GCST006250 | 0.001110342 | 299.9755785 | | 111 | rs56150095 | -0.021968 | 0.5304 | 0.1352 | PDAOO | Intelligence || id:ebi-a-GCST006250 | 0.00134709 | 364.0228422 | | 112 | rs566237 | 0.0187161 | 0.3173 | 0.4067 | PDAOO | Intelligence || id:ebi-a-GCST006250 | 0.001260768 | 340.6665493 | | 113 | rs5750830 | 0.0228911 | 0.7234 | 0.6058 | PDAOO | Intelligence || id:ebi-a-GCST006250 | 0.001183695 | 319.8164844 | | 114 | rs58593843 | -0.027683 | 0.0751 | 0.6639 | PDAOO | Intelligence || id:ebi-a-GCST006250 | 0.000795952 | 214.9708044 | | 115 | rs59142272 | 0.0226956 | 0.1650 | 0.6768 | PDAOO | Intelligence || id:ebi-a-GCST006250 | 0.001004574 | 271.3720916 | | 116 | rs600806 | -0.019293 | 0.7284 | 0.8264 | PDAOO | Intelligence || id:ebi-a-GCST006250 | 0.001203209 | 325.0951199 | | 117 | rs6019535 | 0.0251054 | 0.3082 | 0.9857 | PDAOO | Intelligence || id:ebi-a-GCST006250 | 0.001243593 | 336.0201442 | | 118 | rs60262711 | 0.0159492 | 0.3766 | 0.6893 | PDAOO | Intelligence || id:ebi-a-GCST006250 | 0.001309807 | 353.9345743 | | 119 | rs62181012 | -0.021146 | 0.1980 | 0.2446 | PDAOO | Intelligence || id:ebi-a-GCST006250 | 0.001054164 | 284.7821511 | | 120 | rs62198803 | 0.0190651 | 0.2454 | 0.4151 | PDAOO | Intelligence || id:ebi-a-GCST006250 | 0.001147551 | 310.0396509 | | 121 | rs6508220 | 0.022748 | 0.4793 | 0.0684 | PDAOO | Intelligence || id:ebi-a-GCST006250 | 0.001351823 | 365.3034375 | | 122 | rs6535809 | -0.019648 | 0.4894 | 0.6245 | PDAOO | Intelligence || id:ebi-a-GCST006250 | 0.001353447 | 365.7430084 | | 123 | rs6539284 | 0.0194809 | 0.4170 | 0.7200 | PDAOO | Intelligence || id:ebi-a-GCST006250 | 0.001308858 | 353.6779558 | | 124 | rs6550835 | -0.024809 | 0.3275 | 0.0751 | PDAOO | Intelligence || id:ebi-a-GCST006250 | 0.00126364 | 341.443562 | | 125 | rs6668048 | -0.021458 | 0.4957 | 0.6877 | PDAOO | Intelligence || id:ebi-a-GCST006250 | 0.001353417 | 365.7349824 | | 126 | rs66954617 | 0.0208823 | 0.6235 | 0.2638 | PDAOO | Intelligence || id:ebi-a-GCST006250 | 0.001305911 | 352.8804393 | | 127 | rs67482514 | 0.0178589 | 0.2481 | 0.8948 | PDAOO | Intelligence || id:ebi-a-GCST006250 | 0.001146099 | 309.6469964 | | 128 | rs6770622 | -0.044961 | 0.0380 | 0.1872 | PDAOO | Intelligence || id:ebi-a-GCST006250 | 0.000539534 | 145.6799866 | | 129 | rs6819372 | 0.0197961 | 0.5175 | 0.8637 | PDAOO | Intelligence || id:ebi-a-GCST006250 | 0.001356186 | 366.4842498 | | 130 | rs6860963 | 0.0202625 | 0.2016 | 0.4687 | PDAOO | Intelligence || id:ebi-a-GCST006250 | 0.001064851 | 287.6724505 | | 131 | rs6903716 | -0.017758 | 0.2980 | 0.7235 | PDAOO | Intelligence || id:ebi-a-GCST006250 | 0.001243986 | 336.1263135 | | 132 | rs702222 | -0.019831 | 0.3692 | 0.5594 | PDAOO | Intelligence || id:ebi-a-GCST006250 | 0.001288586 | 348.1928964 | | 133 | rs7069887 | -0.022532 | 0.1506 | 0.0255 | PDAOO | Intelligence || id:ebi-a-GCST006250 | 0.000949837 | 256.5715093 | | 134 | rs7116046 | 0.0157066 | 0.3877 | 0.6663 | PDAOO | Intelligence || id:ebi-a-GCST006250 | 0.001302006 | 351.8238999 | | 135 | rs7248006 | 0.019175 | 0.6128 | 0.4906 | PDAOO | Intelligence || id:ebi-a-GCST006250 | 0.001312168 | 354.5733525 | | 136 | rs72739469 | 0.0343573 | 0.0665 | 0.3302 | PDAOO | Intelligence || id:ebi-a-GCST006250 | 0.000655637 | 177.0496909 | | 137 | rs72768642 | 0.0306239 | 0.0684 | 0.2116 | PDAOO | Intelligence || id:ebi-a-GCST006250 | 0.000685244 | 185.0501654 | | 138 | rs73068339 | 0.0188582 | 0.2825 | 0.3532 | PDAOO | Intelligence || id:ebi-a-GCST006250 | 0.001215216 | 328.3433278 | | 139 | rs7312919 | -0.018146 | 0.3633 | 0.0579 | PDAOO | Intelligence || id:ebi-a-GCST006250 | 0.001269597 | 343.0553383 | | 140 | rs7357604 | -0.015705 | 0.3913 | 0.5608 | PDAOO | Intelligence || id:ebi-a-GCST006250 | 0.001312302 | 354.609816 | | 141 | rs7573001 | -0.016251 | 0.3885 | 0.1096 | PDAOO | Intelligence || id:ebi-a-GCST006250 | 0.001294139 | 349.6954441 | | 142 | rs75973558 | -0.025636 | 0.1230 | 0.5341 | PDAOO | Intelligence || id:ebi-a-GCST006250 | 0.000829151 | 223.9444499 | | 143 | rs7640196 | -0.017454 | 0.2367 | 0.8498 | PDAOO | Intelligence || id:ebi-a-GCST006250 | 0.001173287 | 317.0010806 | | 144 | rs7652296 | -0.016533 | 0.4073 | 0.3274 | PDAOO | Intelligence || id:ebi-a-GCST006250 | 0.001321946 | 357.2193089 | | 145 | rs7731260 | 0.0152959 | 0.4893 | 0.4468 | PDAOO | Intelligence || id:ebi-a-GCST006250 | 0.001348512 | 364.4075378 | | 146 | rs78084033 | 0.0228755 | 0.1382 | 0.6808 | PDAOO | Intelligence || id:ebi-a-GCST006250 | 0.000914229 | 246.9440665 | | 147 | rs7941785 | -0.015512 | 0.6375 | 0.7122 | PDAOO | Intelligence || id:ebi-a-GCST006250 | 0.001302583 | 351.9799331 | | 148 | rs799444 | -0.018415 | 0.5313 | 0.8033 | PDAOO | Intelligence || id:ebi-a-GCST006250 | 0.001341166 | 362.4198915 | | 149 | rs8006700 | -0.018227 | 0.7002 | 0.9154 | PDAOO | Intelligence || id:ebi-a-GCST006250 | 0.001262925 | 341.2501413 | | 150 | rs80170948 | -0.045381 | 0.0430 | 0.9566 | PDAOO | Intelligence || id:ebi-a-GCST006250 | 0.000502005 | 135.5414908 | | 151 | rs8025964 | 0.0170306 | 0.4642 | 0.1591 | PDAOO | Intelligence || id:ebi-a-GCST006250 | 0.001346312 | 363.8122675 | | 152 | rs8051038 | 0.018924 | 0.7344 | 0.2652 | PDAOO | Intelligence || id:ebi-a-GCST006250 | 0.001176614 | 317.9010077 | | 153 | rs8054299 | 0.0230055 | 0.3221 | 0.9289 | PDAOO | Intelligence || id:ebi-a-GCST006250 | 0.00126442 | 341.6547106 | | 154 | rs889169 | 0.0160711 | 0.6203 | 0.1566 | PDAOO | Intelligence || id:ebi-a-GCST006250 | 0.001279646 | 345.774121 | | 155 | rs913264 | 0.0197245 | 0.2920 | 0.3862 | PDAOO | Intelligence || id:ebi-a-GCST006250 | 0.001223187 | 330.4995832 | | 156 | rs9384679 | -0.026724 | 0.3842 | 0.8912 | PDAOO | Intelligence || id:ebi-a-GCST006250 | 0.001329488 | 359.2598434 | | 157 | rs9503599 | 0.0171108 | 0.4408 | 0.7154 | PDAOO | Intelligence || id:ebi-a-GCST006250 | 0.001328782 | 359.0689234 | | 158 | rs9516855 | -0.033427 | 0.0526 | 0.5567 | PDAOO | Intelligence || id:ebi-a-GCST006250 | 0.000607449 | 164.0289397 | | 159 | rs9569206 | 0.0154126 | 0.3540 | 0.7361 | PDAOO | Intelligence || id:ebi-a-GCST006250 | 0.001310265 | 354.0586357 | | 160 | rs967569 | -0.017976 | 0.6543 | 0.1237 | PDAOO | Intelligence || id:ebi-a-GCST006250 | 0.001264312 | 341.6255309 | | 161 | rs9888986 | -0.023502 | 0.1146 | 0.3057 | PDAOO | Intelligence || id:ebi-a-GCST006250 | 0.000868632 | 234.6172283 | | 1 | rs1007934 | 0.0160767 | 0.3929 | 0.8155 | PD || id:ieu-b-7 | Intelligence || id:ebi-a-GCST006250 | 0.001318968 | 356.4133816 | | 2 | rs10189857 | -0.018996 | 0.4171 | 0.0321 | PD || id:ieu-b-7 | Intelligence || id:ebi-a-GCST006250 | 0.001345759 | 363.6627617 | | 3 | rs10189912 | 0.0193366 | 0.3750 | 0.5545 | PD || id:ieu-b-7 | Intelligence || id:ebi-a-GCST006250 | 0.001297202 | 350.5240685 | | 4 | rs1054442 | 0.0214635 | 0.3707 | 0.0239 | PD || id:ieu-b-7 | Intelligence || id:ebi-a-GCST006250 | 0.001313987 | 355.0656127 | | 5 | rs10779271 | -0.016375 | 0.3146 | 0.4150 | PD || id:ieu-b-7 | Intelligence || id:ebi-a-GCST006250 | 0.001265171 | 341.8579395 | | 6 | rs10917152 | 0.0242133 | 0.1332 | 0.0005 | PD || id:ieu-b-7 | Intelligence || id:ebi-a-GCST006250 | 0.000914323 | 246.9696815 | | 7 | rs10917152 | 0.0242133 | 0.1332 | 0.0005 | PD || id:ieu-b-7 | Intelligence || id:ebi-a-GCST006250 | 0.000914323 | 246.9696815 | | 8 | rs11076962 | -0.016936 | 0.2832 | 0.8195 | PD || id:ieu-b-7 | Intelligence || id:ebi-a-GCST006250 | 0.001216756 | 328.7600031 | | 9 | rs11079849 | 0.0165478 | 0.3286 | 0.6129 | PD || id:ieu-b-7 | Intelligence || id:ebi-a-GCST006250 | 0.001250421 | 337.8672945 | | 10 | rs11210871 | 0.0173454 | 0.6881 | 0.6933 | PD || id:ieu-b-7 | Intelligence || id:ebi-a-GCST006250 | 0.001264597 | 341.7025761 | | 11 | rs112780312 | -0.018284 | 0.2860 | 0.7092 | PD || id:ieu-b-7 | Intelligence || id:ebi-a-GCST006250 | 0.001194109 | 322.6334144 | | 12 | rs1145123 | -0.020557 | 0.4757 | 0.9732 | PD || id:ieu-b-7 | Intelligence || id:ebi-a-GCST006250 | 0.001335198 | 360.8049578 | | 13 | rs115064 | -0.016096 | 0.3962 | 0.9812 | PD || id:ieu-b-7 | Intelligence || id:ebi-a-GCST006250 | 0.001315078 | 355.3608677 | | 14 | rs11605348 | -0.016607 | 0.3497 | 0.3953 | PD || id:ieu-b-7 | Intelligence || id:ebi-a-GCST006250 | 0.001277987 | 345.3251568 | | 15 | rs11623436 | -0.015774 | 0.4673 | 0.0756 | PD || id:ieu-b-7 | Intelligence || id:ebi-a-GCST006250 | 0.001345627 | 363.6270573 | | 16 | rs11634187 | -0.022032 | 0.1528 | 0.2460 | PD || id:ieu-b-7 | Intelligence || id:ebi-a-GCST006250 | 0.000959754 | 259.252825 | | 17 | rs11646221 | 0.0177354 | 0.5699 | 0.5606 | PD || id:ieu-b-7 | Intelligence || id:ebi-a-GCST006250 | 0.001335184 | 360.8010524 | | 18 | rs11678106 | 0.0160859 | 0.5049 | 0.0564 | PD || id:ieu-b-7 | Intelligence || id:ebi-a-GCST006250 | 0.00134808 | 364.2907167 | | 19 | rs11720523 | 0.0183255 | 0.4362 | 0.3680 | PD || id:ieu-b-7 | Intelligence || id:ebi-a-GCST006250 | 0.001334395 | 360.5876862 | | 20 | rs11793831 | 0.0278338 | 0.4025 | 0.4824 | PD || id:ieu-b-7 | Intelligence || id:ebi-a-GCST006250 | 0.001319579 | 356.578599 | | 21 | rs11898362 | -0.017981 | 0.2904 | 0.5591 | PD || id:ieu-b-7 | Intelligence || id:ebi-a-GCST006250 | 0.001230453 | 332.4653449 | | 22 | rs12026245 | -0.01786 | 0.4995 | 0.4439 | PD || id:ieu-b-7 | Intelligence || id:ebi-a-GCST006250 | 0.00135568 | 366.3473031 | | 23 | rs12035012 | -0.026992 | 0.2240 | 0.5965 | PD || id:ieu-b-7 | Intelligence || id:ebi-a-GCST006250 | 0.001117474 | 301.9046062 | | 24 | rs12190777 | -0.01703 | 0.2730 | 0.9189 | PD || id:ieu-b-7 | Intelligence || id:ebi-a-GCST006250 | 0.001197067 | 323.433789 | | 25 | rs1233578 | 0.0237975 | 0.1580 | 0.3266 | PD || id:ieu-b-7 | Intelligence || id:ebi-a-GCST006250 | 0.000948621 | 256.2427839 | | 26 | rs12470949 | 0.0171675 | 0.7217 | 0.1820 | PD || id:ieu-b-7 | Intelligence || id:ebi-a-GCST006250 | 0.001225148 | 331.0302031 | | 27 | rs12535854 | 0.0182259 | 0.6607 | 0.1383 | PD || id:ieu-b-7 | Intelligence || id:ebi-a-GCST006250 | 0.001253267 | 338.6373096 | | 28 | rs12646225 | 0.0251275 | 0.1133 | 0.1648 | PD || id:ieu-b-7 | Intelligence || id:ebi-a-GCST006250 | 0.00087829 | 237.2281556 | | 29 | rs1280049 | -0.014993 | 0.5110 | 0.5295 | PD || id:ieu-b-7 | Intelligence || id:ebi-a-GCST006250 | 0.001356037 | 366.4439608 | | 30 | rs12886584 | -0.020517 | 0.1891 | 0.0287 | PD || id:ieu-b-7 | Intelligence || id:ebi-a-GCST006250 | 0.001035786 | 279.8121296 | | 31 | rs13024268 | -0.016661 | 0.3805 | 0.5170 | PD || id:ieu-b-7 | Intelligence || id:ebi-a-GCST006250 | 0.001285443 | 347.3426661 | | 32 | rs13071190 | -0.018095 | 0.3194 | 0.7461 | PD || id:ieu-b-7 | Intelligence || id:ebi-a-GCST006250 | 0.001268645 | 342.7977954 | | 33 | rs13165296 | -0.019636 | 0.1745 | 0.5989 | PD || id:ieu-b-7 | Intelligence || id:ebi-a-GCST006250 | 0.001051534 | 284.0710492 | | 34 | rs13212044 | -0.018368 | 0.2497 | 0.1078 | PD || id:ieu-b-7 | Intelligence || id:ebi-a-GCST006250 | 0.001141749 | 308.4702197 | | 35 | rs13223152 | -0.017645 | 0.4007 | 0.6533 | PD || id:ieu-b-7 | Intelligence || id:ebi-a-GCST006250 | 0.001329268 | 359.2004817 | | 36 | rs13253386 | 0.0201329 | 0.4911 | 0.7910 | PD || id:ieu-b-7 | Intelligence || id:ebi-a-GCST006250 | 0.001346561 | 363.8797838 | | 37 | rs13276212 | 0.0150707 | 0.5106 | 0.0458 | PD || id:ieu-b-7 | Intelligence || id:ebi-a-GCST006250 | 0.001343379 | 363.0185173 | | 38 | rs13395129 | 0.0168652 | 0.3184 | 0.9641 | PD || id:ieu-b-7 | Intelligence || id:ebi-a-GCST006250 | 0.001235026 | 333.702385 | | 39 | rs1362739 | 0.0209452 | 0.4733 | 0.4361 | PD || id:ieu-b-7 | Intelligence || id:ebi-a-GCST006250 | 0.001353689 | 365.8085669 | | 40 | rs1369429 | -0.017633 | 0.6826 | 0.6979 | PD || id:ieu-b-7 | Intelligence || id:ebi-a-GCST006250 | 0.001277775 | 345.2679261 | | 41 | rs1408579 | 0.0160494 | 0.4763 | 0.8230 | PD || id:ieu-b-7 | Intelligence || id:ebi-a-GCST006250 | 0.001346537 | 363.8731634 | | 42 | rs144026674 | 0.0413066 | 0.0471 | 0.3652 | PD || id:ieu-b-7 | Intelligence || id:ebi-a-GCST006250 | 0.000495928 | 133.9000815 | | 43 | rs144246 | 0.0154911 | 0.3753 | 0.6545 | PD || id:ieu-b-7 | Intelligence || id:ebi-a-GCST006250 | 0.00130316 | 352.1361048 | | 44 | rs1589652 | -0.017094 | 0.5624 | 0.5484 | PD || id:ieu-b-7 | Intelligence || id:ebi-a-GCST006250 | 0.001341132 | 362.4106973 | | 45 | rs166820 | 0.0243341 | 0.1591 | 0.5240 | PD || id:ieu-b-7 | Intelligence || id:ebi-a-GCST006250 | 0.001028489 | 277.8390047 | | 46 | rs17002025 | 0.0255982 | 0.1215 | 0.9741 | PD || id:ieu-b-7 | Intelligence || id:ebi-a-GCST006250 | 0.000868801 | 234.6629251 | | 47 | rs17106817 | -0.016911 | 0.2848 | 0.8185 | PD || id:ieu-b-7 | Intelligence || id:ebi-a-GCST006250 | 0.001223591 | 330.6088501 | | 48 | rs17128425 | 0.0255567 | 0.0986 | 0.9988 | PD || id:ieu-b-7 | Intelligence || id:ebi-a-GCST006250 | 0.000814773 | 220.0581374 | | 49 | rs17199964 | -0.039187 | 0.0748 | 0.8363 | PD || id:ieu-b-7 | Intelligence || id:ebi-a-GCST006250 | 0.000648641 | 175.1591927 | | 50 | rs1727307 | -0.017817 | 0.7107 | 0.0883 | PD || id:ieu-b-7 | Intelligence || id:ebi-a-GCST006250 | 0.00123096 | 332.6024636 | | 51 | rs17698176 | 0.0201149 | 0.2195 | 0.0078 | PD || id:ieu-b-7 | Intelligence || id:ebi-a-GCST006250 | 0.001037909 | 280.3864283 | | 52 | rs1812587 | -0.017339 | 0.4568 | 0.3738 | PD || id:ieu-b-7 | Intelligence || id:ebi-a-GCST006250 | 0.00133754 | 361.4387498 | | 53 | rs1831539 | 0.0172035 | 0.4727 | 0.1439 | PD || id:ieu-b-7 | Intelligence || id:ebi-a-GCST006250 | 0.001339678 | 362.0170978 | | 54 | rs1840847 | 0.0163419 | 0.3447 | 0.1459 | PD || id:ieu-b-7 | Intelligence || id:ebi-a-GCST006250 | 0.001283796 | 346.8968422 | | 55 | rs1906252 | 0.0316616 | 0.4834 | 0.0175 | PD || id:ieu-b-7 | Intelligence || id:ebi-a-GCST006250 | 0.001349818 | 364.7611121 | | 56 | rs190925241 | 0.0334286 | 0.0837 | 0.8759 | PD || id:ieu-b-7 | Intelligence || id:ebi-a-GCST006250 | 0.000677162 | 182.8661536 | | 57 | rs190925241 | 0.0334286 | 0.0837 | 0.8759 | PD || id:ieu-b-7 | Intelligence || id:ebi-a-GCST006250 | 0.000677162 | 182.8661536 | | 58 | rs190925241 | 0.0334286 | 0.0837 | 0.8759 | PD || id:ieu-b-7 | Intelligence || id:ebi-a-GCST006250 | 0.000677162 | 182.8661536 | | 59 | rs1962047 | -0.019533 | 0.3559 | 0.0788 | PD || id:ieu-b-7 | Intelligence || id:ebi-a-GCST006250 | 0.001292713 | 349.3094412 | | 60 | rs1972860 | -0.017556 | 0.3201 | 0.9101 | PD || id:ieu-b-7 | Intelligence || id:ebi-a-GCST006250 | 0.001262912 | 341.2466477 | | 61 | rs2007176 | -0.015401 | 0.4557 | 0.1539 | PD || id:ieu-b-7 | Intelligence || id:ebi-a-GCST006250 | 0.001336966 | 361.2833563 | | 62 | rs2008514 | -0.028679 | 0.3739 | 0.0023 | PD || id:ieu-b-7 | Intelligence || id:ebi-a-GCST006250 | 0.001321998 | 357.2333462 | | 63 | rs2071407 | 0.0219741 | 0.6261 | 0.9218 | PD || id:ieu-b-7 | Intelligence || id:ebi-a-GCST006250 | 0.001294424 | 349.7725024 | | 64 | rs2072490 | 0.0169959 | 0.5145 | 0.7147 | PD || id:ieu-b-7 | Intelligence || id:ebi-a-GCST006250 | 0.001348193 | 364.3212423 | | 65 | rs2111490 | -0.015491 | 0.5310 | 0.5243 | PD || id:ieu-b-7 | Intelligence || id:ebi-a-GCST006250 | 0.001344207 | 363.2426875 | | 66 | rs2239647 | 0.0205367 | 0.5400 | 0.8608 | PD || id:ieu-b-7 | Intelligence || id:ebi-a-GCST006250 | 0.001337753 | 361.49624 | | 67 | rs2268894 | 0.0207848 | 0.5417 | 0.0073 | PD || id:ieu-b-7 | Intelligence || id:ebi-a-GCST006250 | 0.001346346 | 363.8215329 | | 68 | rs2285640 | 0.0175137 | 0.5341 | 0.1328 | PD || id:ieu-b-7 | Intelligence || id:ebi-a-GCST006250 | 0.00133856 | 361.7146083 | | 69 | rs2309812 | 0.0228352 | 0.3808 | 0.0480 | PD || id:ieu-b-7 | Intelligence || id:ebi-a-GCST006250 | 0.001300872 | 351.5171907 | | 70 | rs2352974 | -0.030841 | 0.4730 | 0.7076 | PD || id:ieu-b-7 | Intelligence || id:ebi-a-GCST006250 | 0.001344949 | 363.4433565 | | 71 | rs2373353 | 0.0163232 | 0.3648 | 0.1035 | PD || id:ieu-b-7 | Intelligence || id:ebi-a-GCST006250 | 0.001282099 | 346.4377582 | | 72 | rs2373353 | 0.0163232 | 0.3648 | 0.1035 | PD || id:ieu-b-7 | Intelligence || id:ebi-a-GCST006250 | 0.001282099 | 346.4377582 | | 73 | rs2450333 | -0.018829 | 0.5320 | 0.2091 | PD || id:ieu-b-7 | Intelligence || id:ebi-a-GCST006250 | 0.00132214 | 357.2716353 | | 74 | rs2457192 | -0.019751 | 0.7020 | 0.8922 | PD || id:ieu-b-7 | Intelligence || id:ebi-a-GCST006250 | 0.001182056 | 319.37319 | | 75 | rs2508713 | 0.0165308 | 0.3867 | 0.0369 | PD || id:ieu-b-7 | Intelligence || id:ebi-a-GCST006250 | 0.001302683 | 352.0071912 | | 76 | rs2647995 | 0.0197491 | 0.2796 | 0.3613 | PD || id:ieu-b-7 | Intelligence || id:ebi-a-GCST006250 | 0.001215862 | 328.5180732 | | 77 | rs2678210 | -0.018786 | 0.2940 | 0.2278 | PD || id:ieu-b-7 | Intelligence || id:ebi-a-GCST006250 | 0.001214933 | 328.2668005 | | 78 | rs2721173 | -0.016233 | 0.4715 | 0.0493 | PD || id:ieu-b-7 | Intelligence || id:ebi-a-GCST006250 | 0.00135363 | 365.7925096 | | 79 | rs2726491 | -0.02828 | 0.3643 | 0.6801 | PD || id:ieu-b-7 | Intelligence || id:ebi-a-GCST006250 | 0.00129551 | 350.0663692 | | 80 | rs2836921 | 0.0203465 | 0.3445 | 0.5007 | PD || id:ieu-b-7 | Intelligence || id:ebi-a-GCST006250 | 0.001249068 | 337.5012535 | | 81 | rs28620532 | 0.0163543 | 0.3506 | 0.0016 | PD || id:ieu-b-7 | Intelligence || id:ebi-a-GCST006250 | 0.001280792 | 346.0840606 | | 82 | rs287879 | 0.0188666 | 0.2920 | 0.3704 | PD || id:ieu-b-7 | Intelligence || id:ebi-a-GCST006250 | 0.001203697 | 325.2272832 | | 83 | rs2885208 | -0.018939 | 0.1828 | 0.4096 | PD || id:ieu-b-7 | Intelligence || id:ebi-a-GCST006250 | 0.001068536 | 288.6689632 | | 84 | rs2920940 | 0.0247422 | 0.7672 | 0.3018 | PD || id:ieu-b-7 | Intelligence || id:ebi-a-GCST006250 | 0.001138266 | 307.5282202 | | 85 | rs2955280 | -0.014915 | 0.5080 | 0.7556 | PD || id:ieu-b-7 | Intelligence || id:ebi-a-GCST006250 | 0.001353452 | 365.7443461 | | 86 | rs2987390 | 0.0178028 | 0.2733 | 0.5110 | PD || id:ieu-b-7 | Intelligence || id:ebi-a-GCST006250 | 0.001185221 | 320.2292182 | | 87 | rs3128341 | 0.031727 | 0.8070 | 0.6658 | PD || id:ieu-b-7 | Intelligence || id:ebi-a-GCST006250 | 0.001083141 | 292.6188082 | | 88 | rs31768 | -0.018177 | 0.6998 | 0.3484 | PD || id:ieu-b-7 | Intelligence || id:ebi-a-GCST006250 | 0.001211894 | 327.4445023 | | 89 | rs329672 | 0.0174305 | 0.6332 | 0.3722 | PD || id:ieu-b-7 | Intelligence || id:ebi-a-GCST006250 | 0.00129702 | 350.4749282 | | 90 | rs34316 | -0.021049 | 0.5752 | 0.4693 | PD || id:ieu-b-7 | Intelligence || id:ebi-a-GCST006250 | 0.001337188 | 361.3434085 | | 91 | rs34316 | -0.021049 | 0.5752 | 0.4693 | PD || id:ieu-b-7 | Intelligence || id:ebi-a-GCST006250 | 0.001337188 | 361.3434085 | | 92 | rs34320898 | 0.0228693 | 0.1904 | 0.1960 | PD || id:ieu-b-7 | Intelligence || id:ebi-a-GCST006250 | 0.000962991 | 260.1281892 | | 93 | rs34811474 | 0.0289955 | 0.2198 | 0.0098 | PD || id:ieu-b-7 | Intelligence || id:ebi-a-GCST006250 | 0.001030002 | 278.2479678 | | 94 | rs35608616 | -0.018089 | 0.3550 | 0.0811 | PD || id:ieu-b-7 | Intelligence || id:ebi-a-GCST006250 | 0.001260056 | 340.4740077 | | 95 | rs35731967 | -0.021838 | 0.1844 | 0.5602 | PD || id:ieu-b-7 | Intelligence || id:ebi-a-GCST006250 | 0.001011998 | 273.3796229 | | 96 | rs36033 | -0.015969 | 0.4184 | 0.6089 | PD || id:ieu-b-7 | Intelligence || id:ebi-a-GCST006250 | 0.00132743 | 358.7031311 | | 97 | rs3843954 | -0.020758 | 0.2730 | 0.4869 | PD || id:ieu-b-7 | Intelligence || id:ebi-a-GCST006250 | 0.001107346 | 299.1651999 | | 98 | rs3860537 | -0.018854 | 0.7811 | 0.6862 | PD || id:ieu-b-7 | Intelligence || id:ebi-a-GCST006250 | 0.001088045 | 293.9451521 | | 99 | rs405321 | -0.016432 | 0.3154 | 0.0048 | PD || id:ieu-b-7 | Intelligence || id:ebi-a-GCST006250 | 0.001244119 | 336.1624715 | | 100 | rs4463213 | 0.0190655 | 0.5414 | 0.0262 | PD || id:ieu-b-7 | Intelligence || id:ebi-a-GCST006250 | 0.001354392 | 365.9986857 | | 101 | rs4667954 | -0.017315 | 0.2813 | 0.8383 | PD || id:ieu-b-7 | Intelligence || id:ebi-a-GCST006250 | 0.001218329 | 329.1853225 | | 102 | rs4725065 | 0.0165317 | 0.5175 | 0.8535 | PD || id:ieu-b-7 | Intelligence || id:ebi-a-GCST006250 | 0.001352464 | 365.4770018 | | 103 | rs4731392 | 0.0217412 | 0.3175 | 0.3699 | PD || id:ieu-b-7 | Intelligence || id:ebi-a-GCST006250 | 0.001244182 | 336.1794233 | | 104 | rs4793161 | 0.0177187 | 0.7745 | 0.7615 | PD || id:ieu-b-7 | Intelligence || id:ebi-a-GCST006250 | 0.001138882 | 307.6947611 | | 105 | rs4821995 | -0.015999 | 0.6587 | 0.1248 | PD || id:ieu-b-7 | Intelligence || id:ebi-a-GCST006250 | 0.001287243 | 347.8295584 | | 106 | rs4852252 | 0.0207869 | 0.5691 | 0.5953 | PD || id:ieu-b-7 | Intelligence || id:ebi-a-GCST006250 | 0.001346919 | 363.9764684 | | 107 | rs4976976 | 0.0173172 | 0.4008 | 0.4293 | PD || id:ieu-b-7 | Intelligence || id:ebi-a-GCST006250 | 0.001332385 | 360.0437056 | | 108 | rs4981713 | -0.016189 | 0.3840 | 0.3858 | PD || id:ieu-b-7 | Intelligence || id:ebi-a-GCST006250 | 0.001322645 | 357.4082665 | | 109 | rs55754731 | -0.021369 | 0.1692 | 0.9731 | PD || id:ieu-b-7 | Intelligence || id:ebi-a-GCST006250 | 0.001007363 | 272.1260794 | | 110 | rs55763037 | -0.018345 | 0.2126 | 0.4983 | PD || id:ieu-b-7 | Intelligence || id:ebi-a-GCST006250 | 0.001110342 | 299.9755785 | | 111 | rs56150095 | -0.021968 | 0.5324 | 0.6812 | PD || id:ieu-b-7 | Intelligence || id:ebi-a-GCST006250 | 0.00134709 | 364.0228422 | | 112 | rs566237 | 0.0187161 | 0.3180 | 0.0415 | PD || id:ieu-b-7 | Intelligence || id:ebi-a-GCST006250 | 0.001260768 | 340.6665493 | | 113 | rs5750830 | 0.0228911 | 0.7221 | 0.1026 | PD || id:ieu-b-7 | Intelligence || id:ebi-a-GCST006250 | 0.001183695 | 319.8164844 | | 114 | rs58593843 | -0.027683 | 0.0727 | 0.5060 | PD || id:ieu-b-7 | Intelligence || id:ebi-a-GCST006250 | 0.000795952 | 214.9708044 | | 115 | rs59142272 | 0.0226956 | 0.1702 | 0.3554 | PD || id:ieu-b-7 | Intelligence || id:ebi-a-GCST006250 | 0.001004574 | 271.3720916 | | 116 | rs600806 | -0.019293 | 0.7347 | 0.8547 | PD || id:ieu-b-7 | Intelligence || id:ebi-a-GCST006250 | 0.001203209 | 325.0951199 | | 117 | rs6019535 | 0.0251054 | 0.3095 | 0.0456 | PD || id:ieu-b-7 | Intelligence || id:ebi-a-GCST006250 | 0.001243593 | 336.0201442 | | 118 | rs62181012 | -0.021146 | 0.1961 | 0.9179 | PD || id:ieu-b-7 | Intelligence || id:ebi-a-GCST006250 | 0.001054164 | 284.7821511 | | 119 | rs62198803 | 0.0190651 | 0.2436 | 0.4571 | PD || id:ieu-b-7 | Intelligence || id:ebi-a-GCST006250 | 0.001147551 | 310.0396509 | | 120 | rs6508220 | 0.022748 | 0.4860 | 0.3351 | PD || id:ieu-b-7 | Intelligence || id:ebi-a-GCST006250 | 0.001351823 | 365.3034375 | | 121 | rs6535809 | -0.019648 | 0.4889 | 0.9160 | PD || id:ieu-b-7 | Intelligence || id:ebi-a-GCST006250 | 0.001353447 | 365.7430084 | | 122 | rs6539284 | 0.0194809 | 0.4155 | 0.6016 | PD || id:ieu-b-7 | Intelligence || id:ebi-a-GCST006250 | 0.001308858 | 353.6779558 | | 123 | rs6550835 | -0.024809 | 0.3273 | 0.6564 | PD || id:ieu-b-7 | Intelligence || id:ebi-a-GCST006250 | 0.00126364 | 341.443562 | | 124 | rs6668048 | -0.021458 | 0.5019 | 0.0567 | PD || id:ieu-b-7 | Intelligence || id:ebi-a-GCST006250 | 0.001353417 | 365.7349824 | | 125 | rs67482514 | 0.0178589 | 0.2430 | 0.6624 | PD || id:ieu-b-7 | Intelligence || id:ebi-a-GCST006250 | 0.001146099 | 309.6469964 | | 126 | rs6770622 | -0.044961 | 0.0382 | 0.4350 | PD || id:ieu-b-7 | Intelligence || id:ebi-a-GCST006250 | 0.000539534 | 145.6799866 | | 127 | rs6819372 | 0.0197961 | 0.5273 | 0.5261 | PD || id:ieu-b-7 | Intelligence || id:ebi-a-GCST006250 | 0.001356186 | 366.4842498 | | 128 | rs6860963 | 0.0202625 | 0.1921 | 0.0070 | PD || id:ieu-b-7 | Intelligence || id:ebi-a-GCST006250 | 0.001064851 | 287.6724505 | | 129 | rs6903716 | -0.017758 | 0.2999 | 0.9128 | PD || id:ieu-b-7 | Intelligence || id:ebi-a-GCST006250 | 0.001243986 | 336.1263135 | | 130 | rs702222 | -0.019831 | 0.3606 | 0.8869 | PD || id:ieu-b-7 | Intelligence || id:ebi-a-GCST006250 | 0.001288586 | 348.1928964 | | 131 | rs7069887 | -0.022532 | 0.1499 | 0.9684 | PD || id:ieu-b-7 | Intelligence || id:ebi-a-GCST006250 | 0.000949837 | 256.5715093 | | 132 | rs7116046 | 0.0157066 | 0.3874 | 0.6630 | PD || id:ieu-b-7 | Intelligence || id:ebi-a-GCST006250 | 0.001302006 | 351.8238999 | | 133 | rs7172979 | 0.0606345 | 0.0317 | 0.7649 | PD || id:ieu-b-7 | Intelligence || id:ebi-a-GCST006250 | 0.00040776 | 110.0850291 | | 134 | rs7248006 | 0.019175 | 0.6195 | 0.2373 | PD || id:ieu-b-7 | Intelligence || id:ebi-a-GCST006250 | 0.001312168 | 354.5733525 | | 135 | rs72739469 | 0.0343573 | 0.0608 | 0.0750 | PD || id:ieu-b-7 | Intelligence || id:ebi-a-GCST006250 | 0.000655637 | 177.0496909 | | 136 | rs72768642 | 0.0306239 | 0.0678 | 0.5491 | PD || id:ieu-b-7 | Intelligence || id:ebi-a-GCST006250 | 0.000685244 | 185.0501654 | | 137 | rs73068339 | 0.0188582 | 0.2748 | 0.6284 | PD || id:ieu-b-7 | Intelligence || id:ebi-a-GCST006250 | 0.001215216 | 328.3433278 | | 138 | rs7312919 | -0.018146 | 0.3531 | 0.3213 | PD || id:ieu-b-7 | Intelligence || id:ebi-a-GCST006250 | 0.001269597 | 343.0553383 | | 139 | rs7357604 | -0.015705 | 0.3949 | 0.9269 | PD || id:ieu-b-7 | Intelligence || id:ebi-a-GCST006250 | 0.001312302 | 354.609816 | | 140 | rs7573001 | -0.016251 | 0.3944 | 0.5234 | PD || id:ieu-b-7 | Intelligence || id:ebi-a-GCST006250 | 0.001294139 | 349.6954441 | | 141 | rs75973558 | -0.025636 | 0.1241 | 0.6983 | PD || id:ieu-b-7 | Intelligence || id:ebi-a-GCST006250 | 0.000829151 | 223.9444499 | | 142 | rs7640196 | -0.017454 | 0.2307 | 0.4891 | PD || id:ieu-b-7 | Intelligence || id:ebi-a-GCST006250 | 0.001173287 | 317.0010806 | | 143 | rs7652296 | -0.016533 | 0.4012 | 0.1192 | PD || id:ieu-b-7 | Intelligence || id:ebi-a-GCST006250 | 0.001321946 | 357.2193089 | | 144 | rs7731260 | 0.0152959 | 0.4940 | 0.2281 | PD || id:ieu-b-7 | Intelligence || id:ebi-a-GCST006250 | 0.001348512 | 364.4075378 | | 145 | rs78084033 | 0.0228755 | 0.1376 | 0.1632 | PD || id:ieu-b-7 | Intelligence || id:ebi-a-GCST006250 | 0.000914229 | 246.9440665 | | 146 | rs7941785 | -0.015512 | 0.6366 | 0.4090 | PD || id:ieu-b-7 | Intelligence || id:ebi-a-GCST006250 | 0.001302583 | 351.9799331 | | 147 | rs799444 | -0.018415 | 0.5373 | 0.5176 | PD || id:ieu-b-7 | Intelligence || id:ebi-a-GCST006250 | 0.001341166 | 362.4198915 | | 148 | rs8006700 | -0.018227 | 0.6919 | 0.4721 | PD || id:ieu-b-7 | Intelligence || id:ebi-a-GCST006250 | 0.001262925 | 341.2501413 | | 149 | rs8051038 | 0.018924 | 0.7369 | 0.2413 | PD || id:ieu-b-7 | Intelligence || id:ebi-a-GCST006250 | 0.001176614 | 317.9010077 | | 150 | rs8054299 | 0.0230055 | 0.3321 | 0.6706 | PD || id:ieu-b-7 | Intelligence || id:ebi-a-GCST006250 | 0.00126442 | 341.6547106 | | 151 | rs889169 | 0.0160711 | 0.6154 | 0.2107 | PD || id:ieu-b-7 | Intelligence || id:ebi-a-GCST006250 | 0.001279646 | 345.774121 | | 152 | rs913264 | 0.0197245 | 0.2957 | 0.1895 | PD || id:ieu-b-7 | Intelligence || id:ebi-a-GCST006250 | 0.001223187 | 330.4995832 | | 153 | rs9384679 | -0.026724 | 0.3909 | 0.7069 | PD || id:ieu-b-7 | Intelligence || id:ebi-a-GCST006250 | 0.001329488 | 359.2598434 | | 154 | rs9503599 | 0.0171108 | 0.4427 | 0.8297 | PD || id:ieu-b-7 | Intelligence || id:ebi-a-GCST006250 | 0.001328782 | 359.0689234 | | 155 | rs9516855 | -0.033427 | 0.0527 | 0.1018 | PD || id:ieu-b-7 | Intelligence || id:ebi-a-GCST006250 | 0.000607449 | 164.0289397 | | 156 | rs9569206 | 0.0154126 | 0.3590 | 0.2418 | PD || id:ieu-b-7 | Intelligence || id:ebi-a-GCST006250 | 0.001310265 | 354.0586357 | | 157 | rs967569 | -0.017976 | 0.6438 | 0.7072 | PD || id:ieu-b-7 | Intelligence || id:ebi-a-GCST006250 | 0.001264312 | 341.6255309 | | 158 | rs9888986 | -0.023502 | 0.1126 | 0.8675 | PD || id:ieu-b-7 | Intelligence || id:ebi-a-GCST006250 | 0.000868632 | 234.6172283 | |
[truncated: 146,604 more chars]
