# Supplementary material for: Intelligence, education level, and risk of Parkinson’s disease in European populations: A Mendelian randomization study
Source: Front Genet. 2022 Nov 10;13:963163. doi: 10.3389/fgene.2022.963163 (PMC9684183; doi:10.3389/fgene.2022.963163)
Supplement: Supplementary file 2 [file DataSheet1.docx]

**Fig 1. Scatter plots of single-nucleotide polymorphism (SNP) associated with intelligence and Parkinson’s Disease.**

**Fig 2. Scatter plots of single-nucleotide polymorphism (SNP) associated with educational attainment and Parkinson’s Disease.**

**Fig 3. Scatter plots of single-nucleotide polymorphism (SNP) associated with cognitive (test) performance and Parkinson’s Disease.**

**Fig 4. Scatter plots of single-nucleotide polymorphism (SNP) associated with intelligence and MALEPD.**

**Fig 5. Scatter plots of single-nucleotide polymorphism (SNP) associated with educational attainment and MALEPD.**

**Fig 6. Scatter plots of single-nucleotide polymorphism (SNP) associated with cognitive (test) performance and MALEPD.**

**Fig 7. Scatter plots of single-nucleotide polymorphism (SNP) associated with intelligence and FEMALEPD.**

**Fig 8. Scatter plots of single-nucleotide polymorphism (SNP) associated with educational attainment and FEMALEPD.**

**Fig 9. Scatter plots of single-nucleotide polymorphism (SNP) associated with cognitive (test) performance and FEMALEPD.**

**Fig 10. Scatter plots of single-nucleotide polymorphism (SNP) associated with intelligence and PDAOO.**

**Fig 11. Scatter plots of single-nucleotide polymorphism (SNP) associated with educational attainment and PDAOO.**

**Fig 12. Scatter plots of single-nucleotide polymorphism (SNP) associated with cognitive (test) performance and PDAOO.**

**Fig 13. Single SNP analysis of the association between intelligence and Parkinson’s Disease.**

**Fig 14. Single SNP analysis of the association between educational attainment and Parkinson’s Disease.**

**Fig 15. Single SNP analysis of the association between cognitive (test) performance and Parkinson’s Disease.**

**Fig 16. Single SNP analysis of the association between intelligence and MALEPD.**

**Fig 17. Scatter plots of single-nucleotide polymorphism (SNP) associated with educational attainment and MALEPD.**

**Fig 18. Single SNP analysis of the association between cognitive (test) performance and MALEPD.**

**Fig 19. Single SNP analysis of the association between intelligence and FEMALEPD.**

**Fig 20. Single SNP analysis of the association between educational attainment and FEMALEPD.**

**Fig 21. Single SNP analysis of the association between cognitive (test) performance and FEMALEPD.**

**Fig 22. Single SNP analysis of the association between intelligence and PDAOO.**

**Fig 23. Scatter plots of single-nucleotide polymorphism (SNP) associated with educational attainment and PDAOO.**

**Fig 24. Single SNP analysis of the association between cognitive (test) performance and PDAOO.**

**Fig 25. Leave-one-out analysis of the association between intelligence and Parkinson’s Disease.**

**Fig 26. Leave-one-out analysis of the association between educational attainment and Parkinson’s Disease.**

**Fig 27. Leave-one-out analysis of the association between cognitive (test) performance and Parkinson’s Disease.**

**Fig 28. Leave-one-out analysis of the association between intelligence and MALEPD.**

**Fig 29. Leave-one-out analysis of the association between educational attainment and MALEPD.**

**Fig 30. Leave-one-out analysis of the association between cognitive (test) performance and MALEPD.**

**Fig 31. Leave-one-out analysis of the association between intelligence and FEMALEPD.**

**Fig 32. Leave-one-out analysis of the association between educational attainment and FEMALEPD.**

**Fig 33. Leave-one-out analysis of the association between cognitive (test) performance and FEMALEPD.**

**Fig 34. Leave-one-out analysis of the association between intelligence and PDAOO.**

**Fig 35. Leave-one-out analysis of the association between educational attainment and PDAOO.**

**Fig 36. Leave-one-out analysis of the association between cognitive (test) performance and PDAOO.**


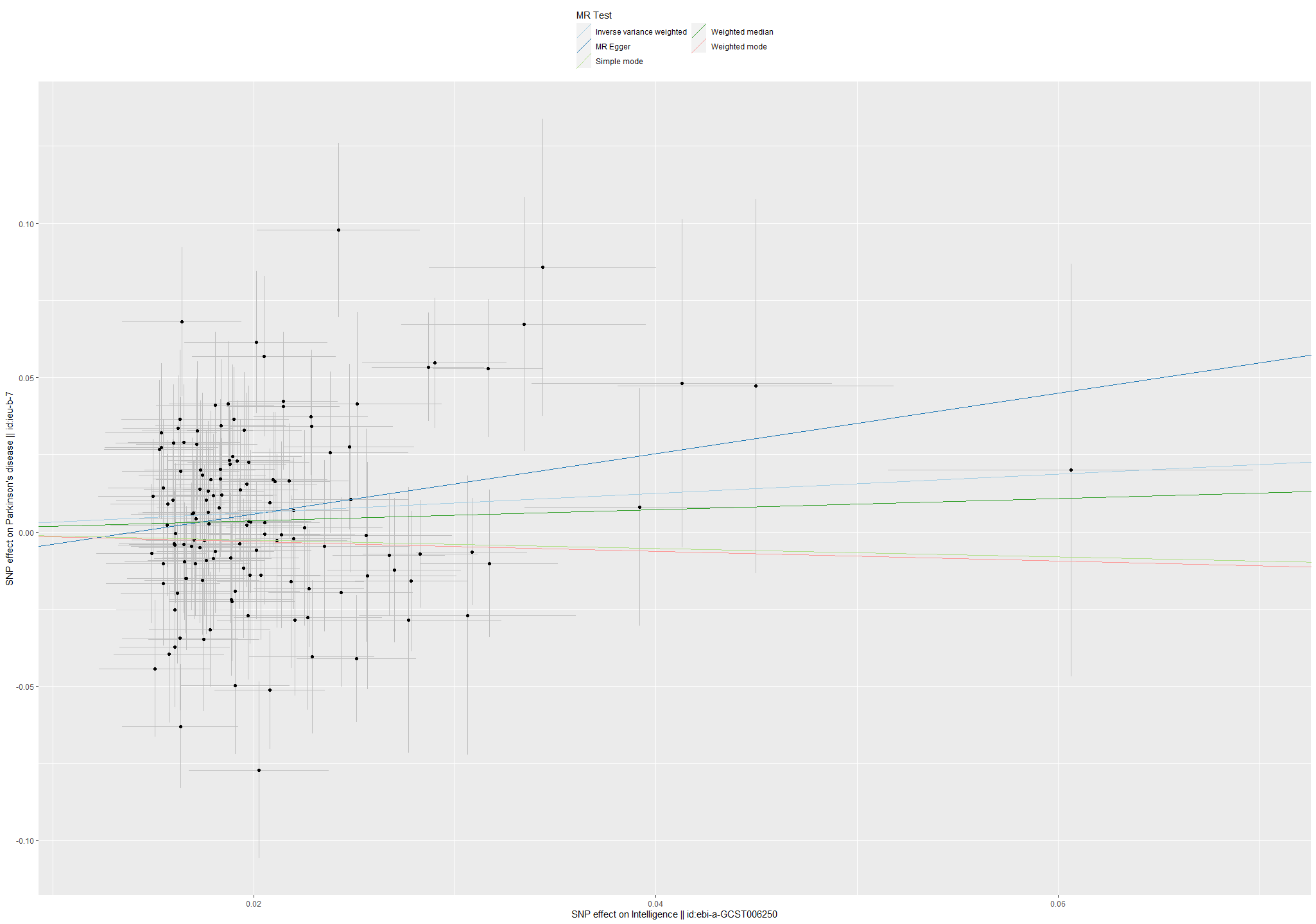


**Fig 1. Scatter plots of single-nucleotide polymorphism (SNP) associated with intelligence and Parkinson’s Disease.**Vertical and horizontal lines around each SNP show a 95% confidence interval. SNP, Single-nucleotide Polymorphism;


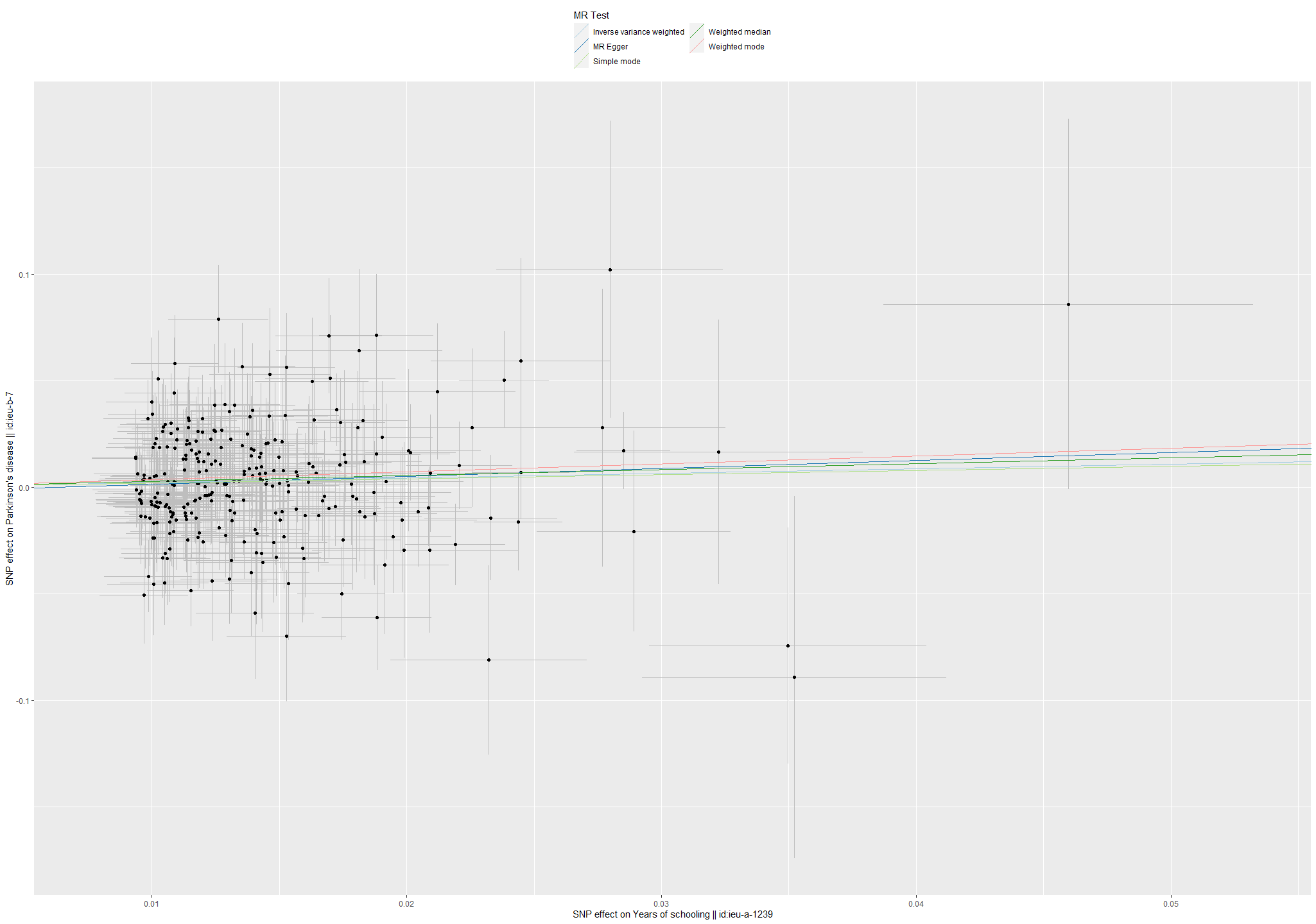


**Fig 2. Scatter plots of single-nucleotide polymorphism (SNP) associated with educational attainment and Parkinson’s Disease.**


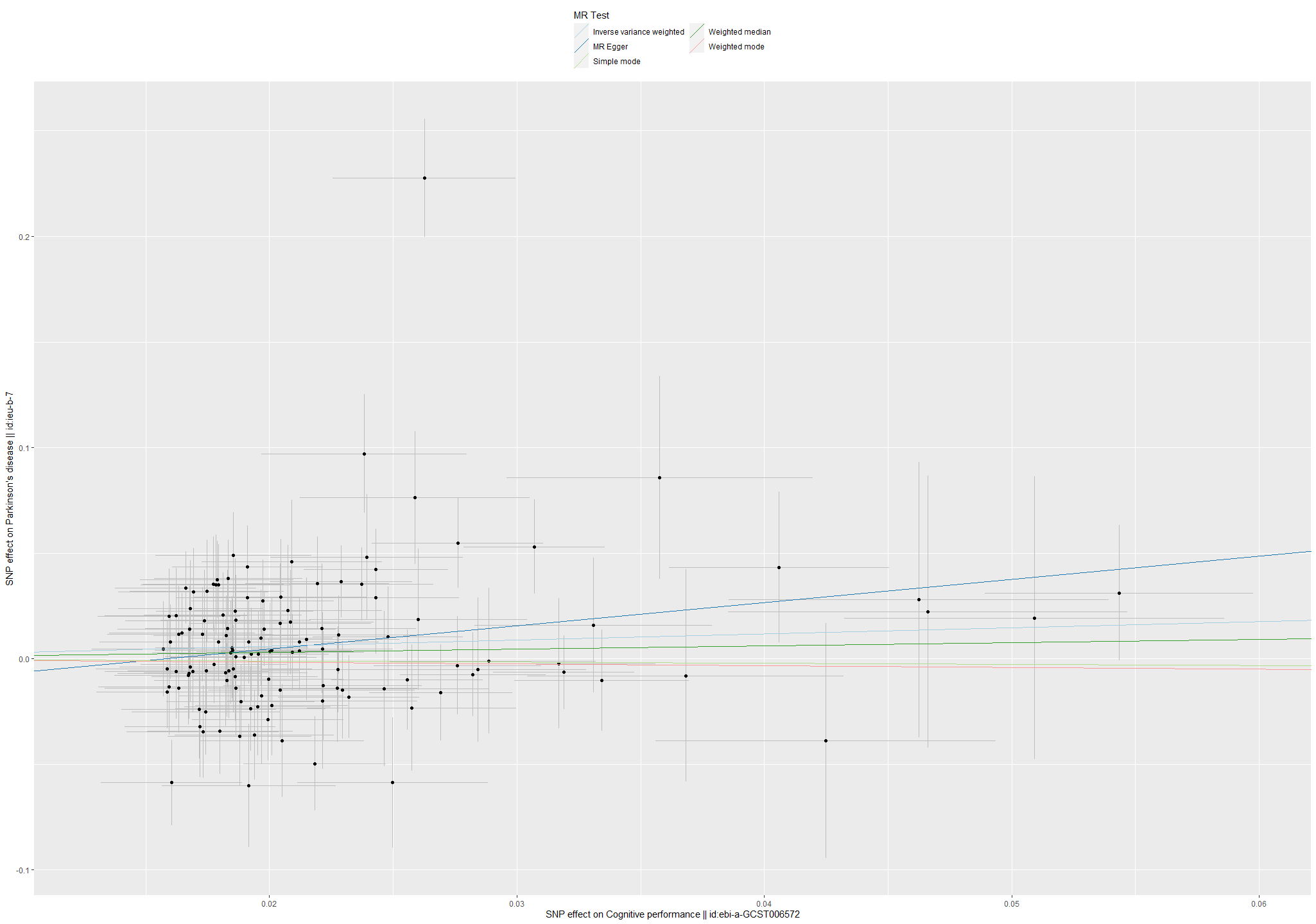


**Fig 3. Scatter plots of single-nucleotide polymorphism (SNP) associated with cognitive (test) performance and Parkinson’s Disease.**


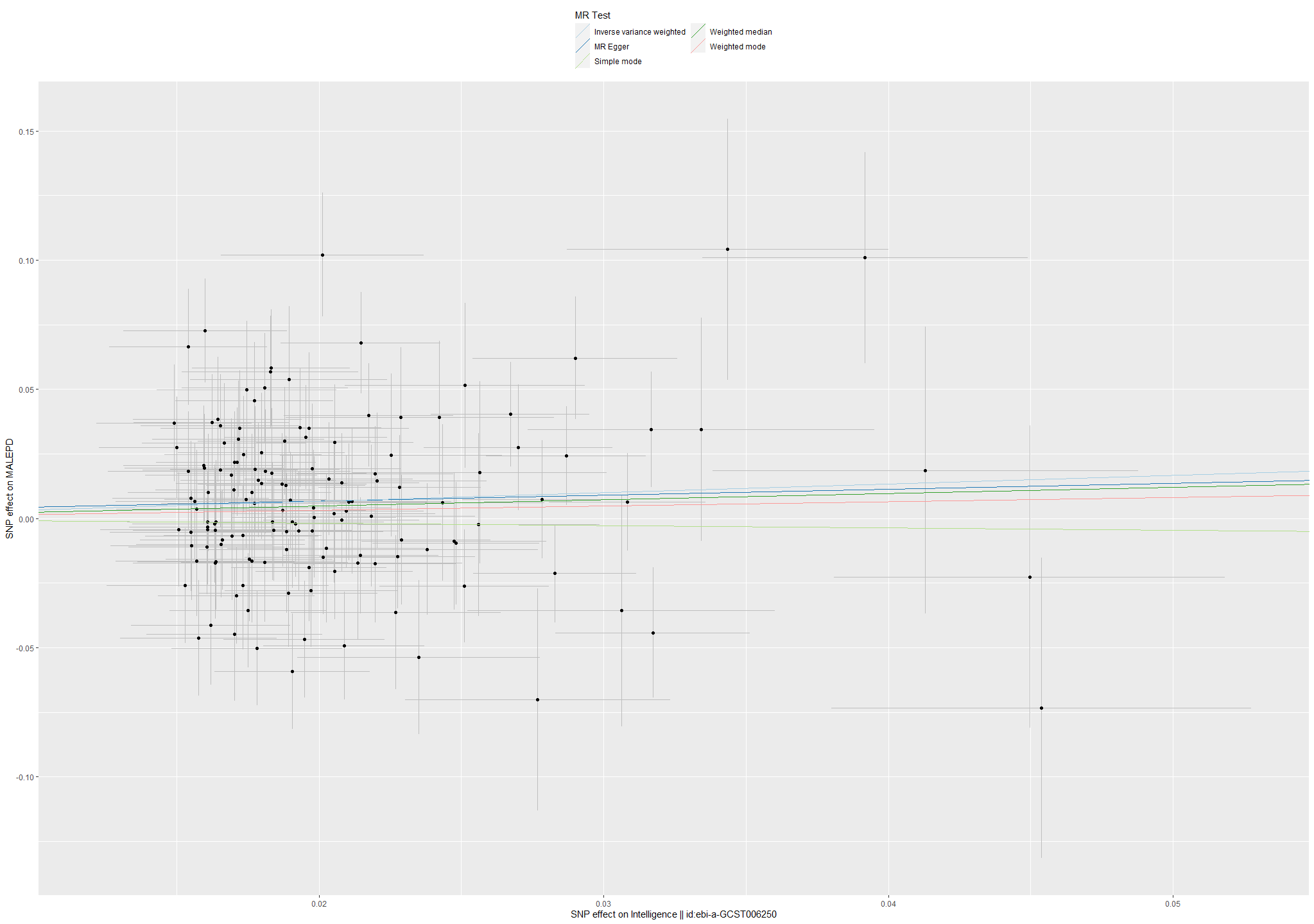


**Fig 4. Scatter plots of single-nucleotide polymorphism (SNP) associated with intelligence and MALEPD.**


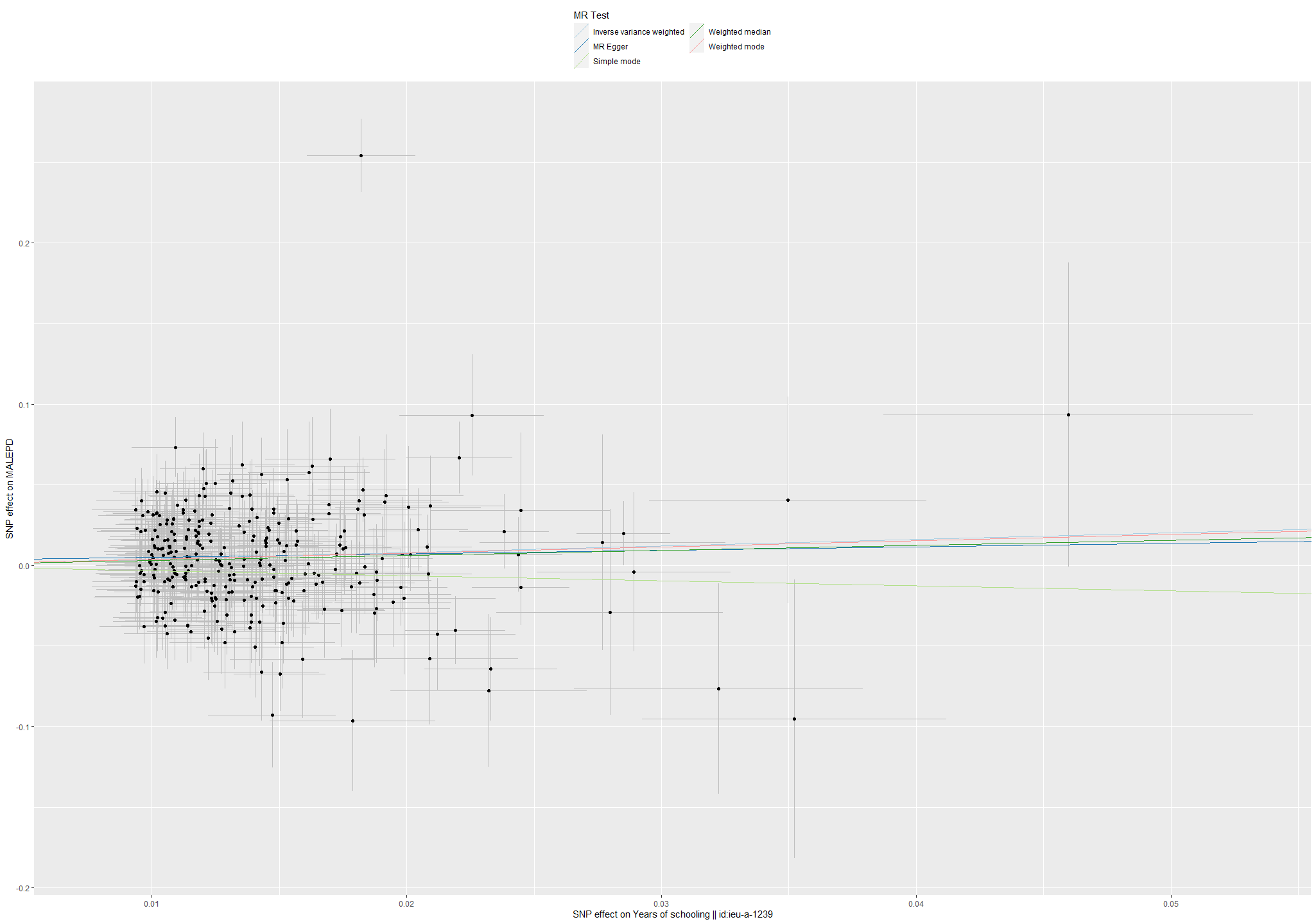


**Fig 5. Scatter plots of single-nucleotide polymorphism (SNP) associated with educational attainment and MALEPD.**


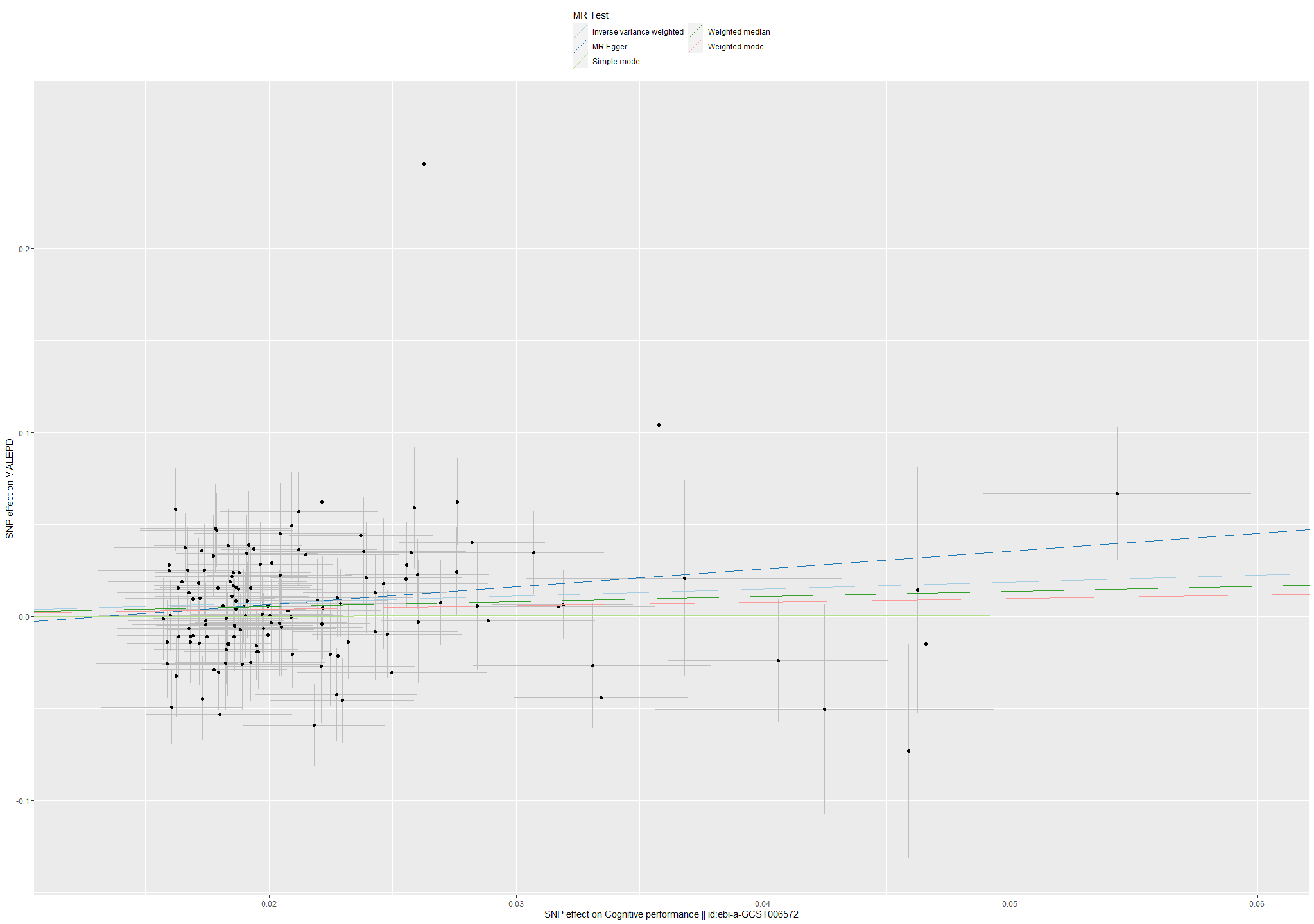


**Fig 6. Scatter plots of single-nucleotide polymorphism (SNP) associated with cognitive (test) performance and MALEPD.**


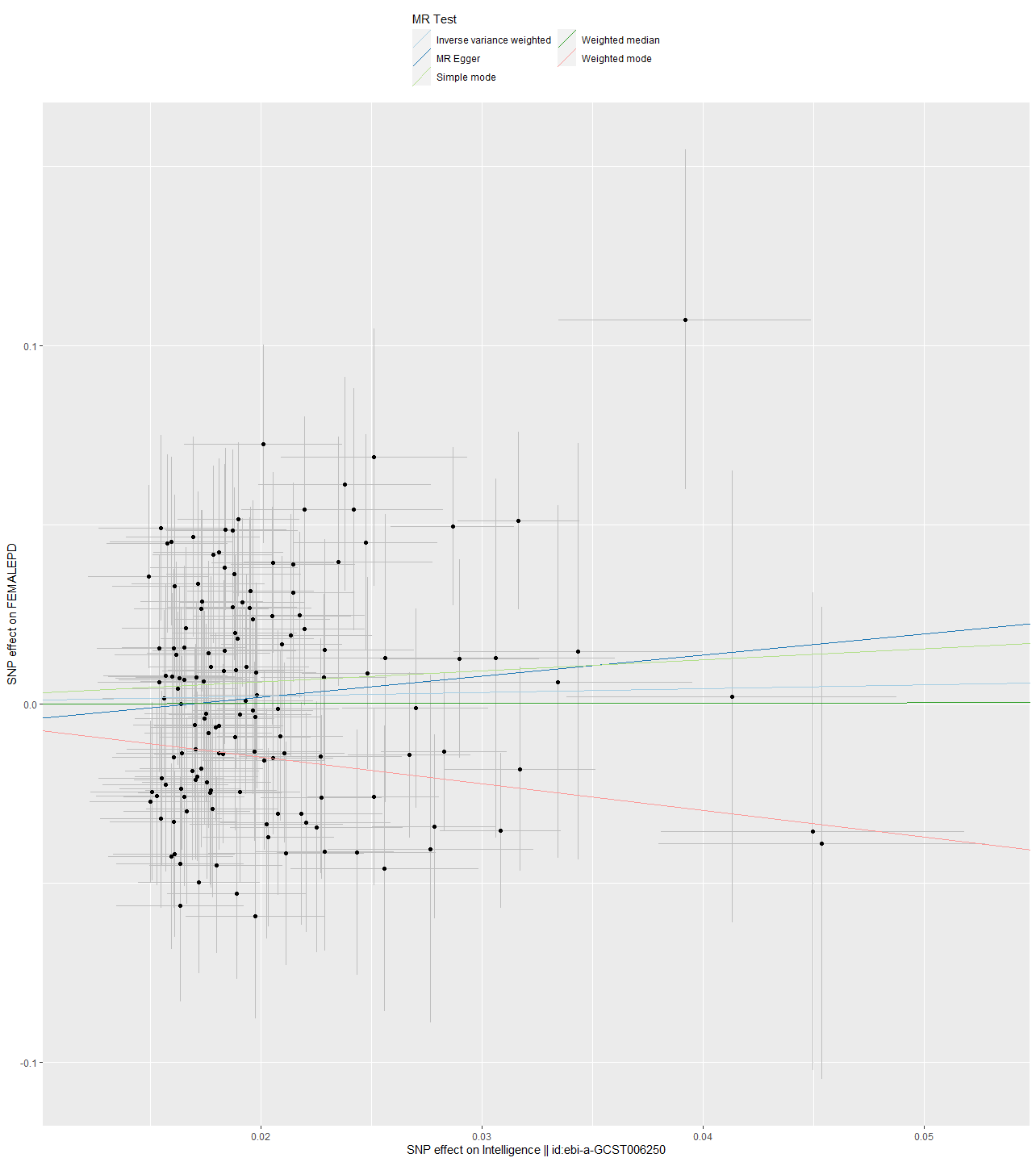


**Fig 7. Scatter plots of single-nucleotide polymorphism (SNP) associated with intelligence and FEMALEPD.**


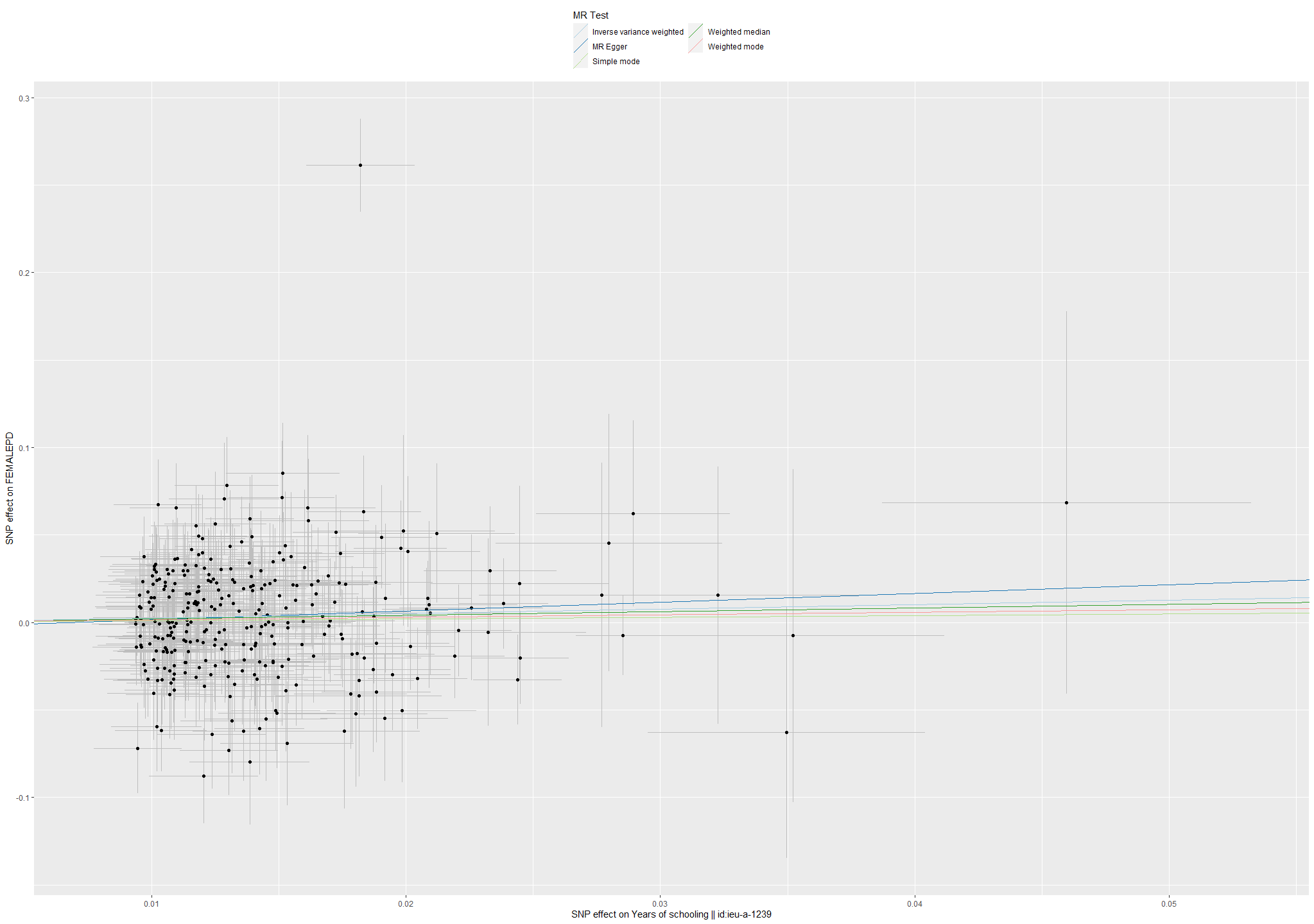


**Fig 8. Scatter plots of single-nucleotide polymorphism (SNP) associated with educational attainment and FEMALEPD.**


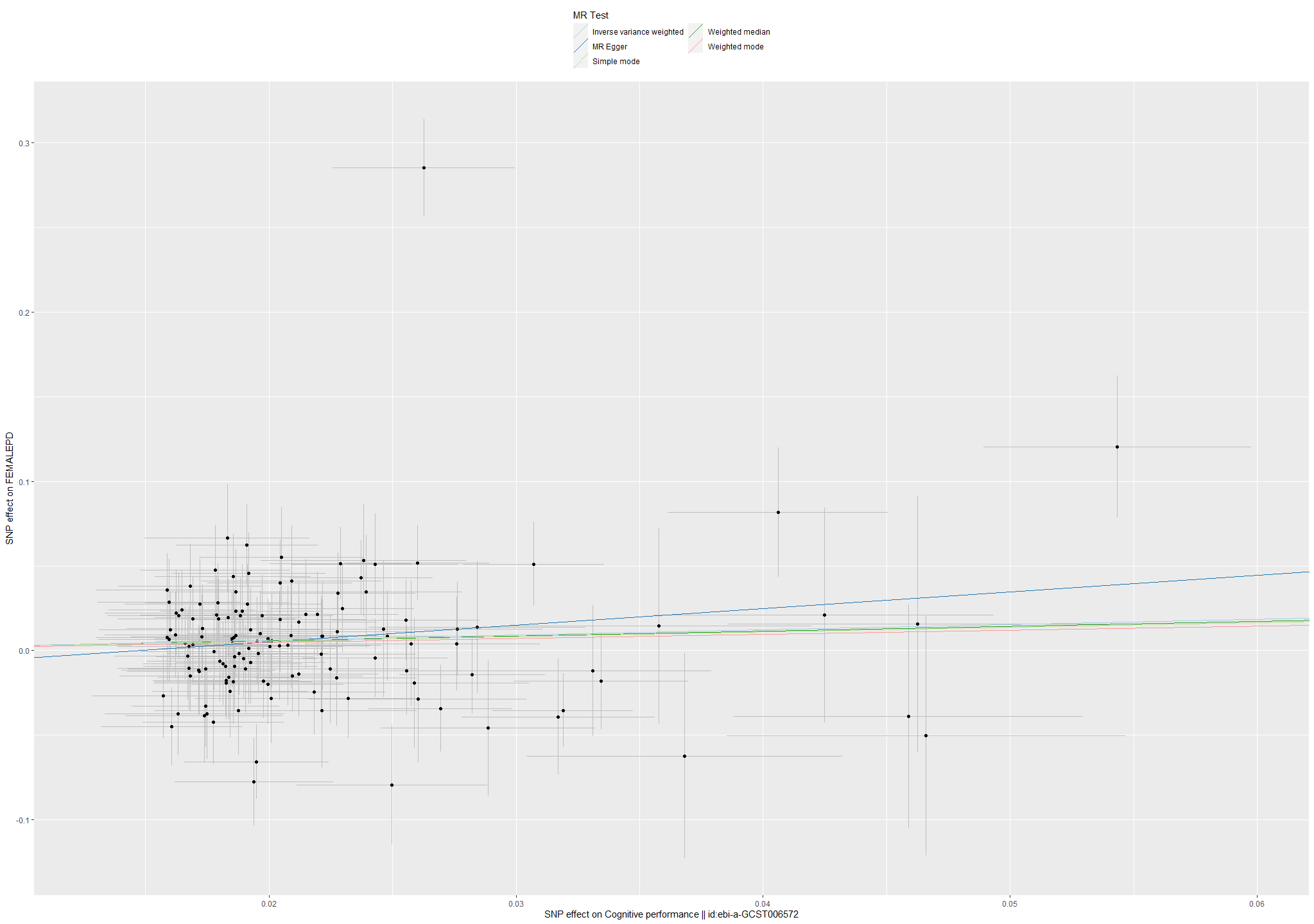


**Fig 9. Scatter plots of single-nucleotide polymorphism (SNP) associated with cognitive (test) performance and FEMALEPD.**


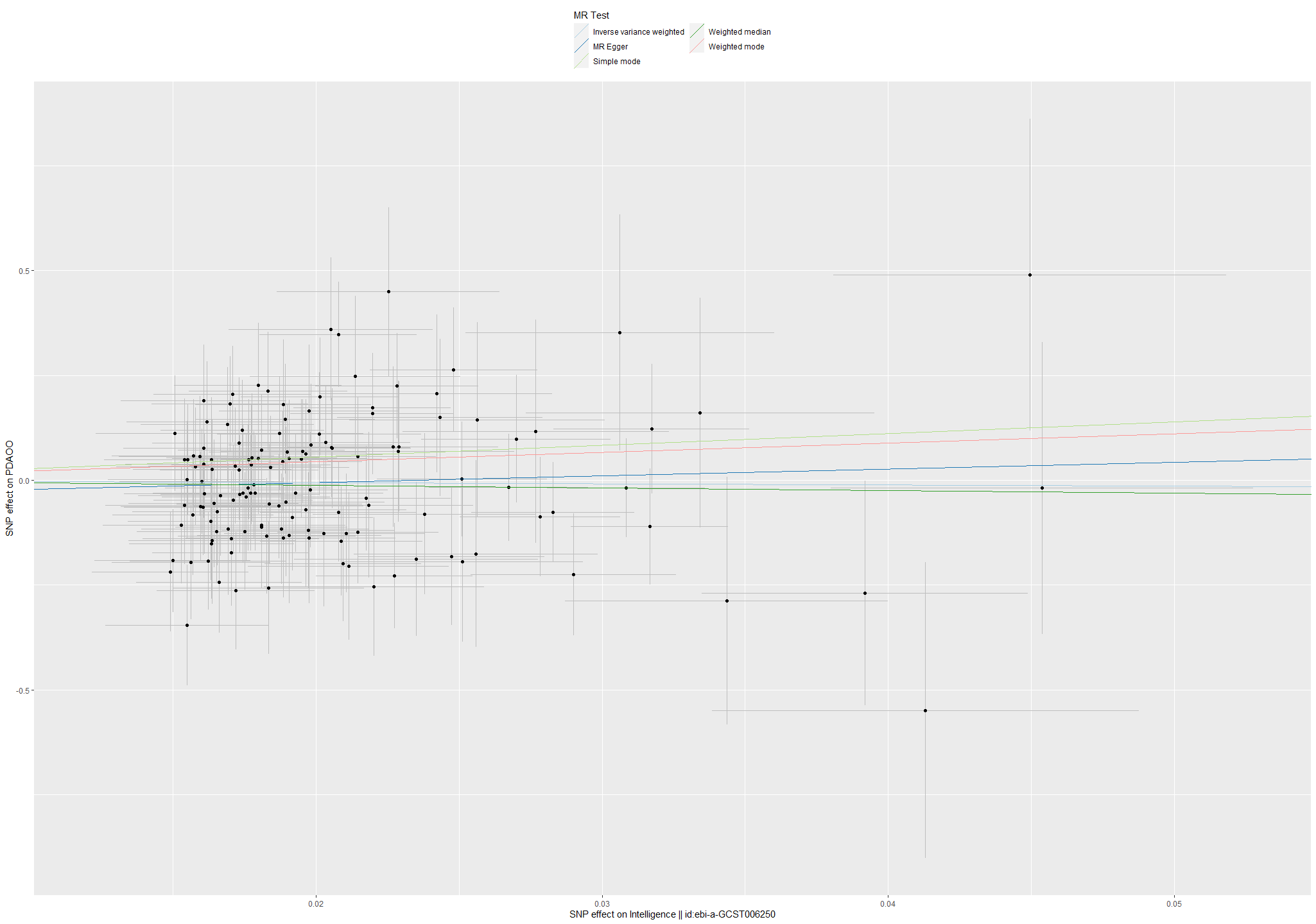


**Fig 10. Scatter plots of single-nucleotide polymorphism (SNP) associated with intelligence and PDAOO.**


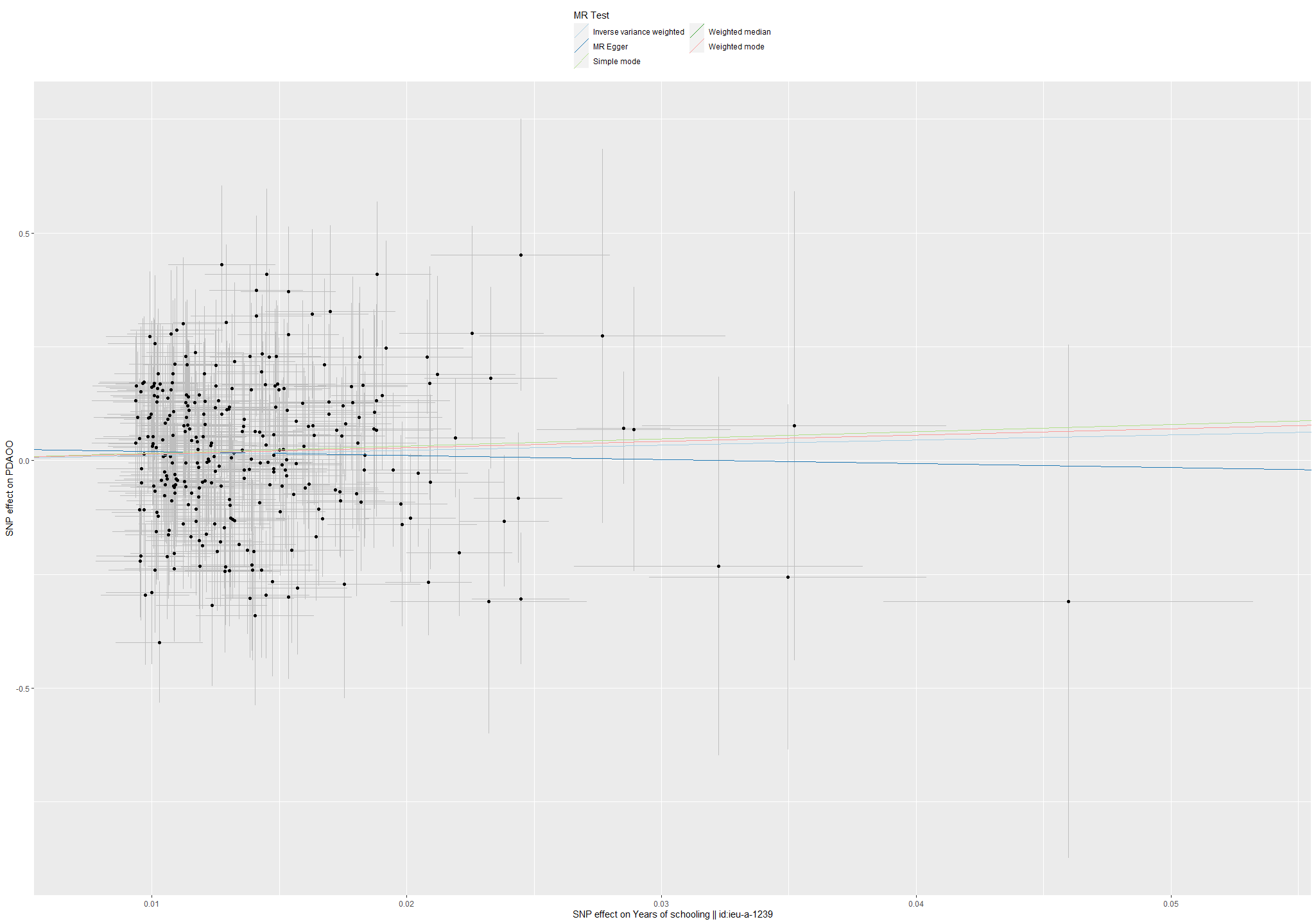


**Fig 11. Scatter plots of single-nucleotide polymorphism (SNP) associated with educational attainment and PDAOO.**


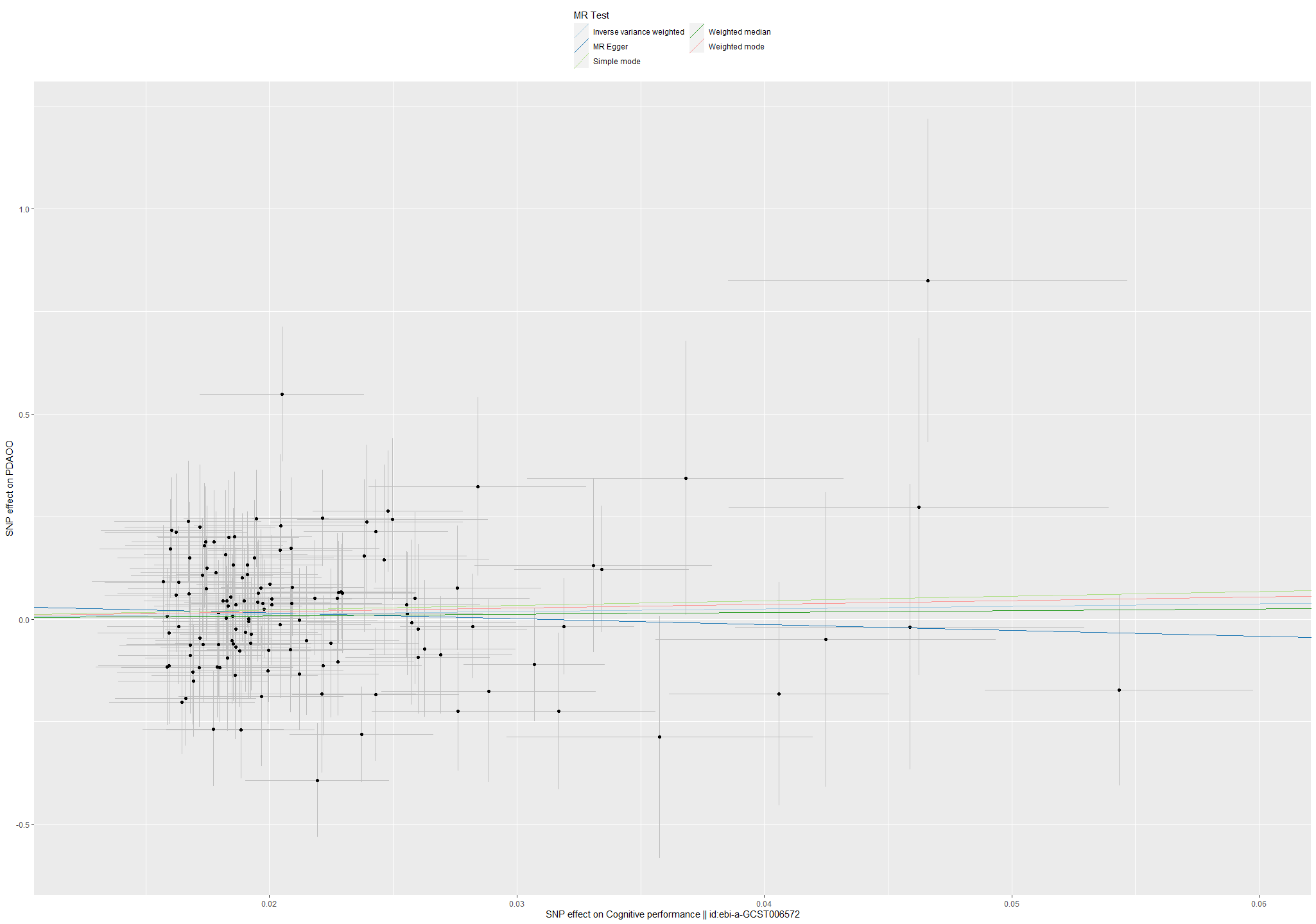


**Fig 12. Scatter plots of single-nucleotide polymorphism (SNP) associated with cognitive (test) performance and PDAOO.**


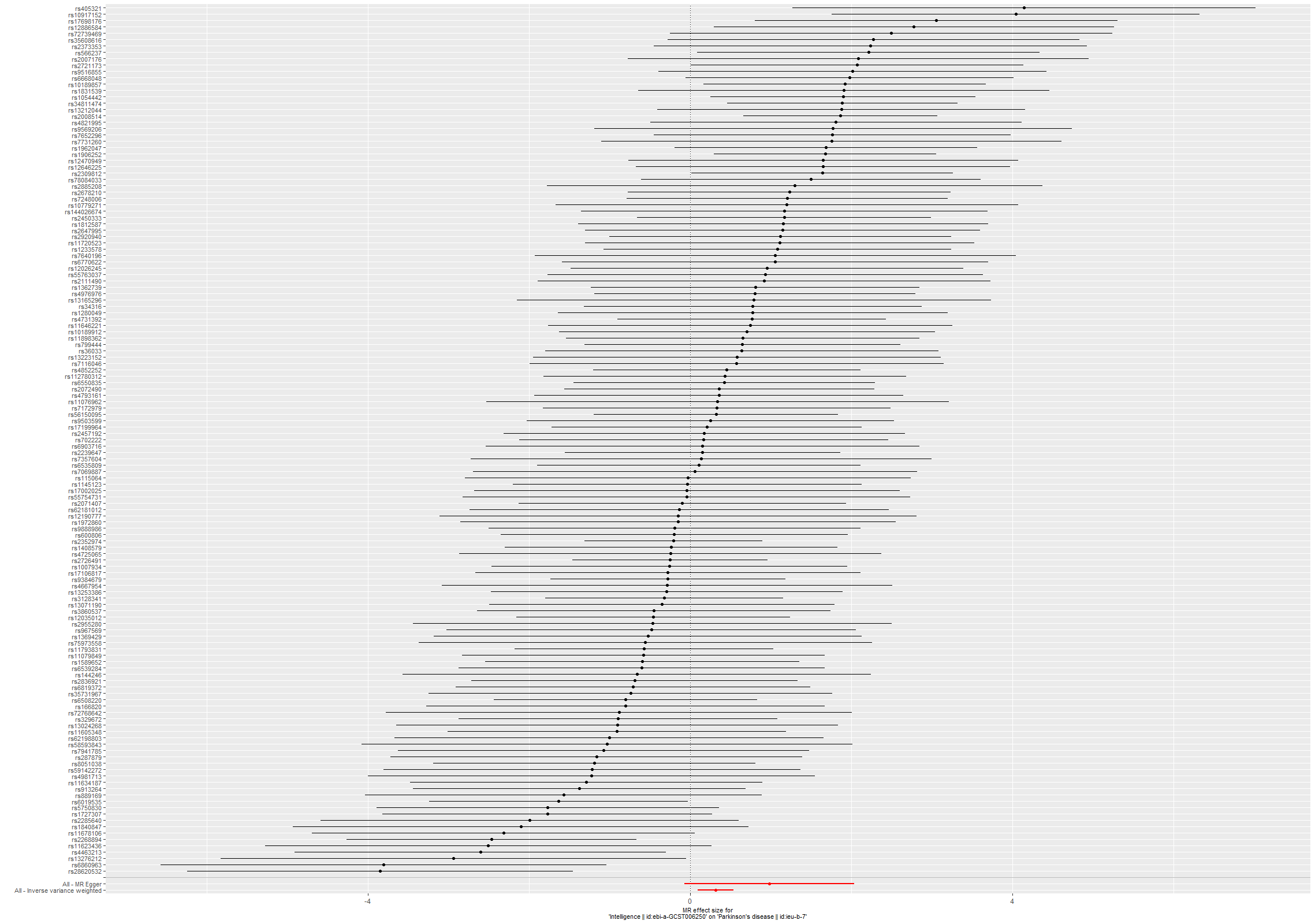


**Fig 13. Single SNP analysis of the association between intelligence and Parkinson’s Disease.** Each horizontal solid line in the forest plots above reflects the result estimated for a single SNP using the Wald ratio method, and those that cross zero indicate that the result is not significant. The bottom red line reflects Association between exposure and PD under the IVW method.


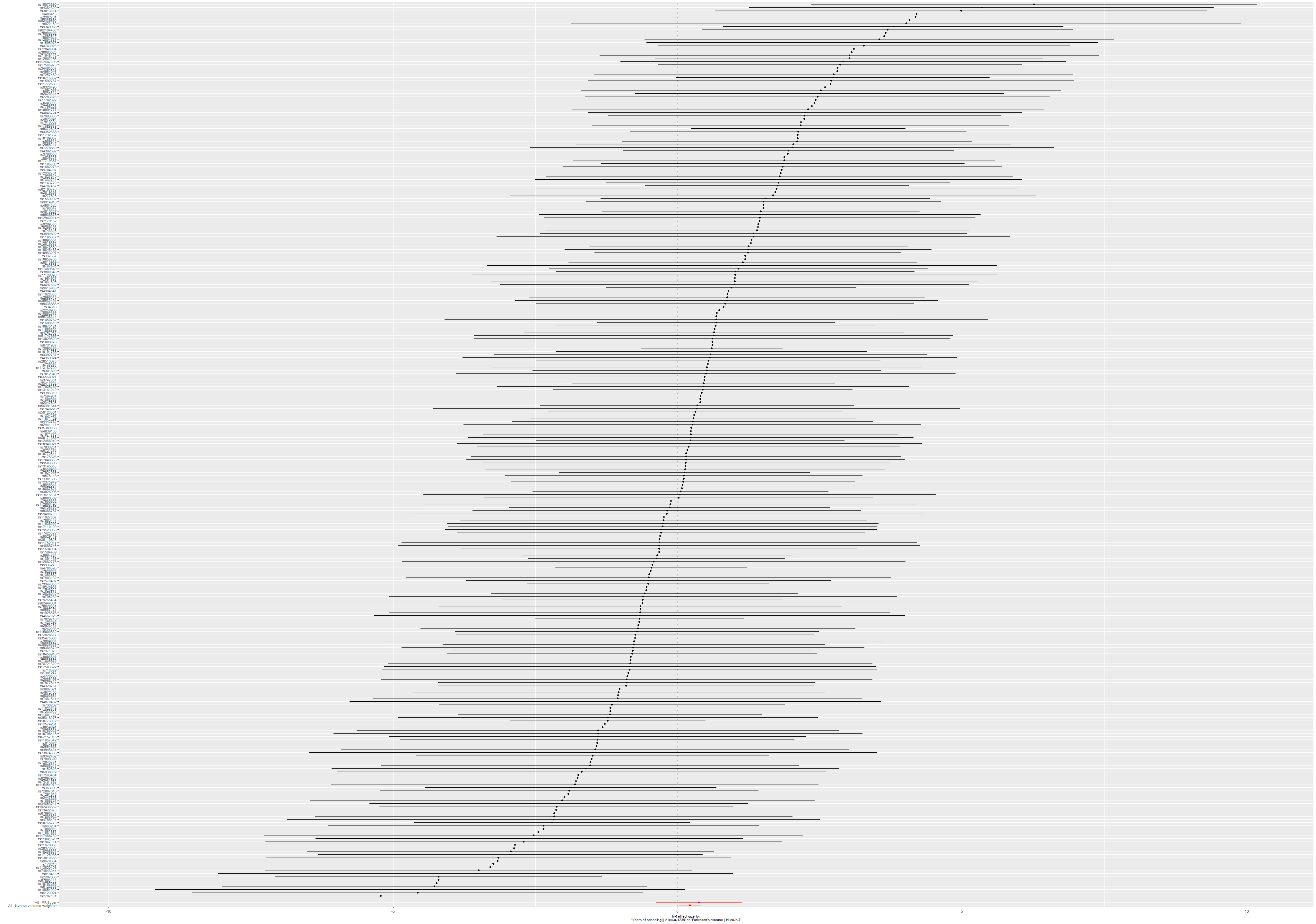


**Fig 14. Single SNP analysis of the association between educational attainment and Parkinson’s Disease.**


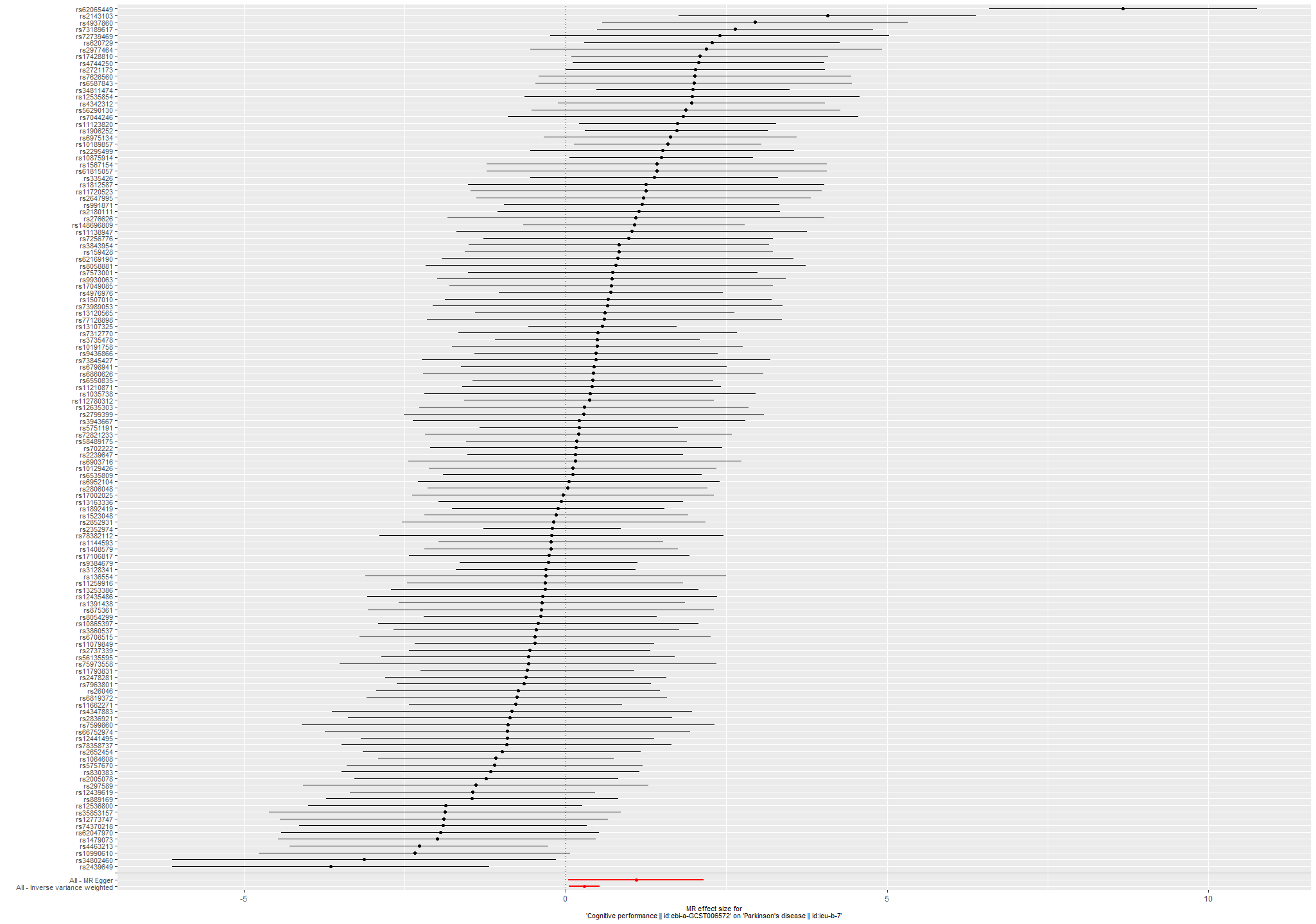


**Fig 15. Single SNP analysis of the association between cognitive (test) performance and Parkinson’s Disease.**


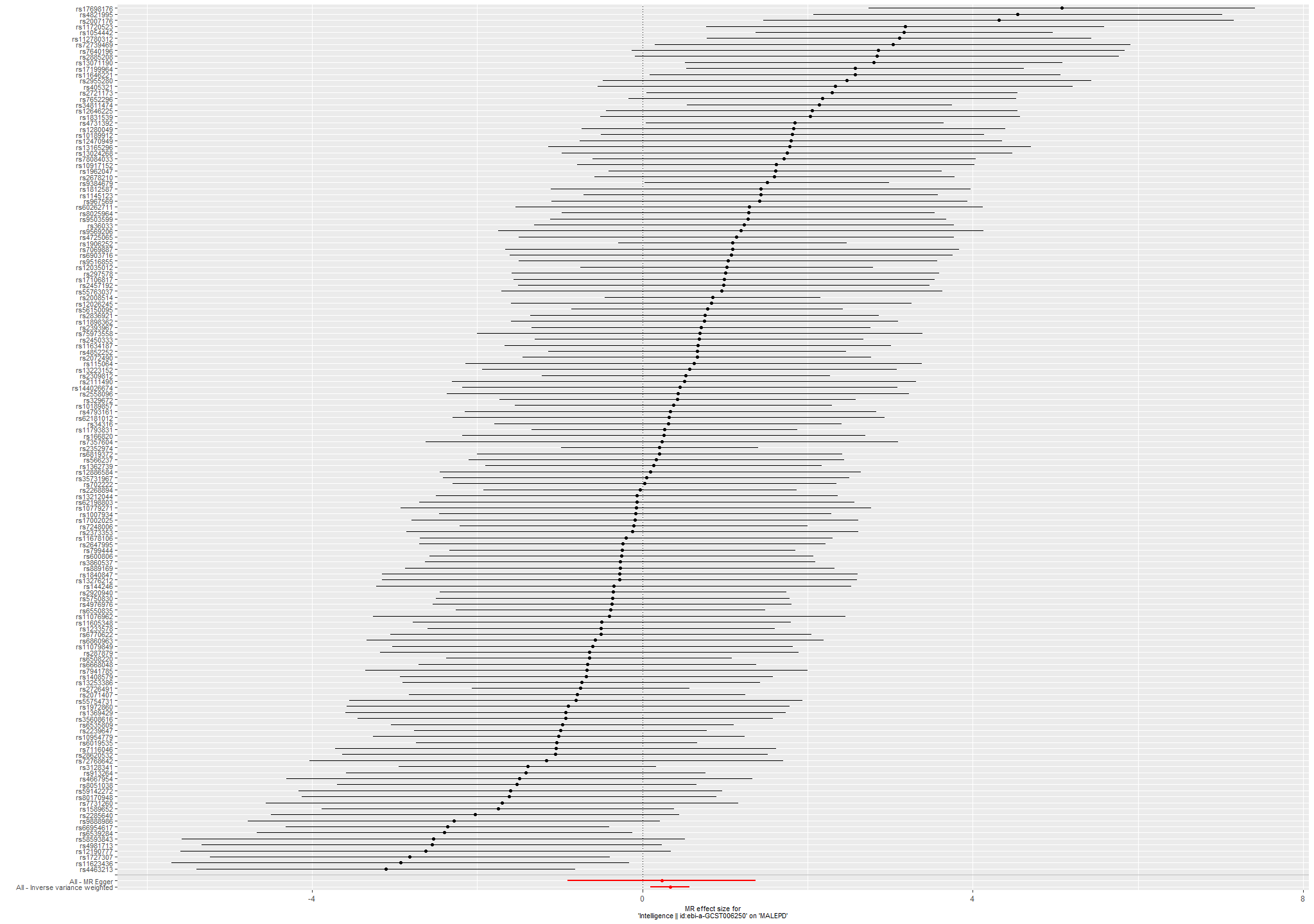


**Fig 16. Single SNP analysis of the association between intelligence and MALEPD.**


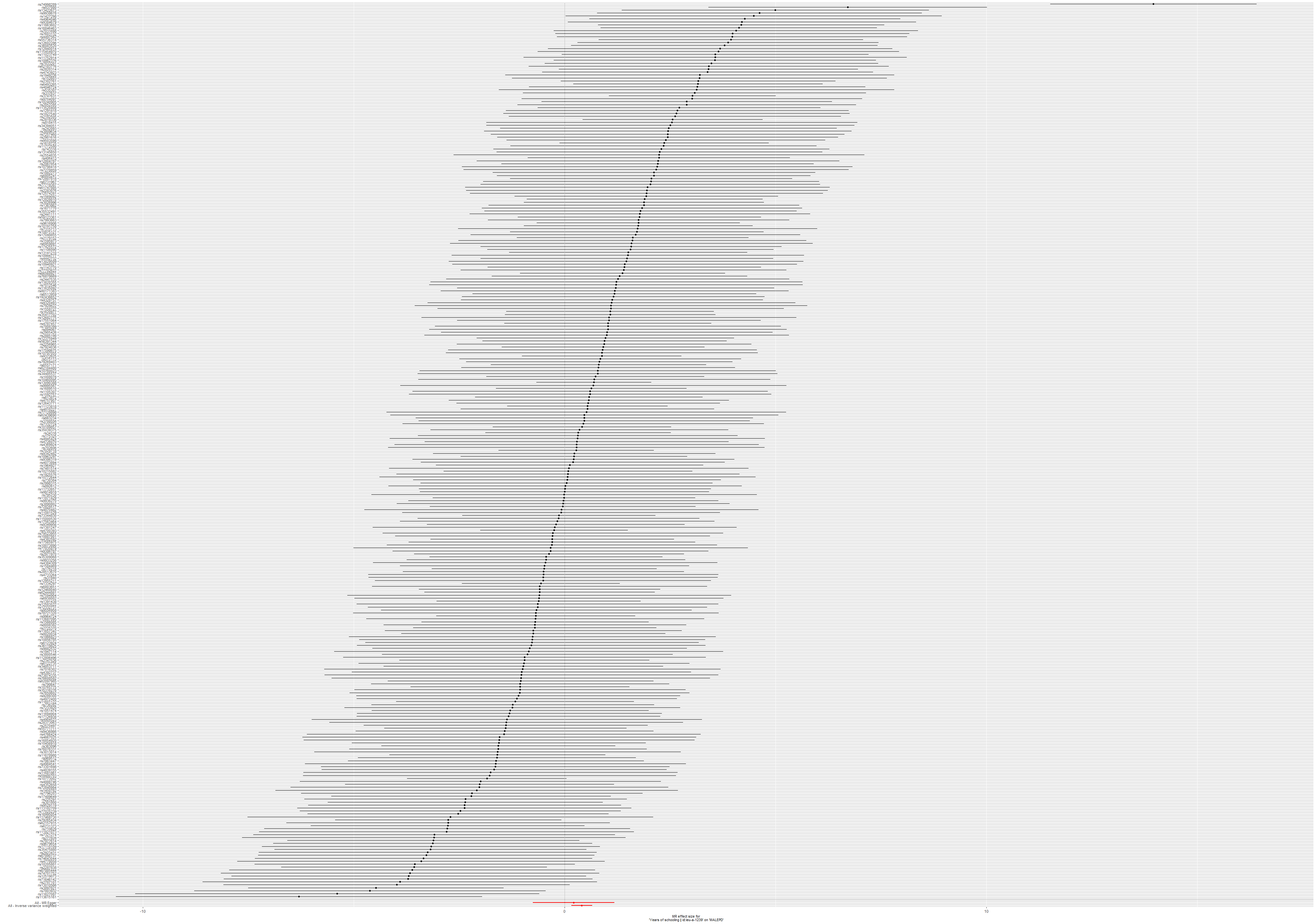


**Fig 17. Scatter plots of single-nucleotide polymorphism (SNP) associated with educational attainment and MALEPD.**


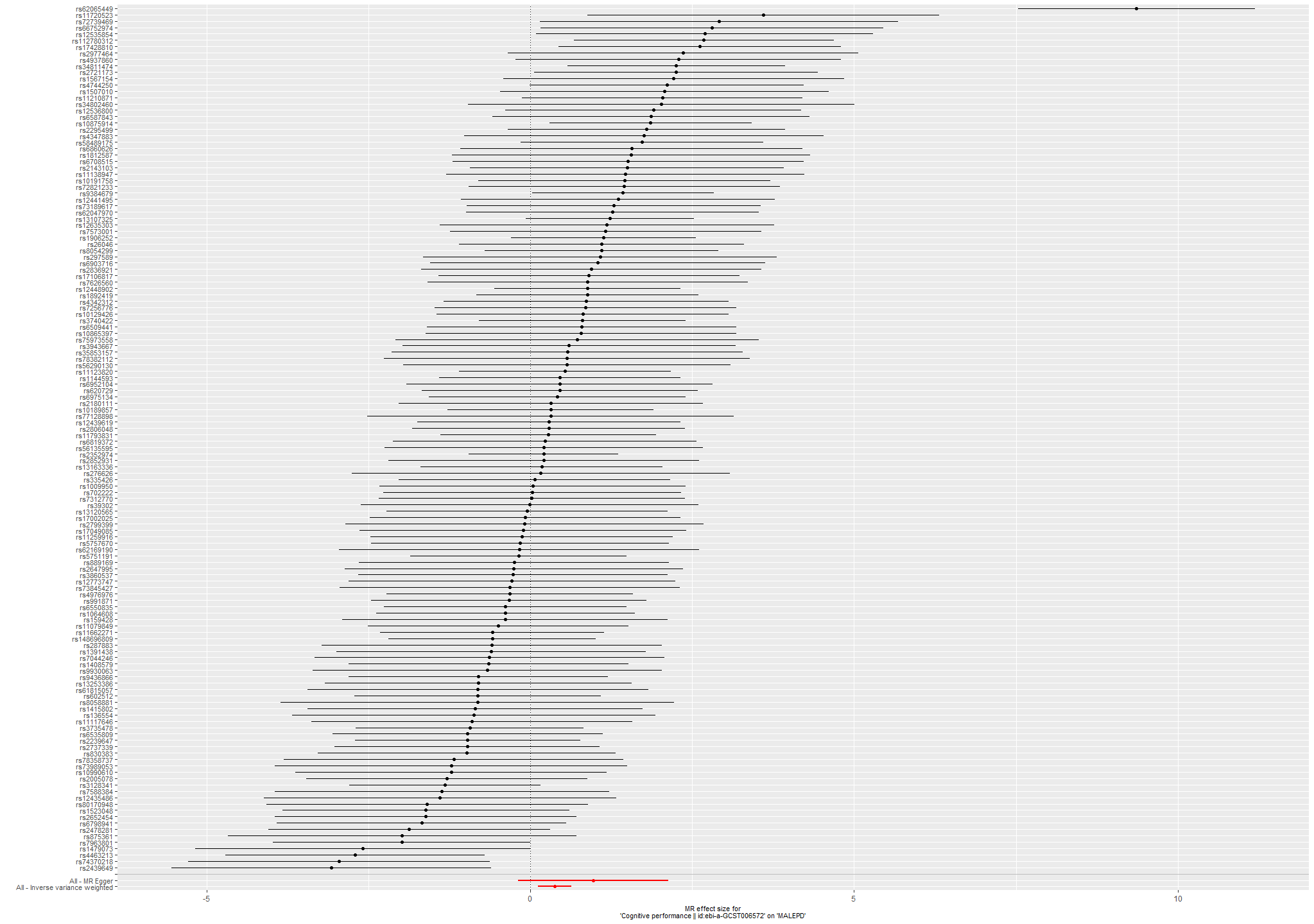


**Fig 18. Single SNP analysis of the association between cognitive (test) performance and MALEPD.**


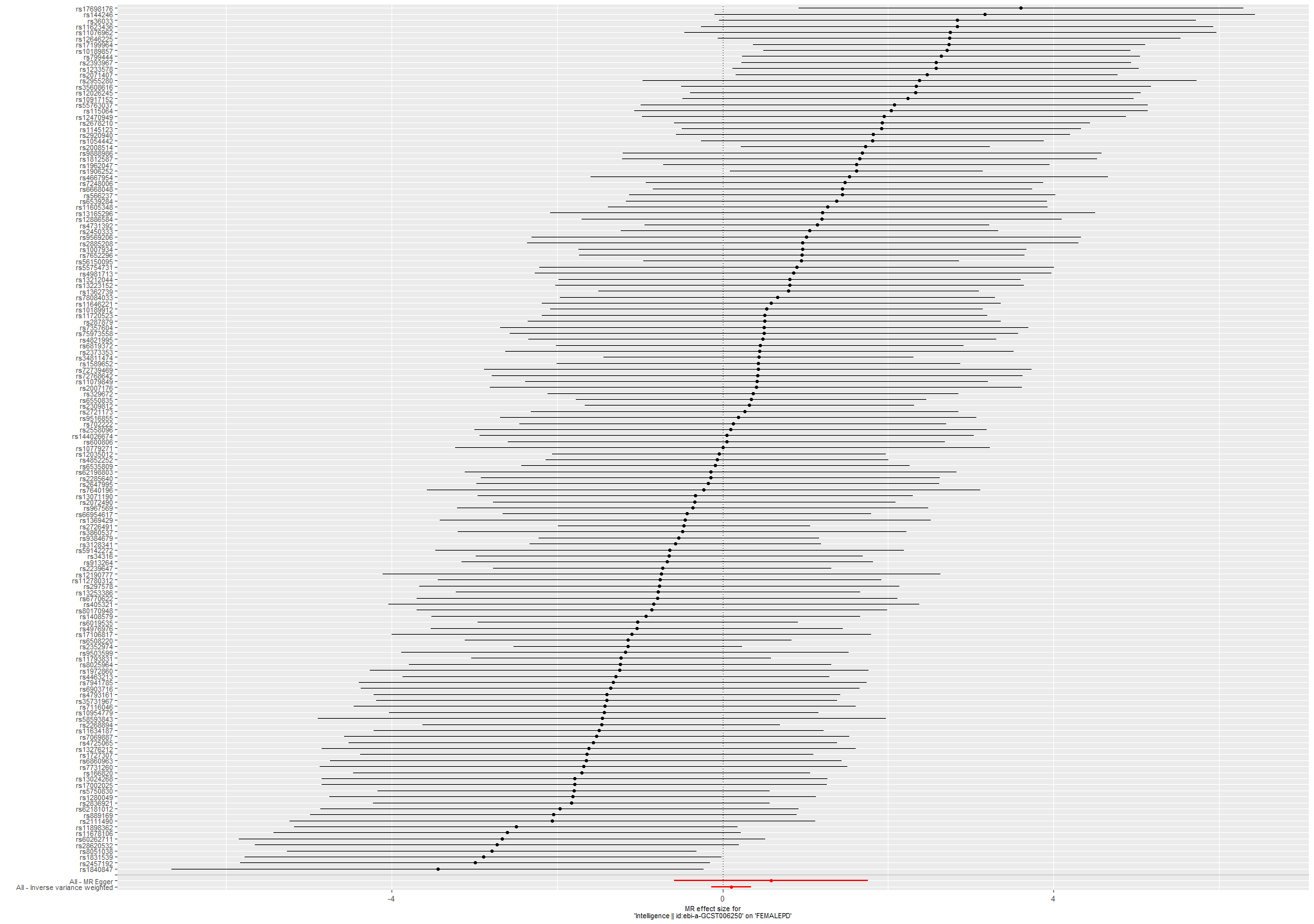


**Fig 19. Single SNP analysis of the association between intelligence and FEMALEPD.**


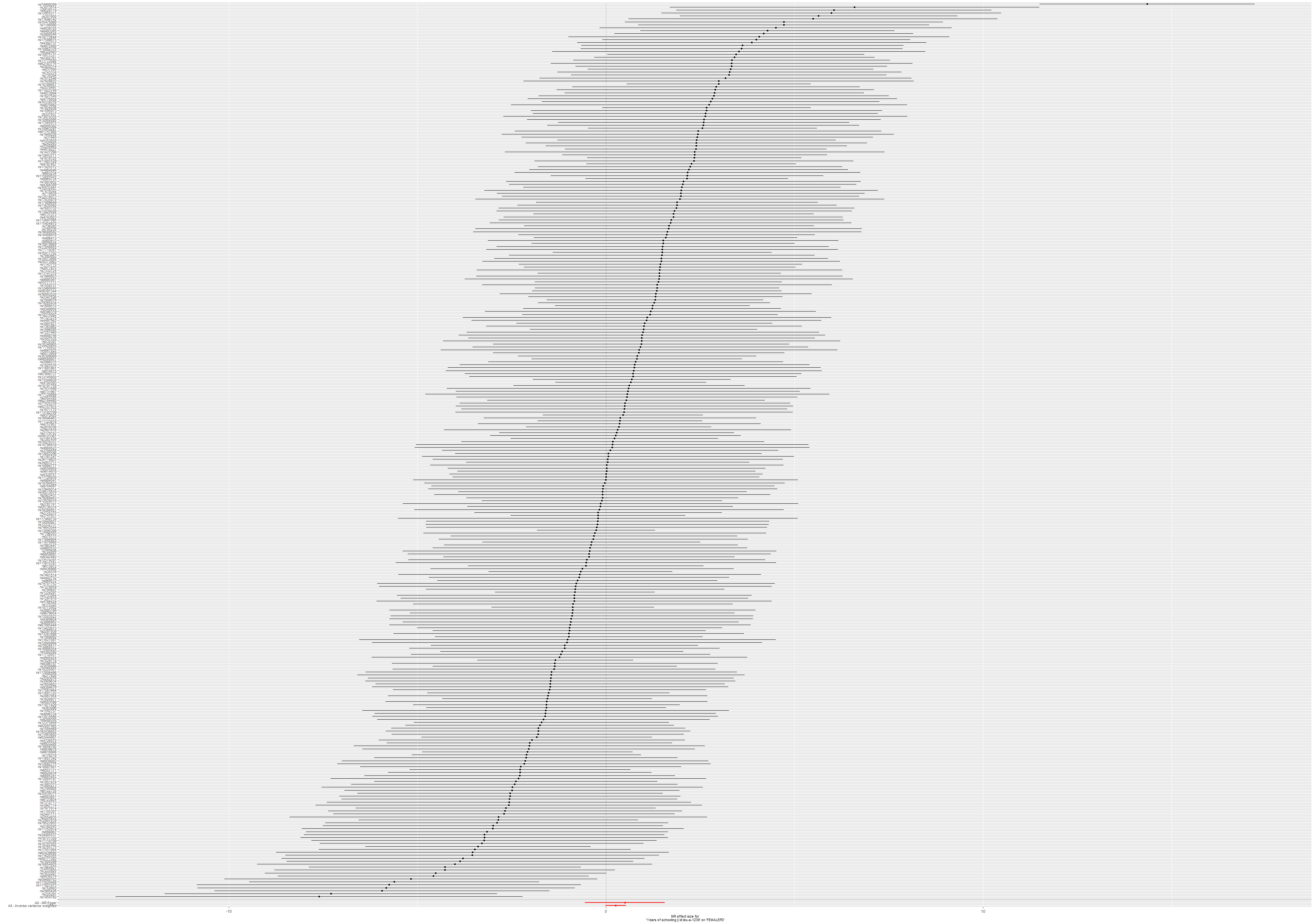


**Fig 20. Single SNP analysis of the association between educational attainment and FEMALEPD.**


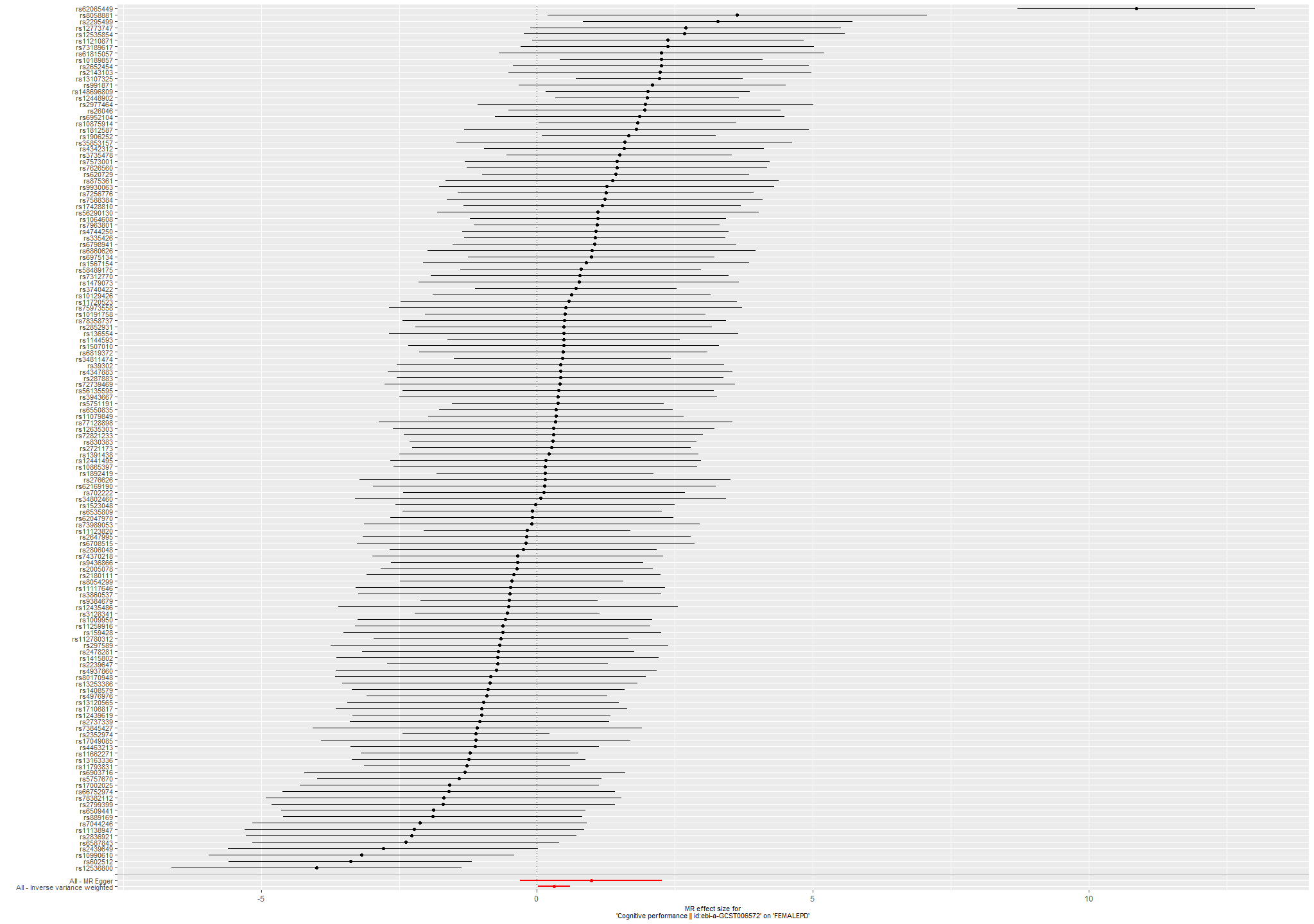


**Fig 21. Single SNP analysis of the association between cognitive (test) performance and FEMALEPD.**


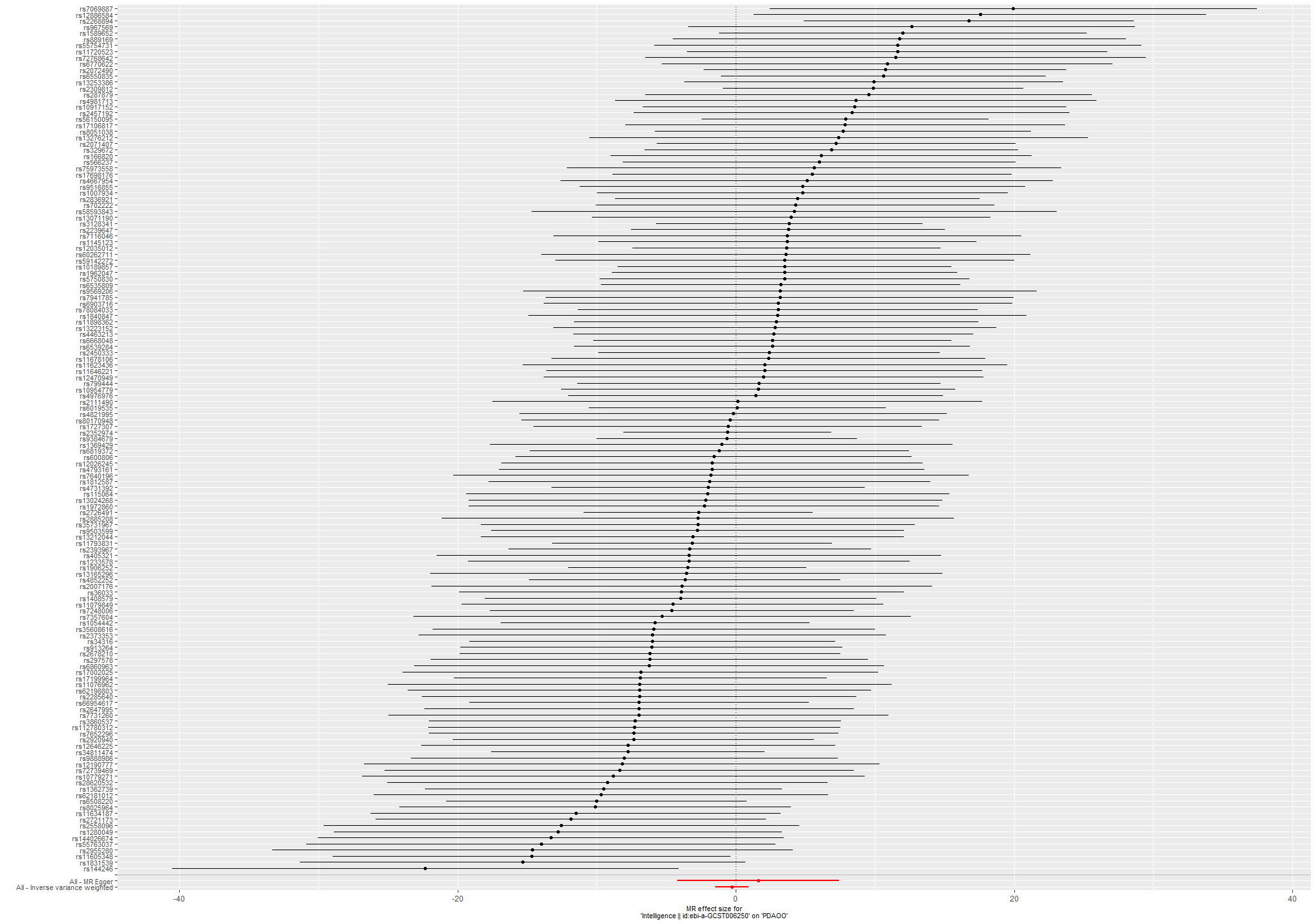


**Fig 22. Single SNP analysis of the association between intelligence and PDAOO.**


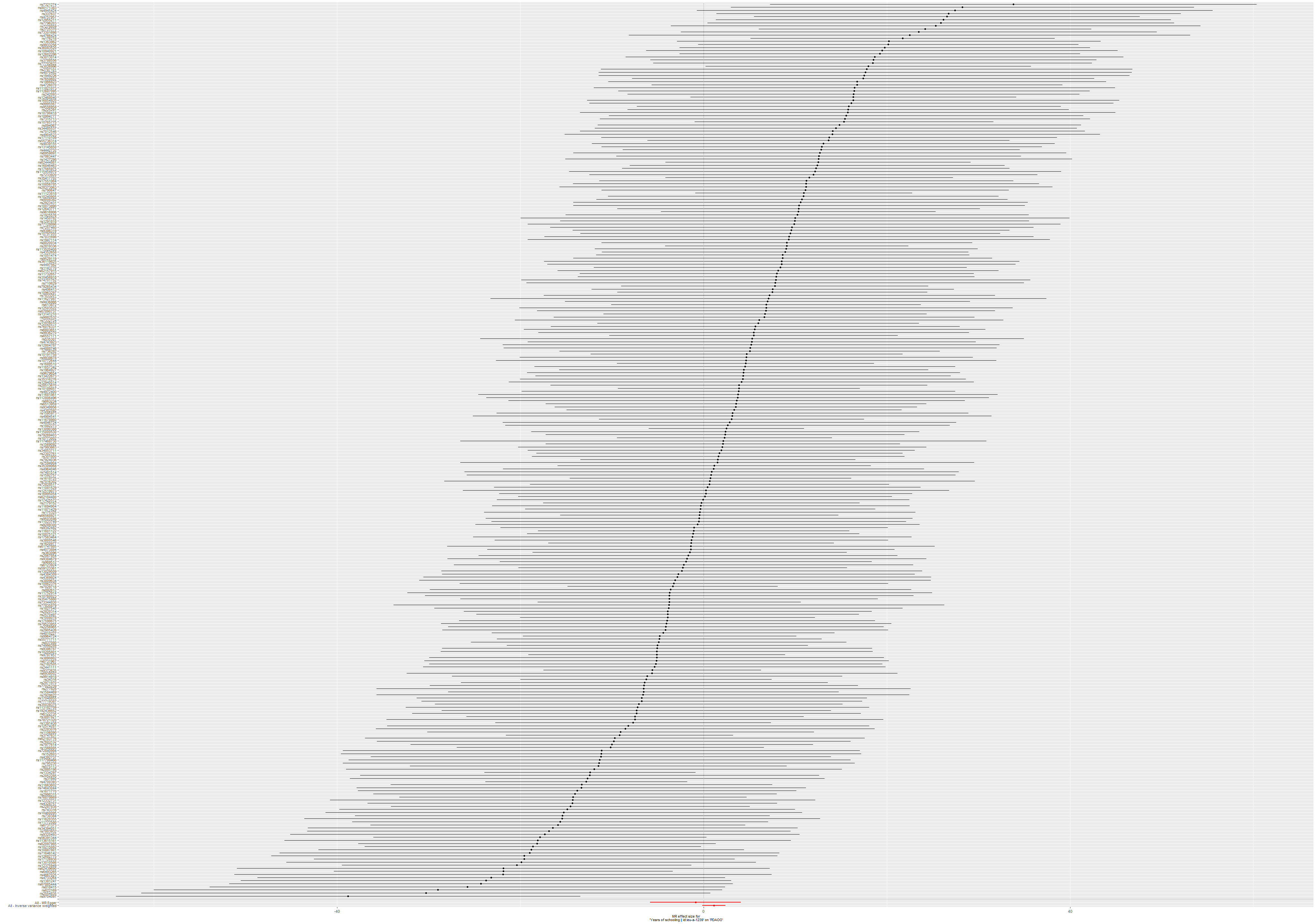


**Fig 23. Scatter plots of single-nucleotide polymorphism (SNP) associated with educational attainment and PDAOO.**


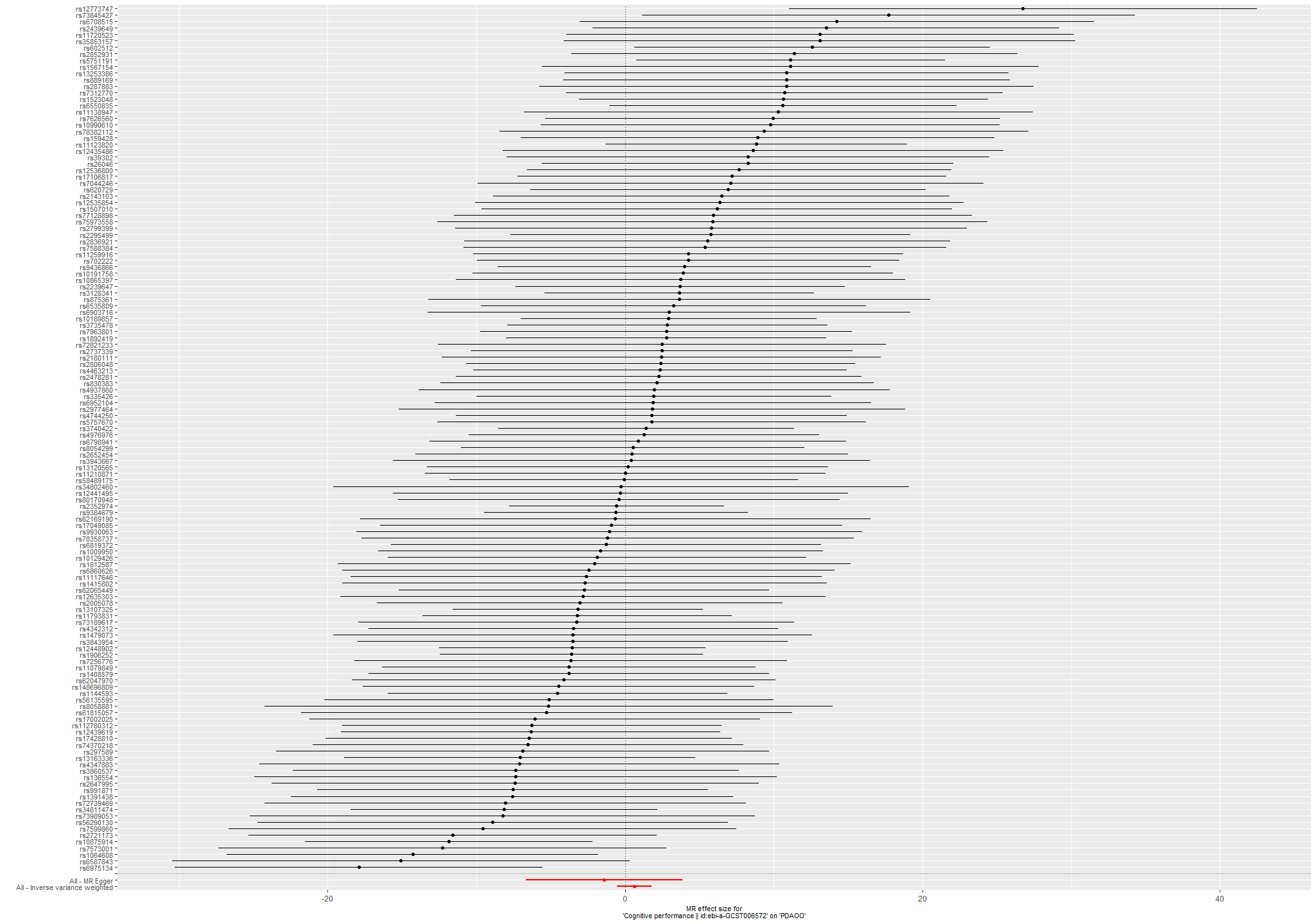


**Fig 24. Single SNP analysis of the association between cognitive (test) performance and PDAOO.**


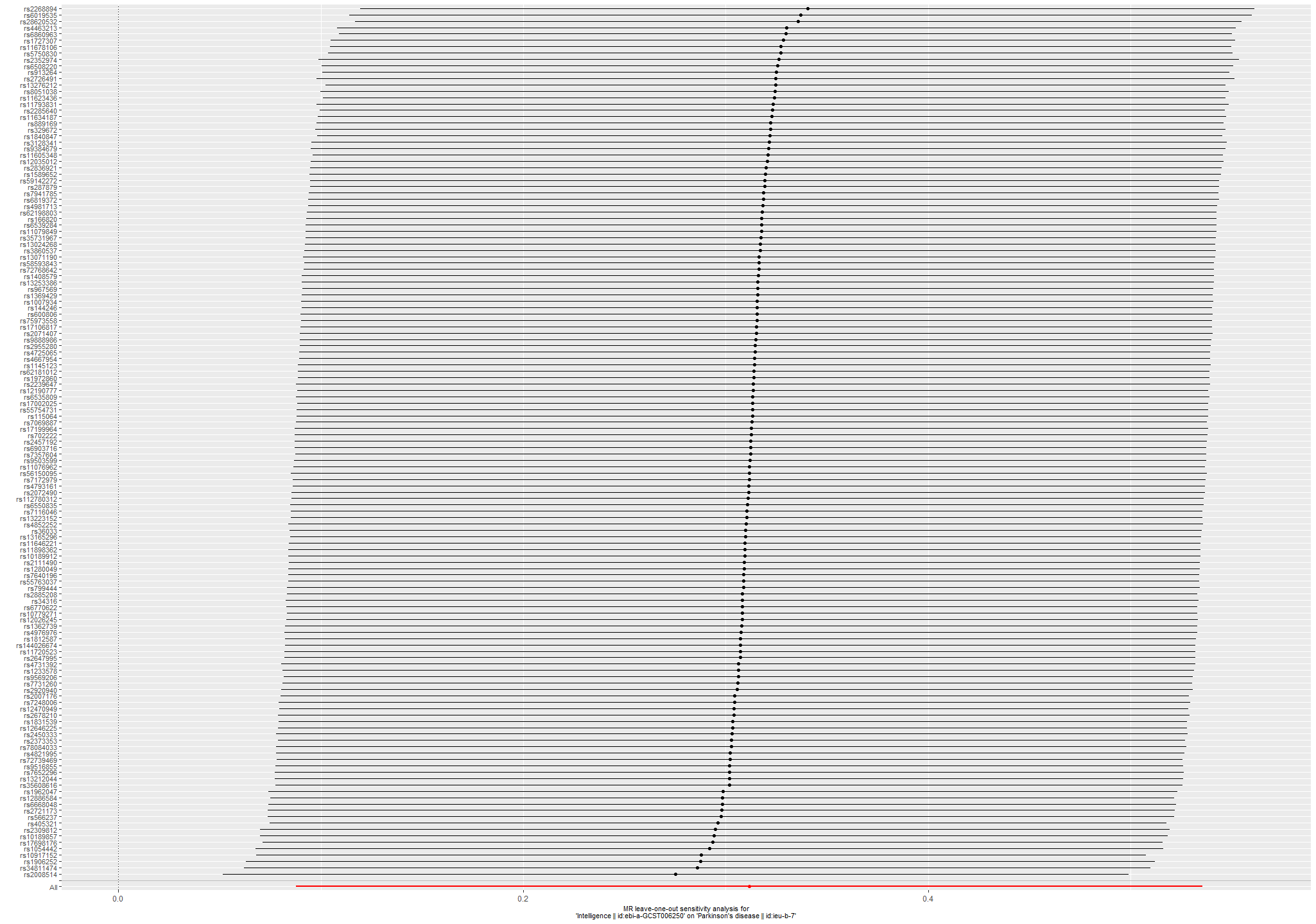


**Fig 25. Leave-one-out analysis of the association between intelligence and Parkinson’s Disease.**


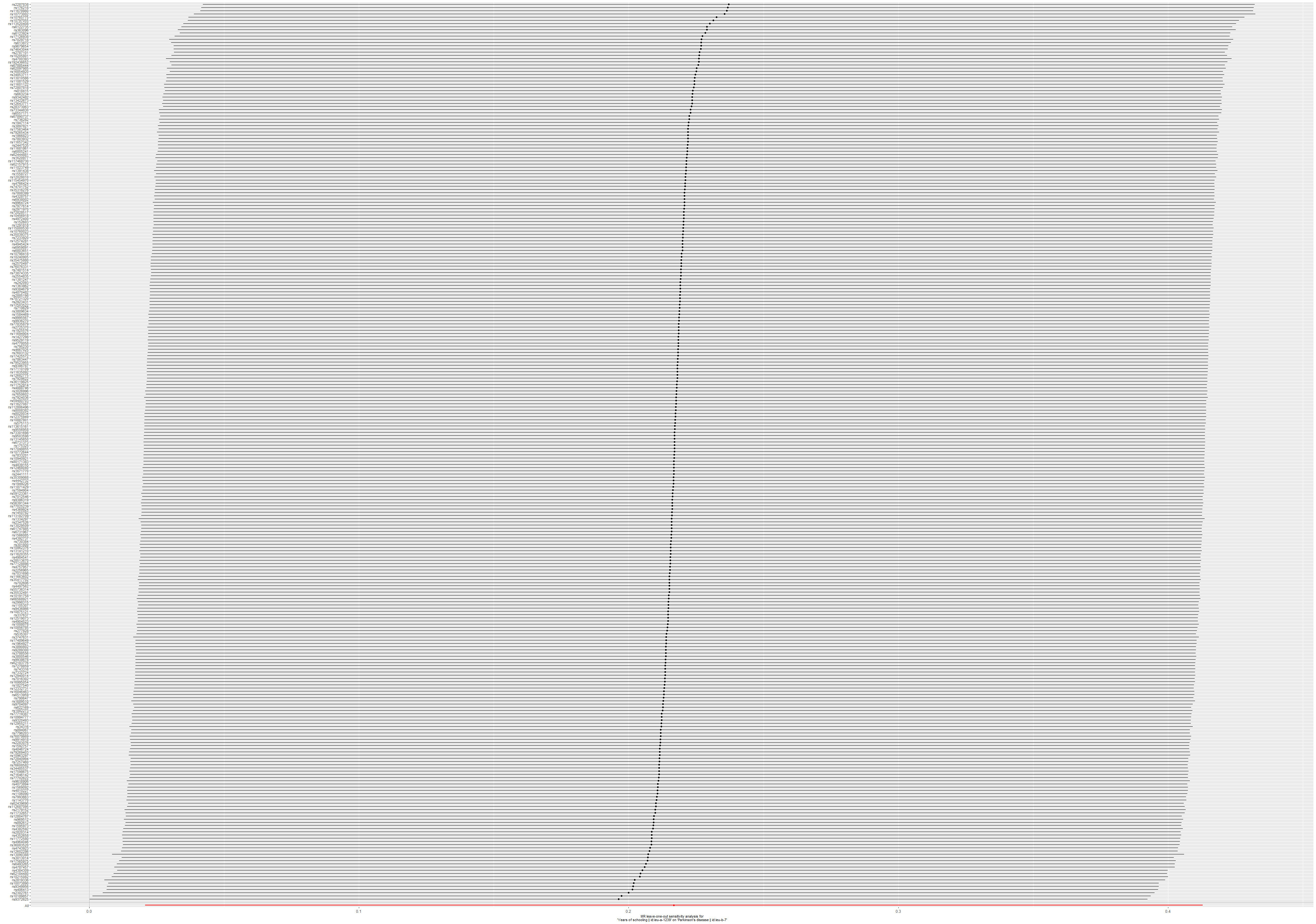


**Fig 26. Leave-one-out analysis of the association between educational attainment and Parkinson’s Disease.**


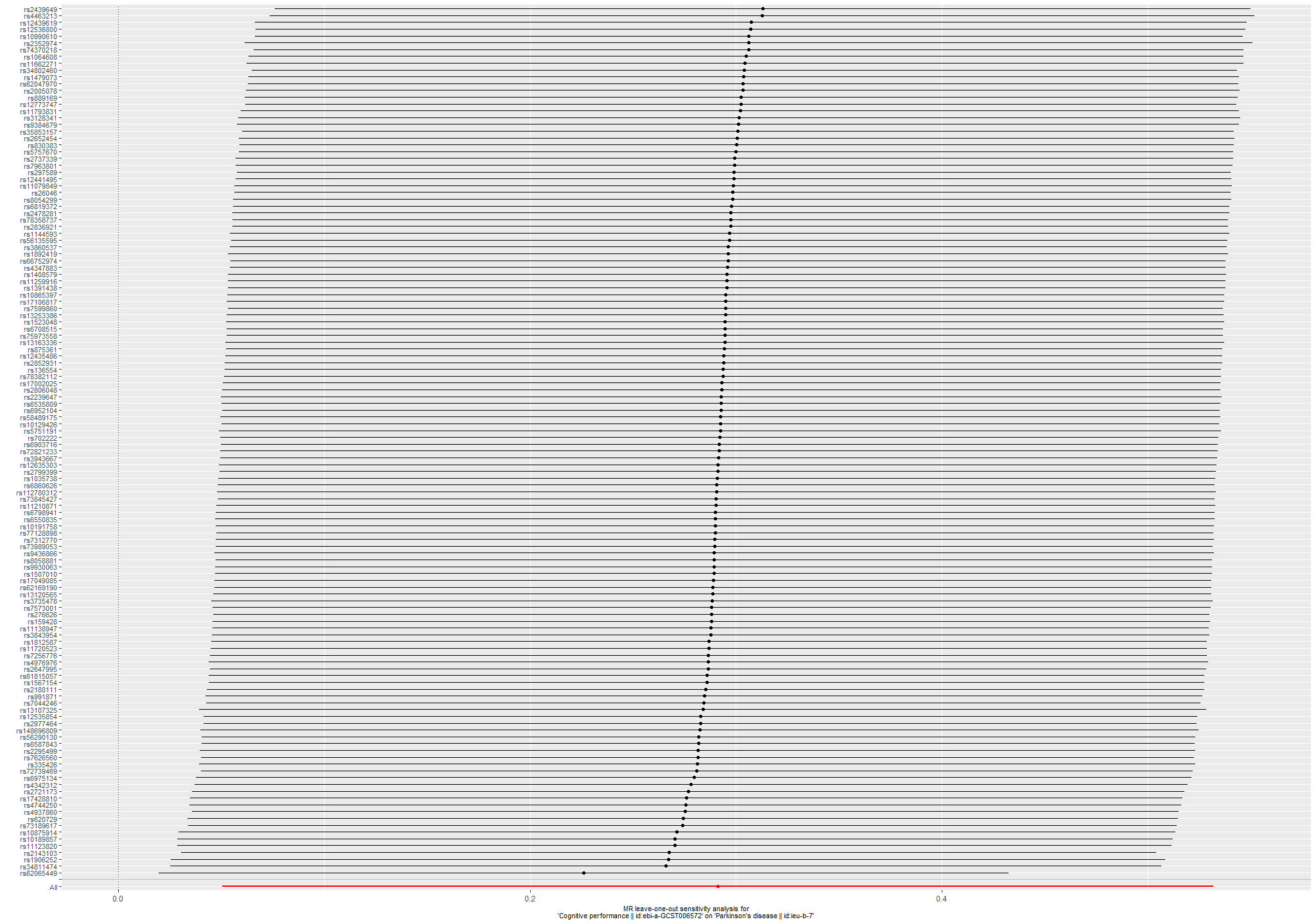


**Fig 27. Leave-one-out analysis of the association between cognitive (test) performance and Parkinson’s Disease.**


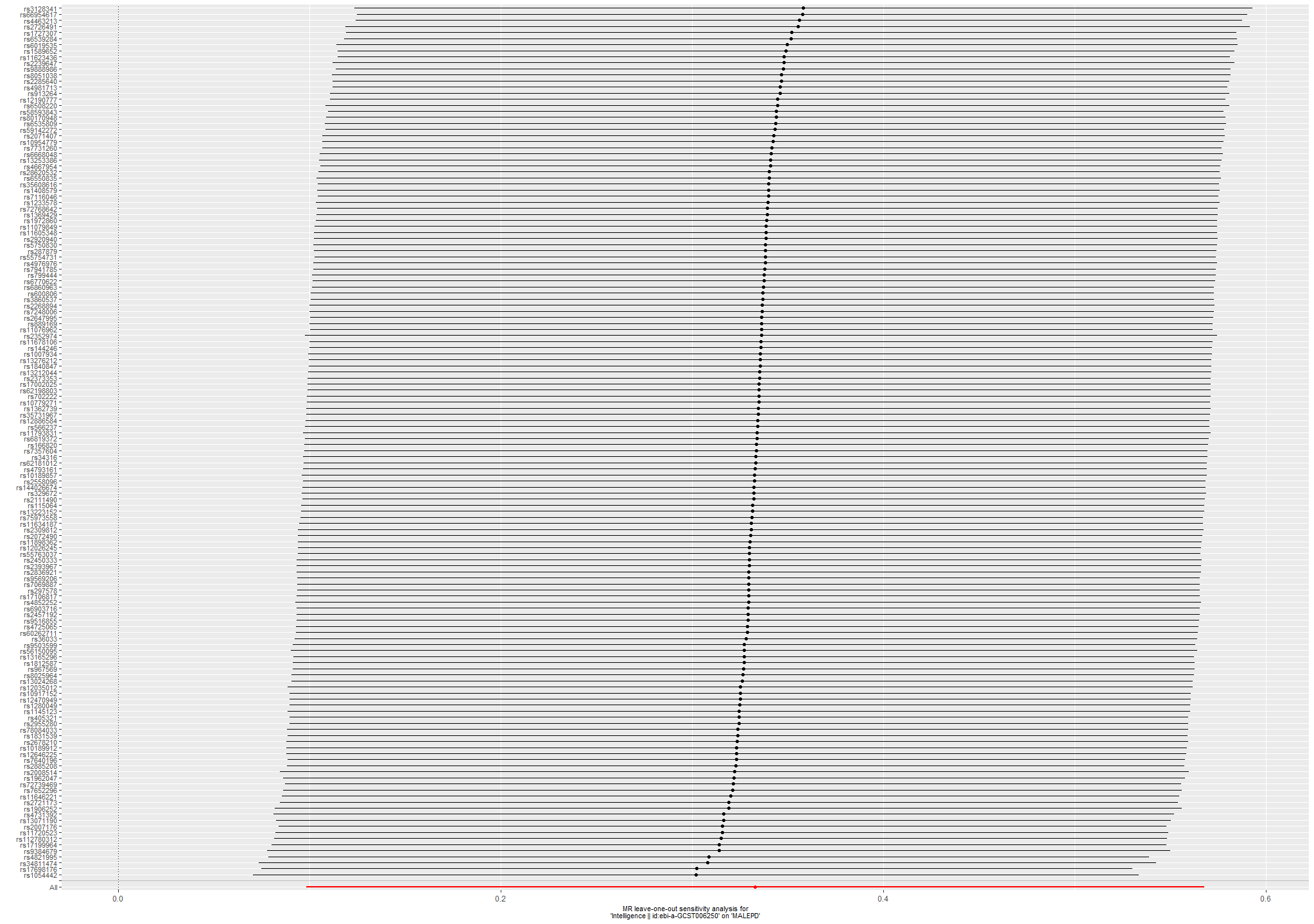


**Fig 28. Leave-one-out analysis of the association between intelligence and MALEPD.**


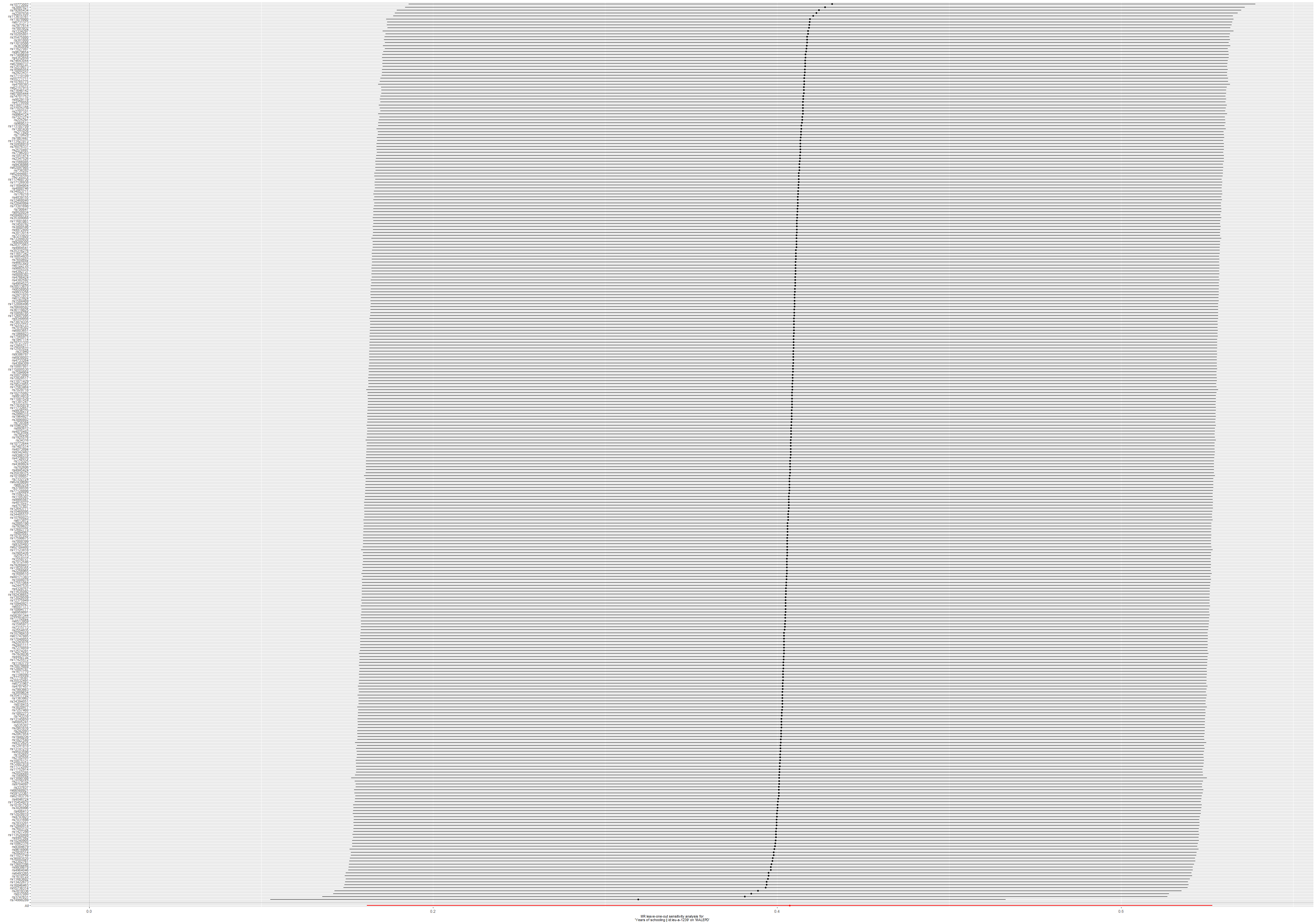


**Fig 29. Leave-one-out analysis of the association between educational attainment and MALEPD.**


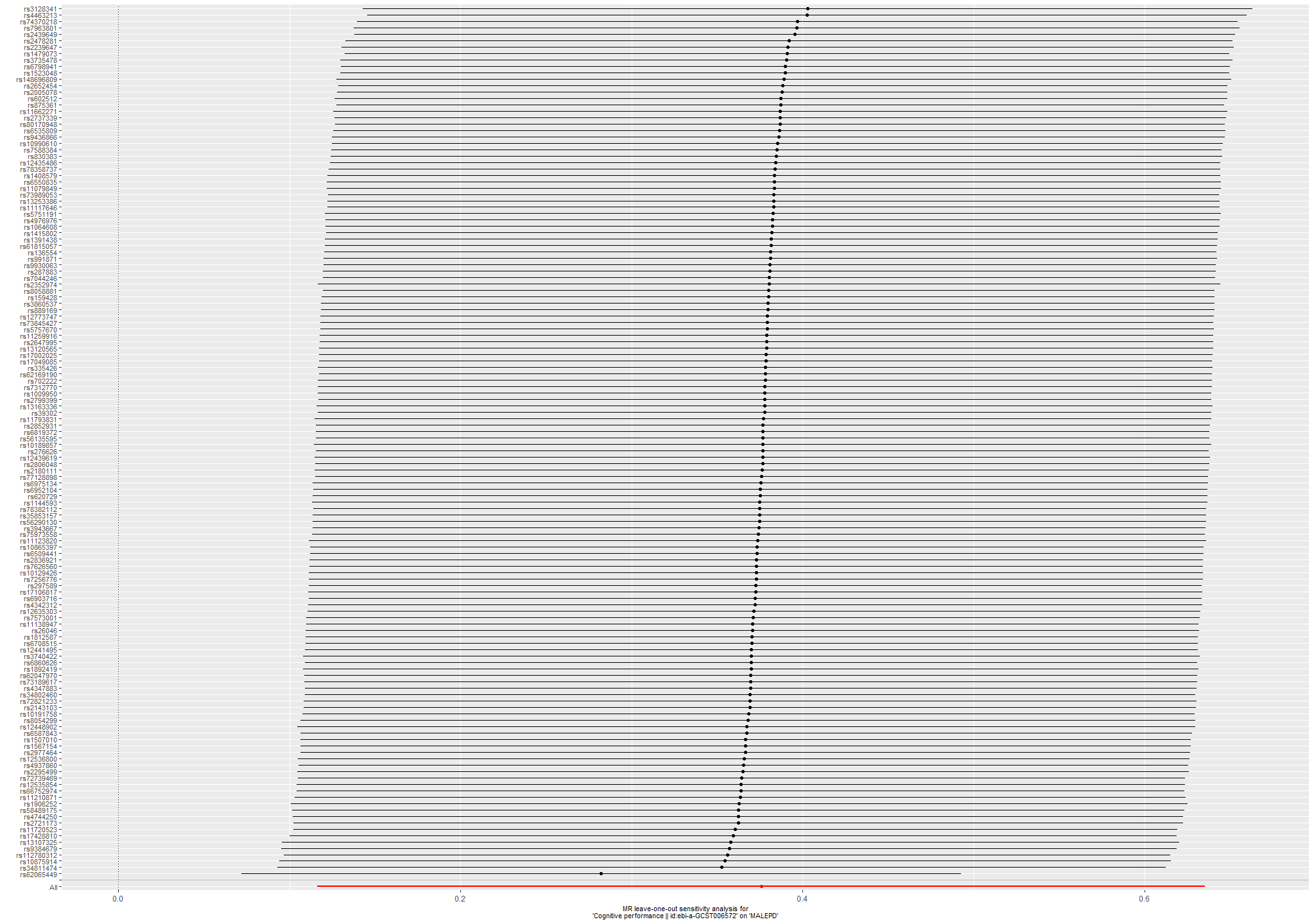


**Fig 30. Leave-one-out analysis of the association between cognitive (test) performance and MALEPD.**


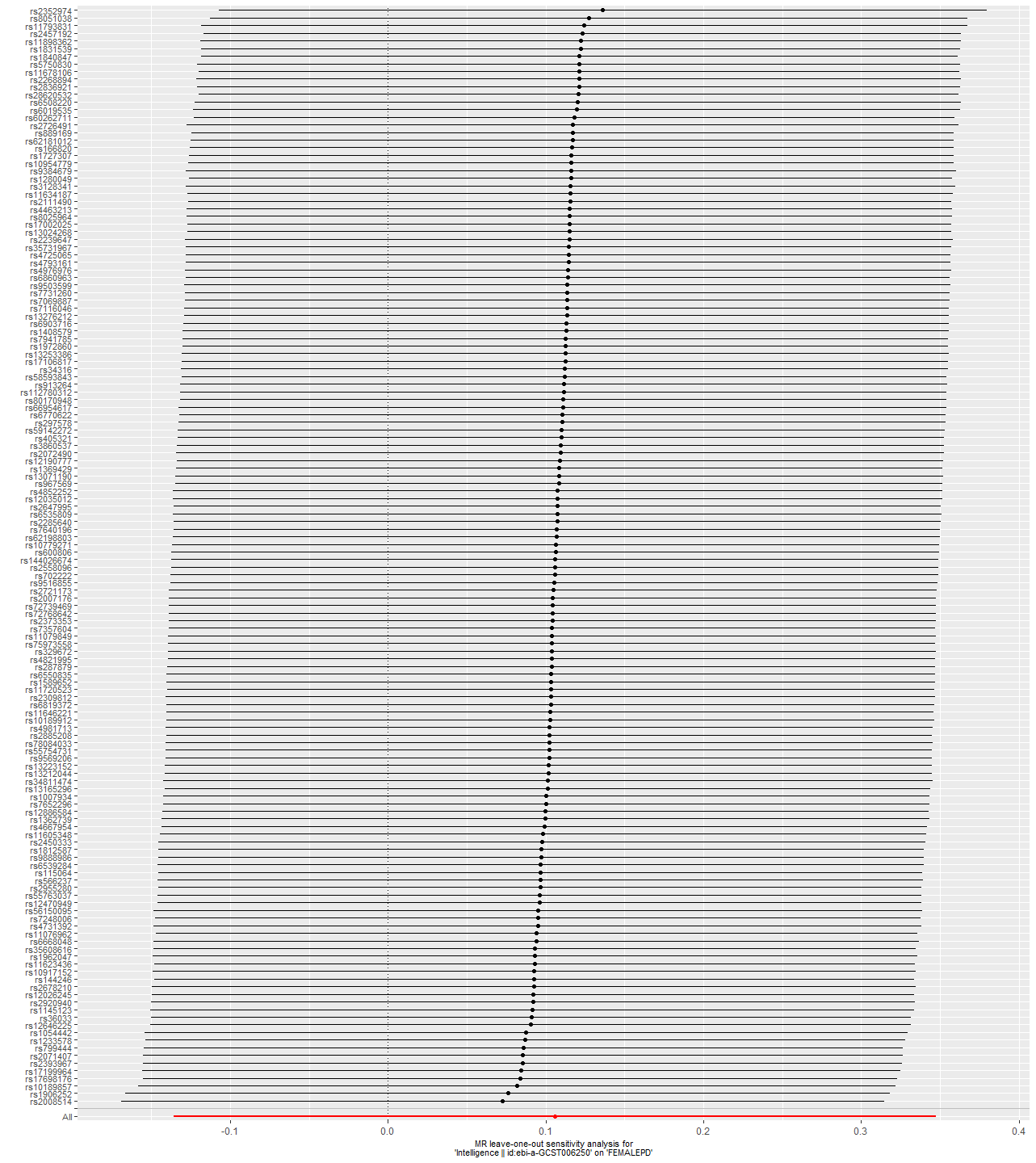


**Fig 31. Leave-one-out analysis of the association between intelligence and FEMALEPD.**


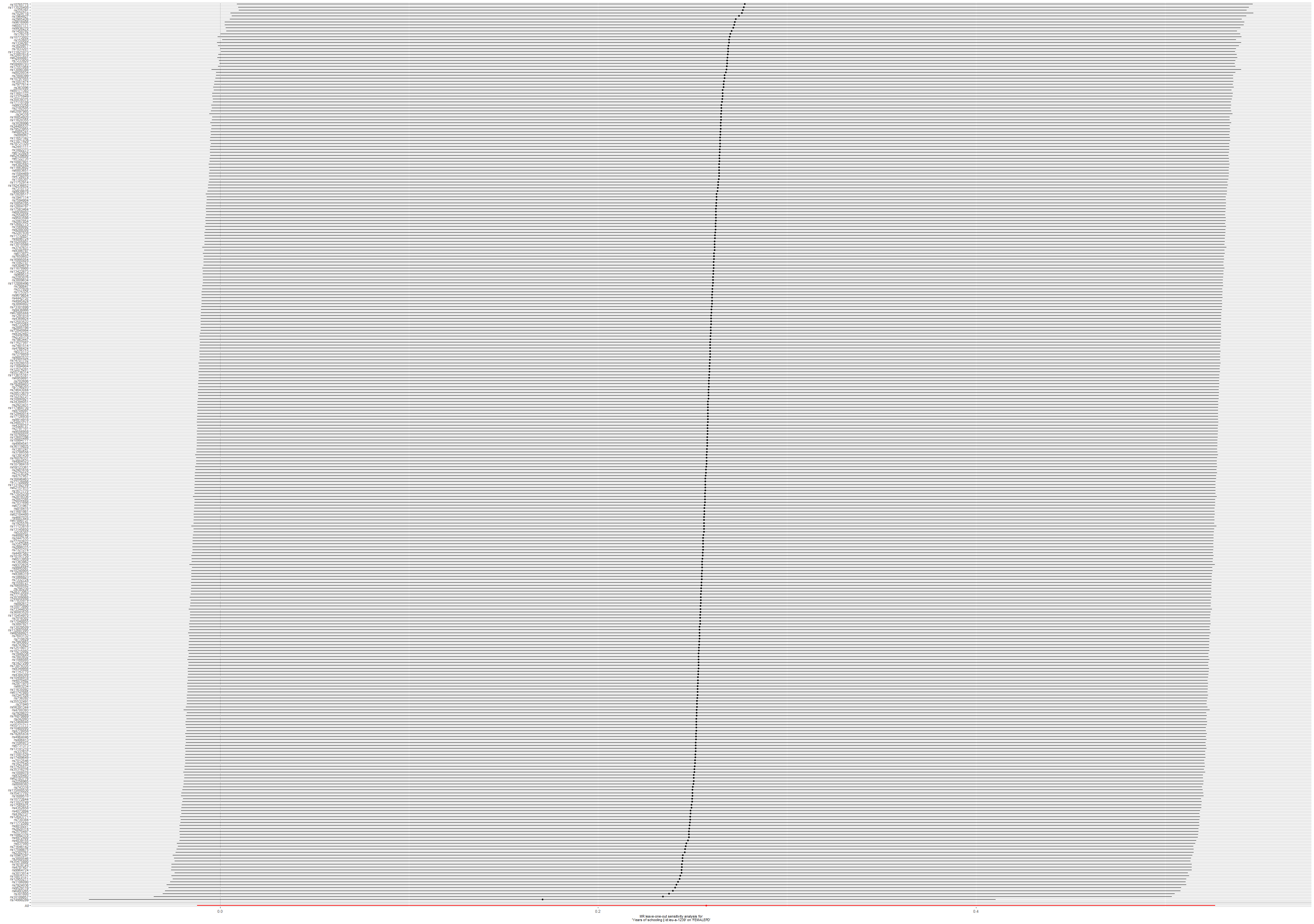


**Fig 32. Leave-one-out analysis of the association between educational attainment and FEMALEPD.**


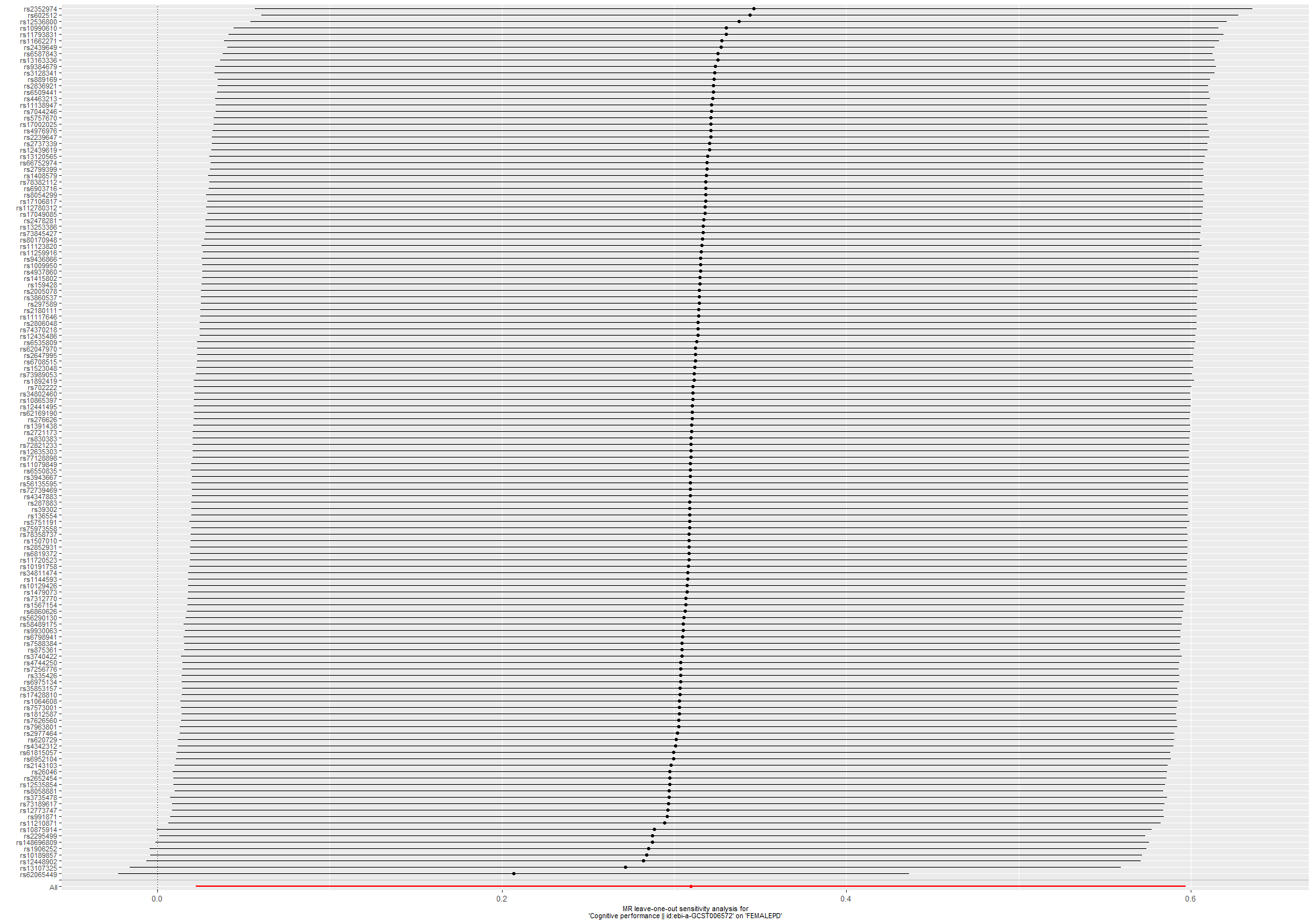


**Fig 33. Leave-one-out analysis of the association between cognitive (test) performance and FEMALEPD.**


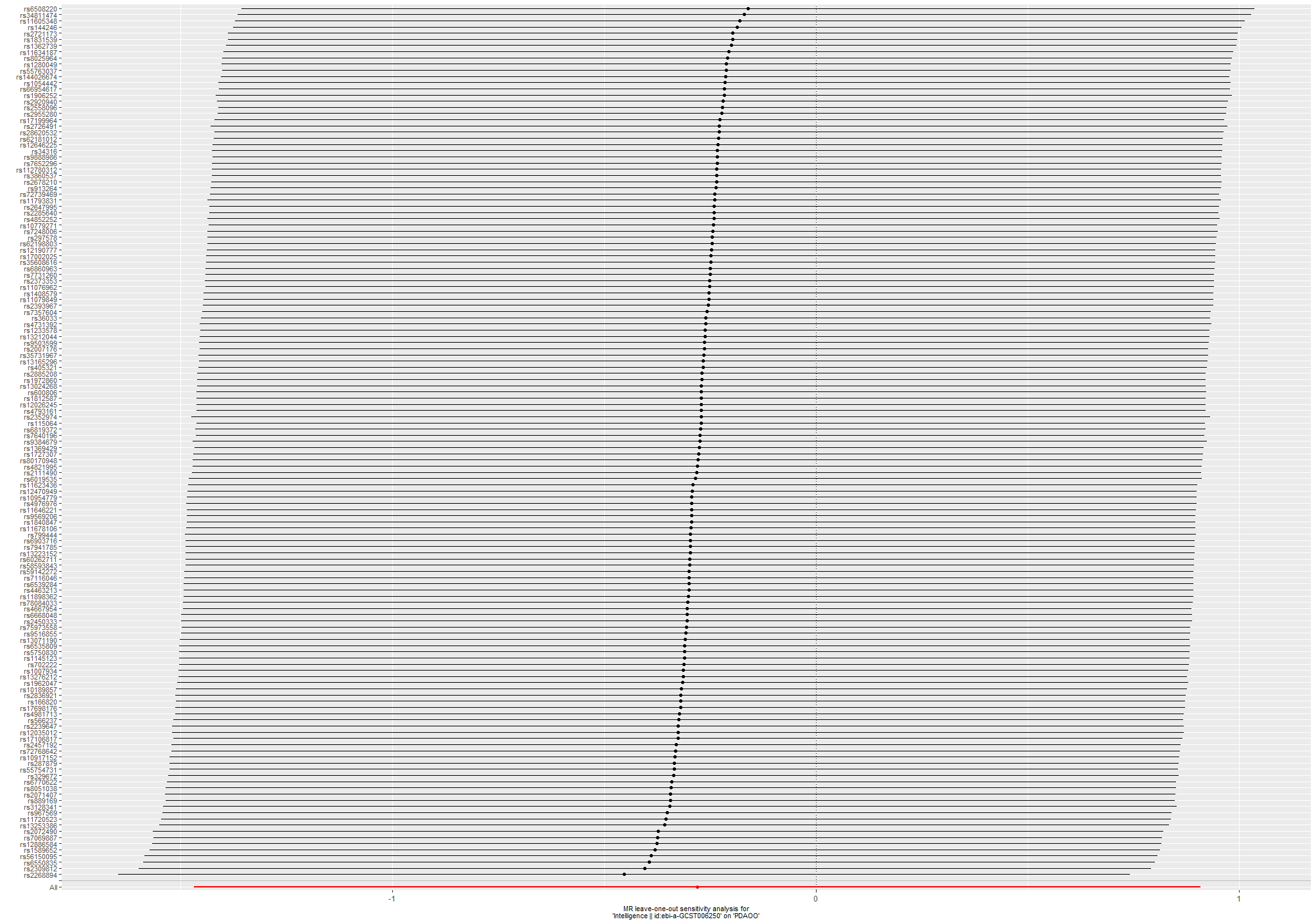


**Fig 34. Leave-one-out analysis of the association between intelligence and PDAOO.**


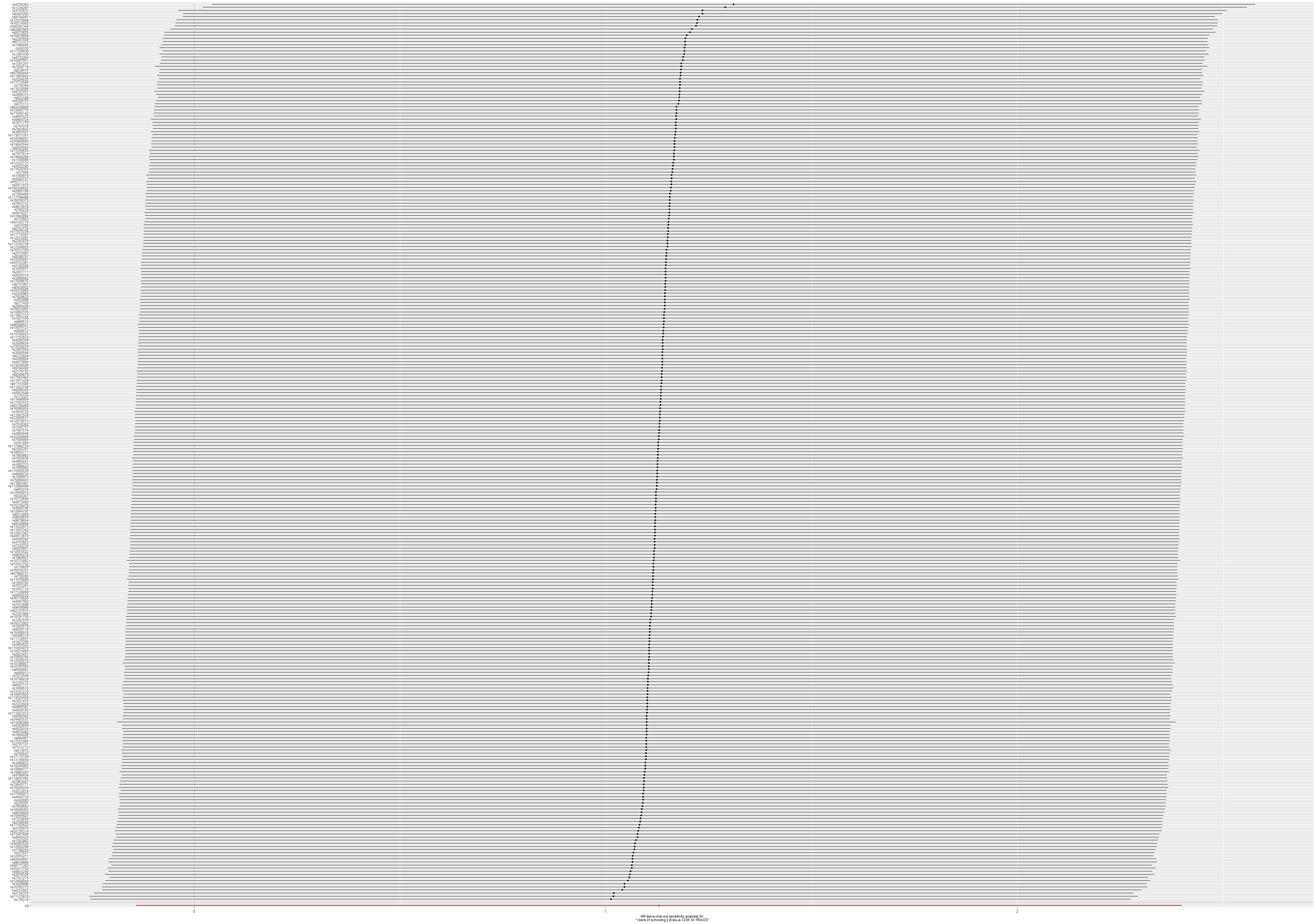


**Fig 35. Leave-one-out analysis of the association between educational attainment and PDAOO.**


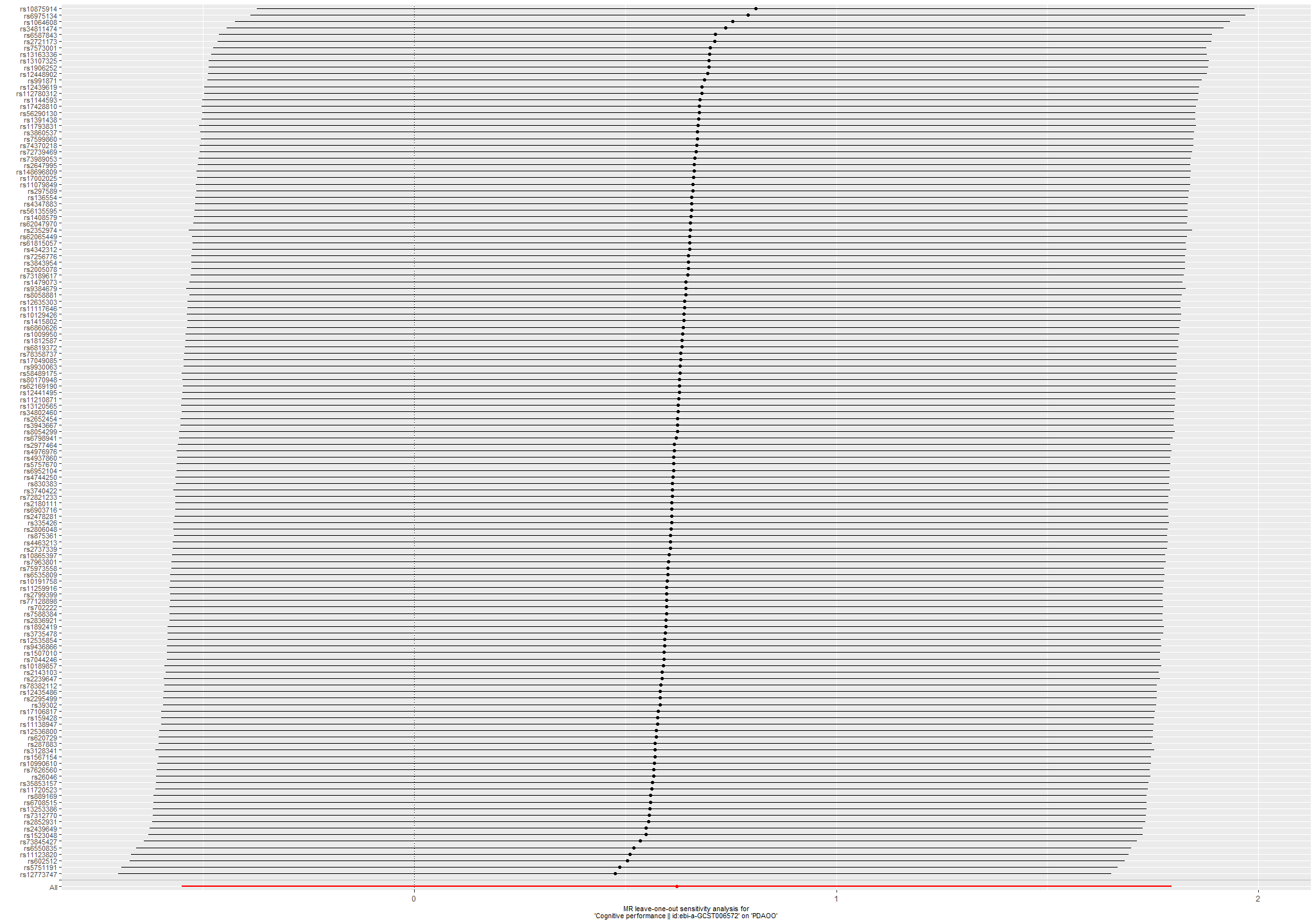


**Fig 36. Leave-one-out analysis of the association between cognitive (test) performance and PDAOO.**
